# Exploration of new superconductors and functional materials, and fabrication of superconducting tapes and wires of iron pnictides

**DOI:** 10.1088/1468-6996/16/3/033503

**Published:** 2015-05-08

**Authors:** Hideo Hosono, Keiichi Tanabe, Eiji Takayama-Muromachi, Hiroshi Kageyama, Shoji Yamanaka, Hiroaki Kumakura, Minoru Nohara, Hidenori Hiramatsu, Satoru Fujitsu

**Affiliations:** 1 Frontier Research Center, Tokyo Institute of Technology, Yokohama 226-8503, Japan; 2 Materials and Structures Laboratory, Tokyo Institute of Technology, Yokohama 226-8503, Japan; 3 Materials Research Center for Element Strategy, Tokyo Institute of Technology, Yokohama 226-8503, Japan; 4 Superconductivity Research Laboratory, International Superconductivity Technology Center (ISTEC), 2-11-19 Minowa-cho, Kohoku-ku, Yokohama, Kanagawa 223-0051, Japan; 5 National Institute for Materials Science, 1-2-1 Sengen, Tsukuba, Ibaraki 305-0047, Japan; 6 Department of Energy and Hydrocarbon Chemistry, Graduate School of Engineering, Kyoto University, Nishikyo-ku, Kyoto 615-8510, Japan; 7 Department of Applied Chemistry, Graduate School of Engineering, Hiroshima University, Higashi-Hiroshima 739-8527, Japan; 8 Department of Physics, Okayama University, Okayama 700-8530, Japan

**Keywords:** superconductivity, iron pnictide, new superconductors, superconducting wire, superconducting tape, functional material, powder in tube

## Abstract

This review shows the highlights of a 4-year-long research project supported by the Japanese Government to explore new superconducting materials and relevant functional materials. The project found several tens of new superconductors by examining ∼1000 materials, each of which was chosen by Japanese experts with a background in solid state chemistry. This review summarizes the major achievements of the project in newly found superconducting materials, and the fabrication wires and tapes of iron-based superconductors; it incorporates a list of ∼700 unsuccessful materials examined for superconductivity in the project. In addition, described are new functional materials and functionalities discovered during the project.

## 1. Introduction

Since the discovery of superconductivity by Heike Kamerling Onnes in 1911 [1], it has been the dream of researchers to realize a room temperature superconductor. Although the fundamental theoretical framework for superconductivity was established in 1957 by the Bardeen–Cooper–Schrieffer (BCS) theory, there exists no theory which can quantitatively predict the critical temperature (*T*_c_) even now [2]. Thus, looking for high *T*_c_ superconductors is like a voyage in a big ocean without a precise compass, i.e., researchers have to move ahead believing their sense and/or intuition referring to what theorists say. In this sense, looking for high *T*_c_ superconductors is a truly challenging subject and typical ‘all or nothing’ research.

It is a historical fact that materials leading to breakthroughs have been discovered in most cases by chance amidst concentrated research efforts undertaken with a unique but flexible view. This is particularly true for the exploration of new superconductors. The group of one of the present authors (HH) discovered superconductivity in LaFePO in 2006 [3] and LaFeAsO_1−*x*_F_*x*_ in 2008 [4] through LaNiPO (*T*_c_ = 3 K) [5] in 2007 in the course of exploring magnetic semiconductors, which started from his extensive research of transparent p-type semiconductors LaCuO*Ch* (where *Ch* = S and Se) with the same crystal structure as the so-called 1111-type layered compounds. P-type conduction in LaCuO*Ch* originates from the mobile holes at the top of the valence band which is composed of *Ch* p orbitals and Cu 3*d* orbitals [6]. It was his idea to novel magnetic semiconductors to utilize strong *d–p* interactions in LaCuO*Ch* by replacing the nonmagnetic Cu^+^ ion with a magnetic 3*d* transition metal cation with a + 2 charge state. In order to keep electro-neutrality upon this substitution, *Ch*^−2^ is required to be replaced by *Pn*^3−^ [7]. This is the reason why his group started to examine the electronic and magnetic properties of La*TM*O*Pn* (where *TM* = 3*d* transition metal, *Pn* = P and As). This effort resulted in the discovery of iron-based superconductors (IBSCs) through the concentrated effort to find high performance p-type transparent semiconductors, which is a branch of his research home ground, transparent oxide semiconductors [8].

The discovery of IBSCs was accepted with surprise by the condensed matter community because iron, with a large magnetic moment, was widely believed to be most harmful to the emergence of superconductivity. Extensive research into these materials started globally, particularly in China [7]. As a result, the discovery of IBSCs was chosen as the breakthrough of the year in 2008 by *Science Magazine* and the paper [4] reporting *T*_c_ = 26 K in LaFeAsO_1*−x*_F_*x*_ became the most cited report among all the original research papers published in 2008.

In early 2009, the Japanese Government announced the launch of a new large funding program—FIRST (**F**unding Program for World-Leading **I**nnovative **R**&D on **S**cience and **T**echnology). The aim of the FIRST Program is to advance the kind of leading-edge research and development that will strengthen Japan’s international competitiveness while contributing to society and people’s welfare through the application of its results. Hideo Hosono’s proposal ‘exploration for novel superconductors and relevant functional materials, and development of superconducting wires for industrial applications’ was selected as one of 30 projects out of ∼800 applications covering a very broad area of science and technology.

It was his expectation to find novel functionalities and materials with high potential through this tough and really challenging work just as IBSCs were found though the exploration of magnetic semiconductors. Hideo Hosono organized the research team to be mainly composed of solid state chemists who have experience and achievements not only in superconductors, but also in the relevant functional materials. Since research in finding new superconductors typically belongs to the domain of condensed matter physics, this team organization is a unique feature of this project. It was his belief that excellent solid state chemists will find new properties by serendipity, even if they fail to succeed in the hunt for new high *T*_c_ materials. This philosophy was set at the beginning of the project, i.e., ‘All or something!’.

This article reviews the major research achievements obtained in our FIRST Project performed over the 4 years from March 2010 through March 2014, along with some background for the research. We have examined more than 1000 materials to seek new superconductors. The fraction of success was relatively small (∼3%), but just as we expected at the outset. So far, unsuccessful results in this field have not been presented in an academic journal. In this review, we have listed the records of materials that we examined in this project, including the unsuccessful materials, based on a consensus among the members of the research team that the consideration of unsuccessful trials will be good fertilizer for future research.

## 2. Overview

At the beginning of this project, Hosono laid down five research targets. These were: (1) the discovery of a new superconductor with *T*_c_ > 77 K, (2) the development of new superconductors with high performance, (3) the development of related materials with outstanding functions, (4) the development of meter-class superconducting wires with a critical current density *J*_c_ > 10^5^ A cm^−2^ based on IBSCs or other novel materials, and (5) the production of prototype Josephson junction and SQUID devices using IBSC thin films. This project consists of six research groups as listed in table 1. Four groups (HH, EM, HKa and SY) have concentrated on the exploration of new superconductors, and the other two groups (KT and HKu) have concentrated on the development of superconducting wires and tapes. The HH group collaborated with five other groups in the field to look for superconductors (MN) and the discovery of catalysis using electride (MH, TS, AS and SK). The HKu group collaborated with YK in the field of superconducting wires. Each group has made an effort to achieve its purpose using its special skills.

**Table 1.** Organization of FIRST Hosono Project.

	Team Leader	Mission
Exploration of Superconductors	Hideo Hosono (Core-Researcher) (Tokyo Institute of Technology)	Exploration of superconductors of transition metal compounds with layered structure collaborated with Minoru Nohara (Okayama University)
		Development of superconducting device by thin film technique
		Development of novel functional materials (especially C12A7 electride) collaborated with Michikazu Hara (Tokyo Institute of Technology), Tohru Setoyama (Mitsubishi Chemical Corporation), Alex Shluger (University College London) and Sung Wng Kim (Sungkyunkwan University)
	Eiji Takayama-Muromachi (National Institute of Materials Science)	Exploration of superconductors using high pressure synthesis technique and their characterization
	Hiroshi Kageyama (Kyoto University)	Exploration of superconductors using low temperature synthesis technique and their characterization
	Shoji Yamanaka (Hiroshima University)	Exploration of superconductors with layered or clathrate structure and their characterization
Development of Superconducting Wire	Keiichi Tanabe (Sub-Core-Researcher) (International Superconductivity Technology Center	Development and evaluation of superconducting wire and superconducting device prepared by thin film technique
	Hiroaki Kumakura (National Institute of Materials Science)	Development and evaluation of superconducting wire prepared by PIT method collaborated with Yoichi Kamihara (Keio Universty)

Though the FIRST Project did not discover a new superconductor with *T*_c_ > 77 K (56–58 K maximum), over 100 new superconductors have been developed and characterized as new types of IBSC (112) have been found, a new dopant into IBSC (H^−^) has been employed to induce superconductivity, intercalation type compounds have been found, cobalt-based and titanium-based superconductors have been found, etc. The search for new superconductors is the most important target of the FIRST Project, and over 40 researchers in four groups have worked on this mission. They have examined more than 1000 materials to seek new superconductors. The number of new superconductors found is a relatively small percentage, as envisioned at the onset, which is part of the motivation for the extensive search. We believe that listing all materials examined, including both successes and failures, is meaningful for the people who work in this field or will join this field in the future, and thus show these in table 2. The details of some representative results will be described in section 3.

**Table 2.** Exploration of new superconductors in this project. (a) Materials which exhibited superconductivity. (b) Materials which exhibited no superconductivity.

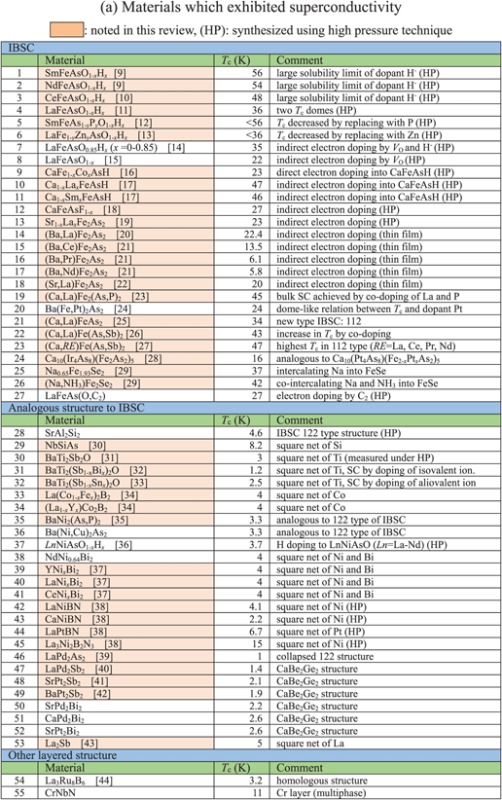
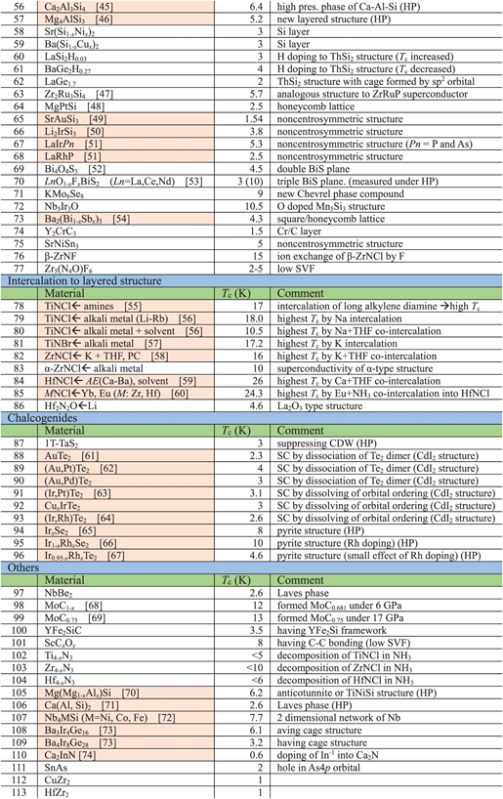
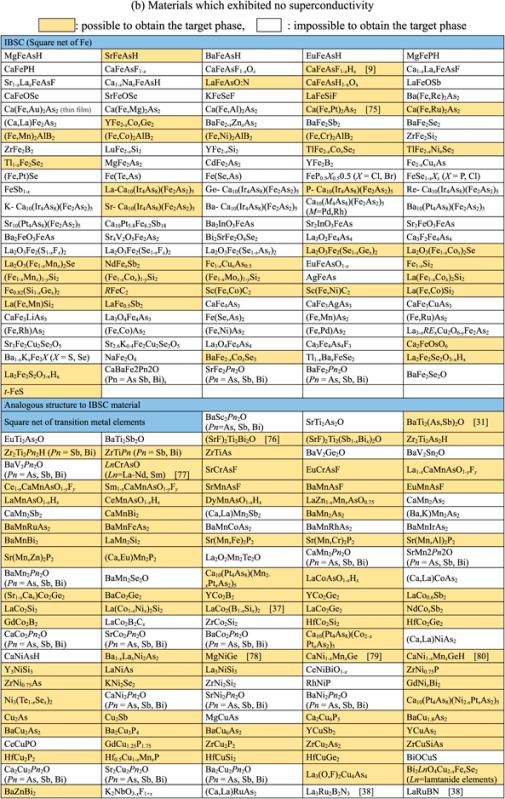
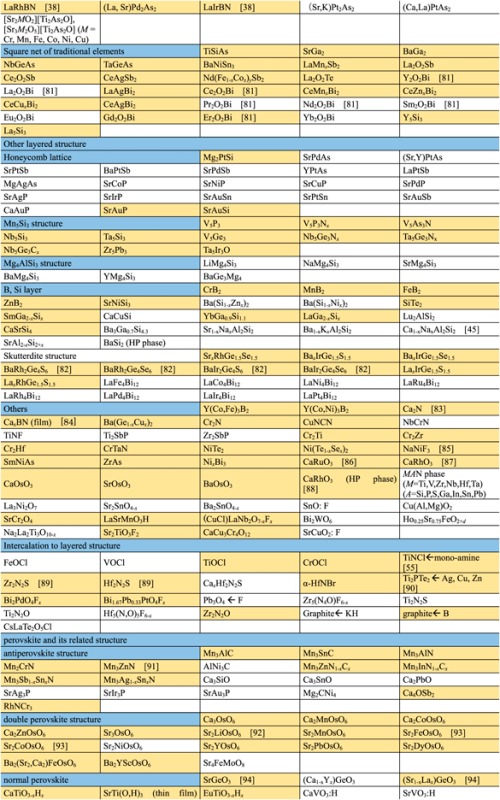
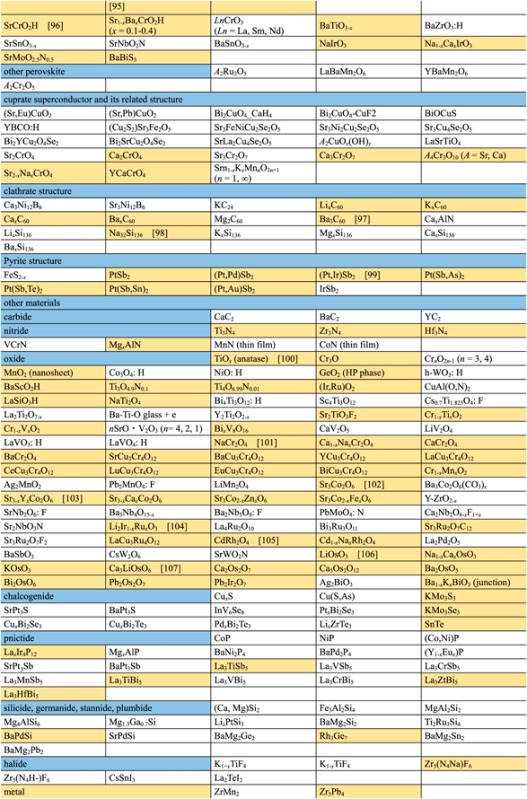

This project produced superconducting wires and tapes with *J*_c_ > 10^5^ A cm^−2^ by the powder-in-tube (PIT) method and has developed efficient magnetic pinning centers for thin film type wires and tapes. Furthermore, the FIRST Project has succeeded in preparing Josephson junction and SQUID devices by using epitaxial thin films of IBSCs, clarifying the physical properties of IBSCs including small anisotropy and high durability in magnetic fields. The induction of a metallic state from an insulating parent material of IBSCs by the electrostatic method was also a result of the research on IBSC thin film devices. We will describe these details in section 4.

The new functional materials developed in the project are rather diverse. The discovery of a highly efficient catalyst for ammonia synthesis, the 12CaO·7Al_2_O_3_ (C12A7) electride, is the most remarkable result [108], which has an impact not only in the academic community, but also in industry. The discovery of the spontaneous decomposition of carbon dioxide gas on the C12A7 electride surface [109], the preparation of stable perovskite titanium oxy-hydride [110], the development of a bipolar oxide semiconductor and its complementary circuit device [111], the development of a new class transparent oxide conductor, SrGeO_3_ [94], the discovery of the first Slater insulator, LiOsO_3_, which is a ferroelectric metal [106], the development of a material showing a new type of giant magnetoresistance, NaCr_2_O_4_ [94], and the discovery of the two-dimensional electride properties in Ca_2_N [83], are also representative results. It is interesting that some of these harvests have resulted in the exploration of new superconductors. These results are to be introduced briefly in section 5.

This project has reported these results in more than 330 original papers and numerous oral and poster presentations including over 170 invited and plenary talks at international meetings. Researchers from the FIRST Project have also applied for over 30 patents.

## 3. New superconductors

In the FIRST Project, four research groups have concentrated on exploring and evaluating novel superconductors, and contributed to the progress of the research of superconductivity through discovery of many novel superconductors and phenomena. We will review these results.

### 3.1. Iron-based superconductors

The history of IBSCs started in 2006 when LaFePO with *T*_c_ = 5 K was found by Hosono’s group [3]. Only a few researchers took notice of this new type of superconductor based on iron with a large magnetic moment. In 2008, the discovery of superconductivity in LaFeAsO_1*−x*_F_*x*_ with *T*_c_ = 26 K by the same group [4] rekindled global interest in this area and opened a new frontier of superconductivity. At the early stage, this superconductor family was called ‘pnictide superconductors’. However, researchers now call them ‘iron-based superconductors’ because several measurements and evaluations have clarified that they all have a similar electronic structure where the 3*d* electrons derived from Fe ion dominate the Fermi level to play a primary role in superconductivity.

Magnetism had long been believed to be incompatible with superconductivity. Thus, the use of elements with a large magnetic moment, typically Fe, Ni and Co, was intentionally avoided in the field of superconductivity. Hence, the discovery of a high-*T*_c_ superconductor based on iron impacted research in this field. Condensed matter scientists encountered a new frontier of superconducting materials.

Immediately after Hosono’s group reported an increase in *T*_c_ (to 43 K) for La FeAs O_0.89_F_0.11_ under high pressure [112], two groups in China [113–115] reported a higher *T*_c_ (=55 K) for SmFeAsO_1*−x*_F_*x*_ under ambient pressure. By now, it is reported that the highest *T*_c_ values (55–58 K) in non-cuprate bulk superconductors are observed for some IBSCs as SmFeAsO_0.74_F_0.26_ [116], SmFeAsO_0.85_ [117], SmFeAsO_0.8_H_0.2_ [9], and Gd_0.8_Th_0.2_SmFeAsO [118].

In 2008, Johrendt’s group in Germany reported (Ba,K)Fe_2_As_2_ (*T*_c_ = 38 K) [119]. Thanks to the ease of growing its single crystals, which have a lateral size of several millimeters using Sn or FeAs as a flux, its physical properties have been well elucidated. In addition, researchers discovered several types of IBSCs and characterized their superconducting properties [120–127].

In this section, we review the recent progress of IBSCs focusing on the results of this project.

#### 3.1.1. Features of IBSCs

##### 3.1.1.1. Crystal structure

**Figure 1.**
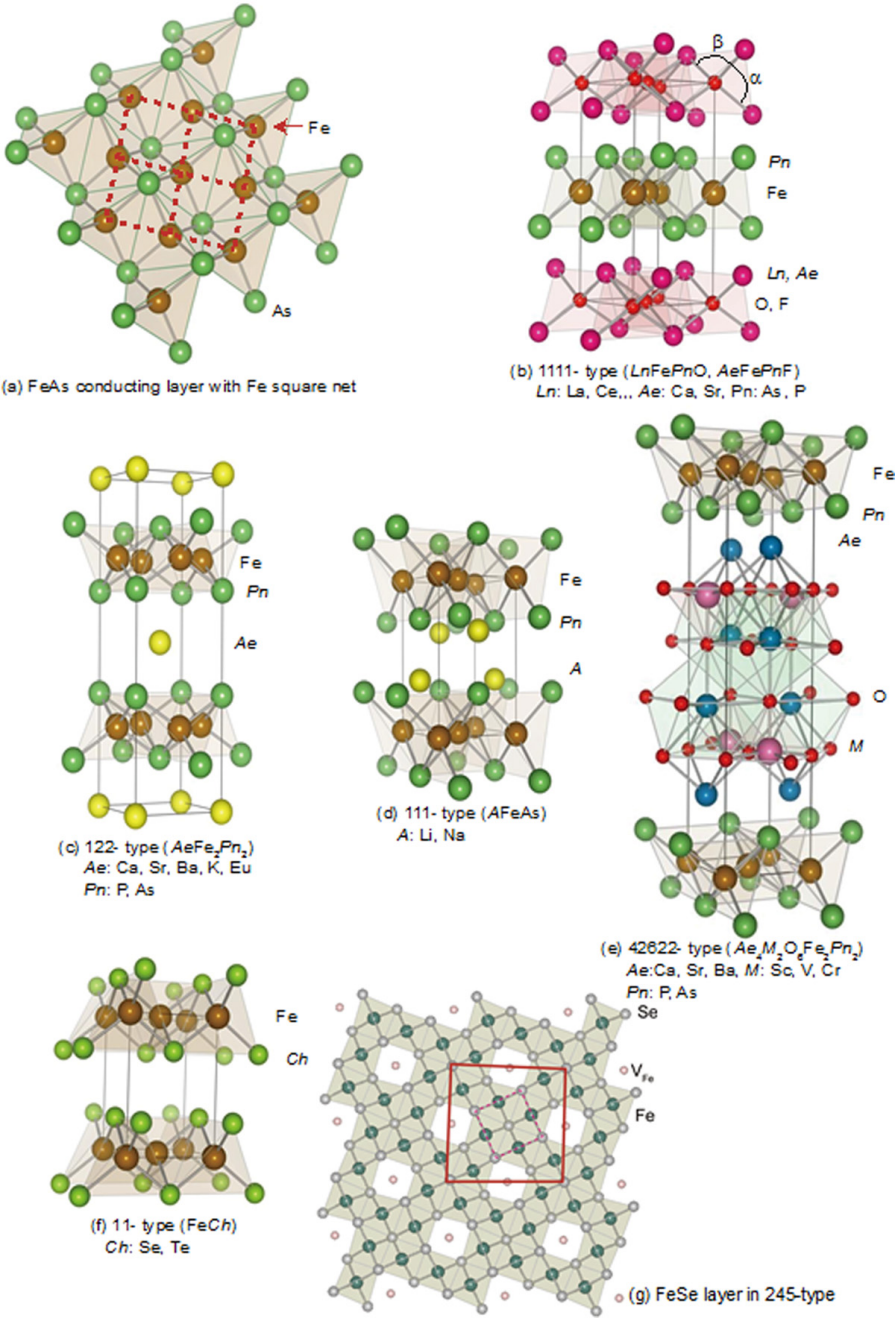
Crystal structures of iron based superconductors. (a) Structure of the FeAs conducting layer, which is common to all IBSCs. The dotted line indicates the Fe square net. (b) 1111-type, (c) 122-type, (d) 111 type (e) 42622-type and (f) 11-type structures. (g) Structure of K_0.775_Fe_1.613_Se_2_ from the [001] direction in the 5^1/2^ × 5^1/2^ × 1) cell showing fully occupied Fe sites decorated with ordered vacancy sites. The dotted square indicates the basal plane of a 122-type unit cell. Reprinted with permission from [125]. Copyright 2012 by Maney Publishing.

Although approximately 100 IBSCs have been reported, their parent materials may be classified into seven types in terms of crystal structures (figure 1). These materials contain a common structural unit of the Fe*Pn* (or Fe*Ch*) layer formed by the square net of Fe^2+^ (as the formal charge), which is tetrahedrally coordinated by four pnictogen (*Pn*) and/or chalcogen (*Ch*) atoms (see figure 1(a)). Unlike cuprate superconductors, where the parent materials are Mott insulators, this layer shows metallic conductivity without doping. An insulating blocking layer is composed of *M*, *M*O or *M*F etc, where *M* indicates a metallic element such as an alkali, alkaline earth, or rare earth metal that lies between Fe*Pn* (or Fe*Ch*) layers. Similar to cuprates, this layered structure provides quasi-two-dimensional carrier transport properties, although the magnitude of anisotropy rather differs depending on the blocking layer. The local structure of the Fe*Pn* layer is affected directly by the atomic (or ionic) size of *M* because *M* elements in the blocking layer bond to Fe elements. The crystal structures and the brief introductions of the seven different parent materials for IBSCs are described below.

(i) *1111-type materials (LnFePnO, Ln: lanthanide, AEFeAsF, AE: alkaline earth, Pn: P, As)*.

1111-type compounds have the same structure as LaFeAsO, and are the prototype version of IBSCs. Due to their atom composition ratios, they are called ‘1111-type’. Figure 1(b) shows their crystal structure, which is a ZrCuSiAs-type structure [128, 129] with a tetragonal *P4*/*nmm* space group. Although LaFePO and LaFeAsO, along with their crystal structures, were identified by Zimmer *et al* 20 years ago [130], they were discovered to be superconductors in 2006 [3] and 2008 [4], respectively. Moreover, their two-dimensionality is relatively high among the seven types, and only this group has *T*_c_ values above 50 K as a bulk form.

The 1111-type compound is composed of an alternating stack of positively charged *Ln*O layers and negatively charged FeAs (or FeP) layers along the *c*-axis. As mentioned above, the local structure of the FeAs layer is the same in all types of IBSCs. The distance between the FeAs layers corresponds to the length of the *c*-axis (∼0.8–0.9 nm). The formal valence state of each atom is *Ln*^3+^, Fe^2+^, As^3−^, and O^2−^. Fe^2+^ contains six electrons in its 3*d* orbital, and these electrons play an essential role in driving the superconductivity and magnetism. The electronic and magnetic properties of La*T*_*M*_*Pn*O (*T*_*M*_: 3*d* transition metal (Cr–Zn)) are summarized in table 3 [120]. The 1111-type compounds composed of Fe and Ni reveal superconductivity.

**Table 3.** Summary of electromagnetic properties of La*T*_*M*_*Pn*O. (*T*_*M*_: 3*d* transition metal, and *Pn*=P or As.)

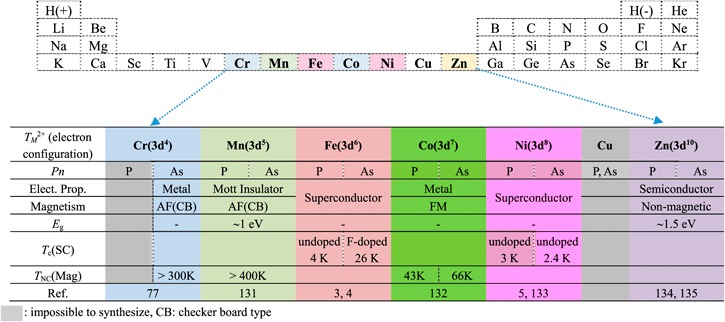

The lanthanide elements from La to Gd can occupy the *Ln* site for the 1111-type of material with *Pn* = P [130]. In the case of *Pn* = As, La to Ho and Y can also occupy the *Ln* site [136]. Additionally, Ca(Fe_1*−x*_Co_*x*_)AsF (*T*_c_ = 22 K) is a fluoride-containing superconductor of this type [138].

In this project, the effect of a hydride ion as a new electron donor to this type was studied earnestly and the results are described in section 3.1.2.

(ii) *122-type materials* (*MFe*_*2*_*Pn*_*2*_*, M: alkali, alkaline earth or Eu*)).

122-type materials have a ‘ThCr_2_Si_2_’ type crystal structure with a tetragonal *I*4/*mmm* space group [137]. This group contains the largest number of compounds among the five parent families.

In the case of *AE*Fe_2_P_2_, not only alkaline earth elements but also lanthanides (La–Pr, Eu) can occupy the *AE* site. In *AE*Fe_2_As_2_, the *AE* site can be occupied by alkaline earth, alkali metal, or Eu^2+^. Figure 1(c) shows the crystal structure of the 122-type. The layer composed of *AE* ions, which is thinner than the *Ln*-–O layer of 1111-type, is sandwiched between the FeAs conducting layers. The distance between the FeAs layers of 122-type (0.5–0.6 nm) is shorter than that of 1111-type (0.8–0.9 nm). Because the nearest FeAs layers face each other with a mirror plane, the lattice parameter *c* is twice the FeAs–FeAs distance. The lattice parameter *a* (∼0.4 nm) is almost the same as that of 1111-type. Consequently, both 1111 and 122-type materials have similar Fe–Fe distance in the FeAs layer. Since single crystals of several millimeters can be obtained using Sn or FeAs as a flux, the physical properties of 122-type are well evaluated compared to other types of IBSCs. Johrendt’s group of Germany was the first to report superconductivity for 122-type materials [119].

In this project, lanthanide element doped 122 superconductors were prepared and evaluated in their bulk and thin-film forms, and these results are described in section 3.1.3.

(iii) *111-type materials* (*AFePn, A: alkali metal*).

While an *AE* ion (alkaline earth ion with formal charge of 2+) is alternately sandwiched between Fe*Pn* layers in 122-type, 111-type compounds contain two *A* ions (*A*: Li^+^, Na^+^) between Fe*Pn* layers in a unit cell. The crystal structure of this type is known as ‘CeFeSi’ type, with a tetragonal *P*4/*nmm* space group (figure 1(d)). This type is compatible with the structure of 1111-type where all the oxygen atoms are removed, and the *Ln* site is occupied by Li^+^ or Na^+^. Wang *et al* [139] (*T*_c_ = 18 K: LiFeAs) and Parker *et al* [140] (*T*_c_ = 10 K: NaFeAs) first reported superconductivity for 111-type materials.

(iv) *Materials with thick blocking layer* (32522-type (*AE*_3_*M*_2_O_5_Fe_2_*Pn*_2_, *M*: Al, Sc)), (42622-type (*AE*_4_*M*_2_O_6_Fe_2_*Pn*_2_, *M*: Sc, V, Cr)), (homologous type (Ca_*n*+1_Sc_*n*_O_*y*_ Fe_2_As_2_: *n* = 3, 4, 5)).

The distance between the Fe*Pn*/*Ch* layers is in the order of the 1111, 122, 111 and 11-types. In contrast, these three types of iron oxy-pnictide have a thick blocking layer composed of a quasi-perovskite structure assembled by *M*O_5_ pyramids and *AE* (see figure 1(e) for the 42622-type (Sr_4_Sc_2_O_6_Fe_2_As_2_)). The FeAs–FeAs distance is 1.55 nm and 2.45 nm for Sr_4_Sc_2_O_6_Fe_2_As_2_ and Ca_6_(Sc_0.4_Ti_0.6_)_5_O_*y*_Fe_2_As_2_, respectively. The groups of Kishio and Shimoyama have studied these types of materials systematically [141–143]. The highest *T*_c_ reported so far is 43 K [143]. Considering the thick blocking layer, this type should have the highest two-dimensionality, but the concrete value of anisotropic properties has not yet been reported because of difficulty of single crystal growth. The 32522-type has been proposed as a promised parent material [144, 145], and the emergence of superconductivity in the 32522-type was reported by Shirage *et al* in 2011 for (Ca_3_Al_2_O_5−*y*_)(Fe_2_*Pn*_2_) (*Pn* = As (*T*_c_ = 30.2 K) and P (*T*_c_ = 16.8 K)) [146].

(v) *Materials containing additional arsenic* (Ca_1*−x*_La_*x*_FeAs_2_), (Ca_10_(*M*_4_As_8_)(Fe_2_As_2_)_5_, (*M*: Pr, Ir)).

These new types of iron pnictide superconductor were found by Nohara’s group of this project. The details for (Ca_1*−x*_La_*x*_FeAs_2_) (*T*_c_ = 43 K) and (Ca_10_(*M*_4_As_8_)(Fe_2_As_2_)_5_, (*M*: Pr, Ir)) (*T*_c_ = 38 K) are described in sections 3.1.4 and 3.1.5, respectively.

(vi) *11-type materials* (*Fe*_1*+x*_*Ch, Ch: Se, Te*).

The 11-type crystal has the simplest structure among the parent compounds and is essentially the alkali metal-free 111-type. This crystal structure is known as ‘*α*-PbO’ type with a tetragonal *P*4/*nmm* space group (figure 1(f)). A typical 11-type superconductor is *β*-FeSe (*T*_c_ = 8 K) [147]. Medvedev *et al* reported the 11-type may exhibit a high *T*_c_ (=37 K) under 8.9 GPa [148].

Furthermore, FeSe attracts attention as one of the candidates showing higher *T*_c_ than boiling temperature of liquid N_2_. Several groups in China reported that the monolayer of FeSe deposited on a SrTiO_3_ substrate showed high *T*_c_ (65 K) in 2012 and they raised *T*_c_ to 100 K [149–153]. Though this superconductivity emerges so far only for monolayers of FeSe deposited on a SrTiO_3_ substrate, a new route to high *T*_c_ materials is expected to be found.

(vii) *245-type materials* (*A*_1*−x*_*Fe*_2*−y*_*Se*_*2*_*: A* = *K*, *Cs*, *Rb*, *Tl*).

In 2010, Guo *et al* reported a potassium-intercalated iron selenide superconductor with relatively high *T*_c_ value (30 K) [154]. The crystal structure changed from 11 to quasi-122-type upon intercalation, of which the space group is assigned to *I*4/*m* due to vacancy ordering as shown in figure 1(g). Though Guo *et al* noted its chemical notation as K_*x*_Fe_2_Se_2_, the detailed chemical and structural analyses for its optimal material showed the composition to be *A*_0.8_Fe_1.6_Se_2_ (=*A*_2_Fe_4_Se_5_) and ordering of Fe vacancies with a 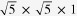 supercell in the 122-type crystal structure [155]. This type of material shows a wide range of non-stoichiometry, and with low Fe concentration is an antiferromagnetic insulator. The superconductivity in A_*x*_Fe_*y*_Se_2_ emerges in the proximity of an antiferromagnetic (AFM) Mott insulating state, similar to the cuprate high temperature superconductors [156]. Many unique properties let us classify this as an independent type apart from the 122-type. Ivanovskii reviewed this material [157].

In this project, we intercalated Na to FeSe employing the ammonothermal method, which cannot be prepared using a conventional thermal treatment at high temperatures. The result is described in section 3.1.6. Using this type of material with low Fe content as a Mott insulator, we examined the effect of the electric field on its electrical transport properties. The results are described in section 4.2.5.

##### 3.1.1.2. Electronic structure

**Figure 2.**
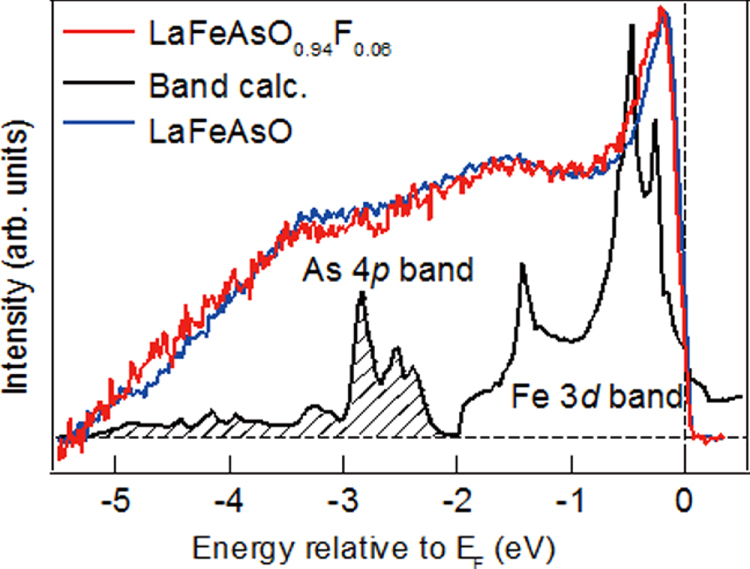
Photoemission spectra of LaFeAsO_1−*x*_F_*x*_ and calculated PDOS. The near-*E*_F_ peak and the weak peak at about −1.5 eV corresponding to Fe 3*d* bands survive, and a broad peak corresponding to the As 3*p* band appears in the range −(3 ∼ 4) eV. Reprinted with permission from [158]. Copyright 2008 by the Physical Society of Japan.

Figure 2 shows the photoemission spectra of LaFeAsO and LaFeAsO_0.94_F_0.06_, and calculated partial density of states (PDOS) for Fe 3*d* and As 4*p* [158]. The Fermi level (*E*_F_) controlling the transport property is primarily formed by a complex tangle of five Fe 3*d* orbitals, due to the small contribution of As, which is unlike cuprate superconductors where only Cu*d*_*x*2−*y*2_ contributes to the *E*_F_. With five bands comprising *E*_F_, multi-pockets, i.e., a disconnected Fermi surface (FS), appear on the FS. The energy levels of *d*_*x*2−*y*2,_ *d*_*xy*_, and *d*_*yz*_ are sensitive to both changes in the symmetry of the FeAs_4_ tetrahedron and the carrier density. Such an electronic structure dominates high *T*_c_ with the unique pairing mechanism.

**Figure 3.**
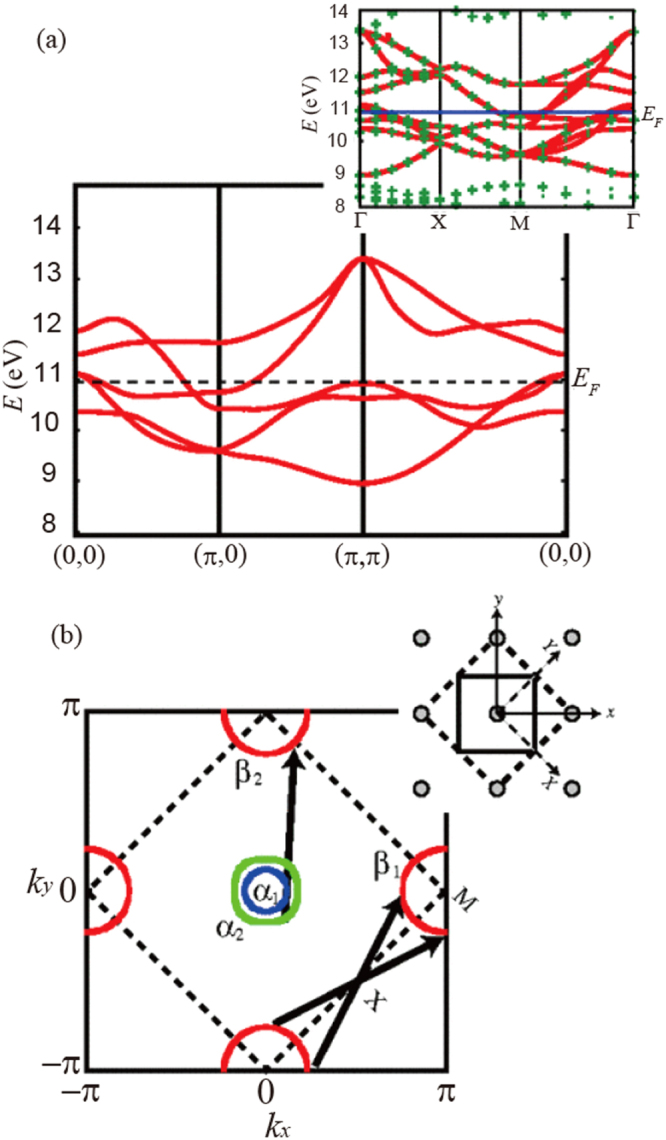
(a) The band structure of the five-band model in the unfolded BZ, where the interlayer hoppings are included. To compare with the ten-band model (thick red lines in the inset; the symbols are the present local-density approximation results), note the original (dashed lines) and the unfolded (solid lines) BZ shown in (b). (b) Fermi surface for *n* = 6.1 (with the interlayer hoppings ignored), with the arrows indicating the nesting vectors. The inset depicts the original (dashed lines) and reduced (solid lines) unit cell in real space. Reprinted with permission from [160]. Copyright 2008 by the American Physical Society.

At the early stage of the theoretical approach for the pairing mechanism, several physicists [159–166] suggested the possibility of spin fluctuation mediated pairing, where the spin fluctuation arises around the nesting vector (*π*, 0) (see figure 3 [160]). The spin fluctuation mediates s_±_-wave pairing, where the gap function has s-wave symmetry, but its sign is reversed between the electron and hole Fermi surfaces.

In contrast, recent experimental results showed that high *T*_c_ is revealed when the nesting is degraded, or even in the absence of the nesting by heavy doping of impurities [11, 149, 151, 154, 167]. To explain the robust superconducting state against impurities, Kontani and Onari [168] proposed a mechanism of the S_++_-wave superconducting state induced by orbital fluctuations, due to the phonon-mediated electron–electron interaction. On the other hand, Suzuki *et al* [169] succeeded in reproducing the general trend of composition dependence of *T*_c_ in *Ln*FeAsO_1*−x*_H_*x*_ (*Ln*: La, Ce, Sm and Gd) by the diagonal (next nearest neighbor) electron hopping model, where the next nearest neighbor (diagonal) hoppings between iron sites dominate over the nearest neighbor ones, plays an important role in the enhancement of the spin fluctuation and thus superconductivity. The theoretical and experimental evaluation for the superconducting mechanism will continue from now on.

##### 3.1.1.3. Electronic phase diagram

Unlike cuprate superconductors whose parent materials are Mott insulators, the parent materials of IBSCs are antiferromagnetic metals with sufficient conduction carriers. Hence, it is considered that carrier doping into IBSCs mainly alter the FS, which in turn leads to suppression of antiferromagnetism.

Here we mainly describe the electronic phase diagram for the 1111-type (*Ln*FeAsO, Ln: rare earth element). The parent materials for the 1111-type have tetragonal crystal structure at room temperature, but transform into orthorhombic structure at lower temperatures. In LaFeAsO, Pauli paramagnetism (PM) is shown around room temperature, and changes into AFM at a slightly lower temperatures (*T*_N_ ∼ 140 K) than that of the structural transitions (*T*_s_ ∼ 160 K) [170, 171].

Generally, superconductivity occurs in the tetragonal phase and not in the orthorhombic phase due to antiferromagnetic ordering in the orthorhombic phase. With doping (e.g., substituting O for F in *Ln*FeAsO), the tetragonal–orthorhombic transition temperature decreases and is accompanied by suppression of the AFM state and superconductivity emerges in succession. Electrons are doped into the bulk, when an element with more valence electrons is substituted. In contrast, holes are doped by substituting an element with fewer valence electrons. In many cases of both 1111 and 122-type, it is possible to substitute Fe or As in the conducting layer and *Ln*, *AE*, O or F in the blocking layer for other elements. The former and the latter are called ‘direct doping’ and ‘indirect doping’, respectively.

**Figure 4.**
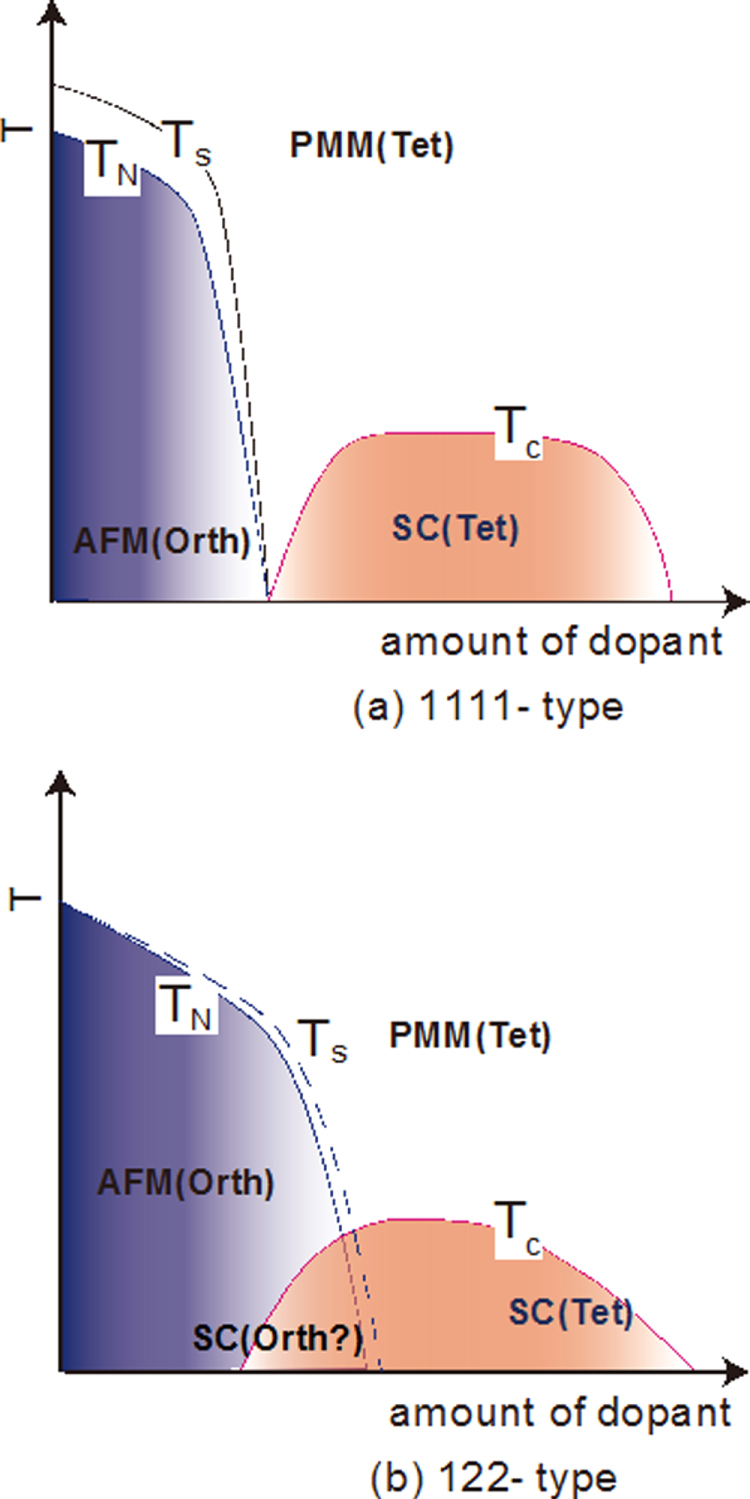
Schematic temperature versus composition phase diagram for (a) 1111-type and (b) 122-type (*T*_s_: structural transition temperature; *T*_N_: magnetic transition temperature; AFM: antiferromagnetic phase; PMM: paramagnetic metal phase; SC: superconducting phase). Reprinted with permission from [125]. Copyright 2012 by Maney Publishing.

The critical temperature (*T*_c_) increases, reaches a maximum, and then decreases as the dopant level increases. Since the decrease in *T*_c_ in the over doping level is due to the precipitation of the secondary phase as SmOF in SmFeAsO_1*−x*_F_*x*_, the proposed phase diagram for 1111-type doped with F does not show the correct *T*_c_ behavior in the over doping region [172–176]. In contrast, Hanna *et al* [9] prepared SmFeAsO_1*−x*_H_*x*_ and showed its optimal *T*_c_ (=55 K) at *x* = 0.20 and decrease in *T*_c_ by additional doping (over doping) without precipitation of the secondary phase, indicating a wide superconducting dome in 1111-type. Figure 4(a) shows the schematic phase diagram for the 1111-type.

The first IBSC reported was formed by electron doping as LaFeAsO_1*−x*_F_*x*_ where F substituted the O site as (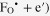 [4]. In addition to the substitution of oxygen sites by F (*T*_c_ = 55 K for SmFeAsO_0.9_F_0.1_) [115], various routes for electron doping have been reported, including the formation of an oxygen vacancy (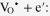 *T*_c_ = 55 K for SmFeAsO_0.85_) using a high-pressure synthesis [117, 177, 178] substitution of H^−^ for an O^2−^ site (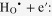 *T*_c_ = 55 K for SmFeAsO_0.8_H_0.2_) [9], substitution of Th for *Ln* (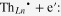 *T*_c_ = 56 K for Gd_0.8_Th_0.2_FeAsO) [118, 179], and substitution of Co, Ni or Ir for Fe (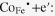 *T*_c_ = 14 K for LaFe_1*−x*_Co_*x*_AsO [180, 181], *T*_c_ = 22 K for I_1*−x*_Co_*x*_AsF [137], 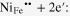 *T*_c_ = 6 K for LaFe_1*−x*_Ni_*x*_AsO [182], 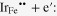 *T*_c_ = 18 K for SmFe_1*−x*_ Ir_*x*_AsO [183]). The optimal amount of doping is 0.1–0.2/Fe atoms for each route, and indirect doping appears to be more effective than direct doping in achieving a high *T*_c_, which should be due to less structural perturbation to the conducting layer.

For the 122-type, the shape of the electronic phase diagram is similar to the 1111-type as a general trend. The remarkable difference between the 1111- and 122-types is whether the AFM and superconducting phases are distinctly overlapped or not as shown in figure 4(b). In the 1111-type, the regions showing AFM and superconductivity are separated or barely overlap, whereas the 122-type materials have AFM regions with a high *T*_c_. The optimal *T*_c_ is apparently located around the temperature corresponding to the extrapolation of the SDW curve to zero temperature, i.e., a superconducting dome appears around the quantum critical temperature of SDW [184]. The emergence of SC by doping of isoelectronic dopant, such as P for As, is also a unique property of the 122-type.

The comparison in doping between the 122- and the 1111-type is shown in table 4.

**Table 4.** Typical example of emergence of superconductivity by doping (*T*_c_ and composition).

	1111		122	
Doping type	indirect	direct	indirect	direct
	55 K (SmFeAsO_0.9_F_0.1_) [115]	14 K (LaFe_0.89_Co_0.11_AsO) [180]	45 K (Ca_0.83_La_0.17_Fe_2_(As,P)_2_	22 K (BaFe_1.8_Co_0.2_As_2_) [186]
Electron	55 K (SmFeAsO_0.85_) [117]	22 K(CaFe_0.9_Co_0.1_AsF) [137]	[23]	19 K (BaFe_1.908_Ni_0.092_As_2_) [187]
	55 K (SmFeAsO_0.8_H_0.2_) [9]	6 K (LaFe_0.96_Ni_0.04_AsO) [182]	22 K (Sr_0.6_La_0.4_Fe_2_As_2_) [19]	24 K (BaFe_1.886_Rh_0.114_As_2_) [188]
	56 K (Gd_0.8_Th_0.2_FeAsO) [118]	18 K (SmFe_0.89_Ir_0.11_AsO) [183]	22 K (Ba_0.93_La_0.07_Fe_2_As_2_) [20]	18 K (BaFe_1.914_Pd_0.086_As_2_) [188]
Hole	13 K(?) (Nd_0.8_Sr_0.2_FeAsO) [185]	no	38 K (Ba_0.6_K_0.4_Fe_2_As_2_) [119]	no
Isoelectronic	no	no	no	30 K (BaFe_2_(As_0.68_P_0.32_)_2_) [189]
22 K (Ba(Fe_0.56_Ru_0.44_)_2_As_2_) [190]				

##### 3.1.1.4. Properties

Compared to MgB_2_ and cuprates, IBSCs have several distinct characteristics. It is included in the unique characteristic of IBSCs that the Fe 3*d* multi-orbital form Fermi surface described in (b) and the parent material is the antiferromagnetic metal described in (c).

Generally, *T*_c_ decreases upon doping with magnetic impurities such as Fe, Ni, and Co. In the case of cuprate superconductors, *T*_c_ of YBa_2_Cu_3_O_7−*y*_ decreases from 90 K to 50 K by substituting Ni (17%) for Cu, and that of La_1.85_Sr_0.15_CuO_4−*y*_ also decreases from 40 K to 4.2 K by substituting Ni (5%) for Cu [191]. The substitution of such elements for Fe on FeSCs with an optimal state shows a similar trend. The superconductivity of NdFeAsO_0.89_F_0.11_ (*T*_c_ = 48 K) disappears by substituting Co (>11%) or Mn (>4%) [192]. In contrast, the emergence of superconductivity by substitution of Co^2+^ (3*d*^7^), Ni^2+^ (3*d*^8^) or other transition metals for Fe^2+^ (3*d*^6^) in the non-superconducting parent material described in (c) is also a unique nature for IBSC.

The high *T*_c_, large upper critical field (*H*_c2_) and small anisotropy are important merits in applying IBSCs practically. Table 5 summarizes these values of IBSCs along with those of MgB_2_ and cuprates. Only cuprates achieve higher *T*_c_ than the boiling point of liquid N_2_ (77 K). It has been reported that the anisotropic ratio of the resistivity (*γ*_*ρ*_) of the 122-type IBSCs is compatible with that of MgB_2_ and smaller than that of cuprates. The *H*_c2_(0) of IBSCs is higher than that of MgB_2_, but is smaller than that of a typical cuprate. The anisotropic ratio of the *H*_c2_, *γ*_H_, of IBSCs is smaller than those of MgB_2_ and cuprates. The *H*_c2_(0) is defined as the upper critical field at 0 K. The *γ*_*ρ*_ means the ratio of the resistivity along the crystal axes directions, *a* (*ρ*(*a*)) and *c* (*ρ*(*c*)) measured just above *T*_c_. The application of IBSCs to superconducting wires and devices will be described in section 4.

**Table 5.** Properties of high *T*_c_ superconductors.

	IBSCs	MgB_2_	Cuprates
Parent material	antiferromagnetic metal (*T*_N_ ∼ 150 K) (excepting for the 245-type)	non-magnetic metal	antiferromagnetic insulator (*T*_N_ ∼ 400 K)
Orbitals composing Fermi level	five Fe 3*d* orbitals	two B2*p* orbitals	single Cu3*d* orbital
*T*_c_	56 K (Gd_0.8_Th_0.2_SmFeAsO) [118]	39 K (pure MgB_2_) [196]	92 K (YBa_2_Cu_3_O_7−*δ*_,) [199]
	38 K (Ba_0.6_K_0.4_Fe_2_As_2_) [119]		105 K (Bi_2_Sr_2_Ca_2_Cu_3_O_10_) [200]
			134 K (HgBa_2_Ca_2_Cu_3_O_8_) [201]
*H*_c2_(0)^//^^*b*^^a^	∼90 T ((Ba_0.55_K))Fe_2_As_2_) [193]	∼40 T (MgB_1.87_C_0.13_) [197]	210 T (YBa_2_Cu_3_O_7−*δ*_,) [202]
	∼150 T (SmFeAsO_0.7_F_0.25_) [193]	∼18 T (pure MgB_2_) [197]	400 T (Bi_2_Sr_2_CaCu_2_O_8_) [202]
*γ*_*ρ*_ = *ρ*(*c*)/*ρ*(*ab*)	3–5 (Ba(Fe_0.936_Co_0.074_)_2_As_2_) [194]	∼3.5 (pure MgB_2_) [198]	30-250 (YBa_2_Cu_3_O_7−*δ*_,) [203–205]
	8-10 (SmFeAsO_0.7_F_0.25_) [94]		>1000 (Bi_2_Sr_2_CaCu_2_O_8_) [206]
*γ*_*H*_=*H*_c2_(0)^//^^*b*^/*H*_c2_(0)^//*c*^	1.1-1.2 ((Ba_0.55_K))Fe_2_As_2_) [193]	∼4 (MgB_1.87_C_0.13_) [197]	6 (YBa_2_Cu_3_O_7−*δ*_,) [202]
	∼1.5 (SmFeAsO_0.7_F_0.25_) [195]	∼6 (pure MgB_2_) [197]	21 (Bi_2_Sr_2_CaCu_2_O_8_) [202]

a H_c2_(0)^//ab^: estimated by extrapolating the *H*_c2_-T curve, where *H*_c2_ is measured by applying a magnetic field along the *ab* plane.

#### 3.1.2. Hydrogen as an electron donor

The most effective route to achieve high *T*_c_ is ‘indirect’ and ‘electron’ doping into the 1111-type (see table 4). As mentioned in the previous section, the solubility limit of F, which is used for the ‘indirect’ and ‘electron’ dopant, is 20% at most. Thus, one could not know the effect on the emergence of SC by the impurity doping for its whole SC dome. So we have explored an appropriate dopant with high solubility limit.

**Figure 5.**
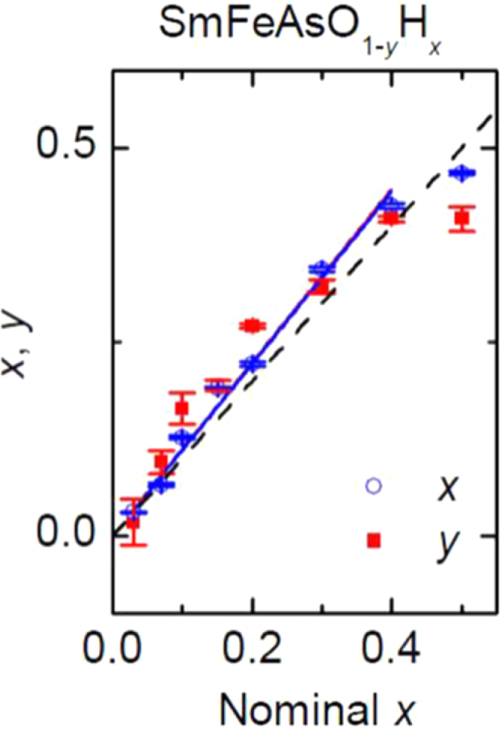
Oxygen deficiency content determined by EPMA (*y*) and hydrogen content estimated by TG-MS (*x*) in SmFeAsO_1–*y*_H_*y*_ as a function of nominal *x* in the starting mixture. The measured *x* is almost equal to *y* and nominal *x*, indicating that the deficiency of the oxygen site is wholly compensated for by the occupation of hydrogen. Reprinted with permission from [9]. Copyright 2011 by the American Physical Society.

**Figure 6.**
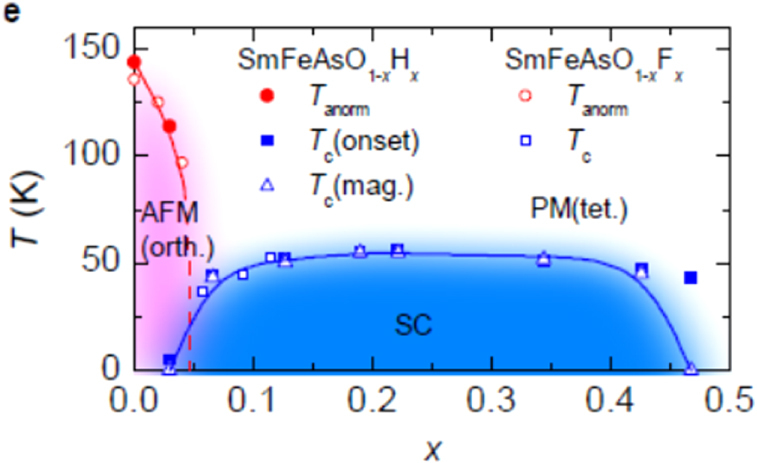
*x*–*T* diagram of SmFeAsO_1–*x*_H_*x*_ superimposed by that of SmFeAsO_1–*x*_F_*x*_. Reprinted with permission from [9]. Copyright 2011 by the American Physical Society.

Out of some candidates of monovalent anions (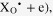 we choose the hydride ion (H^−^) as an electron dopant which could substitute for O^2−^ [9, 12]. Though it is generally considered that the ionic state of hydrogen is a proton (H^+^) in condensed matter, hydrogen is also present as the hydride ion (H^−^) in stable materials such as LaH_2_, CaH_2_ and NaH. Its ionic radius depends on the environment, i.e., 208 pm in Pauling’s estimation, 129 pm in NaH and 106 pm in CaH_2_. We considered that H^−^ should be able to dissolve into 1111-type superconductors with replacement of O^2−^ because the blocking layer is composed of a lanthanide cation which can form stable hydrides. To prevent the evaporation of hydrogen during the preparation process, we employed a high pressure synthesis technique, i.e., under 2 GPa at 1200 °C. The experimental results clearly showed a high solubility limit of H^−^ doping. While the impurity phases as SmAs and/or SmOF precipitate in SmFeAsO_1*−x*_F_*x*_ (*x* > 0.15) [207, 208], such phases could not be observed in SmFeAsO_1*−x*_H_*x*_ (*x* ≤ 0.4). Figure 5 compares the hydrogen content (*x*) and the deficient amount of oxygen (*y*) in the prepared samples per the chemical formula (SmFeAsO_1−*y*_H_*x*_) as a function of nominal *x* in the starting mixture [9]. The former value was determined by TG-MS, and the latter was measured using EPMA. For nominal *x* ≤ 0.4, the hydrogen content agrees with *y* and the nominal *x*, indicating the oxygen site (O^2−^) was successfully substituted with hydrogen (H^−^). Figure 6 shows the electronic phase diagram of SmFeAsO_1*−x*_H_*x*_ superimposed with that of SmFeAsO_1*−x*_F_*x*_, with the fluorine content *x* measured by EPMA as reported by Köhler and Behr [207], in which the *T*_c_ versus *x* plots of SmFeAsO_1*−x*_H_*x*_ and SmFeAsO_1*−x*_F_*x*_ overlap at *x* < 0.15, indicating that hydrogen gives indirect electron doping to the FeAs layer just like fluorine. While the solubility limit of fluorine in the oxygen site is restricted to less than 20% (*x* = 0.2) [172, 207, 208], that of hydrogen can reach 40% for not only Sm-1111 but also all *Ln*-1111 compounds. The wider substitution range is useful for the optimization of the electron-doping level to induce superconductivity and to complete the electronic phase diagram, including the overdoped region.

**Figure 7.**
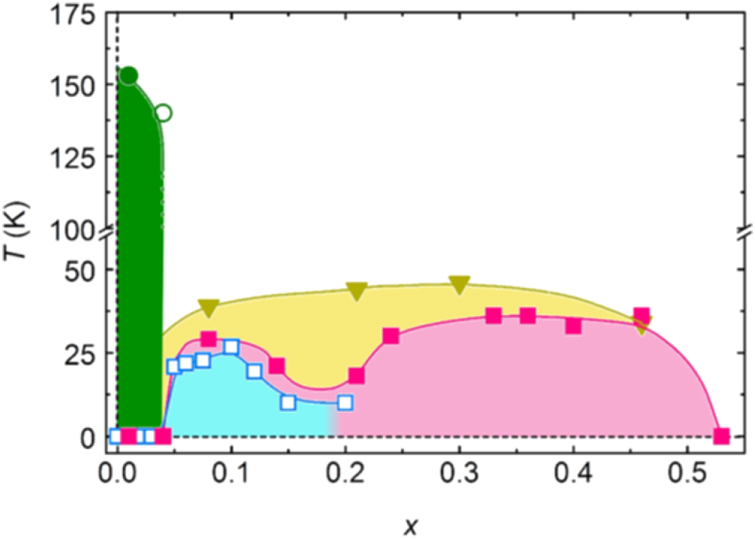
Electronic phase diagram for LaFeAsO_1−*x*_H_*x*_ (filled symbols) and LaFeAsO_1−*x*_F_*x*_ (open symbols). The *T*_*c*_ under ambient pressure (squares) and 3 GPa (inverted triangles) was determined from the intersection of the two extrapolated lines around the superconducting transition and *T*_s_ (circles) was taken as the anomaly kink in the resistivity-temperature (*ρ*–*T*) curve. Reprinted with permission from Macmillan Publishers Ltd: [11], Copyright 2012.

The unique feature of revealed SC is observed in LaFeAsO_1*−x*_H_*x*_ [11]. While *T*_c_–*x* plots for *Ln*FeAsO_1*−x*_H_*x*_ (*Ln* = Ce, Sm, Gd) exhibit a single *T*_c_ dome as shown in figure 6 (for Sm), LaFeAsO_1*−x*_H_*x*_ has a two *T*_c_ dome structure (see figure 7). When the amount of dopant (*x*) is increased, the SC emerged from *x* > 0.04, *T*_c_ increased and reached a maximum at *x* = 0.1 (*T*_c_ = 26 K) and then decreased for a while. Surprisingly after reaching the minimum at *x* = 0.2, *T*_c_ increased again and showed a broad peak (maximum *T*_c_ = 36 K at *x* = 0.35), which could not be attained by F-doping. The two domes merged into a wider single dome with the optimal *T*_c_ (=45 K at *x* = 0.3) by applying a high pressure of 3 GPa. The characteristics of these two *T*_c_ domes in LaFeAsO_1*−x*_H_*x*_ are listed in table 6.

**Table 6.** Characteristics of two domes in LaFeAsO_1−*x*_H_*x*_.

Dome	First	Second
*x*	0.05 ≤ *x* ≤ 0.2	0.2 ≤ *x* ≤ 0.5
Exponent, *n*	0.0 ≤ *n* ≤ 2.3	0.7 ≤ *n* ≤ 2.0
*T*_c_^max^	29 K	36 K
*T*_c_ sensitivity to *x*	High	Low
Under high pressure	Unified	Unified
FS nesting between hole and electron pockets	Strong	Weak
DOS(*E*_F_)	No shoulder	Shoulder

The exponent (*n*) was estimated from the curve fitting of *ρ*-*T* plots to *ρ* = A*T*^*n*^+*ρ*_0_ near *T*_c_.

**Figure 8.**
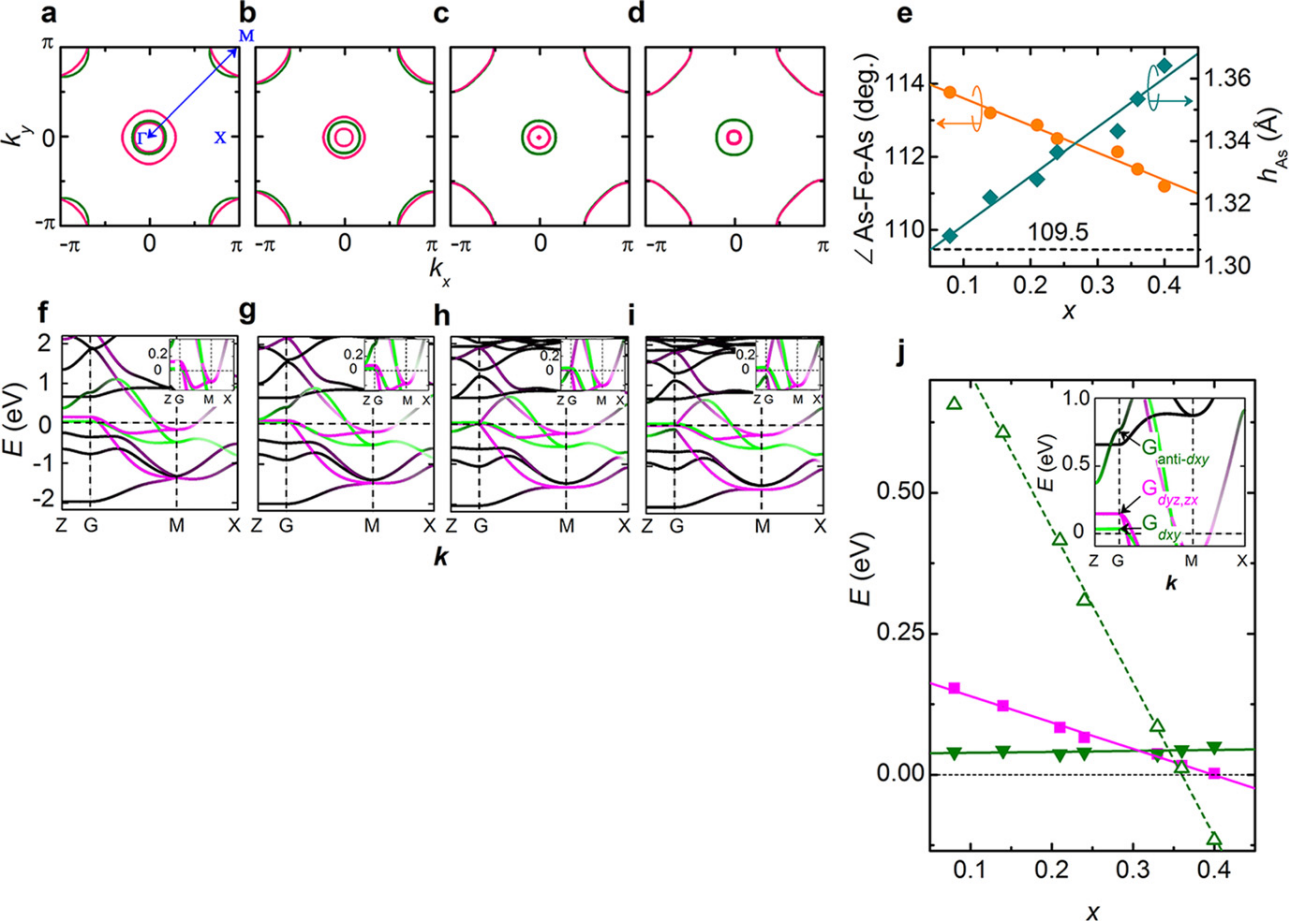
Electronic structure of LaFeAsO_1−*x*_H_*x*_. (a)–(d) Two-dimensional Fermi surface of LaFeAsO_1−*x*_H_*x*_ with *x* = 0.08 (a), 0.21 (b), 0.36 (c) and 0.40 (d). The blue arrow represents the nesting vector in the (*π*–*π*) direction. The contribution of Fe-*d*_*xy*_ and *d*_*yz*,*zx*_ orbitals are colored green and pink, respectively. (f)–(i) Band structures of LaFeAsO_1−*x*_H_*x*_ with *x* = 0.08 (f), 0.21 (g), 0.36 (h) and 0.40 (i). Insets show close-up views of the low energy region. The contribution of Fe-*d*_*xy*_ and *d*_*yz,zx*_ orbitals are colored green and pink, respectively. (j) Variation in energy level of relevant Fe 3*d* bands at G point with *x*. The inset is the band structure of LaFeAsO_0.92_H_0.08_. The 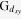 (filled green inverted triangles) and G_anti−*dxy*_ (open green triangles) signify the bonding and anti-bonding states, respectively, for a bond primary composed of two Fe-*d*_*xy*_ orbitals in a unit cell. Also shown is the energy level of degenerate *d*_*yx*,*zx*_ band (G_*dyz*,*zx*_ indicated by filled pink squares). The solid and dashed lines are as a visual guide. Reprinted with permission from Macmillan Publishers Ltd: [11], Copyright 2012.

Iimura *et al*, who found this two dome structure, considered initially that the superconductivity in the low *x* region (first dome) was due to the spin fluctuation mechanism and that in the high *x* region (second dome) was due to the orbital fluctuation mechanism. Their density functional theory (DFT) calculations showed the strong FS nesting between the hole (at *Γ* point) and the electron (at M point) pockets at the low *x* region, which was the most important glue in the spin fluctuation model (see figures 8(a)–(h)). On the other hand, the FS nesting weakened with increasing *x*, while the difference between the energy levels of Fe 3*d* bands (3*d*_*xy*_, 3*d*_*yz*_, and 3*d*_*xx*_) decreased and became almost zero (degeneracy) at *x* = 0.30–0.35 where the maximum *T*_c_ of the second dome occurs (see figure 8(i)).

**Figure 9.**
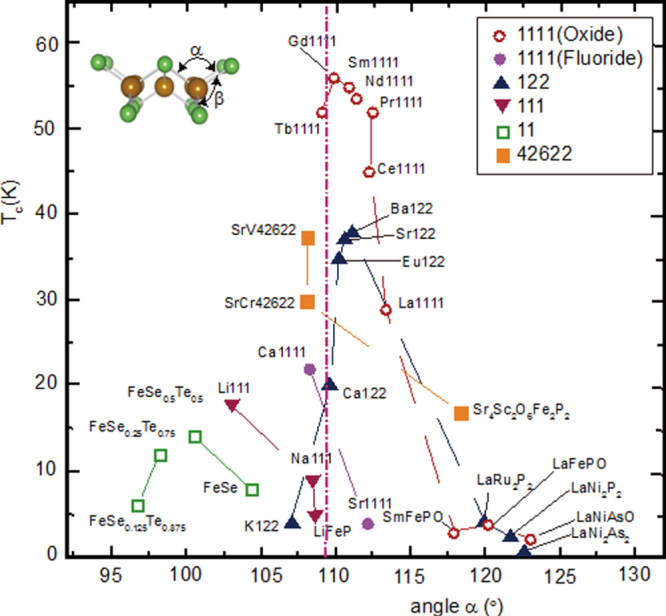
Correlation between *T*_*c*_ and bonding angle *α* of *Pn*(*Ch*)–Fe–*Pn*(*Ch*). *α* is adopted mainly from the data of the parent materials measured at room temperature. *T*_*c*_ shows the highest reported value. Reprinted with permission from [125]. Copyright 2012 by Maney Publishing.

The importance of degeneracy in IBSCs is deduced from the empirical plots reported by Lee *et al*, where the more the bonding angle of As–Fe–As (*α*: see figure 1(b)) is near 109.5° (regular FeAs_4_ tetrahedron), the higher the *T*_c_ revealed [209]. The regular FeAs_4_ tetrahedron is achieved by the degeneracy of Fe 3*d* bands and hence it should be reasonable to consider that the orbital fluctuation mechanism to mediate superconductivity emerges effectively in such a condition. The effect of the degeneracy of Fe 3*d* bands for the emergence of superconductivity is clear from the comparison of a lanthanide cation substituted with La. The *α* of the parent phases of *Ln*FeAsO_1*−x*_H_*x*_ are 114° (La), 112° (Ce), 111° (Sm) and 110° (Gd) and these values increase with doping of F or H. As shown in figure 9, the material of which *α* is close to 109.5° achieves the degeneracy of Fe 3*d* bands and regular tetrahedral structure by a small amount of doping and shows *T*_c_ maximum simultaneously. From this viewpoint, Iimura *et al* proposed that the high *T*_c_ of 1111-type superconductors was mainly due to the orbital fluctuation mechanism.

**Figure 10.**
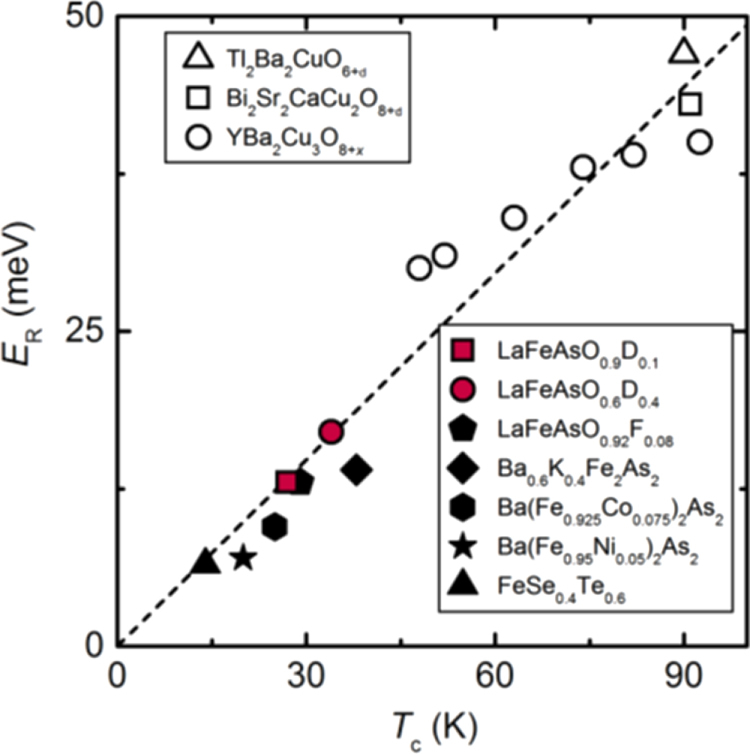
The *T*_*c*_ dependence of *E*_R_ in iron pnictides (filled symbols) and cuprates (open symbols). The red square and circles are the present data for the samples with *x* = 0.1 and 0.4, respectively. The dashed line is the averaged slope of 5.7*k*_B_*T*_c_. Reprinted with permission from [210]. Copyright 2013 by the American Physical Society.

On the other hand, the experimental result using inelastic neutron scattering for LaFeAsO_1*−x*_D_*x*_ suggested the presence of spin fluctuation in both low and high *x* superconducting regions with different wave number [210]. Such spin fluctuation disappeared at the boundary of *x* = 0.2. The relationships between *T*_c_ and *E*_R_ of several cuprate materials and IBSCs containing these two regions are plotted in figure 10, where *E*_R_ is the measured value indicating the strength of spin fluctuation. For superconductors deriving from the spin fluctuation mechanism, it is known that the value of *E*_R_/*k*_B_*T*_c_ is 4–6. As shown in figure 10, all plots line up roughly on the straight line with the gradient of 5.7, which indicates that the spin fluctuation mechanism acts primarily to reveal superconductivity in not only the low *x* but also the high *x* region. The theoretical calculations based on the random-phase approximation indicate that the spin fluctuations at *x* = 0.1 are due to intra-orbital nesting within Fe 3*d*_*yz,zx*_, whereas the spin fluctuations at *x* = 0.4 originate from intra-orbital nesting within Fe 3*d*_*x*2−*y*2_. These results suggest that the orbital multiplicity plays an important role in the doping and/or material dependence of *T*_c_ of the IBSCs.

**Figure 11.**
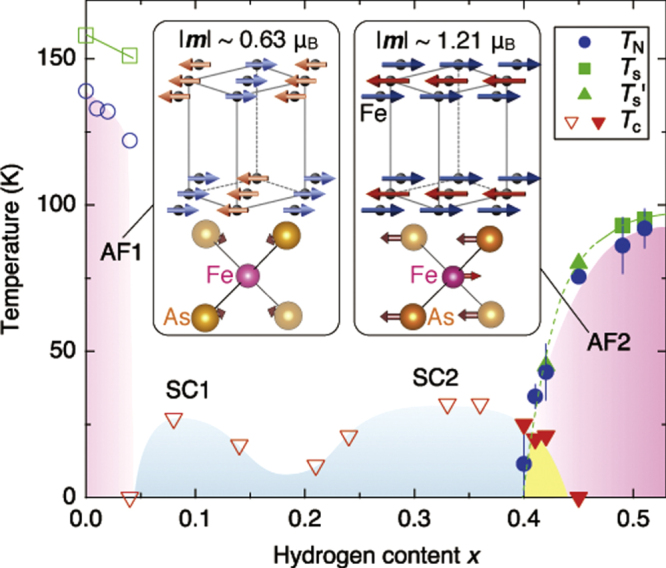
Magnetic, structural and superconducting phase diagram of *La*FeAsO_1−*x*_H_*x*_. The original parent compound with *x* = 0 exhibits a structural transition at *T*_s_ = 155 K, followed by an antiferromagnetic state (AF1) at *T*_N_ = 137 K. With increasing *x*, two superconductivity domes appear: 0.05 ≤ *x* ≤ 0.20 (SC1) with *T*_c,max_ = 26 K, and 0.20 ≤ *x* ≤ 0.42 (SC2) with *T*_c,max_ = 36 K. Eventually, another antiferromagnetic phase (AF2) appears in the range 0.40 ≤ *x* ≤ 0.51. In the advanced parent compound at *x* = 0.51, structural and magnetic transitions occur at *T*_s_ ∼ 95 K and *T*_N_ = 89 K, respectively. *T*_s_’ indicates the *c* axis upturn temperature observed in x-ray measurements. The filled and open marks are obtained from the present and previous results, respectively. The magnetic structures of AF1 (left) and AF2 (right) are shown with their magnetic moments *m*, where the solid lines represent the tetragonal cell. The displacements of the Fe and As atoms across the structural transitions are schematically described by the arrows on the FeAs_4_ tetrahedra from the view of the orthorhombic long axis, in which the Fe and As atoms move by 0.07 Å (0 Å) and 0.06 Å (0.01 Å) in *x* = 0.51 (*x* = 0), respectively. The error bars represent the uncertainty in the least-squares fitting routines. Reprinted with permission from Macmillan Publishers Ltd: [211], Copyright 2014.

The discovery of a two-SC-dome structure implies the existence of responsible two parent materials. Hiraishi *et al* found a parent phase in the high *x* region with different magnetic structure from that on *x* = 0 using the multi-probe method composed of neutron, muon and synchrotron x-ray beams complementarily [211]. It was clarified by the muon spin relaxation (*μ*SR) that new magnetic ordering phase developed with increasing dopant over *x* ∼ 0.4. From the experimental results using neutron and synchrotron x-ray diffraction, it was identified that this magnetic phase has a different antiferromagnetic ordering from that on *x* = 0 and the structural transition occurs at *x* ∼ 0.5 where the magnetic transition temperature shows maximum. Such features are shown in figure 11. This new magnetic phase is tentatively assigned to the parent phase in the high *x* region.

**Figure 12.**
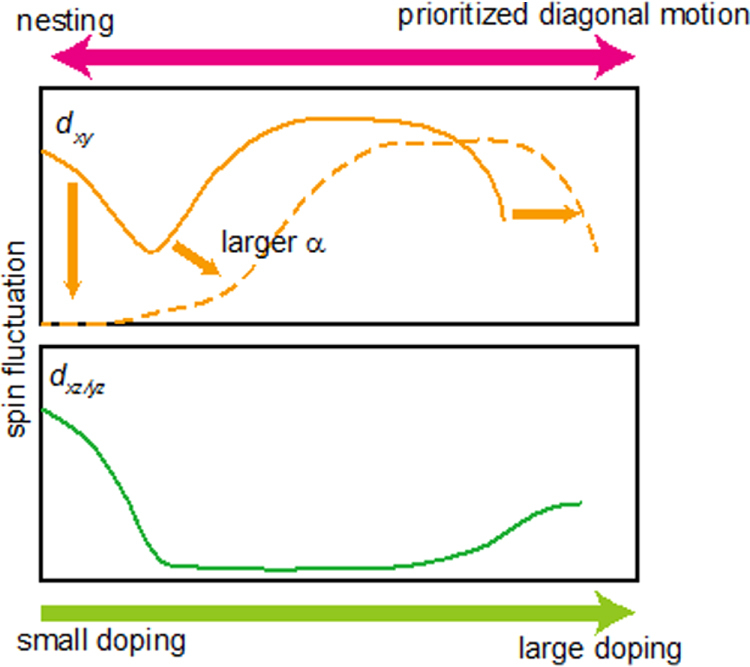
Schematic figure of the spin fluctuation contribution to superconductivity. Reprinted with permission from [169]. Copyright 2014 by the American Physical Society.

In theoretical approach, Suzuki *et al* successfully explained the composition dependence for *Ln*FeAsO_1*−x*_H_*x*_ (*Ln*: La, Ce, Sm, Gd) [169]. They showed that, besides the Fermi surface nesting, a peculiar motion of electrons, where the next nearest neighbor (diagonal) hopping between iron sites dominates over nearest neighbor ones, plays an important role in the enhancement of the spin fluctuation and thus superconductivity. According to their explanation, the crossover between the Fermi surface and this ‘prioritized diagonal motion’ regime occurs smoothly with doping in the Ce, Sm and Gd case, while the two regimes are separated to give a double dome *T*_c_ phase diagram in the La case. The feature of this change is schematically drawn in figure 12.

**Figure 13.**
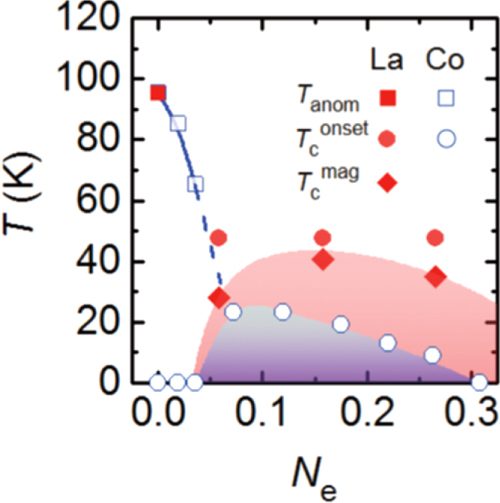
(a) Electronic phase diagram of Ca_1−*x*_La_*x*_FeAsH_1−*y*_O_*y*_ as a function of the total number of doped electrons per iron (*N_e_* = *x*–*y*), superimposed on that of CaFe_1−*x*_Co_*x*_AsH. Reprinted with permission from [17]. Copyright 2014 by the Physical Society of Japan.

The hydrogen doping technique was applied to the alkaline earth metal 1111-type materials, such as CaFeAsF. It reveals superconductivity by replacing Fe with Co (*T*_c_ = 26 K) [137]. Hanna *et al* realized the electron doping by partially replacing F with H and heating them in He to selectively eliminate H as CaFeAsF_1*−x*_H_*x*_ → CaFeAsF_1*−x*_ + *x*/2H_2_ [18]. The *T*_c_ of 29 K was attained by forming an F vacancy (20% of the F site). In the case of direct electron doping into CaFeAsH, the substitution of Fe with Co led this material to become a superconductor (max *T*_c_ = 29 K) [16]. Indirect electron doping (substitution of Ca with La) showed higher *T*_c_ than that of Co doping as shown in figure 13 [17]. The material obtained by substitution of 20% of Ca with La showed the highest *T*_c_ (47 K) in IBSCs without a rare-earth element as the main component.

#### 3.1.3. Rare-earth doped 122 iron arsenides

Rare-earth (*RE*) doping of 122-type iron arsenides has been intensively studied as part of the FIRST Project. The major achievements in this regard include the high-pressure synthesis of (Sr_1–*x*_La_*x*_)Fe_2_As_2_ with a maximum *T*_c_ of 22 K [19] and the growth of thin films of (Sr_1–*x*_La_*x*_)Fe_2_As_2_ (maximum *T*_c_ = 20.8 K) [22] and (Ba_1–*x*_La_*x*_)Fe_2_As_2_ (maximum *T*_c_ = 22.4 K) by pulsed laser deposition (PLD) [20, 21]. Further, (Ba_1–*x*_*RE*_*x*_)Fe_2_As_2_ with *RE* = Ce, Pr and Nd has also been obtained by means of a nonequilibrium film growth process [21]. The success of *RE* doping enabled us to compare the phase diagrams of electron (La) and hole (K) doped BaFe_2_As_2_ as well as those of indirectly (La or K) and directly (Co) doped BaFe_2_As_2_. Another achievement is the simultaneous La and P doping of CaFe_2_As_2_ [23]: melt-grown crystals of (Ca_1–*x*_La_*x*_)Fe_2_(As_1–*y*_ P_*y*_)_2_ exhibited bulk superconductivity with a maximum *T*_c_ of 45 K for 0.12 ≤ *x* ≤ 0.18 with *y* = 0.06, while (Ca_1–*x*_La_*x*_)Fe_2_As_2_ without phosphorus exhibited filamentary superconductivity [23]. Characteristic two-dimensional Fermi surfaces were observed by means of angle-resolved photoemission spectroscopy (ARPES) in the 45 K phase of La- and P-doped CaFe_2_As_2_ [212].

To induce superconductivity in 122-type *AE*Fe_2_As_2_ (where *AE* = alkaline-earth elements), both types of doping carrier, i.e., holes and electrons, are typically used by substituting appropriate aliovalent elements, which include alkali metals (*A*) such as K, which substitute for *AE* sites as in hole-doped (Ba_1–*x*_K_*x*_)Fe_2_As_2_ [119], and transition metals (*TM*) such as Co, which substitute for Fe sites as in electron-doped Ba(Fe_1–*x*_Co_*x*_)_2_As_2_ [186, 193]. The doping can be classified into two types for the 122-type *AE*Fe_2_As_2_, namely, ‘indirect doping’ for doping at sites other than the Fe sites and ‘direct doping’ for doping at the Fe sites [21]. Because the superconducting FeAs and intermediary *AE* layers are spatially separated, direct doping has a major influence on carrier transport and thus superconductivity. Tables 7 and 8 summarize the maximum *T*_c_ of directly doped *AE*(Fe_1–*x*_ *TM*_*x*_)_2_As_2_ and indirectly doped (*AE*_1–*x*_*A*_*x*_)Fe_2_As_2_, respectively. The indirectly hole-doped (Ba_1–*x*_K_*x*_)Fe_2_As_2_ exhibits a maximum *T*_c_ of 38 K, which is considerably higher than those for directly electron-doped *AE*(Fe_1–*x*_*TM*_*x*_)_2_As_2_. Therefore, we expected that a new indirect ‘electron’ doping at the *AE* sites for *AE*Fe_2_As_2_ would lead to high-*T*_c_ superconductivity, as expected from the markedly higher *T*_c_ observed for indirectly electron-doped SmFeAs(O_1–*x*_F_*x*_) (55 K) [115] than that for directly electron-doped Sm(Fe_1–*x*_Co_*x*_)AsO (17 K) [232].

**Table 7.** The maximum superconducting transition temperature *T*_c_ (K) of transition metal (TM)-doped AE(Fe_1–*x*_TM_*x*_)_2_As_2_, where AE=Ca, Sr and Ba; and TM=Co, Ni, Ru, Rh, Pd, Ir and Pt. For instance, *T*_*c*_ = 20 K for Ca(Fe_1–*x*_Co_*x*_)_2_As_2_. Pt-doped Ca(Fe_1–*x*_ Pt_*x*_)_2_As_2_ does not exhibit superconductivity [75].

	Fe	Co	Ni
Ca		20 [213]	15 [214]
Sr		19.2 [215]	9.8 [216]
Ba		23 [186, 193]	20.5 [217]
	Ru	Rh	Pd
Ca		14 [218]	10 [219]
Sr	19.3 [220]	21.9 [221]	8.7 [221]
Ba	22 [190]	23.2 [188]	19 [188]
		Ir	Pt
Ca		22 [222]	no SC [75]
Sr		24.2 [221]	16.5 [223]
Ba		28 [224]	23 [225]

**Table 8.** The maximum superconducting transition temperature *T*_c_ (K) of the alkali metal (A)-doped (*AE*_1–*x*_ *A*_*x*_)Fe_2_As_2_, where *AE*=Ca, Sr and Ba; and *A*=Na, K, Rb and Cs.

	Na	K	Rb	Cs
Ca	26 [226]			
Sr	26 [227]	36.5 [228, 229]		37.2 [228]
Ba	34 [230]	38 [119]	23 [231]	

However, indirect electron doping of Sr(Ba)Fe_2_As_2_ by substituting the divalent Sr(Ba) sites with trivalent *RE* ions was difficult to perform by means of conventional solid-state reactions. While Muraba *et al* [19] and Wu *et al* [233] examined the La substitution for SrFe_2_As_2_ and BaFe_2_As_2_, respectively, solid-state reactions of the ingredient mixture for (Sr_1–*x*_La_*x*_)Fe_2_As_2_ or (Ba_1–*x*_La_*x*_)Fe_2_As_2_ did not yield the La-substituted 122 phase upon using the conventional glass-tube technique.

In contrast, the indirect RE doping of CaFe_2_As_2_ was possible by a conventional melt-growth technique for *RE* = La, Ce, Pr and Nd [234–237]. These materials exhibited superconductivity at *T*_c_ = 40–49 K: Saha *et al* [234] reported *T*_c_ = 47 K in (Ca_1–*x*_Pr_*x*_)Fe_2_As_2_; Gao *et al* [235] reported *T*_c_ = 42.7 K in (Ca_1–*x*_La_*x*_)Fe_2_As_2_; and Lv *et al* [236] reported *T*_c_ = 49 K in (Ca_1–*x*_Pr_*x*_)Fe_2_As_2_. These values of *T*_c_ are considerably higher than those reported for directly electron-doped Ca(Fe_1–*x*_*TM*_*x*_)_2_As_2_ and indirectly hole-doped (Ca_1–*x*_*A*_*x*_)Fe_2_As_2_ listed in tables 7 and 8, respectively. However, the shielding volume fractions of *RE*-doped CaFe_2_As_2_ were as low as <1% at 40 K [234, 235] or the shielding signal around 40 K was completely suppressed by the application of a weak magnetic field of 20 Oe [236].

**Figure 14.**
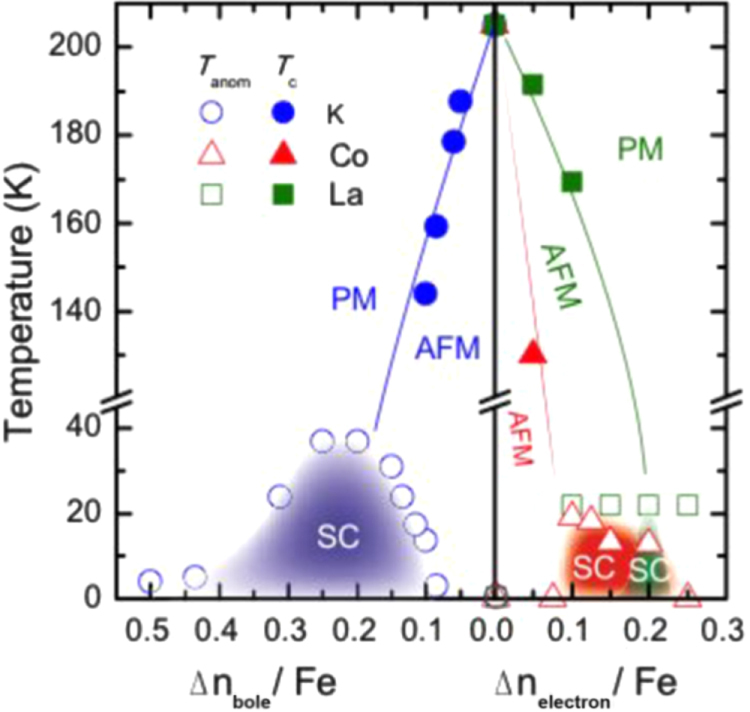
Electronic phase diagrams for electron-doped (Sr_1–*x*_La_*x*_)Fe_2_As_2_ [19] and Sr(Fe_1–*x*_Co_*x*_)_2_As_2_ [215] and for hole-doped (Sr_1–*x*_K_*x*_)Fe_2_As_2_ [229]. The ratio *Δn*_electron_/Fe and *Δn*_hole_/Fe denote the injected number of electrons and holes per Fe atom, respectively. Reprinted with permission from [19]. Copyright 2010 by the American Physical Society.

Under such circumstances, Muraba *et al* have succeeded in the indirect La doping of SrFe_2_As_2_ by applying a high-pressure synthesis process to obtain (Sr_1–*x*_La_*x*_)Fe_2_As_2_ polycrystals [19]. The ionic radius of La^3+^ (116 pm) is smaller than that of Sr^2+^ (126 pm) and therefore, it is natural that high-pressure synthesis is effective for obtaining La-substituted SrFe_2_As_2_. Muraba *et al* used a belt-type anvil cell to generate a pressure of 2 or 3 GPa at 1000 °C for 2 h and obtained solid solutions of (Sr_1–*x*_La_*x*_)Fe_2_As_2_ for 0 ≤ *x* ≤ 0.5. Further, (Sr_1–*x*_ La_*x*_)Fe_2_As_2_ exhibited bulk superconductivity over a narrow range around *x* = 0.4. The almost *x*-independent *T*_c_ (shown in figure 14) suggested the inhomogeneous replacement of La dopants at the Sr sites. Figure 14 compares the electronic phase diagram of (Sr_1–*x*_La_*x*_)Fe_2_As_2_ [19] with those of Sr(Fe_1–*x*_ Co_*x*_)_2_As_2_ [215] and (Sr_1–*x*_K_*x*_)Fe_2_As_2_ [229]. Here, the doped carrier number per Fe, i.e., *Δn*/Fe = *x*/2 for (Sr_1–*x*_La_*x*_)Fe_2_As_2_ and (Sr_1–*x*_La_*x*_)Fe_2_As_2_ and *Δn*/Fe = *x* for Sr(Fe_1–*x*_Co_*x*_)_2_As_2_, is plotted in place of the doping composition *x*. The directly electron-doped Sr(Fe_1–*x*_Co_*x*_)_2_As_2_ exhibits superconductivity for 0.1 ≤ *Δn*/Fe ≤ 0.2 with a maximum *T*_c_ of 19 K at *Δn*/Fe ∼ 0.1, at which point the antiferromagnetic ordering vanishes [215]. The maximum *T*_c_ of the indirectly electron-doped (Sr_1–*x*_La_*x*_)Fe_2_As_2_ is slightly higher than but close to that of directly electron-doped Sr(Fe_1–*x*_Co_*x*_)_2_As_2_. These results contrast markedly with the observed higher *T*_c_ and wider superconducting range of the direct hole-doped system (Sr_1–*x*_ K_*x*_)Fe_2_As_2_ (37 K) [228, 229], shown in figure 14, and (Sr_1–*x*_ Cs_*x*_)Fe_2_As_2_ (37 K) [228]. The superconducting dome continues to the end member of this family, i.e., KFe_2_As_2_ [228, 229] and CsFe_2_As_2_ [228] at *Δn*/Fe = 0.5.

**Figure 15.**
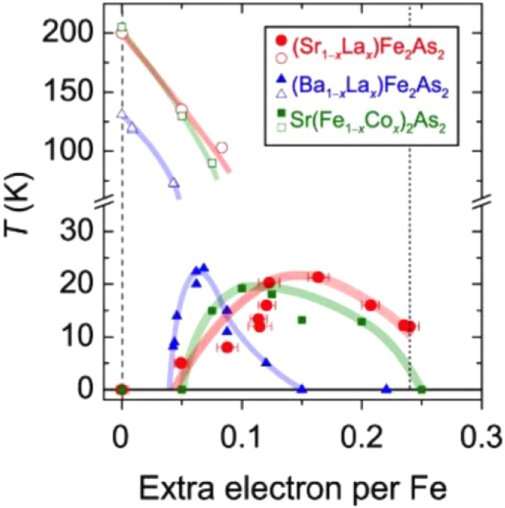
Electronic phase diagrams of (Sr_1–*x*_La_*x*_)Fe_2_As_2_ [22] and (Ba_1–*x*_La_*x*_)Fe_2_As_2_ [20] epitaxial films. The *T*_anom_ and 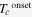 values are indicated by open and closed symbols, respectively. Those of Sr(Fe_1–*x*_Co_*x*_)_2_As_2_ [215] are shown for comparison. Reprinted with permission from [22]. Copyright 2013 by IEEE.

Another promising technique that we have developed for the indirect *RE* doping of SrFe_2_As_2_ and BaFe_2_As_2_ is the non-equilibrium pulsed laser deposition (PLD) method [20–22]. Hiramatsu *et al* [22] and Katase *et al* [20, 21] have succeeded in the homogeneous doping of *RE* dopants in the films fabricated by PLD, and they have reported observing a superconducting dome from underdoped to overdoped regions irrespective of the largely different ionic radii of Ba^2+^ (142 pm), Sr^2+^ (126 pm) and La^3+^ (116 pm). Here, we mention that CaFe_2_As_2_ epitaxial films could not be obtained by PLD [238]. Thin films of (Sr_1–*x*_La_*x*_)Fe_2_As_2_ (thickness = 200 nm) were grown on (La,Sr)(Al,Ta)O_3_ (LSAT) (001) single crystals at a film-growth temperature of 750 °C [22], while thin films of (Ba_1–*x*_*RE*_*x*_)Fe_2_As_2_ (thickness = 150–250 nm) were grown on MgO (001) single crystals at an optimized film-growth temperature of 850 °C [20, 21]. Further, (Sr_1–*x*_La_*x*_)Fe_2_As_2_ thin films were successfully obtained for 0.0 ≤ *x* ≤ 0.48 and (Ba_1–*x*_La_*x*_)Fe_2_As_2_ for 0.0 ≤ *x* ≤ 0.44. The lattice parameters showed monotonic decrease with increasing *x* (La content), thereby indicating that the substitution of the La^3+^ ion for the Sr^2+^ and Ba^2+^ ions was achieved in the epitaxial films [20, 22]. Figure 15 shows the electronic phase diagrams of (Sr_1–*x*_La_*x*_)Fe_2_As_2_ [22] and (Ba_1–*x*_La_*x*_)Fe_2_As_2_ [20] epitaxial films. Here, the doped carriers per Fe (=*x*/2) for these two compounds are plotted for comparison with the phase diagram of the directly electron-doped Sr(Fe_1–*x*_Co_*x*_)_2_As_2_ [215]. The antiferromagnetic ordering at *T*_anom_, which is determined by the anomaly in resistivity, is suppressed as the La content (*x*) increases. The maximum onset superconducting transition temperatures 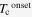 are 20.8 and 22.4 K at *x*/2 = 0.16 and 0.07 for (Sr_1–*x*_La_*x*_)Fe_2_As_2_ [22] and (Ba_1–*x*_La_*x*_)Fe_2_As_2_ [20], respectively. The maximum values of 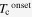 and the corresponding *x* values of (Sr_1–*x*_La_*x*_)Fe_2_As_2_ and (Ba_1–*x*_La_*x*_)Fe_2_As_2_ are very close to those reported for directly doped Sr(Fe_1–*x*_Co_*x*_)_2_As_2_ [215] (shown in figure 15) and Ba(Fe_1–*x*_Co_*x*_)_2_As_2_ [193] (not shown), respectively. In addition, the suppression rate of *T*_anom_ is similar for both compounds. These results sharply contrast with those of the 1111-type system, in which the maximum *T*_c_ of indirectly electron-doped SmFeAs(O_1–*x*_F_*x*_) (55 K) [115] is considerably higher than that for directly electron-doped Sm(Fe_1–*x*_Co_*x*_)AsO (17 K) [232]. These results can yield a clue to elucidate the mechanism of superconductivity in iron-based materials.

Katase *et al* [21] have also succeeded in obtaining (Ba_1–*x*_*RE*_*x*_)Fe_2_As_2_ thin films with *RE* = Ce, Pr and Nd with reduced solubility limits of *x* = 0.29, 0.18 and 0.13, respectively. Thin films of (Ba_1–*x*_*RE*_*x*_)Fe_2_As_2_ exhibited superconductivity at 13.4, 6.2 and 5.8 K for Ce, Pr and Nd dopants, respectively [21]. The *T*_c_ values of *RE*-doped SrFe_2_As_2_ and BaFe_2_As_2_ are listed in table 9.

**Table 9.** The maximum superconducting transition temperature *T*_c_ (K) of rare-earth (*RE*) doped (*AE*_1–*x*_ *RE*_*x*_)Fe_2_As_2_, where *AE*=Ca, Sr and Ba; and *RE*=La, Ce, Pr and Nd.

	La	Ce	Pr	Nd
Ca	45 [23]	37 [234]	49 [236]	
Sr	22 [19]			
Ba	22.4 [20]	13.4 [21]	6.2 [21]	5.8 [21]

**Figure 16.**
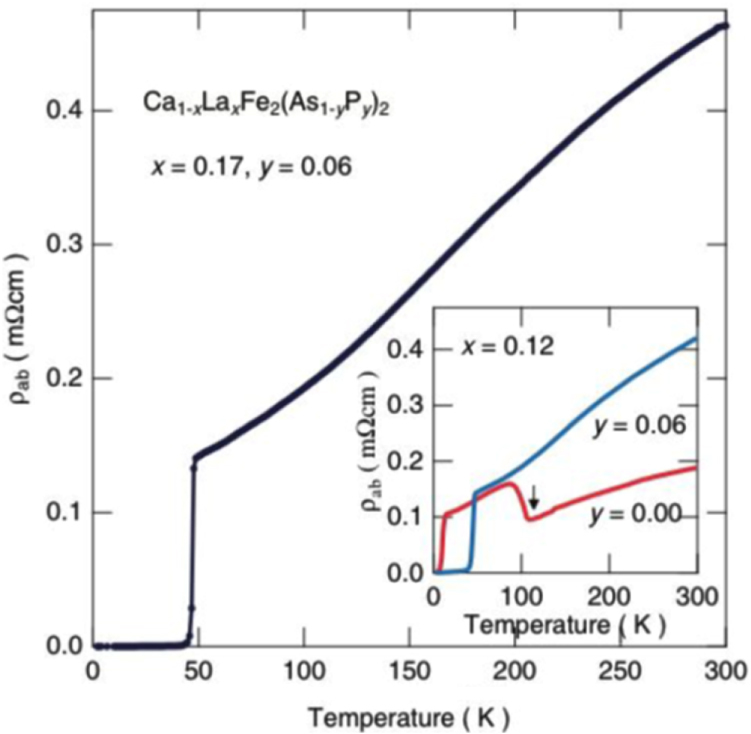
Temperature dependence of electrical resistivity *ρ*_*ab*_ for (Ca_1–*x*_La_*x*_)Fe_2_(As_1–*y*_P_*y*_)_2_ (*x* = 0.17 and *y* = 0.06) [23]. The resistivity starts to decrease at a transition temperature 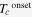 of 48 K and becomes zero below 45 K. The inset shows *ρ*_*ab*_ for *x* = 0.12 and *y* = 0.00 and 0.06. The arrow indicates the antiferromagnetic/tetragonal–orthorhombic structural transition. Reprinted with permission from Macmillan Publishers Ltd: [23], Copyright 2014.

**Figure 17.**
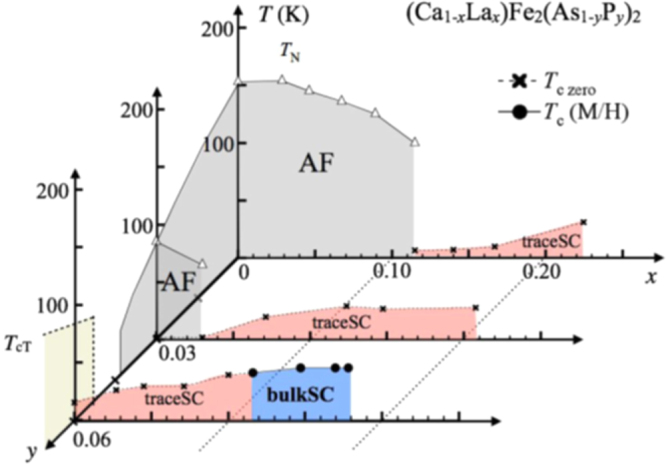
*T*–*x*–*y* electronic phase diagrams for (Ca_1–*x*_La_*x*_)Fe_2_(As_1–*y*_P_*y*_)_2_. *T*_*c*_(*M*/*H*) denotes the bulk superconducting transition temperature determined from the magnetization. 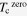 denotes the temperature below which the electrical resistivity becomes negligibly small. *T*_N_ represents the antiferromagnetic and structural transition temperature. *T*_cT_ denotes the transition temperature at which the high-temperature uncollapsed tetragonal (ucT) phase transforms into the low-temperature collapsed tetragonal (cT) phase. AF and SC indicate the antiferromagnetic and superconducting phases, respectively. Reprinted with permission from Macmillan Publishers Ltd: [23], Copyright 2014.

A conventional melt-growth method can be used to achieve indirect *RE* doping of CaFe_2_As_2_, although the obtained samples do not exhibit bulk superconductivity. In this project, two kinds of indirect doping, i.e., aliovalent La doping at the Ca sites and isovalent P doping at the As sites, were combined to induce bulk superconductivity in CaFe_2_As_2_ [23]. The substitution of aliovalent La for Ca resulted in electron doping without leading to a change in the lattice parameters because the ionic radius of La^3+^ (116 pm) and Ca^2+^ (112 pm) are similar, while the substitution of isovalent P for As resulted in a decrease in the lattice parameters without leading to a change in the number of carriers. This enabled us to tune the number of charge carriers and lattice parameters independently, and thus to optimize superconductivity in (Ca_1–*x*_La_*x*_)Fe_2_(As_1–*y*_P_*y*_)_2_, which resulted in bulk superconductivity at *x* = 0.17 and *y* = 0.06. The resistivity shows a sharp drop at 48 K and becomes zero at 45 K [23], as shown in figure 16. A clear diamagnetic signal, together with robust diamagnetism against the increase in magnetic field, is the reason for the bulk superconductivity at 45 K in this material [23]. Figure 17 shows the *T*–*x*–*y* phase diagram of (Ca_1–*x*_La_*x*_)Fe_2_(As_1–*y*_P_*y*_)_2_ [23]. Bulk superconductivity emerges in the range of 0.12 ≤ *x* ≤ 0.18 and *y* = 0.06.

Angle-resolved photoemission spectroscopy (ARPES) [212] revealed that (Ca_0.82_La_0.18_)Fe_2_(As_0.94_P_0.06_)_2_ with *T*_c_ = 45 K possesses only cylindrical hole- and electron-like Fermi surfaces (FS). The size of the *β* hole-like FS is nearly the same as that of the *ε* electron-like FS, and both FS have a weak *k*_*z*_ dispersion, thus giving rise to a quasi-nesting. This feature is similar to that for directly electron-doped Ba(Fe_1–*x*_ Co_*x*_)_2_As_2_ (*T*_c_ = 23 K). Sunagawa *et al* [212] pointed out that a noticeable difference between (Ca_0.82_La_0.18_)Fe_2_(As_0.94_P_0.06_)_2_ and Ba(Fe_1–*x*_Co_*x*_)_2_As_2_ is the dimensionality of the inner hole-like FS; the inner hole-like FS (*α*_2_) of the former shows a cylindrical shape, while that of the latter shows a strong *k*_*z*_ dispersion and is closed near the *Γ* point. It has been suggested that the tendency toward quasi-nesting between *α*_2_ and *β*, together with *β* and ε, can induce high *T*_c_ in (Ca_0.82_La_0.18_)Fe_2_(As_0.94_P_0.06_)_2_ [212].

#### 3.1.4. New type of 112 IBSC

**Figure 18.**
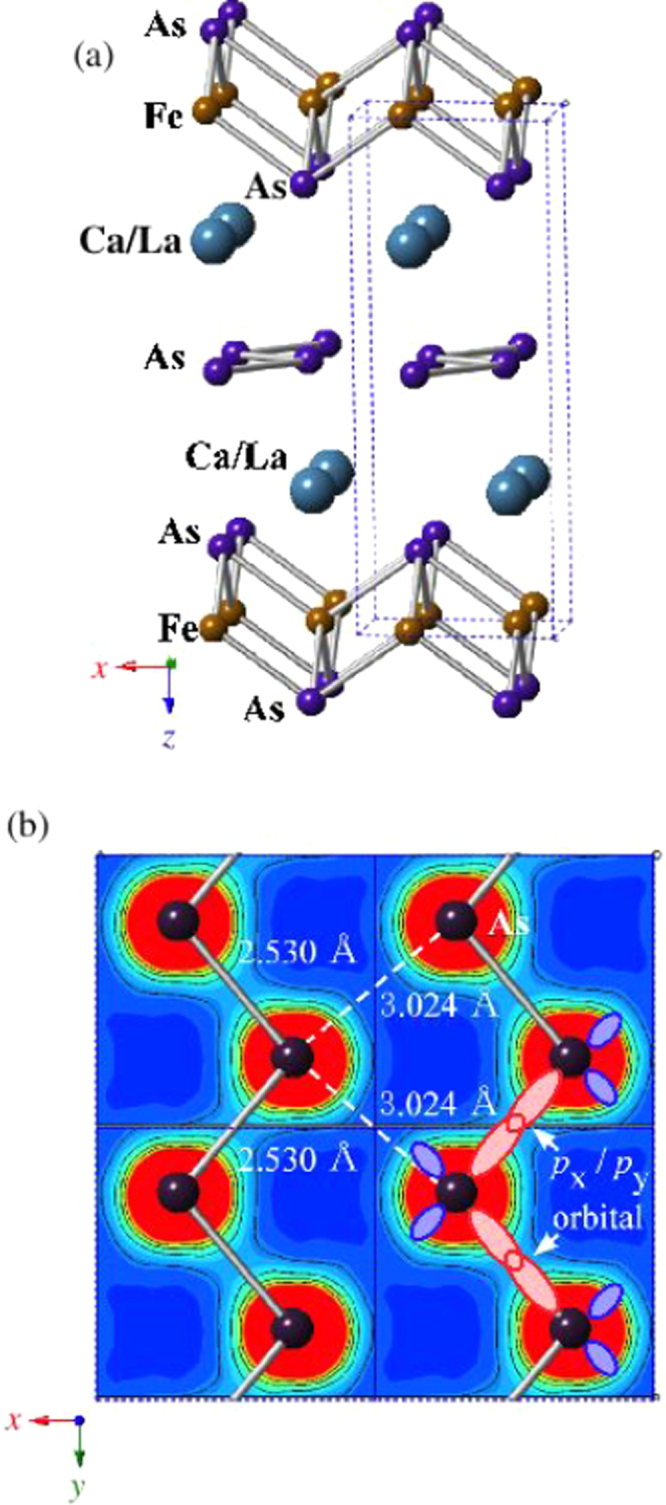
(a) The crystal structure of (Ca_1–*x*_La_*x*_)FeAs_2_ (monoclinic, space group P2_1_). (b) Top view of the arsenic zigzag chains. Arsenic 4*p*_*x*_ and 4*p*_*y*_ orbitals are schematically shown. The background color contour map shows the charge distributions obtained by synchrotron x-ray diffraction analysis. The charge accumulation between the adjacent As atoms in the zigzag chains suggests the formation of covalent bonds. Reprinted with permission from [25]. Copyright 2013 by the Physical Society of Japan.

The prominent achievements of the FIRST Project include the discovery of the 112-type iron arsenide superconductor (Ca_1–*x*_La_*x*_)FeAs_2_ [25], whose structure is shown in figure 18, and the enhancement of superconducting transition temperature *T*_c_ up to 47 K by the simultaneous La and Sb doping of the 112 phase [26, 27]. In this subsection, we overview the crystal structure and superconducting properties of the newly discovered 112 phase.

The superconductivity of the 112 phase was first reported in (Ca_1–*x*_La_*x*_)FeAs_2_ and (Ca_1–*x*_Pr_*x*_)FeAs_2_ by Katayama *et al* [25] and Yakita *et al* [239], respectively. The substitution of a rare-earth element is essential to obtaining the 112 phase [25, 239]. A conventional melt-growth technique was used to obtain tiny single crystals [25, 239]. (Ca_1–*x*_La_*x*_)FeAs_2_ exhibited superconductivity at *T*_c_ = 34 K [25], while (Ca_1–*x*_ Pr_*x*_)FeAs_2_ exhibited *T*_c_ of ~20 K with a broad resistive transition [239]. In a subsequent study, Sala *et al* [240] performed a high-pressure synthesis and obtained (Ca_1–*x*_*RE*_*x*_)FeAs_2_ for *RE* = La–Gd. Moreover, (Ca_1–*x*_*RE*_*x*_)FeAs_2_ for *RE* = Pr, Nd, Sm, Eu and Gd showed superconductivity at 10–15 K with a small shielding fraction of 5–20% [27, 240], while (Ca_1–*x*_Ce_*x*_)FeAs_2_ did not exhibit superconductivity [27, 240]. The *T*_c_ of (Ca_1–*x*_*RE*_*x*_)FeAs_2_ is summarized in table 10.

**Table 10.** The superconducting transition temperature *T*_c_ (K) of RE-doped (Ca_1–*x*_*RE*_*x*_)FeAs_2_ and *RE*- and Sb-doped (Ca_1–*x*_*RE*_*x*_)Fe(As_1–*y*_Sb_*y*_)_2_ as determined by magnetic measurements.

*RE*	(Ca_1–*x*_*RE*_*x*_)FeAs_2_	(Ca_1–*x*_*RE*_*x*_)Fe(As_1–*y*_Sb_*y*_)_2_
La	34 [25]	47 [27]
Ce		43 [27]
Pr	20 [239]	43 [27]
Nd	11.9 [240]	43 [27]
Sm	11.6 [240]	
Eu	9.3 [240]	
Gd	12.6 [240]	

The 112-type (Ca_1–*x*_*RE*_*x*_)FeAs_2_ compound crystallizes in a monoclinic structure with the space group of 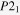 (no. 4) [25] or 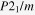 (No. 11) [239, 240]. The structure consists of alternately stacked FeAs and zigzag As bond layers with a Ca/La layer between them, as shown in figure 18(a). The most prominent feature of this structure is the presence of the one-dimensional zigzag As chains along the *b*-axis, as shown in figure 18(b). The short As–As bond length of approximately 2.53 Å indicates the formation of arsenic single bonds where arsenic has a formal valence of As^–^ (4*p*^4^ configuration). The presence of two unpaired electrons in As^–^ underlies the formation of two chemical bonds per As atom that yield a zigzag chain. In contrast, the arsenic at FeAs layers forms the As^3–^ valence state with the filled 4*p*^6^ configuration. Thus, the chemical formula of the 112 phase can be written as (Ca^2+^_1–*x*_ *RE*^3+^_*x*_)(Fe^2+^As^3–^)As^–^ · *xe*^–^ with excess charge of *xe*^–^/Fe, which is injected into the superconducting FeAs layers. This formula can be compared with that of the 1111-type fluoride, (Ca^2+^_1–*x*_ *RE*^3+^_*x*_)(Fe^2+^As^3–^)F^–^ · *xe*^–^ [137, 241], where F^–^ with filled 2*p*^6^ orbitals forms an undistorted square network. In this manner, the 112-type structure can be related to the 1111-type structure of CaFeAsF. However, the chemical bonding of the intermediary layer is completely different between CaFeAsF and CaFeAs_2_; the intermediary CaF layers consist of strong ionic bonds, while the CaAs layers consist of zigzag As chains with covalent bonds, which are weakly coupled to the adjacent Ca layers. Thus, the interlayer distance between the adjacent FeAs layers of (Ca_1–*x*_La_*x*_)FeAs_2_ (~10.35 Å) [27] is considerably larger than that of CaFeAsF (~8.6 Å) [137], but is comparable to that of Ca_10_(Pt_4_As_8_)(Fe_2–*x*_Pt_x_As_2_)_5_ (~10 Å) with the Pt_4_As_8_ intermediary layers exhibiting a covalent nature [242, 243].

**Figure 19.**
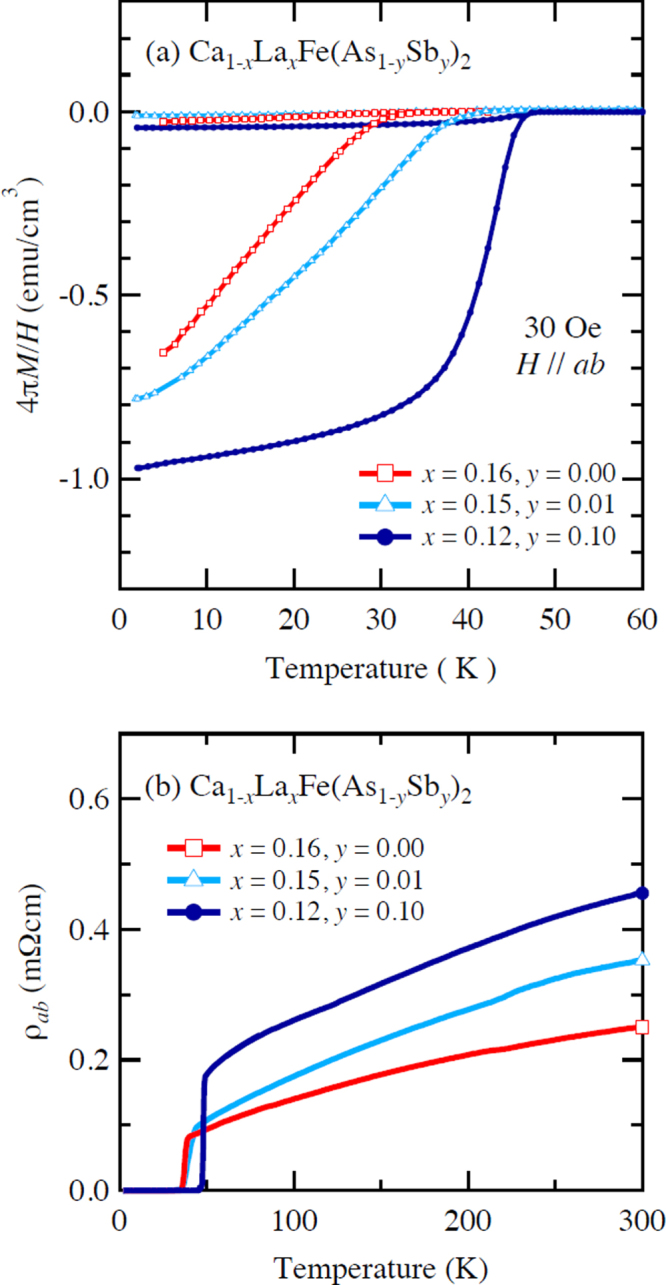
(a) Temperature dependence of the magnetization *M* of (Ca_1–*x*_La_*x*_)Fe(As_1–*y*_Sb_y_)_2_ measured at a magnetic field *H* of 30 Oe parallel to the *ab* plane under zero-field-cooling and field-cooling conditions. (b) Temperature dependence of the electrical resistivity *ρ*_*ab*_ of (Ca_1–*x*_La_*x*_)Fe(As_1–*y*_Sb_*y*_)_2_ parallel to the *ab*-plane. Reprinted with permission from [27]. Copyright 2014 by the Physical Society of Japan.

**Figure 20.**
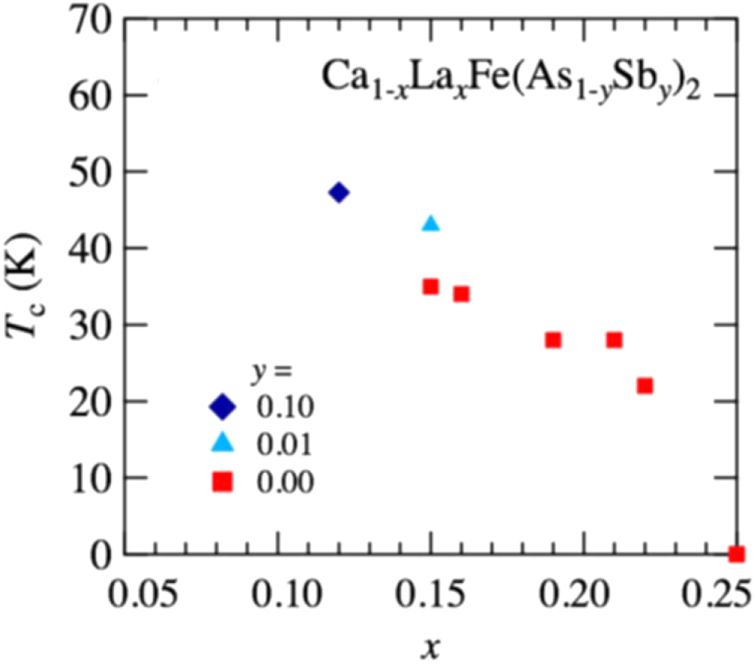
Dependence of the superconducting transition temperature *T*_*c*_ of (Ca_1–*x*_La_*x*_)Fe(As_1–*y*_Sb_*y*_)_2_ on the La content *x*. Reprinted with permission from [27]. Copyright 2014 by the Physical Society of Japan.

Kudo *et al* [26, 27] examined simultaneous doping, i.e., aliovalent La doping at the Ca sites and isovalent P or Sb doping at the As sites, and they observed that *T*_c_ increased up to 47 K for La- and Sb-doped (Ca_1–*x*_La_*x*_)Fe(As_1–*y*_Sb_*y*_)_2_ [27]. Figure 19(a) shows the temperature dependence of the magnetization of (Ca_1–*x*_La_*x*_)Fe(As_1–*y*_Sb_*y*_)_2_. The La-doped sample without Sb (*y* = 0) shows superconductivity at *T*_c_ = 34 K. The Sb doping results in an increase in *T*_c_ to 43 K for *y* = 0.01 and 47 K for *y* = 0.10. The enhancement of *T*_c_ is also evident in the electrical resistivity *ρ*_*ab*_ of the La-doped *y* = 0.10 sample. The resistivity *ρ*_*ab*_ exhibits a sharp drop at 49 K, and zero resistivity is observed at 47 K, as shown in figure 19(b). *T*_c_ was also enhanced by Sb doping of (Ca_1–*x*_*RE*_*x*_)Fe(As_1–*y*_Sb_*y*_)_2_ for *RE* = Ce, Pr and Nd [27], as summarized in table 10. Figure 20 shows the *T*–*x* phase diagram of (Ca_1–*x*_*RE*_*x*_)Fe(As_1–*y*_Sb_*y*_)_2_ [27]. The 112 phase can be obtained at *x* ≥ 0.15 for *y* = 0.0. The superconducting transition temperature *T*_c_ is highest (35 K) at the lowest boundary of *x* = 0.15, and it decreases monotonically with the La content *x*. Superconductivity disappears at *x* ≥ 0.25. The lower limit of *x* is extended down to *x* = 0.12 for *y* = 0.10, at which the highest *T*_c_ of 47 K is observed [27].

Recently, Zhou *et al* [244] have successfully grown large single crystals of (Ca_1–*x*_La_*x*_)FeAs_2_ with *T*_c_ = 42.6 K. Using these crystals, they estimated a critical current density *J*_c_ of 3.5 ×
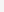
10^5^ A cm^−2^ from the magnetic hysteresis loops at 5 K, which indicates a strong bulk pinning. The anisotropic upper critical field *H*_*c*2_(0) was estimated to be 39 and 166 T for the out-of-plane (*H*//*c*) and in-plane (*H*//*ab*) directions, respectively. The *H*_c2_ anisotropy parameter *γ* was ~2 near *T*_c_. The moderate anisotropy and high *T*_c_ indicates the potential of (Ca_1–*x*_La_*x*)_FeAs_2_ for practical applications.

#### 3.1.5. 10-3-8 and 10-4-8 iron arsenide superconductors

The rich chemistry of arsenic allowed us to develop various iron arsenide superconductors. Arsenic exhibits a wide variety of chemical networks, which are known as catenation, depending on the number of valence electrons. For instance, neutral arsenic has three unpaired electrons (4*p*^3^), and thus, it forms three single bonds per As atom, thereby resulting in a buckled honeycomb network of *α*-As (trigonal, space group 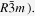 Monovalent arsenic (As^–^) has two unpaired electrons (4*p*^4^), and thus forms two single bonds per As atom, which results in either one-dimensional zigzag chains or cis–trans chains, or molecular-like 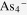 tetramers. The 112-type iron arsenide superconductor (Ca_1–*x*_La_*x*_)FeAs_2_ consists of As zigzag chains [25], as described in the previous subsection. Cis–trans chains can be observed in LaAgAs_2_ [245] and As_4_ tetramers in skutterudite CoAs_3_, for instance. Divalent arsenic (As^2–^) has one unpaired electron (4*p*^5^), and thus forms a single bond per As atom, which results in molecular-like 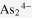 dimers, as seen in pyrite-type PtAs_2_. The 122-type iron arsenide superconductor CaFe_2_As_2_ exhibits the formation of molecular As_2_ between adjacent FeAs layers along the *c*-axis at the collapsed tetragonal phase transition [218, 234, 246]. The As_2_ molecular bonds are broken in the uncollapsed tetragonal phase. Here, the formal valence of arsenic is As^3–^, and the 4*p* orbitals are completely occupied, and thus, no direct chemical bonds are formed between As. The 10-3-8- and 10-4-8-type iron arsenide superconductors Ca_10_Pt_3_As_8_(Fe_2–*x*_Pt_*x*_As_2_)_5_ and Ca_10_Pt_4_As_8_(Fe_2–*x*_Pt_*x*_As_2_)_5_ consist of As_2_ dimers in the Pt_3_As_8_ and Pt_4_As_8_ intermediary layers, respectively [242, 247, 248].

Superconductivity in the 10-3-8 and 10-4-8 compounds was first reported by Kakiya *et al* [242], Löhnert *et al* [247] and Ni *et al* [248] for Pt-based Ca_10_Pt_3_As_8_(Fe_2–*x*_Pt_*x*_As_2_)_5_ and Ca_10_Pt_4_As_8_(Fe_2–*x*_Pt_*x*_As_2_)_5_. The 10-4-8 compound exhibited a maximum *T*_c_ of 38 K [242, 249]. The 10-3-8 compound showed a lower *T*_c_ of 13 K [242] by Pt doping of Ca_10_Pt_3_As_8_(Fe_2–*x*_Pt_*x*_As_2_)_5_. The *T*_c_ value was enhanced up to 30 K by La doping of (Ca_0.8_La_0.2_)_10_(Pt_3_As_8_)(Fe_2–*x*_Pt_*x*_As_2_)_5_ [249]. Three polymorphs have been identified in the 10-4-8 phase, i.e. tetragonal (*P*4/*n*), triclinic (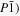 and monoclinic (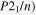 structures, while the 10-3-8 compound crystallizes in a triclinic (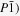 structure. Hieke *et al* [250] reported the observation of the 10-3-8 phase for Pd-based Ca_10_Pd_3_As_8_(Fe_2_As_2_)_5_, which exhibited superconductivity at 17 K by La substitution for Ca.

**Figure 21.**
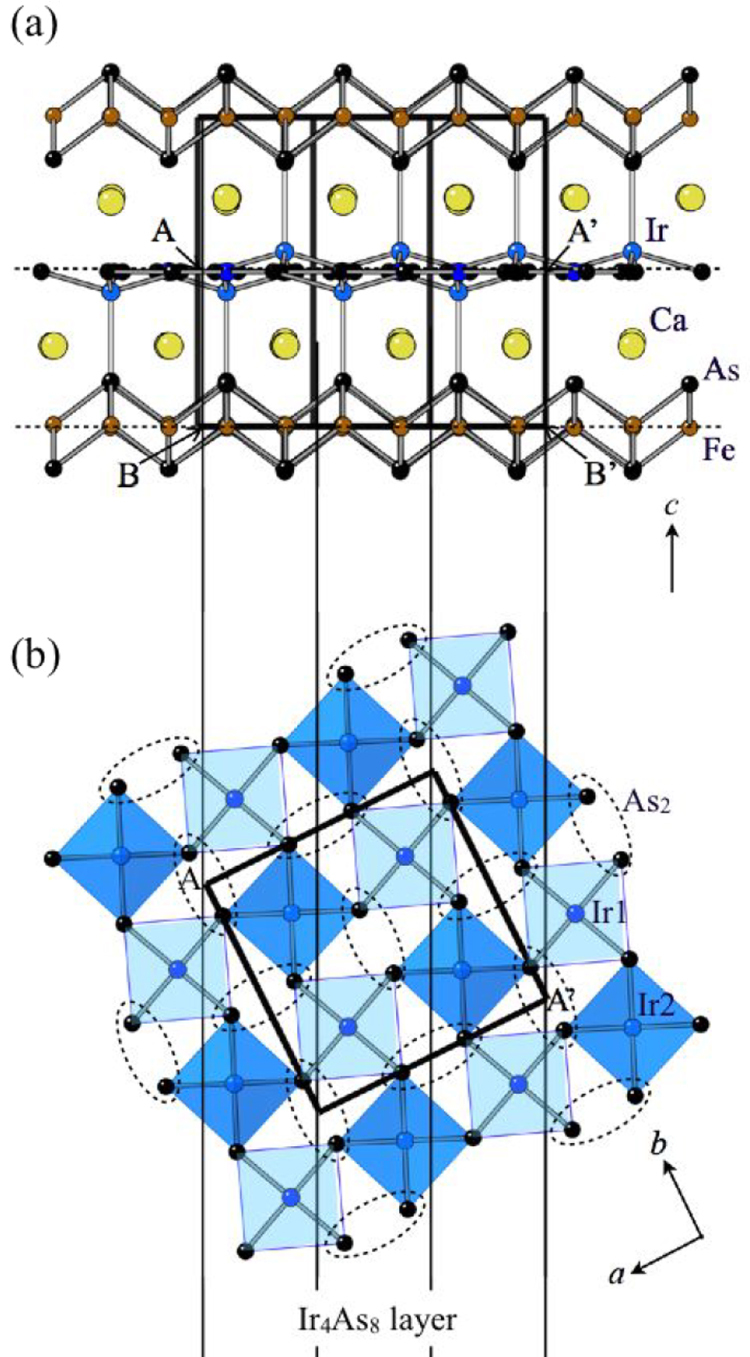
Crystal structure of 10-4-8 type Ca_10_Ir_4_As_8_(Fe_2–*x*_Ir_*x*_As_2_)_5_ with tetragonal structure (space group *P*4/*n*). (a), (b) Schematic overviews and the Ir_4_As_4_ layer, respectively. The blue and dark-blue hatches in (b) indicate IrAs_4_ squares with coplanar Ir1 and non-coplanar Ir2, respectively. The dashed ellipsoids in (b) represent As_4_ dimers. Reprinted with permission from Macmillan Publishers Ltd: [28], Copyright 2013.

**Figure 22.**
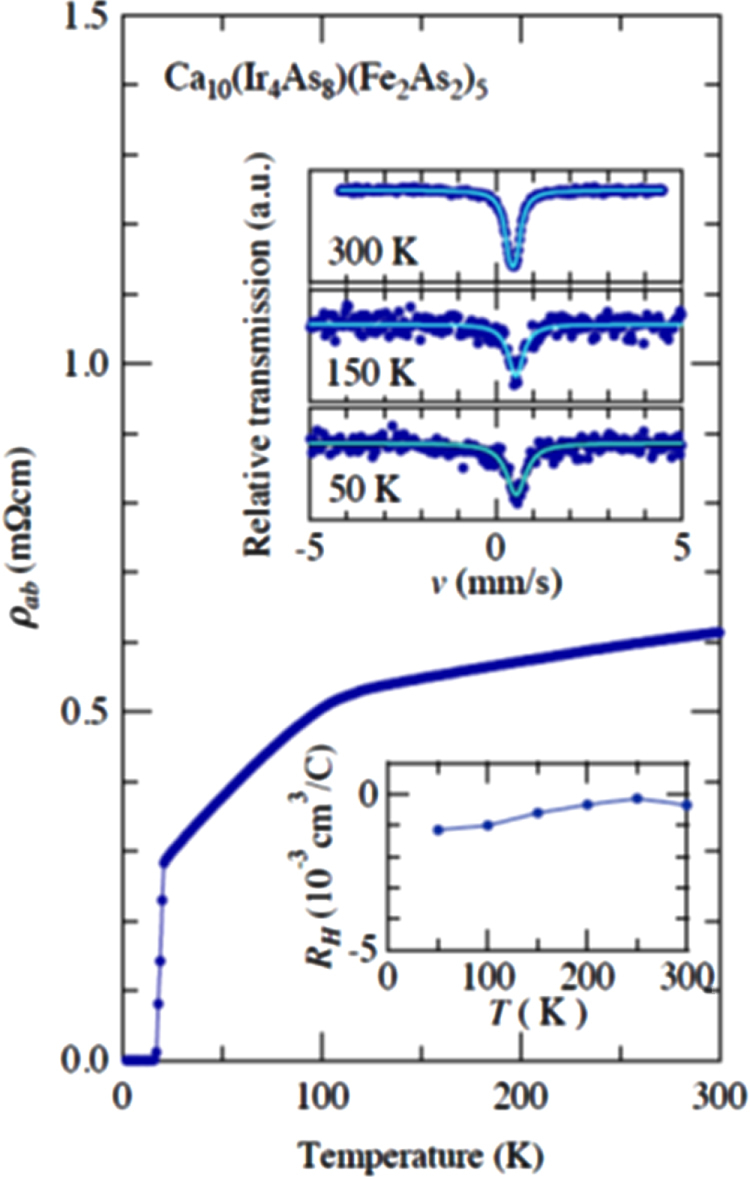
Temperature dependence of the electrical resistivity *ρ*_*ab*_ for Ca_10_Ir_4_As_8_(Fe_2–*x*_IrxAs_2_)_5_. The upper inset shows ^57^Fe-Mössbauer spectra together with fitting curves. The lower inset shows the temperature dependence of the Hall coefficient *R*_H_. Reprinted with permission from Macmillan Publishers Ltd: [28], Copyright 2013.

Under the aegis of the FIRST Project, Kudo *et al* [28] discovered a new member of the 10-4-8 family with Ir_4_As_8_ intermediary layers, i.e. Ca_10_Ir_4_As_8_(Fe_2–*x*_Ir_*x*_As_2_)_5_. The compound crystallizes in the tetragonal structure with the space group *P*4/*n*, which is isotypic to one of the polymorphs of Pt-based 10-4-8. The crystal structure, shown in figure 21, consists of characteristic IrAs_4_ squares, which are rotated alternately to form As_2_ dimers. Figure 22 shows the temperature dependence of the in-plane electrical resistivity *ρ*_*ab*_ of Ca_10_Ir_4_As_8_(Fe_2–*x*_Ir_*x*_As_2_)_5_ with *x* = 0.07–0.08 [28, 251]. The resistivity *ρ*_*ab*_ reached zero at 17 K. Magnetic measurements demonstrated large shielding signals below *T*_c_ = 16 K. The small and almost *T*-independent Hall coefficient *R*_H_, shown in the inset of figure 22, suggests overdoping, which most probably resulted in the low *T*_c_. Another feature of interest is the kink in *ρ*_*ab*_ at approximately 100 K. This kink is not due to antiferromagnetic ordering, since the singlet-peak structure of the ^57^Fe-Mössbauer spectra remained unchanged down to 50 K (as shown in the inset of figure 22), but due to a structural phase transition [251]. Katayama *et al* [251] performed single-crystal x-ray diffraction and identified that the transition is characterized by the displacement of Ir2 at the non-coplanar sites along the *c*-axis, thereby resulting in doubled periodicity along the *c*-axis without breaking the *P*4/*n* symmetry. The structural phase transition suggests that either Ir charge or orbital degrees of freedom are active in the intermediary Ir_4_As_8_ layers. Sawada *et al* [252] performed ARPES measurements of Ca_10_Ir_4_As_8_(Fe_2–*x*_Ir_*x*_As_2_)_5_, thereby demonstrating that the Fe 3*d* electrons in the FeAs layers form hole-like and electron-like Fermi surfaces at the zone center and corners, respectively, as commonly observed in iron arsenide superconductors; Ir 5*d* electrons are metallic and glassy most probably due to atomic disorder related to the Ir 5*d* orbital instability.

Another achievement of the FIRST Project is the growth of superconducting nanowhiskers of Ca_10_Pt_4_As_8_(Fe_1.8_Pt_0.2_As_2_)_5_ by Li *et al* [253]. The typical whiskers obtained had a length of 0.1–2.0 mm, width of 0.4–5.0 *μ*m and thickness of 0.2–1.0 *μ*m. High-resolution transmission electron microscopy (TEM) images showed that the whiskers exhibited excellent crystallinity and that whisker growth occurred along the *a*-axis of the tetragonal (*P*4/*n*) structure. The whiskers exhibited superconductivity with *T*_c_ of 33 K, *H*_*c*2_ (*H*//*c*) of 52.8 T and *J*_c_ of 6.0 × 10^6^ A cm^−2^ (at 26 K). Since cuprate high-*T*_c_ whiskers are fragile ceramics, the present intermetallic superconducting whiskers with high *T*_c_ are more suitable for device applications.

#### 3.1.6. Intercalated FeSe superconductors

The structurally simplest FeSe with space group *P*4*/nmm* has become a very attractive material. Though pure FeSe shows low *T*_c_ (8 K), it can be drastically enhanced by a factor of 5 under external high pressure [147, 148]. Moreover, the single-layer FeSe film epitaxially grown on SrTiO_3_ substrates has a wide superconducting gap (∼20 meV), which implies that *T*_c_ could be as high as 65 K in thin films despite the bulk *T*_c_ being ∼40 K [149]. More recently, high *T*_c_ over 100 K has been reported in this type of single-layer FeSe [153]. Another high-*T*_c_ bulk SC derived from FeSe is intercalated *A*_x_Fe_2−*y*_Se_2_ with ThCr_2_Si_2_ structure (but classified to 245-type due to the exact composition) that is synthesized at a high temperature (∼1300 K) but is only available for the large-sized monovalent metals such as *A* (K, Rb, Cs and Tl) [154–157, 254–256]. The bulk *T*_c_ of *A*_*x*_Fe_2−*y*_Se_2_ is ∼30 K and the average crystal structure is body-centered tetragonal (space group *I4/mmm*) [154]. However, the synthesis of FeSe intercalated by smaller alkali metals such as Li and Na has not succeeded using conventional high temperature processes.

The low-temperature method is another approach to intercalation, which is widely used and is suitable for intercalating alkali and alkaline earth metals, even those with small ionic radii. Many superconductors such as *A*_*x*_C_60_ and *A*_*x*_*M*N*X* (*A*: Li–K, Ca–Ba, Yb and Eu; *M*: Ti, Zr and Hf; and *X*: Cl, Br and I) were obtained through this method [55, 257]. Among them, the ammonothermal method, which uses liquid ammonia as a solvent and makes the starting materials react under high pressure in an autoclave, is a useful way to prepare the meta-stable and/or non-equilibrium materials. The relatively mild reaction keeps the host structure intact; therefore, the pure charge transfer without destroying the conductive layer is expected to favor the higher *T*_c_.

Application of this method to intercalate *A* into FeSe was first carried out by a group from the Institute of Physics, China [258]. They obtained several superconductors with a higher *T*_c_ of 30–46 K (FeSe intercalated by Li, Na, K, Ca, Sr, Ba, Eu, and Yb) compared with the samples prepared by the high-temperature method. In this report, they demonstrated that the ammonothermal method was useful to synthesize the intercalated FeSe superconductors with relatively high *T*_c_, while they did not mention remaining ammonia molecules or ions in their early stage of this research. Subsequently, they studied K_*x*_Fe_2_Se_2_+NH_3_ systematically and found two superconducting phases, K_0.3_Fe_2_Se_2_(NH_3_)_0.47_ (*T*_c_ = 44 K) and K_0.6_Fe_2_Se_2_(NH_3_)_0.37_ (*T*_c_ = 30 K), where they noted that the most important factor to control *T*_c_ was the potassium content [259]. Then, they prepared ammonia-free K_0.3_Fe_2_Se_2_ and K_0.6_Fe_2_Se_2_ by removing NH_3_ at 200°C completely and showed the same *T*_c_. The theoretical and/or empirical reason for higher *T*_c_ of K_0.3_Fe_2_Se_2_(NH_3_)_0.47_ has not yet been reported.

**Figure 23.**
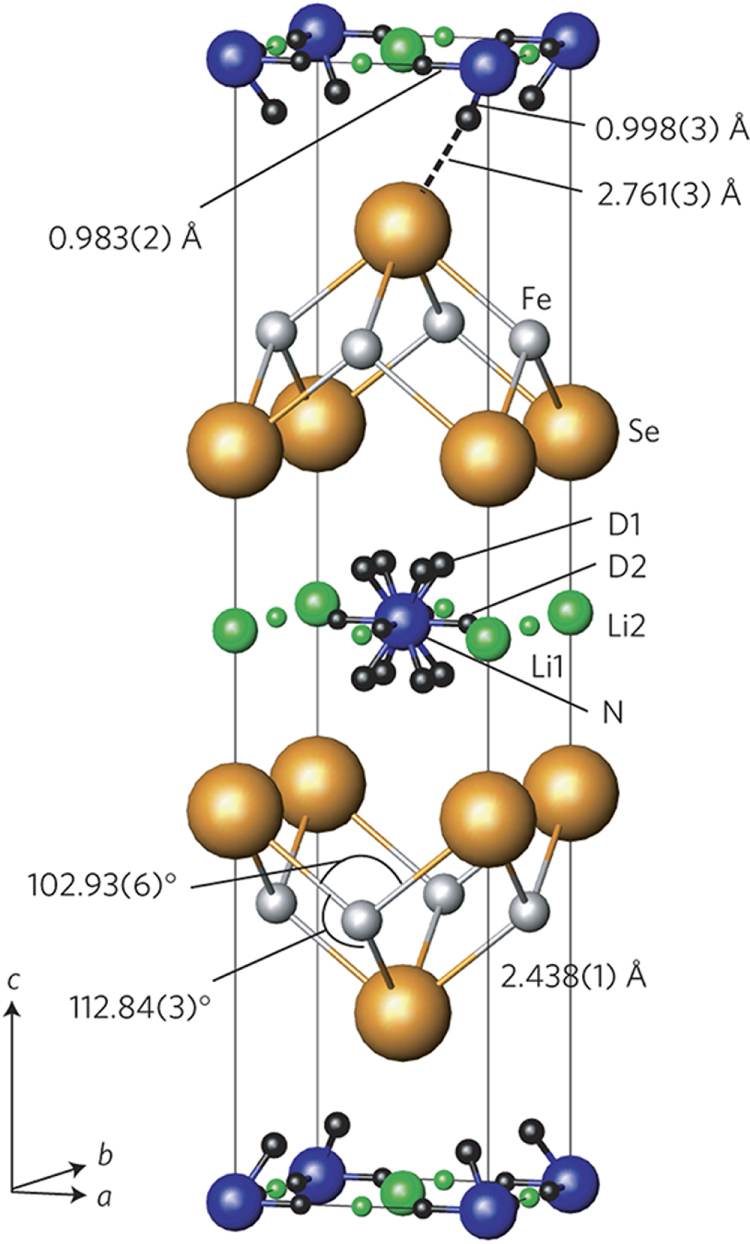
The 298 K crystal structure of Li_0:6(1)_(ND_2_)_0:2(1)_(ND_3_)_0:8(1)_Fe_2_Se_2_. Refinement was against neutron powder diffraction data (GEM instrument). In the model each square prism of Se atoms contains either an [ND_2_] anion or an ND_3_ molecule and these are both modeled as disordered over four orientations. The sizes of the spheres representing the Li atoms are in proportion to their site occupancies. Reprinted with permission from Macmillan Publishers Ltd: [260], Copyright 2013.

**Figure 24.**
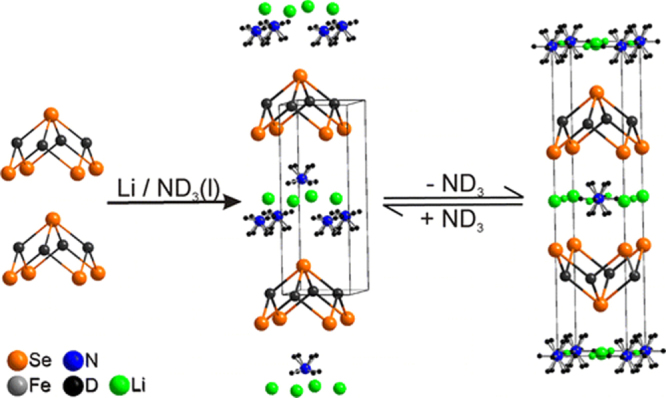
Schematic of the intercalation of lithium and ammonia into FeSe. Reprinted with permission from [261]. Copyright 2014 by the American Chemical Society.

In contrast, a group from the University of Oxford, UK, concentrated on the FeSe co-intercalated by Li and ammonia, and analyzed its precise crystal structure using the powder neutron diffraction [260, 261]. They synthesized Li_0.6_(ND_3_)_0.8_(ND_2_)_0.2_Fe_2_Se_2_ (*T*_c_ = 43 K) by the ammonothermal method and showed its crystal structure as in figure 23. This crystal structure indicates that the resulting compound intercalates as not only the ammonia molecule but also the amide anion. Furthermore they found reversible adsorption and desorption of ammonia by controlling pressure of ammonia at <−10 °C and obtained Li_0.6_(ND_2.7_)_1.7_Fe_2_Se_2_ (ammonia-rich phase) with *T*_c_ = 39 K by exposing Li_0.6_(ND_3_)_0.8_(ND_2_)_0.2_Fe_2_Se_2_ (ammonia-poor phase) to 1 atm of ammonia (ND_3_) at −10 °C (see figure 24). By intercalating more ammonia, the spacing between Fe layers (*d*) increased from 8.26 Å of the poor phase to 10.59 Å of the rich phase. Though the *T*_c_ of the ammonia-poor phase is higher than that of the ammonia-rich phase, the lithium content is the same in both phases. This result looks to be inconsistent with Ying’s result [259] who noted that the *T*_c_ depends on the intercalated alkali metal content. While the difference of *T*_c_ between the poor and the rich phase is not so large (4 K) compared with the case of potassium (14 K), the effect of intercalated amide anion is also unclear.

**Figure 25.**
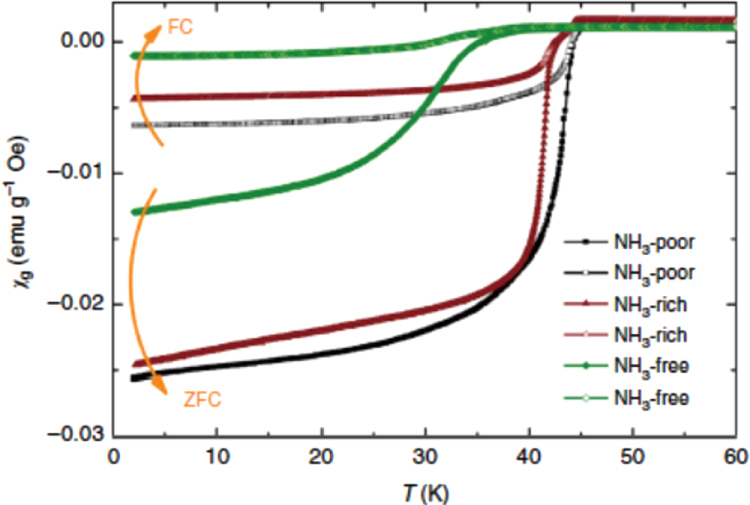
The magnetization curves of three Na/NH_3_ intercalated FeSe measured with the zero-field-cooling (ZFC) and field-cooling (FC) modes at *H* = 10 Oe. Reprinted with permission from Macmillan Publishers Ltd: [29], Copyright 2014.

In our research project, the group from Tokyo Institute Technology, Japan, synthesized three samples intercalated by sodium and ammonia as ammonia-free (phase I), ammonia-poor (phase II) and ammonia-rich phases (phase III) using the ammonothermal method and showed superconductivity with *T*_c_ = 37, 45 and 42 K, respectively (see figure 25) [29]. They prepared these phases by changing the Na/NH_3_ ratio (0.03 to 0.3 mol^−1^). For phase I, the reaction vessel was evacuated to ∼10^−2^ Pa after immersion for 3 h at 223–243 K. The chemical compositions measured by EPMA were Na_0.65_Fe_1.93_Se_2_ for phase I and Na_0.80_(NH_3_)_0.60_Fe_1.86_Se_2_ for phase II. Phase III was so unstable (decomposed easily even at 250 K) that the composition could not be defined. From powder x-ray diffraction measurements, the spacing between Fe layers (*d*) was 6.8, 8.7 and 11.1 Å for phases I, II and III, respectively. The lower *T*_c_ and the wider *d* of the ammonia-rich phase (phase III) than those of ammonia-poor phase (phase II) are similar to the case of Li.

These results are summarized in table 11. It has been considered that the *d* value could be a possible guideline to explore the high *T*_c_ superconductor. In IBSCs, the *d* of FeSe (*T*_c_ = 8 K), LiFeAs (*T*_c_ = 18 K), BaFe_2_As_2_ (*T*_c_ = 38 K), and SmFeAsO (*T*_c_ = 56 K) are 5.5, 6.4, 6.5 and 8.7 Å, respectively, which looks similar to the general rule for cuprate superconductors. Expecting that the material with wider *d* could reveal higher *T*_c_, Ogino *et al* [141–143] prepared IBSCs with a thick blocking layer with *d* = 15.5 to 24.5 Å. Unfortunately, the maximum *T*_c_ in their attempt was 43 K for Ca_6_(Sc,Ti)_5_O_y_Fe_2_As_2_ (*d* = 24.5 Å) (see section 3.1.1). In this case of these intercalates, the optimal *d* is 7.5–8.8 Å of the ammonia-poor phase. It looks as though *d* is not a critical factor to control *T*_c_ widely.

**Table 11.** Intercalated FeSe superconducting phases.

	NH_3_-rich			NH_3_-poor				NH_3_-free	
			Metal-poor		Metal-rich			
	*d*	*T*_c_	*d*	*T*_c_	*d*	*T*_c_	*d*	*T*_c_
Li^+^	∼9.0	39	—	—	∼8.3	44	—	—
Na^+^	∼11.1	42	—	—	∼8.7	45	∼6.8	37
K^+^	∼10.2	?	∼7.8	44	∼7.4	30	7.14	∼44
*AE*^2+^	—	—	8.0–8.4	35–40	∼10.3	38–39	—	—
*RE*^2+^	—	—	∼8.1	42	∼10.2	40–42	—	—

‘–’: none, ‘?’: unknown. The separation of nearest Fe layers d (Å) and *T*_c_ (K) of intercalated (A/AE/RE)x(NH3)y(NH2)zFe2Se2 (A: alkali metals, AE: alkaline earth metals, RE: rare earth metals) superconductors synthesized by the ammonothermal method shown in the literature [29, 258–261].

An important feature of these intercalates is none or very small deficiency of Fe compared to those synthesized by the conventional high temperature method. In the case of conventionally prepared (Tl, K)Fe_*x*_Se_2_, superconductivity emerges over *x* = 1.7 and the compound with smaller Fe content is an antiferromagnetic insulator [156]. The maximum Fe content of the compound prepared by the high temperature method is reported to be *x* ∼ 1.9 and the maximum *T*_c_ is 31 K. Zhang *et al* reported that the compound with excess Fe (K_0.87_Fe_2.19_Se_2_) which was prepared by the Bridgman method using Fe_1+*x*_Se as a starting material revealed a sharp drop of resistivity at 44 K, but zero resistivity was observed at a lower temperature (25 K) and the shielding volume fraction at >25 K was very small (<1%) [262]. These results suggest that a small deficiency of Fe is favorable to achieve high *T*_c_, which is due to the large amount of indirect electron doping efficiently. The ammonothermal method is profitable to prepare such a condition.

### 3.2. Superconductivity in layered titanium compounds

Since the discovery of high-*T*_c_ superconductivity in a layered perovskite oxide (La,Ba)_2_CuO_4_ with a CuO_2_ square lattice [263], numerous efforts have been dedicated to obtaining new superconducting families. Although this led to a series of discoveries of other high-*T*_c_ families such as MgB_2_ [196], fullerenes [264] and iron pnictides [4], the transition temperatures never exceed liquid nitrogen temperature. In addition, the mechanism of high *T*_c_ superconductivity in copper oxides remains unsolved and is still under debate despite intensive investigations.

**Figure 26.**
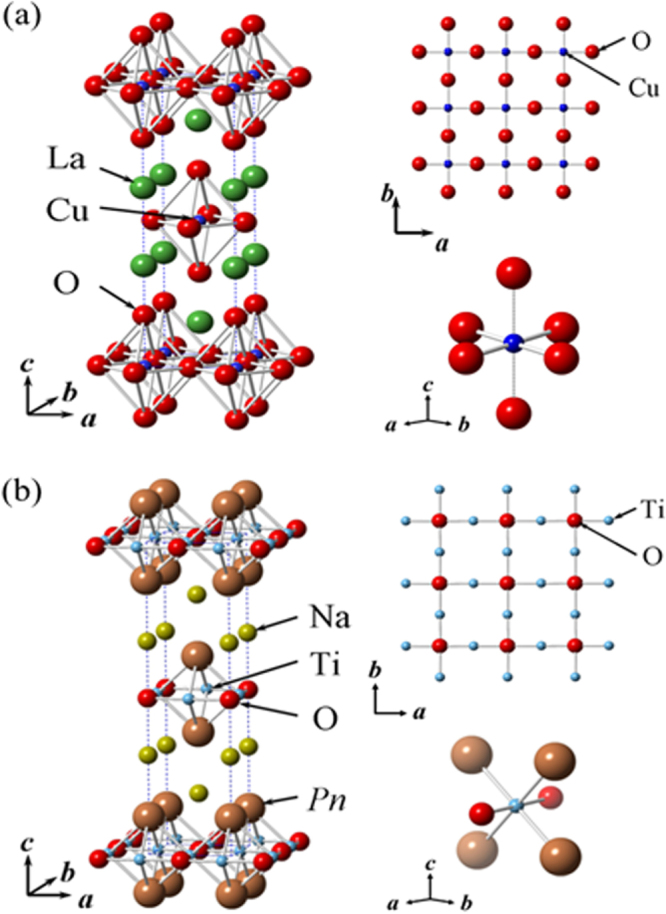
(a) Crystal structure of La_2_CuO_4_, CuO_2_ sheets (top) and CuO_6_ octahedron (bottom). (b) Crystal structure of Na_2_Ti_2_*Pn*_2_O, Ti_2_O sheets (top) and TiO_2_*Pn*_4_ octahedron (bottom).

Searching parent structures having a *d*^1^ square lattice may be a plausible strategy to access novel superconductivity, which is hole–electron symmetric with the *d*^9^ square lattice. Sr_2_VO_4_ with V^4+^ (*d*^1^), which is isostructural with La_2_CuO_4_ (figure 26(a)), is a promising candidate material though all attempts to inject carriers were so far unsuccessful [265, 266]. When the ligand field splitting energy (LFSE) for an octahedral environment is taken into account, however, one notices that the electronic structures of Sr_2_VO_4_ and La_2_CuO_4_ are quite different from each other. In the *d*^9^ case, one unpaired electron occupies one of the anti-bonding *e*_g_ orbitals, *x*^2^ − *y*^2^, which is widely separated in energy from the *z*^2^ orbital due to the Jahn–Teller effect and gives a half-filled electronic configuration. In the *d*^1^ case, one electron occupies the non-bonding *t*_2g_ (*xy*, *yz*, *zx*) orbitals. A certain octahedral distortion may be present, but is not enough to lift the orbital degeneracy significantly, so that the system should be better approximated by a 1/6-filled configuration.

**Figure 27.**
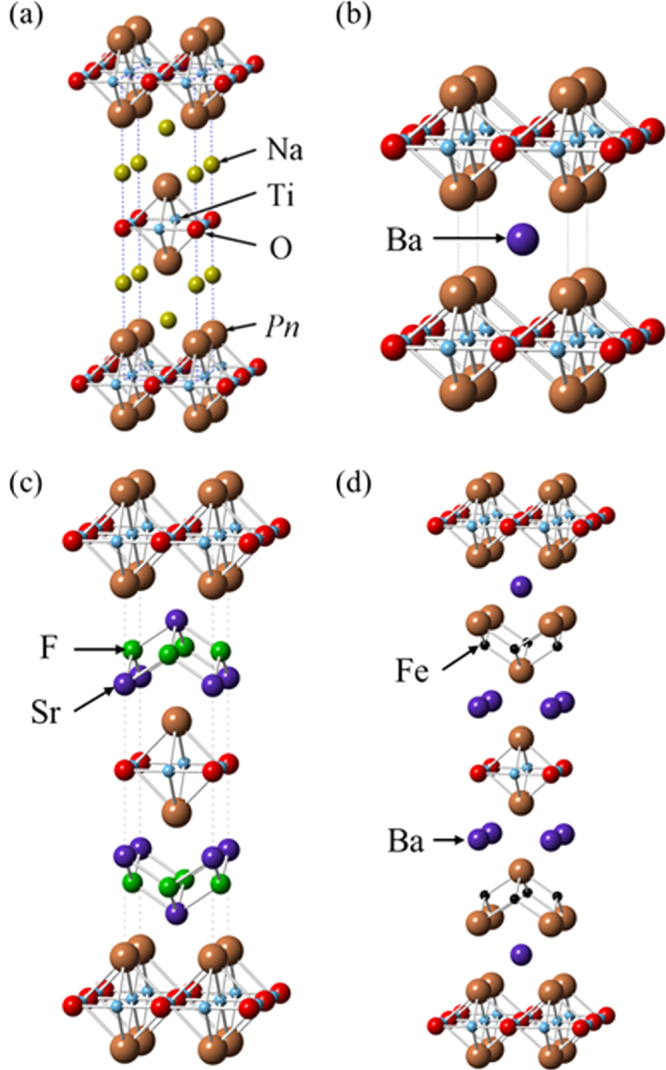
Crystal structures of (a) Na_2_Ti_2_*Pn*_2_O, (b) BaTi_2_*Pn*_2_O, (c) (SrF)_2_Ti_2_*Pn*_2_O, and (d) (Ba_2_Fe_2_As_2_)Ti_2_As_2_O.

In order to tune the *t*_2g_ orbital levels more drastically (than possible in oxides), we looked for mixed anionic compounds, where a *d*^1^ metal is coordinated octahedrally by two kinds of anion species. Due to the large difference between anions in terms of valence, electronegativity and ionic radius, a mixed-anion coordination geometry might provide a unique opportunity to split *t*_2g_ orbitals to a greater extent. Such a situation is realized in layered titanium oxypnictides Na_2_Ti_2_*Pn*_2_O (*Pn* = As, Sb), the structure of which is illustrated in figure 26(b) [267]. The Na_2_Ti_2_*Pn*_2_O structure appears to be similar to that of La_2_CuO_4_, but contains an inverse Ti_2_O square lattice. The trivalent titanium ion (*d*^1^) has an octahedral TiO_2_Sb_4_ coordination, and the TiO_2_Sb_4_ octahedra share edges to form a two-dimensional network. Like cuprate superconductors, [TiO_2_Sb_2_]^2–^ layers can be sandwiched by various block layers. For example, BaTi_2_As_2_O with a Ba^2+^ layer, (SrF)_2_Ti_2_As_2_O with a [(SrF)_2_]^2+^ layer, and (Ba_2_Fe_2_As_2_)Ti_2_As_2_O with a [Ba_2_Fe_2_As_2_]^2+^ layer are reported (figure 27) [268–271].

Unlike cuprate superconductors, known ‘parent’ compounds in the layered titanium oxypnictides are already metallic without carrier doping [272, 273]. Instead of superconductivity, they show anomalies in magnetic susceptibility and electric resistivity likely ascribed to charge density wave (CDW) and/or spin density wave (SDW) transitions. The density wave transition temperatures *T*_DW_ are 330 K for Na_2_Ti_2_As_2_O, 120 K for Na_2_Ti_2_Sb_2_O and 200 K for BaTi_2_As_2_O. It is hence expected that the suppression of density wave phase is a key to induce superconductivity. In 2012, we prepared a new compound BaTi_2_Sb_2_O and observed a bulk superconductivity transition at 1 K [31]. Doan *et al* independently showed the enhanced *T*_c_ of 5.5 K in Na-doped BaTi_2_Sb_2_O [274]. These reports have sparked a lot of investigation into superconductivity in layered titanium oxypnictides. In this section, we demonstrate the present status of our understanding of this new superconducting family from both experimental [31–33, 275–284] and theoretical [285–289] viewpoints.

#### 3.2.1. Superconductivity in BaTi_2_Sb_2_O

**Figure 28.**
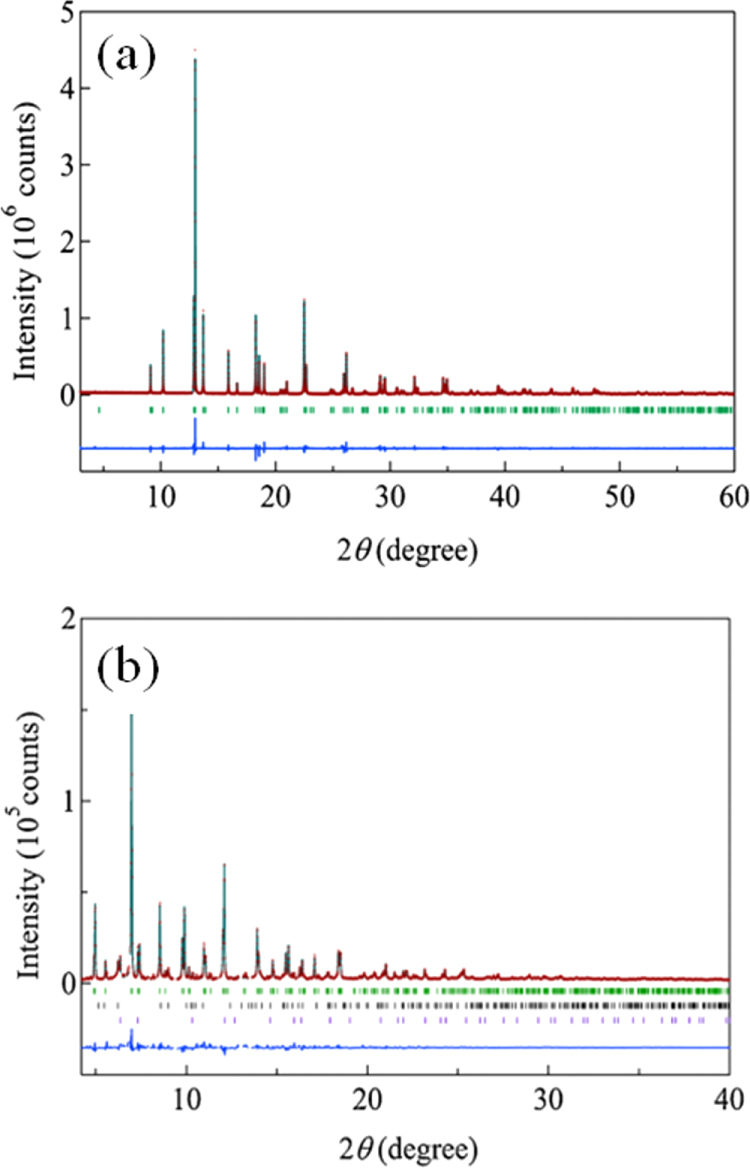
Refined synchrotron x-ray patterns of (a) BaTi_2_Sb_2_O [31], and (b) BaTi_2_Bi_2_O [33]. Reprinted with permission. Copyright 2012 for [31] and 2013 for [33] by the Physical Society of Japan.

**Figure 29.**
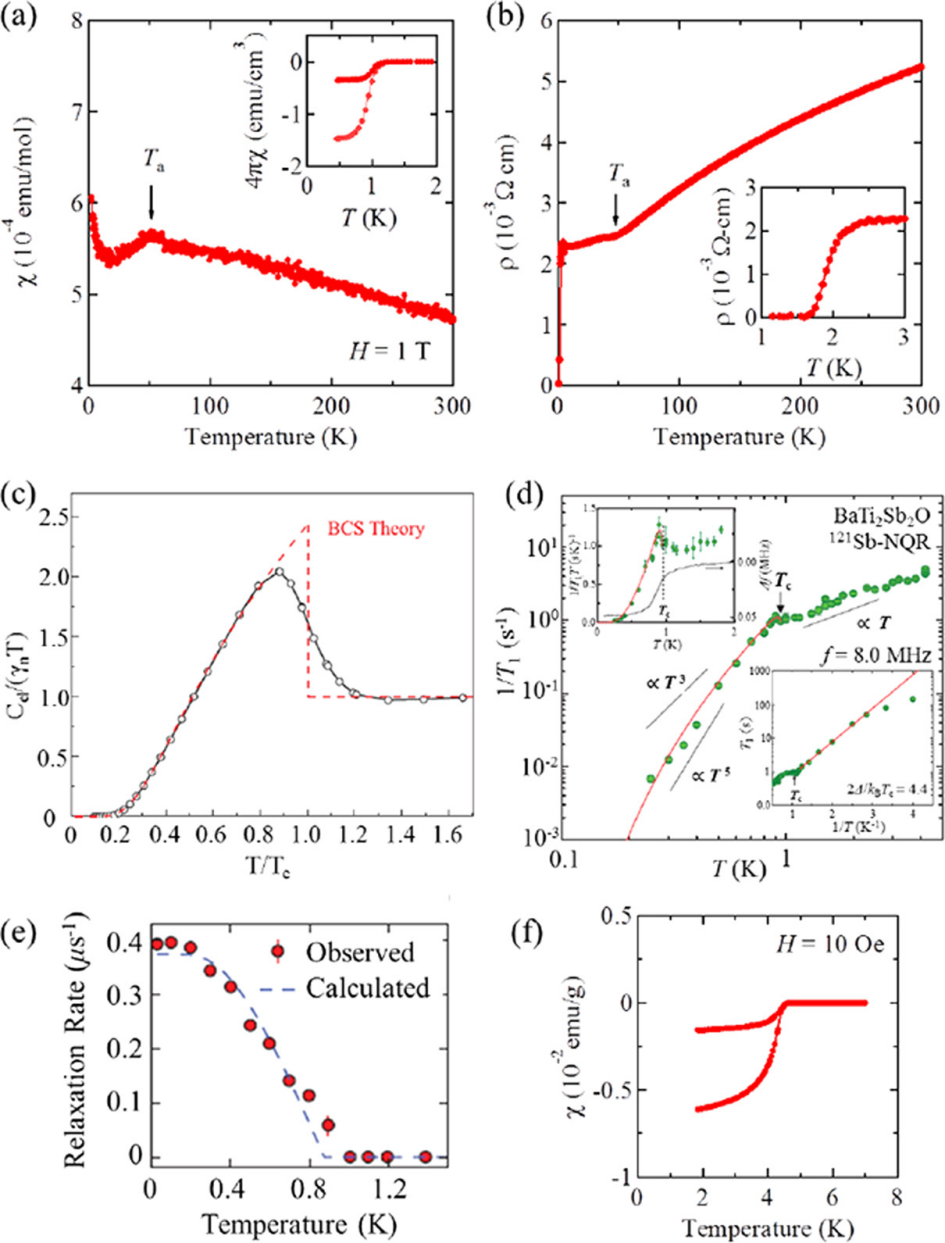
(a) Magnetic susceptibility and (b) electric resistivity of BaTi_2_Sb_2_O (taken from [31]). (c) Specific heat capacity of Ba_0.85_Na_0.15_Ti_2_Sb_2_O (taken from [278]). (d) ^121^Sb-NQR result: spin-lattice relaxation rate of BaTi_2_Sb_2_O, taken from [275]. (e) *μ*SR result: superconducting relaxation rate of BaTi_2_Sb_2_O. (f) Magnetic susceptibility of BaTi_2_Bi_2_O (taken from [33]). Reprinted with permission. Copyright 2012 for [31] and 2013 for [33] by the Physical Society of Japan. Copyright 2013 for [275] and [278] by the American Physical Society.

A pure phase of BaTi_2_Sb_2_O was synthesized by the conventional solid-state reaction method using BaO (99.99%), Ti (99.9%) and Sb (99.9%) in stoichiometric quantity [31]. A pellet specimen was wrapped in tantalum foil, sealed in a quartz tube, and typically heated at 1000 °C for 40 h, followed by controlled cooling at a rate of 50 °C h^−1^ to room temperature. The product is air and moisture sensitive. BaTi_2_Sb_2_O is tetragonal with lattice constants of *a* = 4.11039(2) Å, *c* = 8.08640(4) Å at room temperature. BaTi_2_As_2_O has larger cell parameters, *a* = 4.046 Å, *c* = 7.272 Å. Figure 28(a) shows the result of the synchrotron x-ray diffraction refinement for BaTi_2_Sb_2_O with the space group of *P*4/*mmm*. Magnetic susceptibility and resistivity for BaTi_2_Sb_2_O (figures 29(a) and 4(b)) show a distinct anomaly at around *T*_DW_ = 50 K, which should be related to a density wave transition. Upon further cooling, the *ρ*–*T* curve showed zero resistivity, indicating a superconducting transition. A large diamagnetic signal associated with the shielding effect was observed at *T*_c_ = 1 K. The shielding volume fraction is as large as 58%, providing firm evidence for bulk superconductivity in BaTi_2_Sb_2_O. Heat capacity measurements revealed a distinct peak at around *T*_c_, further supporting the bulk superconductivity.

#### 3.2.2. Nature of superconductivity in BaTi_2_Sb_2_O

It has been revealed from various experiments on BaTi_2_Sb_2_O that the superconducting state is classified to a fully gapped *s*-wave state. The analysis of the specific heat at *T*_c_ gives Δ*C*(*T*_c_)/*γT*_c_ ∼ 1.36, which is consistent with the BCS (Bardeen–Cooper–Schrieffer) weak coupling limit value of 1.43 [31]. Gooch *et al* demonstrated that the temperature variation of the electronic heat capacity on Ba_1–*x*_Na_*x*_Ti_2_Sb_2_O (*x* = 0 and 0.15) is well described by a weak coupling BCS function with 2*Δ*/*k*_B_*T*_c_ = 2.9, as shown in figure 29(c) [278]. Kitagawa *et al* reported that the temperature dependence of inverse spin–lattice relaxation time (1/*T*_1_) in ^121^Sb nuclear quadrupole resonance (NQR) shows a coherence peak just below *T*_c_ and decreases exponentially at low temperatures. From the slope of the plot, the magnitude of the superconducting gap is estimated to be 2*Δ*/*k*_B_*T*_c_ = 4.4 (figure 29(d)) [275]. A superconductivity-induced muon relaxation rate *σ*_sc_, which is proportional to the penetration depth as *λ*^–2^, shows robust bulk superconductivity below *T*_c_ ∼ 1 K (figure 29(e)) [277]. A fit to a BCS *s*-wave model in the weak coupling limit gave *T*_c_ = 0.87 ± 0.03 K and *σ*_sc_ (*T* = 0) = 0.37 ± 0.01 *μ*s^–1^. Strong electron correlation in the present material is suggested from the Wilson ratio of *R*_W_ = 2.21, which is much larger than *R*_W_ = 1 for the free-electron approximation [31].

#### 3.2.3. Density wave state

**Figure 30.**
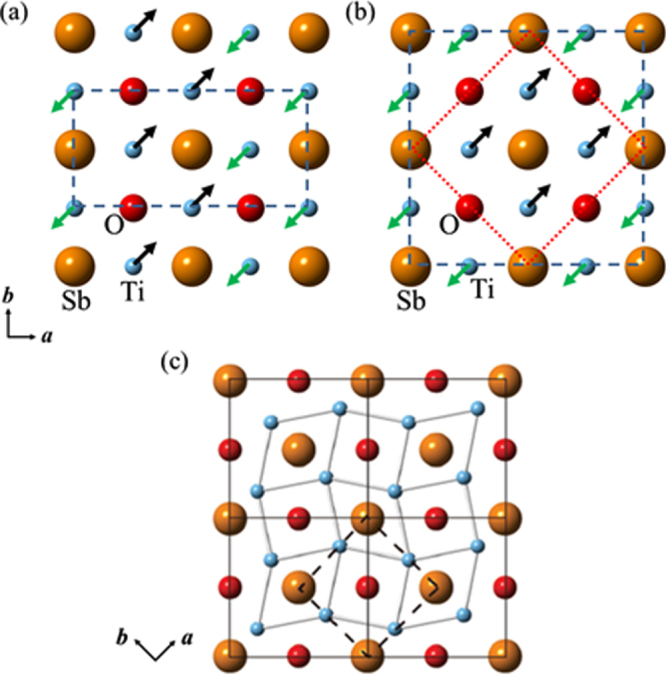
Theoretically proposed spin/charge density wave models for BaTi_2_Sb_2_O or BaTi_2_As_2_O. (a) A bicolinear type and (b) blocked checkerboard type SDW model [290, 291] (taken from [291]). (c)√2 × √2 × 1 superstructure as a result of CDW transition (taken from [292]). Reprinted with permission. Copyright 2013 for [291] by IOP Publishing and 2013 for [292] by the American Physical Society.

While the superconducting state is described well by the weak coupling BCS scheme, the nature of the pairing mechanism, namely, whether the superconductivity in BaTi_2_Sb_2_O is driven by the electron–phonon coupling or spin-fluctuation, remained unclear until recently. Theoretically, for BaTi_2_Sb_2_O, a magnetic instability associated with Fermi surface nesting, leading to a SDW state with a propagation vector of (*π*, 0) (figure 30(a)), was theoretically proposed by D Singh [290]. A sign-changing *s-*wave state within a scenario of spin-fluctuation mediated superconductivity was also suggested. The same type of bi-collinear antiferromagnetic state was shown for Na_2_Ti_2_Sb_2_O by X W Yan *et al*, while Na_2_Ti_2_As_2_O was suggested to have a blocked checkerboard antiferromagnetic state with a 2 × 2 magnetic unit cell (figure 30(b)) [291]. On the other hand, first-principles calculations of the phonon dispersions and electron–phonon coupling for BaTi_2_Sb_2_O by Subedi [292] revealed lattice instability near the zone corners, which leads to a charge-density wave phase with a √2 × √2 × 1 superstructure arising from a coherent distortion corresponding to elongation or compression of the Ti squares without an enclosed O, such that the Ti squares with O rotate either clockwise or counterclockwise as shown in figure 30(c).

**Figure 31.**
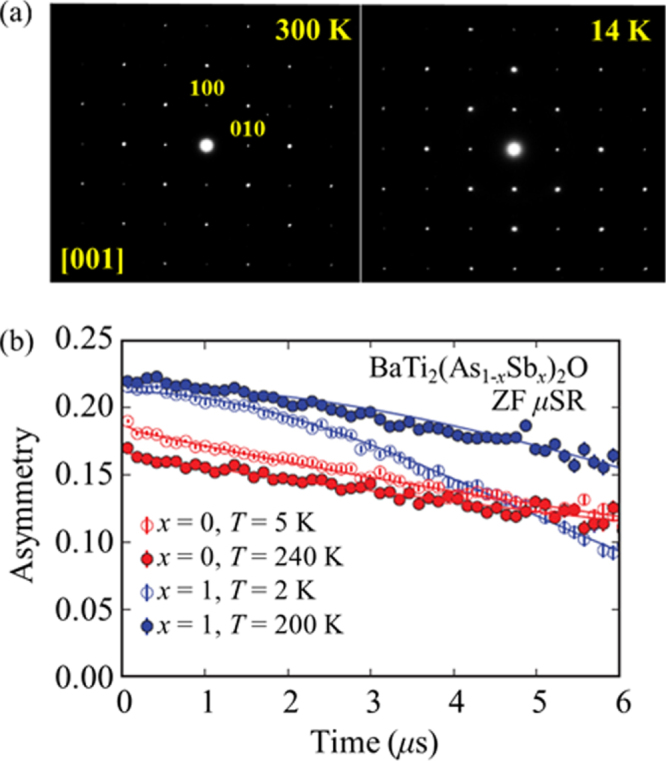
(a) Selected area electron diffraction patterns taken along the [001] axis for BaTi_2_Sb_2_O at 300 K and 14 K. (b) Asymmetry versus time spectra from zero-field muon spin relaxation measurements on BaTi_2_As_2_O and BaTi_2_Sb_2_O. Reprinted with permission from [277]. Copyright 2013 by the American Chemical Society.

As shown in figure 31(b), zero-field muon spin relaxation measurements showed no significant increase in relaxation rate at the density wave ordering temperature, indicating that the density wave is of the charge rather than spin type [277]. ^121/123^Sb-NQR measurements revealed that the in-plane four-fold symmetry is broken at the Sb-site below *T*_DW_ ∼ 40 K, without an internal field appearing at the Sb site, indicating a commensurate CDW ordering [275]. However, the absence of any superstructure peaks in high-resolution electron and neutron diffraction below *T*_DW_ (figure 31(a)) signifies that the charge density wave does not involve modulation of atomic arrangement, implying a nontrivial nature of the CDW state. Recent in-depth structural studies have further suggested that BaTi_2_As_2_O forms a symmetry-breaking nematic ground state that can be naturally explained as an intra-unit-cell nematic charge order with *d*-wave symmetry, pointing to the ubiquity of the phenomenon [293]. These findings, together with the key structural features in these materials being intermediate between the cuprate and iron-pnictide high-temperature superconducting materials, render the titanium oxypnictides an important new material system to understand the nature of nematic order and its relationship to superconductivity.

#### 3.2.4. Aliovalent cation/anion substitution

Since the ‘parent’ phase BaTi_2_Sb_2_O is a 1 K superconductor, coexisting with the CDW state with *T*_DW_ ∼ 50 K, it can be expected that the superconducting transition temperature can be enhanced by destabilizing the CDW phase. It was shown by Doan *et al* that the divalent Ba site in BaTi_2_Sb_2_O is substitutable by monovalent Na ions up to 33% [274]. The *T*_c_ in Ba_1–*x*_Na_*x*_Ti_2_Sb_2_O gradually increases with increasing *x* (hole concentration) and attained a maximum *T*_c_ of 5.5 K at *x* = 0.33. Although the CDW state is gradually destabilized by the Na substitution, it still persists (e.g., *T*_DW_ = 30 K at *x* = 0.25): *T*_DW_ forms a downward concave curve in the region of a higher *x*, showing a saturation tendency. The alkaline-earth metal substitution by K [279] and Rb [280] is also effective in raising *T*_c_. The K substitution (Ba_0.88_K_0.12_Ti_2_Sb_2_O) provides the highest *T*_c_ of 6.1 K, which is due to smaller chemical disorder because of the similarity in ionic radius between K^+^ and Ba^2+^.

**Figure 32.**
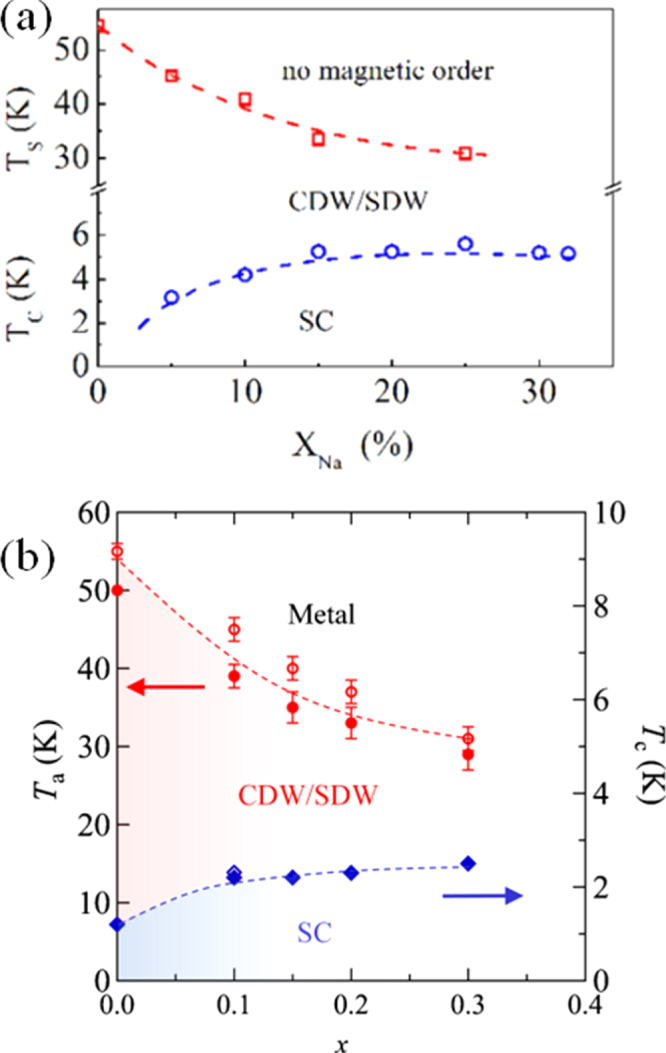
Phase diagram of (a) Ba_1–*x*_Na_*x*_Ti_2_Sb_2_O (taken from [274]) and (b) BaTi_2_(Sb_1−*x*_Sn_*x*_)_2_O (taken from [33]). Reprinted with permission. Copyright 2014 for [33] by the Physical Society of Japan and 2012 for [274] by the American Chemical Society.

It is also possible to control physical properties by aliovalent anion Sb substitution. We have prepared BaTi_2_(Sb_1–*x*_Sn_*x*_)_2_O for *x* ≤ 2.5 and obtained the electronic phase diagram as a function of *x* as shown in figure 32(b) [33]. A qualitative resemblance is seen between phase diagrams of hole doped systems, BaTi_2_(Sb_1–*x*_Sn_*x*_)_2_O and (Ba_1–*x*_A_*x*_)Ti_2_Sb_2_O (A = alkali metals). The saturated behavior of *T*_c_ in the highly doped regime may be related to the robustness of the CDW phase. From quantitative point of view, however, BaT_2_(Sb_1–*x*_Sn_*x*_)_2_O differs from (Ba_1–*x*_Na_*x*_)Ti_2_Sb_2_O. The maximum *T*_c_ in BaTi_2_(Sb_1–*x*_Sn_*x*_)_2_O is 2.5 K at *x* = 0.3, which is remarkably smaller than *T*_c_ = 5.5 K in (Ba_1–*x*_Na_*x*_)Ti_2_Sb_2_O at *x* ∼ 0.33. The reduced *T*_c_ in the former system is attributed to the greater chemical disorder induced by the *Pn*-site substitution and also to the less pnictogen in the Sb/Sn atoms in comparison with Ba/Na atoms.

#### 3.2.5. Isovalent anion substitution

The comparison between BaTi_2_As_2_O (*T*_DW_ = 200 K, *T*_c_ = 0 K) and BaTi_2_Sb_2_O (*T*_DW_ = 50 K, *T*_c_ = 1 K) led us to prepare the isovalent anion substitution system BaTi_2_Bi_2_O so as to destabilize the CDW state and stabilize the superconducting state. It is shown from the structural refinement (figure 28(b)), BaTi_2_Bi_2_O, the first oxybismuthide in this family, is isostructural with BaTi_2_Sb_2_O [31]. The magnetic susceptibility and electric resistivity indeed showed no anomaly indicative of CDW transition, meaning that hole doping is less effective in terms of destabilizing the CDW state than the isovalent anionic substitution. As a result, the enhanced *T*_c_ of 4.6 K is successfully observed in BaTi_2_Bi_2_O. The lattice parameters of BaTi_2_Bi_2_O (*a* = 4.12316(4) Å, *c* = 8.3447(1) Å) are not so different from those of BaTi_2_Sb_2_O, indicating that the lattice expansion is not the primary factor to destabilize the CDW state. Compared with Sb, Bi is less electronegative and provides more covalent Ti–*Pn* bonding. Furthermore, the Bi 6*p* orbital is more diffuse than the Sb 5*p* orbital. Therefore, in BaTi_2_Bi_2_O the Bi 6*p* orbital possibly contributes more to the density of states at the Fermi surface than the Sb 5*p* orbital in BaTi_2_Sb_2_O. It results in weaker nesting of the Fermi surface for BaTi_2_Bi_2_O and suppressed CDW instability.

**Figure 33.**
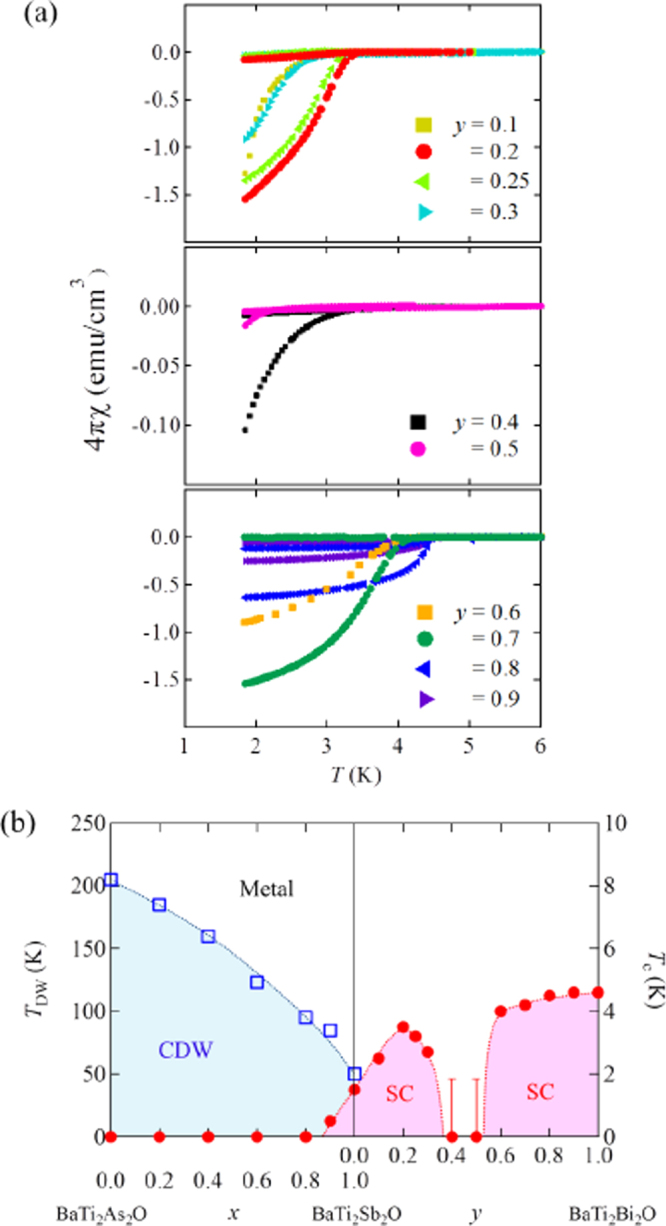
(a) Low-temperature magnetic susceptibility for BaTi_2_(Sb_1–*y*_Bi_*y*_)_2_O. (b) The electronic phase diagram of BaTi_2_(As_1–*x*_Sb_*x*_)_2_O and BaTi_2_(Sb_1–*y*_Bi_*y*_)_2_O, demonstrating the presence of two superconducting phases. Reprinted with permission from [32]. Copyright 2013 by the Physical Society of Japan.

The possession of three compounds BaTi_2_*Pn*_2_O (*Pn*=As, Sb, Bi) allows us to prepare isovalent anionic solid solutions, BaTi_2_(As_1–*x*_Sb_*x*_)_2_O (0 ≤ *x* ≤ 1) and BaTi_2_(Sb_1–*y*_Bi_*y*_)_2_O (0 ≤ *y* ≤ 1) [32]. Despite the Vegard law behavior of the unit cell parameters in both solid solutions, it was unexpectedly observed a novel electronic phase diagram as summarized in figure 33(b), in marked contrast to that of the aliovalent substitution system (figure 32(a)). The gradual destabilization of the CDW state is seen going from *x* = 0 to *x* = 1 in BaTi_2_(As_1–*x*_Sb_*x*_)_2_, whereas superconductivity appears at *T*_c_ = 0.5 K for *x* = 0.9. The *T*_c_ increases with increasing *y* (Bi), takes a maximum value of 3.5 K for *y* = 0.2 and further Bi substitution lead to a decrease in *T*_c_. For *y* = 0.4 and 0.5, no superconductivity is seen down to the lowest temperature measured (1.85 K).

Most remarkably, superconductivity revives at *y* = 0.6, with an increased *T*_c_ toward *y* = 1 (4.6 K). The presence of the second superconducting state for 0.6 ≤ *y* ≤ 1 is quite unusual and implies a novel mechanism behind the superconductivity. It is theoretically shown that a multiband structure involving three Ti 3*d* orbitals (*d*_*xy*_, *d*_*z*2_, and *d*_*x*2–*y*2_) is present at the Fermi surface [290, 294]. It is thus likely that the second superconducting phase is different in nature from the first superconducting phase. Recently, a two-dome structure in *T*_c_ has also been reported in the iron pnictide superconductor LaFeAs(O_1–*x*_H_*x*_) [11], where the multiband structure comprising of Fe 3*d* orbitals is proposed. It should be noted that the two-dome structure in LaFeAs(O_1–*x*_H_*x*_) is induced by the aliovalent substitution and the two superconducting regions are not separated. Hence, BaTi_2_*Pn*_2_O might give a better opportunity to understand the nature of multiband Fermi surface.

#### 3.2.6. Interlayer interactions

In BaTi_2_*Pn*_2_O, the CDW state becomes destabilized as *Pn* = As (*T*_DW_ = 200 K) → Sb (*T*_DW_ = 50 K) → Bi (*T*_DW_ = 0 K). The magnetic susceptibility and electric resistivity of (SrF)_2_Ti_2_Bi_2_O (figure 29(f)) exhibit no signature of CDW transition [76]. However, zero-resistivity and diamagnetic signal are absent in (SrF)_2_Ti_2_Bi_2_O in spite of the fact that the in-plane lattice parameters are similar to each other. Since both (SrF)_2_Ti_2_Bi_2_O and BaTi_2_Bi_2_O contain the [Ti_2_Bi_2_O]^2–^ unit, it is possibly essential to consider the role of the *A*^2+^ unit, or the interlayer coupling in order to explain the difference in their superconducting properties. The (SrF)_2_^2+^ unit is bulky with metal halide double layers, which provides an elongated interlayer distance of 10.685 Å, as compared with 8.345 Å in BaTi_2_Bi_2_O. Hence, the interlayer interaction, at least to a certain extent, is a key component for the appearance of superconductivity. Indeed, the application of external pressure on Ba_1–*x*_Na_*x*_Ti_2_Sb_2_O results in the increase in *T*_c_ [286].

#### 3.2.7. Mixed anion compounds

**Figure 34.**
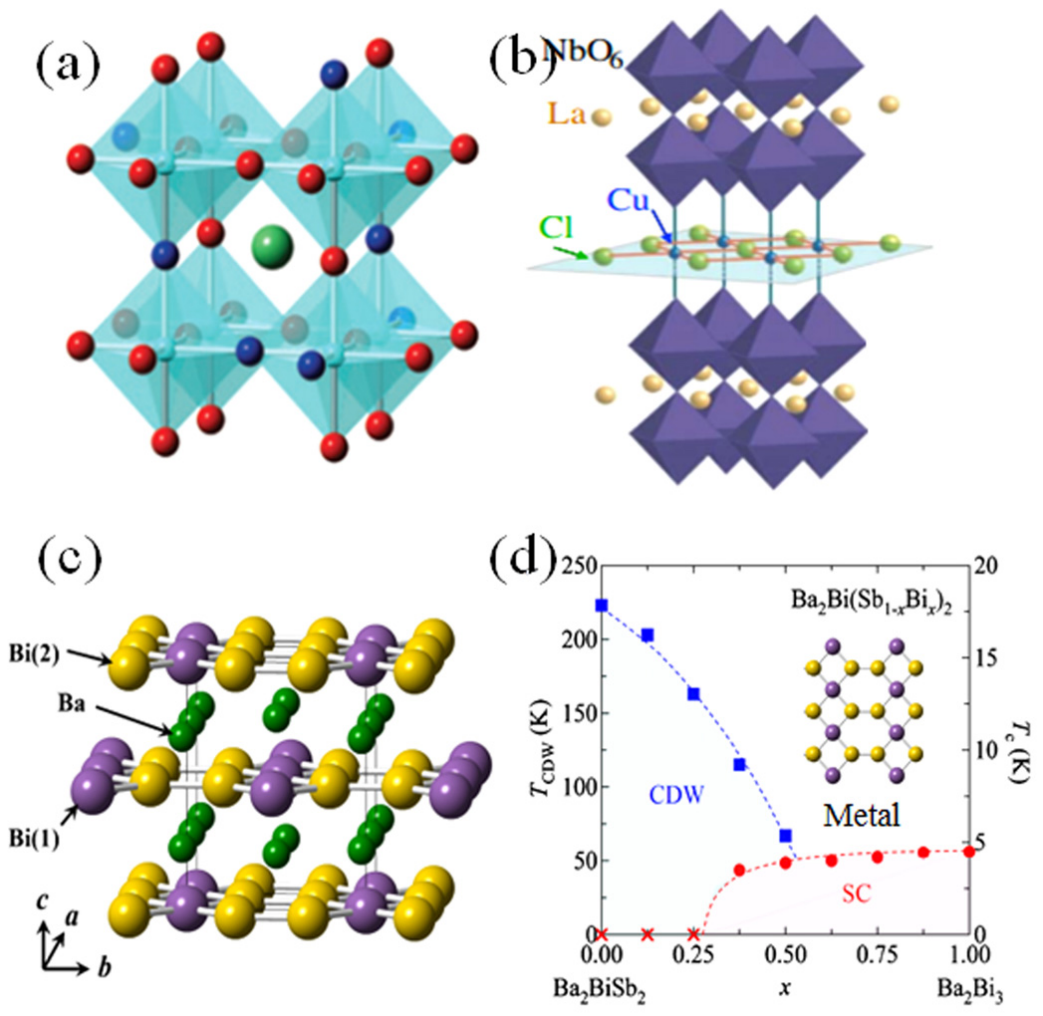
Crystal structure of (a) BaTi(O,H)_3_, (b) (CuCl)LaNbO_7_ and (c) BaBiSb_2_. (d) The phase diagram of Ba_2_Bi(Sb_1–_xBi_*x*_)_2_. Reprinted with permission from [54]. Copyright 2014 by the Physical Society of Japan.

Most functional materials explored by us are single-anion compounds such as oxides, sulfides, chlorides, and bromides. Hence, exploring materials with mixed anion configurations around a transition metal would be promising toward realizing new or improved functional properties including high *T*_c_ superconductivity. For example, oxyhydride cubic perovskite BaTiO_3–*x*_H_*x*_ (*x* ∼ 0.6) with TiO_6–*n*_H_*n*_ (*n* = 0, 1, 2) octahedral coordination (figure 34(a)), obtained by a topochemical reducing reaction using CaH_2_ [268], shows a novel hydride exchangeability with hydrogen gas at moderate temperature [110]. Electron doping to Ti 3*d t*_2g_ band by hydride reduction makes *AE*TiO_3–*x*_H_*x*_ (*AE* = alkaline earth metal) metallic, with high conductivity of 10^2^–10^4^ S cm^−1^, although superconductivity is absent [95]. SrCrO_2_H with CrO_4_H_2_ octahedra, directly prepared from high temperature and high pressure reaction, also adopts the cubic perovskite structure and exhibits the highest magnetic transition temperature among chromium oxides [96]. An oxychloride layered perovskite (CuCl)LaNb_2_O_7_ with Cu^2+^ (*S* = 1/2) in CuO_2_Cl_4_ octahedral coordination (figure 34(b)), prepared by a topochemical ion-exchange reaction of RbLaNb_2_O_7_ [295], shows spin–liquid behavior with a finite gap in the excitation spectrum, due to quantum fluctuations enhanced by two-dimensional structure [296]. Ba_2_BiSb_2_, a hypervalent compound with a unique ‘square-honeycomb’ lattice (figure 34(c)) exhibits a CDW transition [54]. Isovalent anion substitution by Bi (i.e., Ba_2_Bi(Sb_1–x_Bi_*x*_)_2_ leads to the destabilization of the CDW phase and superconductivity with the maximum *T*_c_ of 4.4 K for Ba_2_Bi_3_ (*x* = 1) as shown in figure 34(d).

#### 3.2.8. Conclusions

We have demonstrated recent studies on layered titanium compounds *A*Ti_2_*Pn*_2_O (*Pn* = As, Sb, Bi) with Ti^3+^ in a square lattice, where Ti is coordinately octahedrally with four *Pn* and two O atoms. A newly synthesized BaTi_2_Sb_2_O shows superconductivity at *T*_c_ = 1.2 K. The superconducting phase is of fully gapped BCS-type and is competing/coexisting with a CDW phase with *T*_DW_ of 1.2 K. The aliovalent cation/anion substitution leads to the stabilization of the superconducting state while the CDW state is destabilized only partially. The isovalent anion solid solution, BaTi_2_(As_1–*x*_Sb_*x*_)_2_O, shows a conventional phase diagram with the superconducting phase competing with the CDW phase. However, another isovalent anion solid solution, BaTi_2_(Sb_1–*y*_Bi_*y*_)_2_O, reveals the appearance of a second superconducting state for 0.6 < *y*. The presence of the two superconducting phases strongly indicates the multi-orbital contribution to superconductivity, as also seen in iron arsenic superconductors. The nature of the second superconducting phase is not clear. This phase is possibly competing with other (presently unseen) phases. Further experiments as well as theories are necessary. It is important to note that superconductivity is only observed for *A* = Ba. Further exploratory studies may find superconducting materials with different blocking layers. Finally, we believe that mixed anionic materials are a fruitful playground for novel functional properties including high *T*_c_ superconductivity.

### 3.3. Intercalation compounds with layered and cage-like structures

High-*T*_c_ superconductors recently developed have layered or cage-like structures, and include cuprates [263], iron pnictides [4], MgB_2_ [196], alkali metal doped fullerides [297], and so on. They have intercalated structures composed of charged (doped) layers or frameworks coupled with charge reservoirs in the interlayer or cage-like space. In this study, we will also explore new superconductors, focusing on layered and cage-like structures with covalent networks, and doped electrons via intercalation. Layer structured metal nitride halides, and alloys with clathrate related structures have been developed; electrons are doped by means of intercalation using the interstices between the layers and the cages.

#### 3.3.1. Intercalation compounds of metal nitride halides

**Figure 35.**
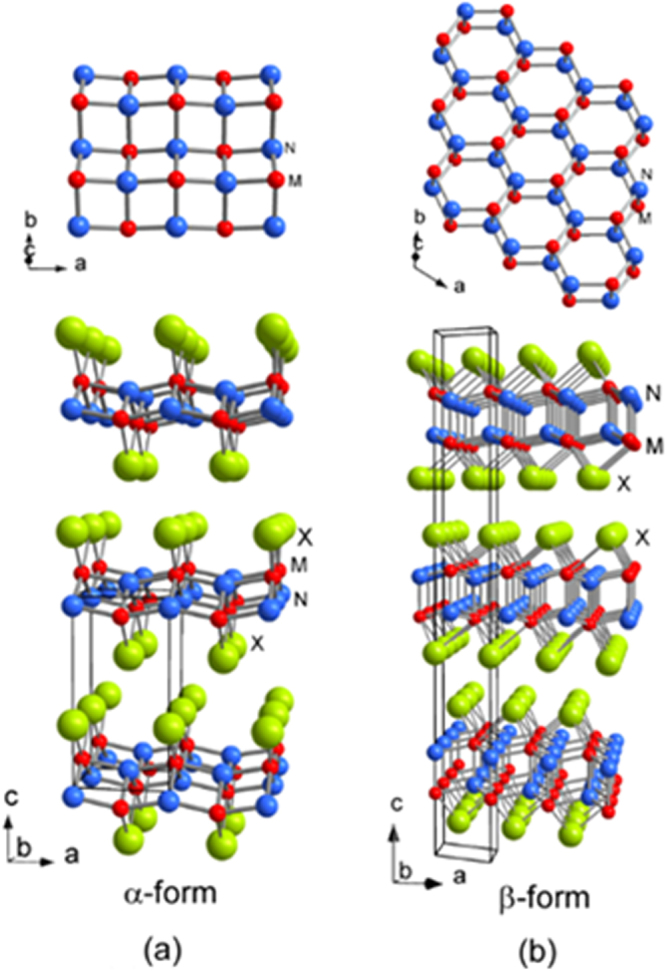
Schematic crystal structures of (a) the *α*- and (b) the *β*-forms of *M*N*X* (*M* = Ti, Zr, Hf; *X* = Cl, Br, I): small red balls, *M*; blue balls, N; and large green balls, *X*. The lower part of the illustration shows the views along the *b*-axes, and the upper part shows the two-dimensional nitride layers of each form. Reproduced from [299] with permission of The Royal Society of Chemistry.

There are two kinds of layered polymorphs in metal nitride halides *M*N*X* (*M* = Ti, Zr, Hf; *X* = Cl, Br, I), *α*- and *β*-forms with the FOCl and the SmSI structures, respectively [298, 299]. The *α*-form layered polymorph has an orthogonal *M*N layer network separated by halogen layers as shown in figure 35(a). The *β*-form consists of double honeycomb-like *M*N layers sandwiched between close-packed halogen layers as shown in figure 35(b). Both polymorphs are band semiconductors with gaps larger than 2.4–4 eV. We have already reported that high-*T*_c_ superconductivity is obtained in *β*-HfNCl upon electron doping by intercalation of alkali metals. The highest *T*_c_ was observed in the lithium and tetrahydrofuran (THF) cointercalated compound Li_0.48_(THF)_y_HfNCl at 25.5 K [300]. The Zr homologue Li_x_ZrNCl also exhibits superconductivity at *T*_*c*_ ∼ 14 K [301]. The electron doped *M*NCl (*M* = Zr, Hf) shows unconventional superconductivity [302, 303]; the electron carrier concentration is very low, but the electron–phonon coupling constant observed (*λ*_e–ph_ << 1) is too small to explain the relatively high *T*_c_ values [304]. Unexpectedly large gap ratios have been observed by specific heat (2*Δ*/*k*_B_*T*_c_ = 6.5) [305] and tunneling spectroscopy (2*Δ*/*k*_B_*T*_c_ = 7–10) [306] measurements. The small isotope effect is also unconventional [307]. Since these superconductors do not contain any magnetic ions, a magnetic pairing mechanism is excluded. Charge and spin fluctuations have been proposed as the possible candidates for the pairing mechanism [308–310].

The *β*-form layered compounds have been highly electron-doped by using liquid ammonia solutions of alkaline earth as well as rare-earth metals. TiNCl with the *α*-form layered structure has been changed into superconductors by electron doping with alkali metals and organic bases such as pyridine and alkylene diamines.

##### 3.3.1.1. Intercalation of alkaline-earth and rare-earth metals in *β*-HfNCl and *β*-ZrNCl

**Figure 36.**
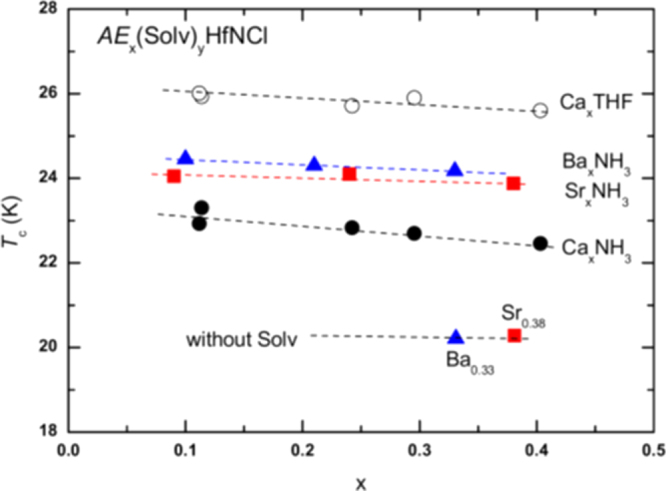
*T*_*c*_ of the as-prepared *AE*_*x*_(NH_3_)_*y*_HfNCl (*AE* = Ca (●), Sr (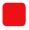), Ba (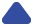)) as a function of concentration *x*. *T*_*c*_ values of the evacuated *AE*_*x*_HfNCl (*AE* = Sr, Ba) and the THF-cointercalated Ca_*x*_(THF)_*y*_HfNCl are compared in the same figure. Reprinted with permission from [59]. Copyright 2013 by IOP Publishing.

In alkali metal (*A*) intercalated compounds, *A* atoms occupy the octahedral or trigonal prism sites between chlorine layers of *β*-*M*NCl [58, 311, 312]. The largest metal concentration attained is expected to be *x* = 0.5 for *A*_x_*M*NCl (*M* = Zr, Hf). We are interested in the electron doping to a much higher concentration using multivalent metals. Alkaline earth (*AE* = Ca, Sr, Ba) [59] and rare earth metals (*RE* = Eu, Yb) [60] have been successfully introduced into the interlayer space of parent materials by using liquid ammonia solutions, which make it possible to study the effect of high doping concentration and additional magnetic spin, respectively. The dependence of the metal doping concentration *x* of *AE*_x_(*Solv*)_y_HfNCl (*AE* = Ca, Sr, Ba; *Solv* = NH_3_, THF) onto the *T*_c_ is shown in figure 36 [59]. Note that the *T*_c_ is hardly influenced by the doping concentration *x* up to *x* ∼ 0.4, corresponding to *x* ∼ 0.8 for monovalent alkali metal intercalated compounds *A*_*x*_(*Solv*)_*y*_HfNCl. The superconductors are not yet over-doped. The *T*_c_ decreases in the following order of the increasing basal spacing (*d*) with intercalation of Sr, Ba (*d* = ~10 Å) > Ca_*x*_(NH_3_)_*y*_, Ba_*x*_(NH_3_)_*y*_, Sr_*x*_(NH_3_)_*y*_ (*d* = 12 Å) > Ca_*x*_(THF)_*y*_ (*d* = 15 Å), although a minimum level of doping is certainly necessary for the superconductivity. The as-prepared compounds are cointercalated with ammonia used as a solvent, which can be replaced with THF. With varying electron-doping concentrations and interlayer spacings, the highest *T*_c_ of 26.0 K was obtained for the Ca and THF cointercalated compound Ca_0.11_(THF)_*y*_HfNCl, a new record of high *T*_c_ in the electron-doped metal nitride chloride system [59].

**Figure 37.**
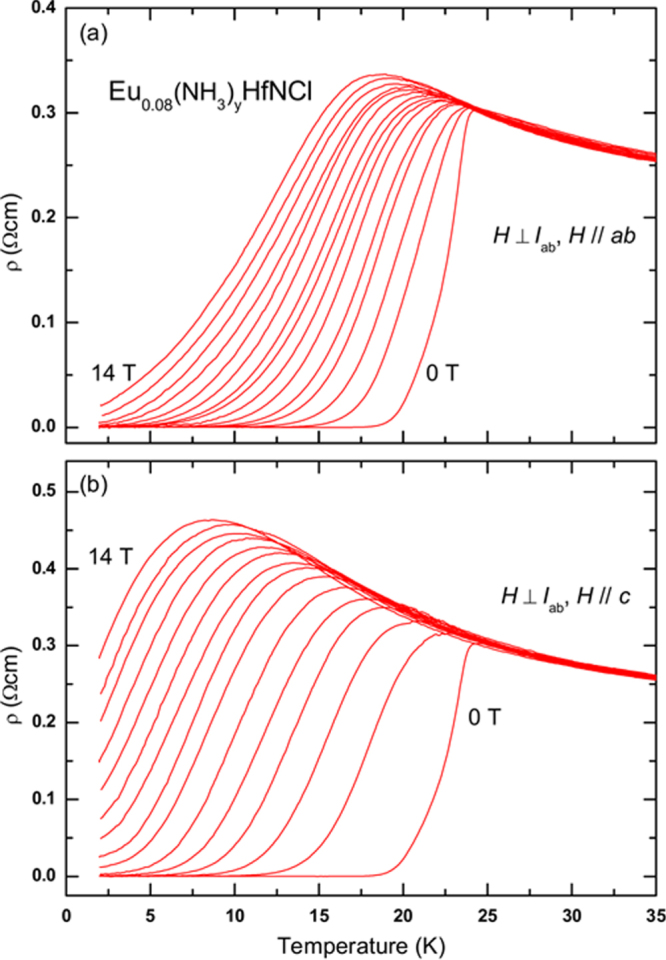
Temperature dependences of the resistivity of Eu_0.08_(NH_3_)_*y*_HfNCl in various magnetic fields for (a) *Hǁab* (*H*⊥*I*_*ab*_) and (b) *Hǁc* (*H* ⊥ *I*_*ab*_). The applied magnetic field was increased from 0 to 14 T with an interval of 1 T. Reprinted with permission from [60]. Copyright 2013 by IOP Publishing.

Multivalent rare-earth metals (Eu and Yb) can be solved in liquid ammonia, and intercalated into *β*-*M*NCl (*M* = Zr, Hf) from the solutions [60]. The *T*_c_ values of the ammonia cointercalated compounds *RE*_*x*_(NH_3_)_*y*_HfNCl (*RE* = Eu, Yb) are comparable with those of *AE*_*x*_(NH_3_)_*y*_HfNCl (figure 36); 24.1 and 23.0 K for Eu_0.13_(NH_3_)_*y*_ and Yb_0.11_(NH_3_)_*y*_, respectively. The temperature dependence of the magnetic susceptibility of *RE*_*x*_(NH_3_)_*y*_*M*NCl measured in a temperature range of 100–300 K suggests that the *RE* metals exist as the paramagnetic ions Eu^+2^ and Yb^+3^. The anisotropic magnetoresistance measurement has evidenced that the paramagnetism of Eu^2+^ and Yb^3+^ can coexist with the superconductivity even under high magnetic fields up to 14 T, as shown in figure 37 for Eu_0.08_(NH_3_)_*y*_HfNCl. The anisotropic upper critical fields (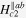 and 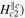 were determined with the magnetic field parallel to the *ab* plane and *c* axis, respectively, on a uniaxially oriented pellet sample. The anisotropy parameter *γ* = (d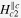/d*T*)/(d/d*T*) = 4.1, comparable to those of other electron-doped *β*-*M*NCl superconductors, 3.7 for Li_0.48_(THF)_*y*_HfNCl [313] and 4.5 for ZrNCl_0.7_ [314].

**Figure 38.**
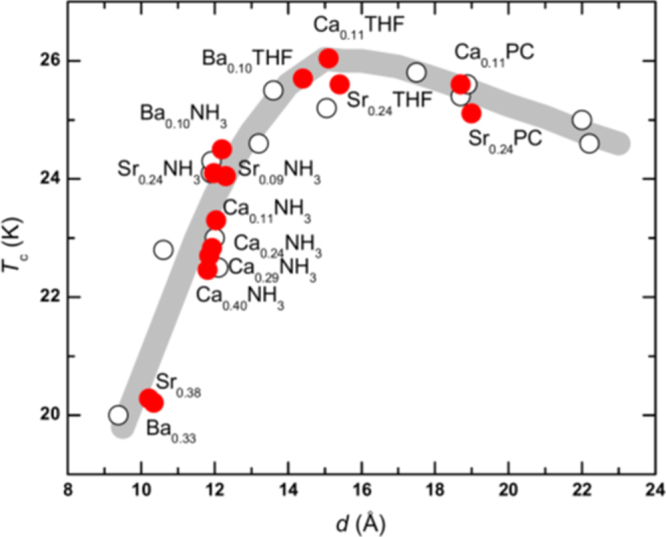
*T*_*c*_ dependence on the basal spacing *d* of *AE*_x_(*Solv*)_y_HfNCl (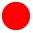), and from alkali metal and rare earth metal intercalated compounds (○). Reprinted with permission from [59]. Copyright 2013 by IOP Publishing.

All the *T*_c_ values of the electron doped *β*-HfNCl so far determined can fit on a single curved line as a function of the basal spacing (*d*) as shown in figure 38 [59], suggesting that the alkali, alkaline-earth and rare-earth metals act as similar electron dopants, and the *T*_c_ is hardly dependent on the doping concentration. A very similar trend was also observed in the electron doped *β*-ZrNCl [315]. It should be noted that the *T*_c_ increases with increasing *d* upon cointercalation of NH_3_ and THF, and then decreases gradually with the further increase of *d* upon cointercalation of PC.

##### 3.3.1.2. Intercalation compounds of TiNCl with alkali metals

In a previous study we prepared the alkali metal intercalation compounds of the *α*-form structured HfNBr, *A*_*x*_(THF)_*y*_HfNBr (*A* = Li, Na) [316]. The intercalation was successful, and the color of the layered crystals changed from pale yellow to black. However, the resulting compounds were found to not be superconductors, but insulators with resistivity >10^7^ *Ω*cm. This is quite a contrast to the fact that the electron doped *β*-HfNBr shows high-*T*_c_ superconductivity, like *β*-HfNCl. Later we have used *α*-form layered crystals TiNCl, which are prepared by the ammonolysis of TiCl_4_ at elevated temperatures, followed by purification via chemical transport [317]. TiNCl can be intercalated with alkali metals as well as neutral organic molecules such as pyridine and diamines. The intercalation compounds become superconductors. The reason for the quite different behaviors of the two kinds of *α*-form crystals TiNCl and HfNBr is not clear.

(i) *Alkali metal intercalation in TiNCl*.

**Figure 39.**
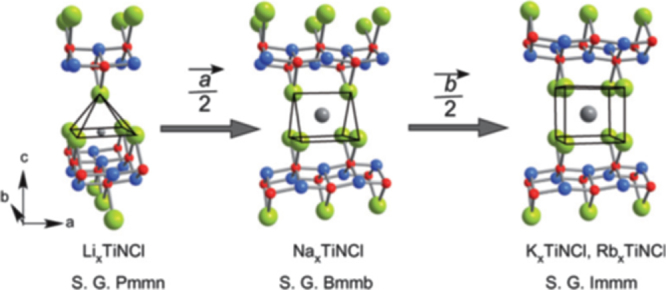
Schematic structures of the TiNCl polytypes with intercalated cations between the layers. Reproduced from [317] with permission of The Royal Society of Chemistry.

In the first attempt of the intercalation, TiNCl was subjected to reaction with various kinds of metal azides *A*N_3_ (*A* = Li, Na, K, Rb) at elevated temperatures under vacuum [317]. The azides are thermally decomposed to metal and nitrogen, and the resulting metal is intercalated into the interlayer space between chloride layers. Part of the alkali metal is also used to extract or deintercalate chloride ions from the interlayer space, forming a metal chloride

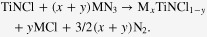
The metal intercalated compounds show superconductivity with *T*_c_ ∼ 16 K, although the superconducting volume fractions determined from the diamagnetic expulsion were found to be as low as 5–30%. The Rietveld analysis of the x-ray powder diffraction data revealed that the TiNCl crystalline layers are mutually shifted to accommodate the metal atoms between the chloride layers as shown in figure 39. The space groups of the resulting new polytypes are *Bmmb* for Na, and *Immm* for K or Rb intercalated compounds. The space group of Li_x_TiNCl is unchanged from *Pmmn* of the pristine crystal.

**Figure 40.**
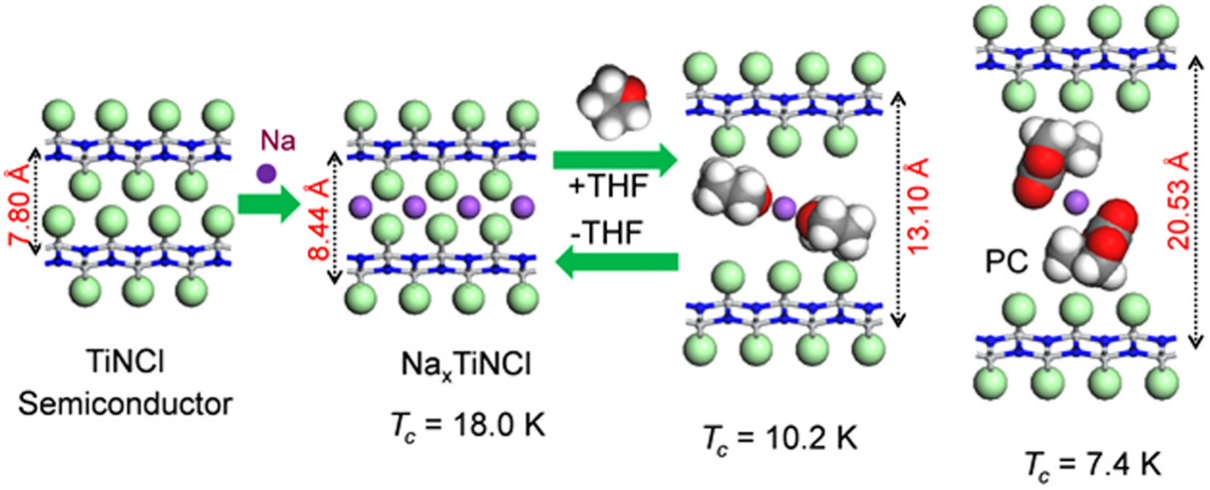
Schematic illustration showing the expansion of the basal spacing of TiNCl upon intercalation of Na and cointercalation of solvent molecules. Reprinted from [303] with permission from Elsevier.

**Figure 41.**
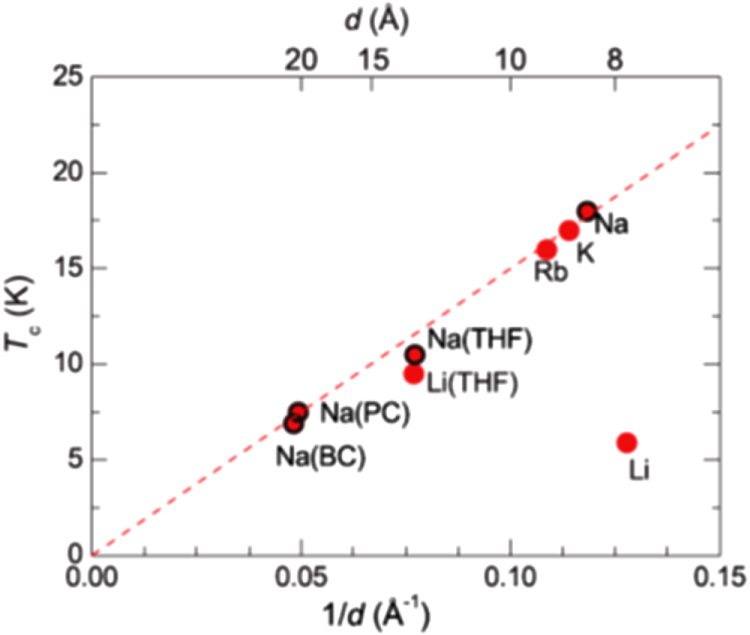
*T*_*c*_ versus 1/*d* for *Ax*TiNCl (*A* = Li, Na, K, Rb) superconductors with and without cointercalation [56]. Reprinted with permission from [56]. Copyright 2012 by the American Physical Society.

A mild intercalation reaction using metal naphthalene solutions in THF can produce superconductors with a much higher volume fraction >60% [56]. The as-prepared compound is co-intercalated with THF. As shown in figure 40, the basal spacing (*d*) of the compounds varies depending on the cointercalation conditions. Na_0.16_(THF)_*y*_TiNCl has a basal spacing of 13.10 Å (*T*_c_ = 10.2 K), which decreases to 8.44 Å (*T*_c_ = 18.0 K) upon removal of THF by prolonged evacuation at 90 °C. The THF molecules can be replaced with larger size solvent molecules PC (propylene carbonate); the basal spacing increases to 20.53 Å with decreasing *T*_c_ to 7.4 K. Figure 41 shows the *T*_c_ values as a function of 1*/d* for alkali metals and solvent cointercalated compounds. The *T*_c_ decreases with the increase of the basal spacing (*d*). The data fit on a linear line passing through the origin, suggesting the importance of the Coulomb interlayer coupling in the pairing mechanism in this system. It should also be noted that the *T*_c_ of non-cointercalated compounds *A*_*x*_TiNCl (*A* = Na, K, Rb) also fit on this line except Li_0.13_TiNCl. The as-prepared sample Li_0.13_(THF)_*y*_TiNCl has a basal spacing of 13.1 Å similar to that of Na_0.16_(THF)_*y*_TiNCl, and a *T*_c_ = 10.2 K. The non-cointercalated compound Li_0.13_TiNCl was obtained by evacuation at 150 °C, which has the smallest basal spacing of 7.8 Å, the same as that of the pristine TiNCl. Unexpectedly, *T*_c_ was found to be ∼6.0 K, much lower than the value expected for the small basal spacing of figure 41. Li ions are small enough in size to penetrate into chlorine layers, forming double LiCl layers between [TiN]_2_ layers, [TiN]_2_(Li_0.13_Cl)(ClLi_0.13_)[TiN]_2_, in which Li ions are located close to TiN superconducting layers in parallel with Cl atoms. On the other hand, in the THF cointercalated compound, Li ions can be coordinated with THF molecules between chlorine layers [ClTi_2_N_2_Cl]Li_0.26_(THF)_*y*_[ClTi_2_N_2_Cl]. The low *T*_c_ of Li_0.13_TiNCl against the small *d* suggests that the location of positive centers may also influence the Coulomb interlayer coupling for superconductivity. The linear relation shown in figure 41 appears to be applied to the structure where the positive centers are located between the chlorine layers as shown in figure 40 for the Na_0.16_(THF)_*y*_ cointercalated compound. Another systematic study on the relation between the basal spacing and *T*_c_ has been performed on TiNBr, and again a similar linear relation was found [57].

(ii) *Anisotropic superconducting properties*.

**Figure 42.**
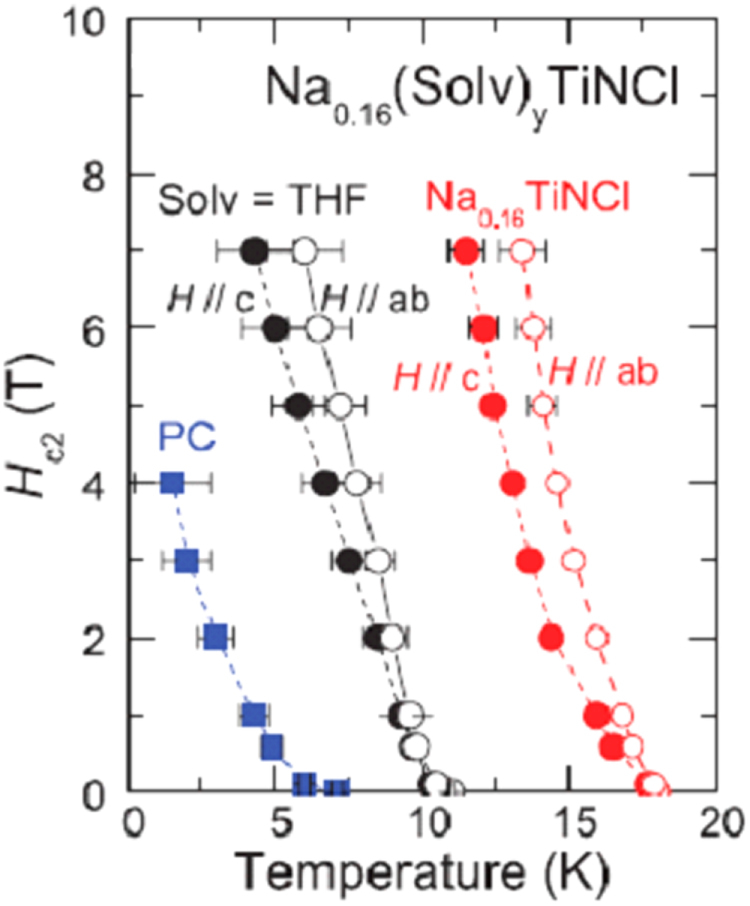
*H*_*c*2_–*T* phase diagram of Na-intercalated compounds with and without cointercalation. Reprinted with permission from [56]. Copyright 2012 by the American Physical Society.

The anisotropic magnetic susceptibility of the layered superconductor Na_0.16_(THF)_*y*_TiNCl was measured on the highly oriented pellet sample with the magnetic field parallel and perpendicular to the *c* and *ab* plane [56]. Figure 42 shows the anisotropic *H*_*c*2_ thus determined as a function of temperature. The anisotropy parameter was calculated to be *γ* = 1.5 and 1.2 for Na_*x*_(THF)_*y*_TiNCl and Na_*x*_TiNCl, respectively. Note that the TiNCl superconductors exhibit rather isotropic or 3D character. The expansion of the basal spacing from 8.44 to 13.10 Å by cointercalation of THF has little effect on the anisotropy. The superconductor derived from TiNBr also shows a similar *γ* value [57]. The characteristic superconducting parameters of the *α*- and the *β*-structured layered nitride superconductors are compared in table 12. The anisotropy parameter *γ* = *ξ*_*ab*_/*ξ*_*c*_ (ratio of the coherence lengths in the *ab* plane and along the *c*-axis) for K_0.21_TiNBr is calculated to be ∼1.3, close to that found in electron-doped TiNCl; Na_0.16_TiNCl (*γ* = 1.2, *T*_c_ = 18.1 K) and Na_0.16_(THF)_*y*_TiNCl (*γ* = 1.5, *T*_c_ = 10.2 K) [56]. In contrast, the *β*-structured nitrides show the anisotropy parameter *γ* as large as 3.7–4.5 [59, 60, 313, 314]. The small anisotropy parameter *γ* appears to be one of the characteristic features of the *α*-structured superconductors. The coherence length along the *c*-axis (*ξ*_*c*_) of *β*-Li_0.48_(THF)_*y*_HfNCl is about 16 Å, comparable with the basal spacing 17.8 Å, i.e., the separation of the superconducting layers. This suggests that the superconducting *β*-form layers may be weakly Josephson coupled. On the other hand, in the *α*-form TiNBr and TiNCl, the *ξ*_*c*_ are more than three times larger than the basal spacing, implying that the nitride layers are more strongly coupled.

**Table 12.** Characteristic superconducting parameters of the *α*- and *β*-structured layered nitride superconductors [57].

Compound	*T*_*c*_ (K)	*d* (Å)	ξ_*ab*_ (Å)	ξ_c_ (Å)	λ_*ab*_ (Å)	*γ*	Reference
*α*-K_0.21_TiNBr	17.2	9.5	53	41	3045	1.3	[57]
*α*-Na_0.16_TiNCl	18.1	8.4	33	28	4746	1.2	[56]
*α*-Na_0.16_(THF)_y_TiNCl	10.2	13.1	55	35	—	1.5	[56]
*β*-ZrNCl_0.7_	13	9.8	71	16	—	4.5	[314]
*β*-Li_0.48_(THF)_y_HfNCl	25.5	18.7	60	16	4630	3.7	[313]
*β*-Eu_0.08_(NH_3_)_y_HfNCl	24.3	11.9	61	15	—	4.1	[60]
*β*-Ca_0.11_(THF)_y_HfNCl	26.0	15.0	47	12	—	4.1	[59]

High-*T*_c_ layered cuprate superconductors have large anisotropy parameters *γ* on *H*_c2_, varying in the range of 3–30 [318]. Iron pnictide superconductors *Ln*FeAs(O, F) (*Ln* = La, Sm, Nd) recently discovered also show a large anisotropy of *γ* = 4–9 [319–323]. The large anisotropy parameters of the layered compounds have been considered to be important to realize high-*T*_c_ superconductivity. The electron-doped *β*-ZrNCl and *β*-HfNCl are also classified into this category. However, the Ba-122 superconductors such as (Ba, K)Fe_2_As_2_ with *T*_c_ = 38 K have been developed, which have a small isotropic parameter *γ* = 1.5–1.9 [231, 319, 324–327]. The layer coupling through intervening (Ba, K) atoms seems to be stronger than those of the 1111 pnictides coupled through metal oxide layers. It is interesting to note that *β*-ZrNCl_0.7_ (table 12), which is electron-doped by a partial deintercalation of chlorine atoms from the interlayer space, has an anisotropy parameter as large as 4.5. In *β*-ZrNCl_0.7_ with *d* = 9.8 Å, the nitride layers should be directly coupled without intervening alkali atoms. Nevertheless, the anisotropy parameter is comparable to, or even larger than, that of the cointercalated compound *β*-Li_0.48_(THF)_*y*_HfNCl. It is evident that the interlayer separation is not a decisive parameter for the anisotropy on *H*_c2_. The small anisotropy on *H*_c2_ and the Coulomb coupling between the superconducting layers should be the relevant nature of the superconductivity of the *α*-form layered nitrides [57]. For more discussion on the superconducting mechanisms and the anisotropy of the two different kinds of layered nitrides, a theoretical study including the electric band structure is required [328].

##### 3.3.1.3. Intercalation compounds of TiNCl with neutral amines

**Figure 43.**
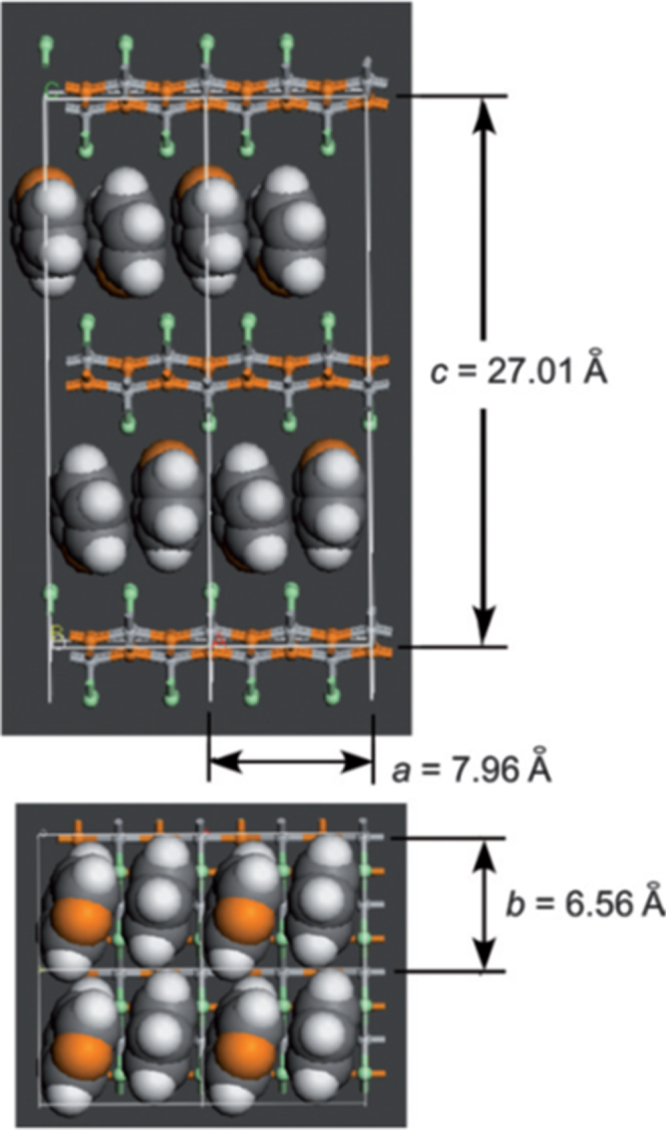
The arrangements of pyridine (Py) molecules in Py_0.25_TiNCl obtained by geometrical optimization. A 2 × 2 × 1 supercell is used; the views along the *b*-axis (top) and along the *c*-axis (bottom). Nitrogen atoms are orange-colored. Reproduced from [317] with permission of The Royal Society of Chemistry.

In the formation of the intercalation compound of *β*-form layered compounds, organic solvent molecules are cointercalated with metal atoms; organic molecules alone cannot be intercalated. In contrast, *α*-form TiNCl can intercalate neutral organic molecules without metal atoms. TiNCl can form an intercalation compound with pyridine from liquid and gas phases, Py_0.25_TiNCl. The basal spacing increases to 13.5 Å with the molecular plane oriented perpendicular to the layers as schematically shown in figure 43 [317]. The compound becomes a superconductor with *T*_c_ = 8.6 K. The *T*_c_ is different from those of the alkali metal intercalated compounds. It is interesting to develop different kinds of organic compounds which can be intercalated to obtain high-*T*_c_ superconductors. Although the doping mechanism is not yet clear in the intercalation compound with pyridine, it would be reasonable to estimate that the lone pair electrons of nitrogen atoms in pyridine may act as electron donors to the TiNCl layers. It was reported that FeOCl isotypic with TiNCl forms an intercalation compound with pyridine, and the electrical conductivity increases by about seven orders due to the charge transfer from the organic Lewis base to the FeOCl layers [329]. In that study, various kinds of aliphatic amines have been intercalated into TiNCl to develop new superconductivity.

**Figure 44.**
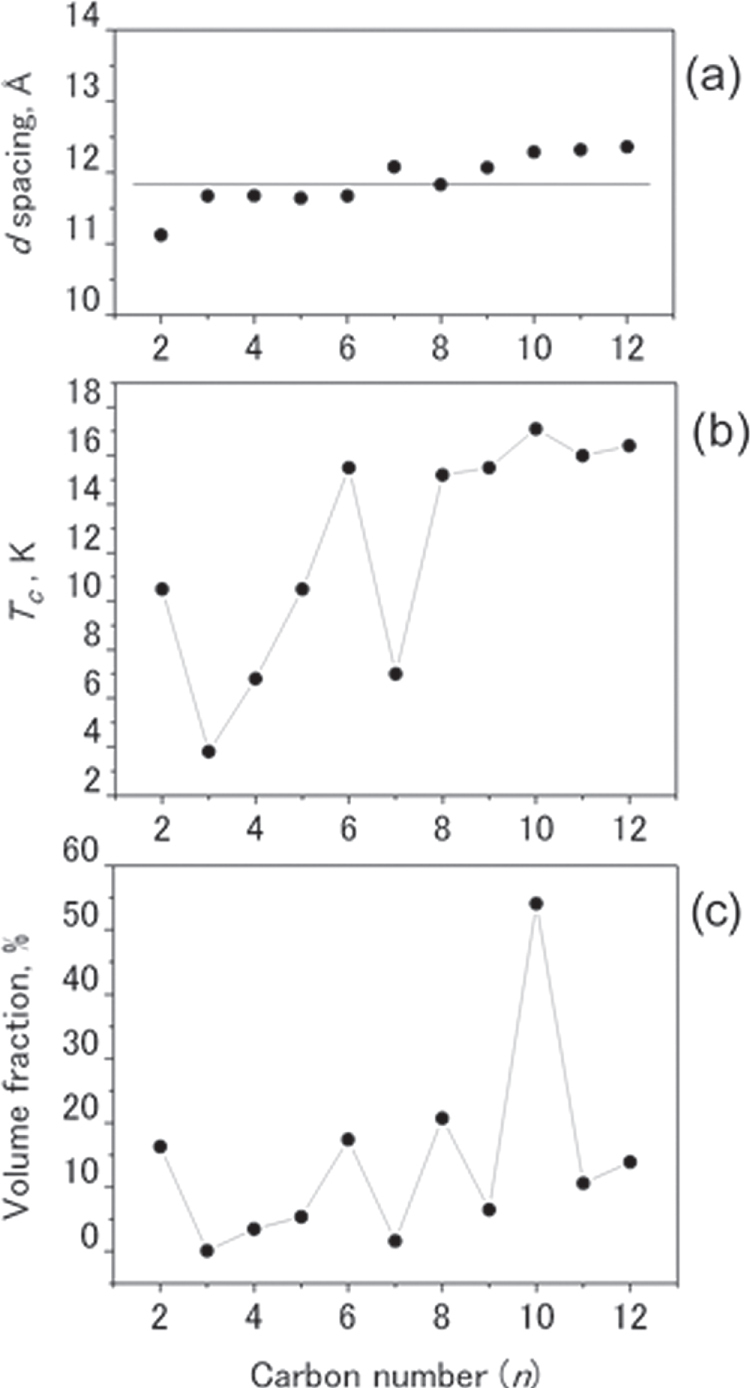
(a) Basal spacing (*d*), (b) superconducting transition temperature (*T*_*c*_) and (c) superconducting volume fraction as a function of the number of carbon atoms (*n*) in the alkylene chains of diamine in the (NH_2_C_*n*_H_2*n*_NH_2_ (2 ≤ *n* ≤ 12))_*x*_-TiNCl compounds. Reproduced from [55] with permission of The Royal Society of Chemistry.

**Figure 45.**
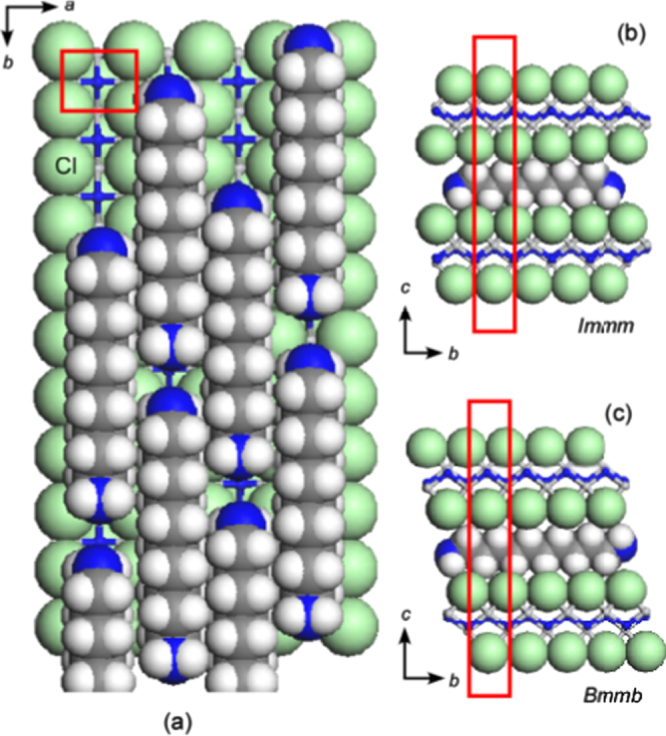
Schematic illustration showing the arrangements of alkylene diamine molecules between TiNCl layers; (a) *ab* projection: the linear alkylene diamine molecules are oriented parallel to the layers and aligned along the *b*-axis, (b) the *bc* projection for the arrangement of nonamethylene diamine (NMDA) molecules in the interlayer space of the TiNCl host structure with the space group *Immm*, and (c) the *bc* projection for the arrangement of decamethylene diamine (DMDA) molecules in the interlayer space of the host structure with the space group *Bmmb*. The unit cells are shown in red color in the projections. Reproduced from [55] with permission of The Royal Society of Chemistry.

*n*-alkyl monoamines (C_*n*_H_2*n*+1_NH_2_, 3 ≤ *n* ≤ 12) can form intercalation compounds with TiNCl, expanding the basal spacing to a value in the range of 12.0 to 37 Å, with the alkyl chains oriented in various ways. All of the compounds with *n*-alkyl monoamines are not superconductors down to 2 K [55]. It is interesting that ethylene diamine (NH_2_CH_2_CH_2_NH_2_) can form a similar intercalation compound with TiNCl with a basal spacing of 11.12 Å, which shows superconductivity with *T*_c_ = 10.5 K [55]. Systematic studies have been done using alkylene diamines with different numbers of carbon atoms, NH_2_C_n_H_2n_NH_2_ (2 ≤ *n* ≤ 12). The results are shown in figure 44 [55]. Most of the diamine intercalation compounds are superconductors. The basal spacings are near 12.5 Å irrespective of the chain length of diamines, suggesting that the alkylene chains are aligned with the molecular axis parallel to the layers, and oriented along the *b*-axis as shown in figure 45. Diamines with an even number of carbon atoms appear to have larger superconducting volume fractions, and the diamines with longer alkylene chains are suitable for higher *T*_c_. The compound with *n* = 10 (decamethylene diamine, DMDA) shows a large volume fraction > 50%, and *T*_c_ = 17.1 K, which is comparable with *T*_c_ = 18.1 K of Na_0.16_TiNCl. Mechanisms for the superconductivity are not clear, and remain open problems for physicists as well as chemists.

#### 3.3.2. Silicon clathrates and related compounds with cage-like structures

Using high pressure and high temperature (HPHT) conditions we have prepared the barium-containing silicon clathrate compound Ba_8_Si_46_, which shows superconductivity with *T*_c_ = 8.0 K [330]. This is the first superconductor with a clathrate structure. The application of HPHT conditions is favorable for synthesizing silicon-rich binary phases such as LaSi_5_, LaSi_10_, BaSi_6_, Ba_24_Si_100_, Ba_8_Si_46_, NaSi_6_, Na_8_Si_46_, and Na_x_Si_136_ [98, 331–334]. The silicon rich compounds are generated under high pressure by obeying Le Chatelier’s principle; the molar volume of the reactant of the system decreases in the product by forming covalent networks and high coordination environments. Electrons are doped from metals into the covalent networks. Most compounds are found to become superconductors. In this study of silicon clathrates and related compounds, ternary systems of ubiquitous (commonly found) elements have been developed using HPHT conditions.

##### 3.3.2.1. Ternary system Ca-Al-Si under HPHT conditions

**Figure 46.**
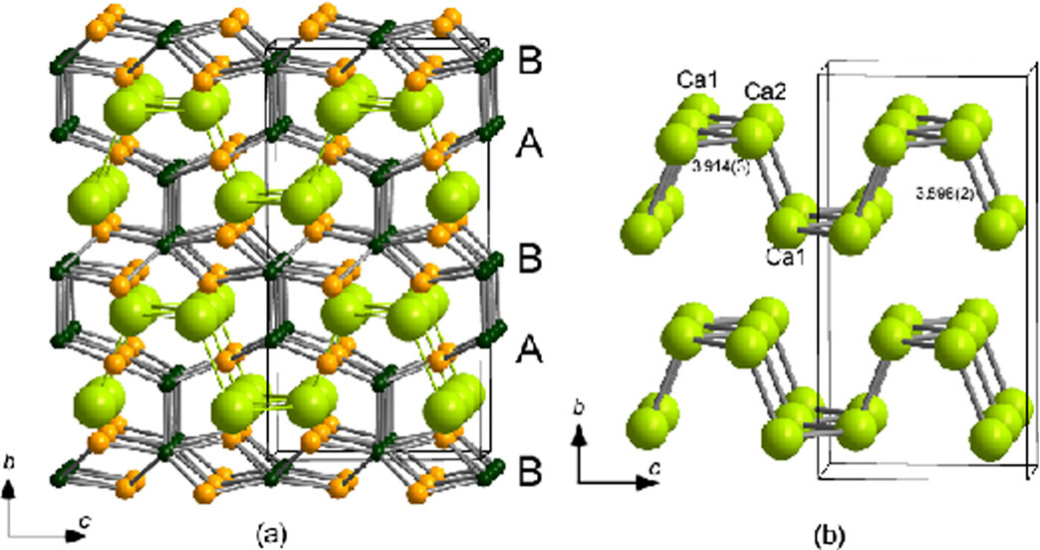
Schematic representation of the crystal structure of (a) Ca_2_Al_3_Si_4_ composed of A [AlSi_2_] and B [Al_2_Si_2_] layers with Ca atoms: small black balls, Si; orange balls, Al; green balls, Ca. (b) [Ca_2_] layer which is isomorphous with black phosphorus. Reprinted from [45] with permission from Elsevier.

Ca–Al–Si ternary metal mixtures were pre-melted using an arc furnace, followed by remelting at 1000–1200 °C using radio frequency induction heating under an Ar atmosphere in an *h*-BN crucible. The cooled ternary mixtures were supplied for the HPHT treatment up to 5–13 GPa and 600–1000 °C by using a Kawai-type multianvil apparatus. A new ternary compound Ca_2_Al_3_Si_4_ was obtained above 650 °C under a pressure of 5 GPa. It crystallizes with the space group *Cmc*2_1_ and the lattice parameters *a* = 5.8846(8), *b* = 14.973(1), and *c* = 7.7966(8) Å [45]. The structure is composed of an aluminum silicide framework [Al_3_Si_4_] and layer structured [Ca_2_] network interpenetrating with each other as shown in figure 46. The [Ca_2_] subnetwork has an isomorphous structure with black phosphorus. The compound shows superconductivity with *T*_c_ of 6.4 K.

**Figure 47.**
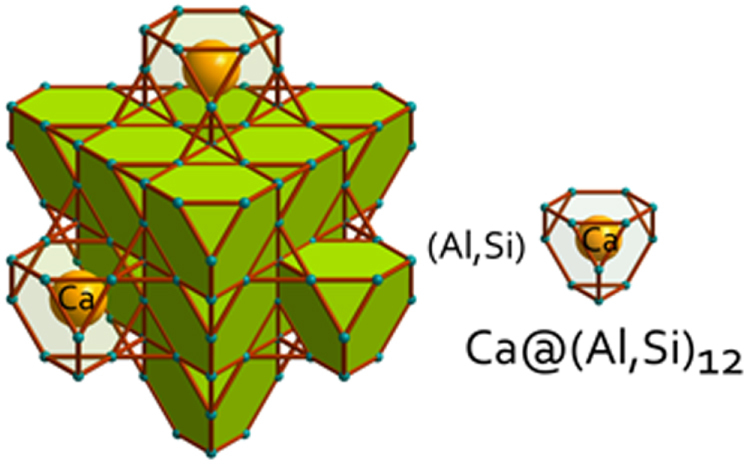
Crystal structure of the Laves phase Ca(Al,Si)_2_ composed of face-sharing truncated Ca@(Al,Si)_12_ tetrahedra. Reprinted with permission from [71]. Copyright 2013 by the American Chemical Society.

Under a higher pressure of 13 GPa at 1000 °C, solid solutions Ca(Al_1*−x*_Si_*x*_)_2_ (0.35 ≤ *x* ≤ 0.75) isomorphous with the cubic Laves phase were obtained [71]. As shown in figure 47 the structure can be regarded as a kind of clathrate compound composed of face-sharing truncated tetrahedral cages with Ca atoms at the center, Ca@(Al,Si)_12_. The compound with a stoichiometric composition CaAlSi shows superconductivity with *T*_c_ of 2.6 K [71]. This is the first superconducting Laves phase compound composed solely of commonly found elements.

##### 3.3.2.2. Ternary system Al–Mg–Si under HPHT conditions

Although the ternary system Al–Mg–Si is an important subject in the development of commercial Al alloys [335], Mg_2_Si with antifluorite structure is the only compound known as bulk phase in the ternary system under ambient pressure. However, various kinds of fine coherent precipitates are formed in the Al matrix during low temperature aging, which are considered to play an important role for the hardening of a commercial Al alloy. It is reasonable to assume that the ternary and binary precipitates found in Al-based alloys are formed under high pressure generated by Al matrix of the alloy. New binary and ternary compounds in the ternary system Al–Mg–Si have been prepared using HPHT conditions, and the structures are determined using single crystals. Some of them become superconductors.

Ternary compounds Mg(Mg_1–*x*_Al_*x*_)Si (0.3 < *x* < 0.8) have been prepared under HPHT conditions of 5 GPa at 800–1100 °C. The single crystal study revealed that the compound (*x* = 0.45) is isomorphous with the anticotunnite, or the TiNiSi structure, and crystallizes with space group *Pnma*, with lattice parameters *a* = 6.9242(2), *b* = 4.1380(1), *c* = 7.9618(2) Å, and *Z* = 4. The compound with *x* > 0.5 shows superconductivity with a *T*_c_ ∼6 K [70]. The compound is a peritectic solid solution associated with other phases such as Mg_9_Si_5_, Al, and Si, depending on cooling protocols in the preparation. The band structure calculation on the composition of MgAlSi suggests that the Al and Mg orbitals mainly contribute to the density of states near the Fermi level, and the substitution of Mg with Al favors superconductivity.

**Figure 48.**
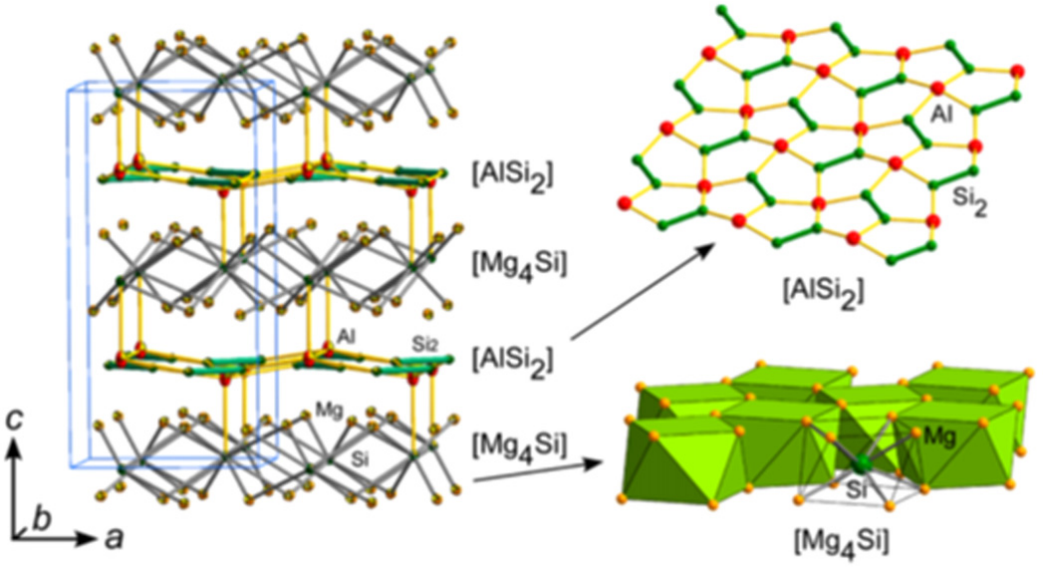
Structure of Mg_4_AlSi_3_ composed of alternate stacking of [Mg_4_Si] and [AlSi_2_] layers. The [AlSi_2_] layers form an almost coplanar Cairo pattern with a short Si–Si bond distance (2.43 Å). The [Mg_4_Si] layers consist of Mg antitetragonal prism polyhedra surrounding Si atoms. Reprinted with permission from [46]. Copyright 2013 by the American Chemical Society.

Two kinds of magnesium-based compounds Mg_9_Si_5_ and Mg_4_AlSi_3_ have been prepared under a similar HPHT condition. Single crystal study revealed that Mg_9_Si_5_ crystallizes in space group *P*6_3_ (no. 173) with the lattice parameters *a* = 12.411(1) Å, *c* = 12.345(1) Å, and *Z* = 6 [46]. The structure can be derived from the high pressure form Mg_2_Si with anticotunnite structure; excess Si atoms of Mg_9_Si_5_ form Si–Si pairs in the prismatic cotunnite columns running along the *c*-axis. Mg_4_AlSi_3_ is obtained by a rapid cooling of a ternary mixture Mg:Al:Si = 1:1:1 from ∼800 °C to room temperature under a pressure of 5 GPa. The compound crystallizes in space group *P4/ncc* (no. 130) with the lattice parameters *a* = 6.7225(5) Å, *c* = 13.5150(9) Å, and *Z* = 4 [46]. As shown in figure 48, the structure consists of an alternate stacking of [AlSi_2_] layers having a Cairo pattern and [Mg_4_Si] antitetragonal prismatic layers. It can be viewed as composed of hexa-Si-capped tetragonal prismatic cages Mg_8_Si_6_ with an Al atom at the center of each cage, Al@Mg_8_Si_6_. The compound shows superconductivity with a transition temperature *T*_c_ = 5.2 K. The formation regions of the two kinds of new magnesium-based compounds have been proposed.

##### 3.3.2.3. An attempt to prepare carbon analogs for silicon clathrate compounds

Silicon and carbon chemistries are often discussed comparatively from the viewpoint of belonging to the same group in the periodic table [336, 337]. It is interesting to prepare metal doped carbon clathrate compounds, in which carbon forms a *sp*^3^ clathrate network-like open diamond framework. A hole-doped diamond was found to become a superconductor [338]. If the synthesis of an electron doped carbon clathrate is realized, it is expected that the carbon *sp*^3^ framework with a high Debye temperature should exhibit high-*T*_c_ superconductivity [339].

We have already obtained 3D carbon frameworks with cages by polymerization of C_60_ crystals under HPHT conditions [340, 341]. An attempt has been made to prepare a Ba doped clathrate-like structure from Ba-doped fulleride, Ba_3_C_60_, using HPHT conditions.

**Figure 49.**
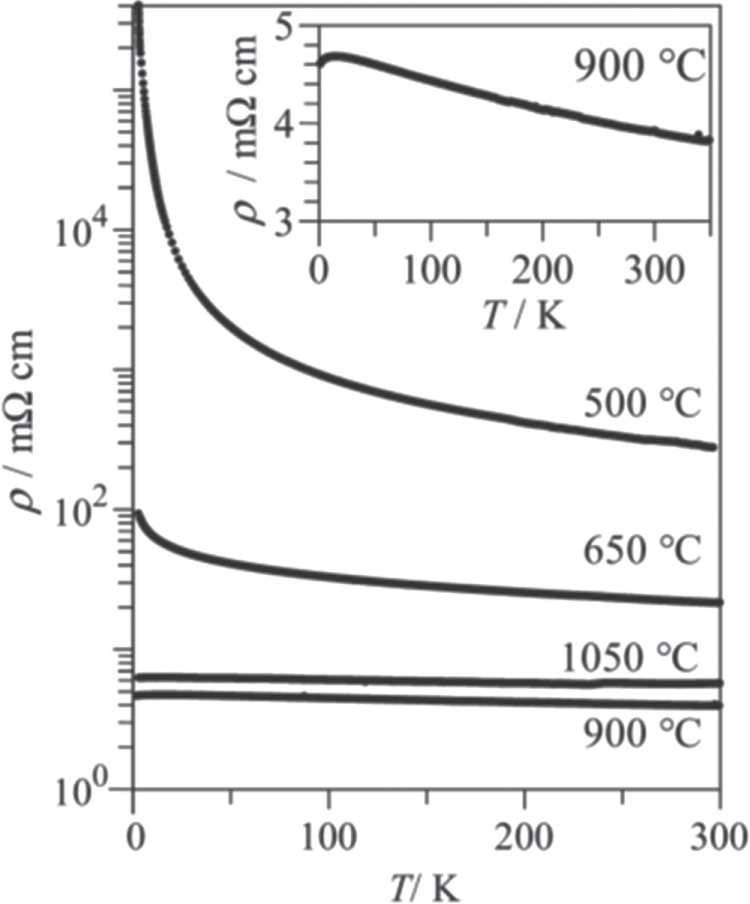
Temperature dependences of the electrical resistivity of Ba_3_C_60_ samples obtained by treatment at various temperatures under 15 GPa. The inset shows the resistivity in an enlarged linear scale for the sample obtained at 900 °C under 15 GPa. Reprinted from [97] with permission from Elsevier.

**Figure 50.**
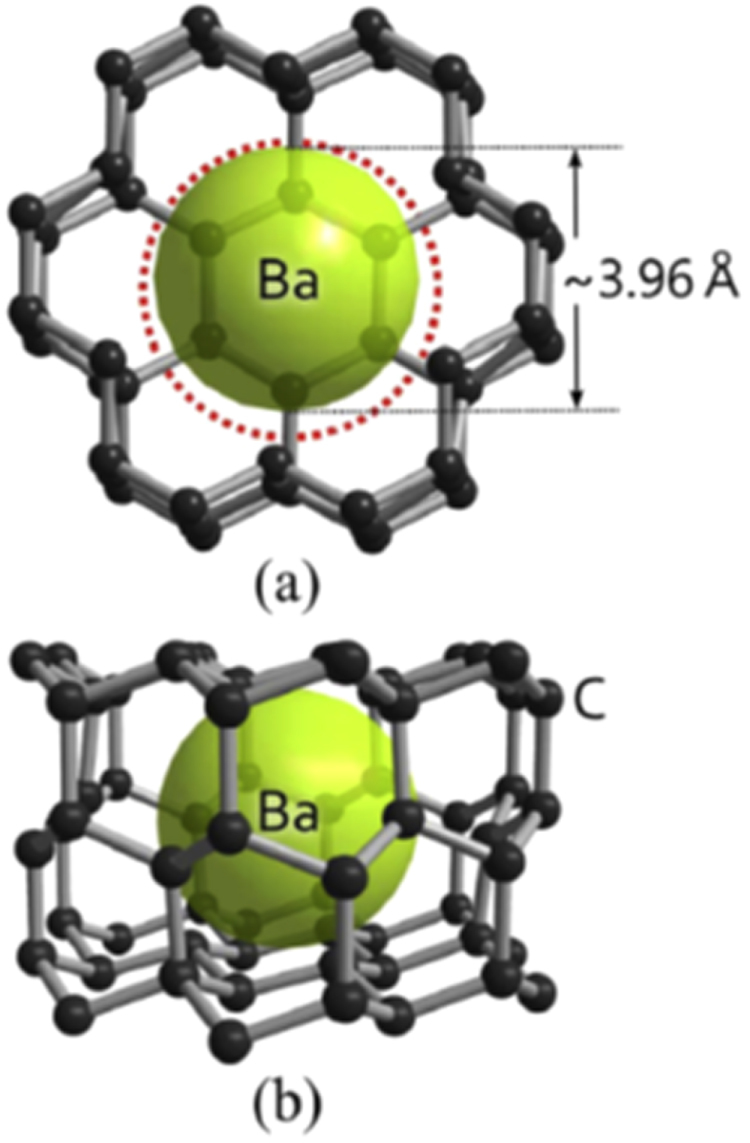
Schematic illustrations of a local arrangement of carbon network encapsulating a Ba atom in Ba_3_C_60_ collapsed by HPHT treatment, (a) top and (b) side views; the Ba atom (covalent diameter 3.96 Å) replaces carbon atoms in a six-membered ring with comparable size. The covalent diameter (4.2 Å) of a six-membered carbon ring (red circle in (a)) includes the diameter of the six-membered ring (2.9 Å) and a fringe with a width of a half length of the C–C bonds formed with carbon atoms surrounding the ring. Reprinted from [97] with permission from Elsevier.

The powder Ba_3_C_60_ sample was compressed using a Kawai-type multianvil press at 5–15 GPa and 500–1150 °C [97]. The x-ray powder diffraction (XRD) pattern showed that the Ba_3_C_60_ compressed under 15 GPa at 900 °C is changed into an amorphous solid, which was found to be chemically stable in air and even in water. The high Vickers micro-hardness (1700 kg mm^−2^) and the Raman spectra of the solids suggest that the C_60_ molecules are collapsed to form an amorphous 3D polymer encapsulating the Ba atoms. As shown in figure 49, the solid obtained by compression under 15 GPa at 900 °C shows a semi-metallic conductivity. It is interesting to note that the Hall coefficient of this sample is positive in the whole temperature range <300 °C, indicating that the dominant carriers are holes. As shown in figure 50, the covalent diameter of a Ba atom is too large to substitute one C atom in the carbon matrix. The diameter is rather comparable with the covalent diameter of a six-membered carbon ring. If a six-membered carbon ring with 12 electrons in the *sp*^3^ hybridized orbitals is substituted by a Ba atom with a similar size having only two valence electrons, the carbon matrix should be efficiently hole-doped. The electrical characteristics such as conductivity, Hall coefficient, carrier density, and mobility of the carbon matrix encapsulating Ba atoms were found to be comparable with those of B-doped diamond [97]. The Ba-encapsulated carbon matrix obtained in this study is amorphous. It is interesting to prepare crystalline carbon analogs for silicon clathrate compounds.

### 3.4. Other new superconductors

Besides superconductors described in the preceding sections, many new superconductors have been discovered in this project. In this section, some of them are overviewed.

#### 3.4.1. AM_2_X_2_-type (122-type, M ≠ Fe) superconductors

**Figure 51.**
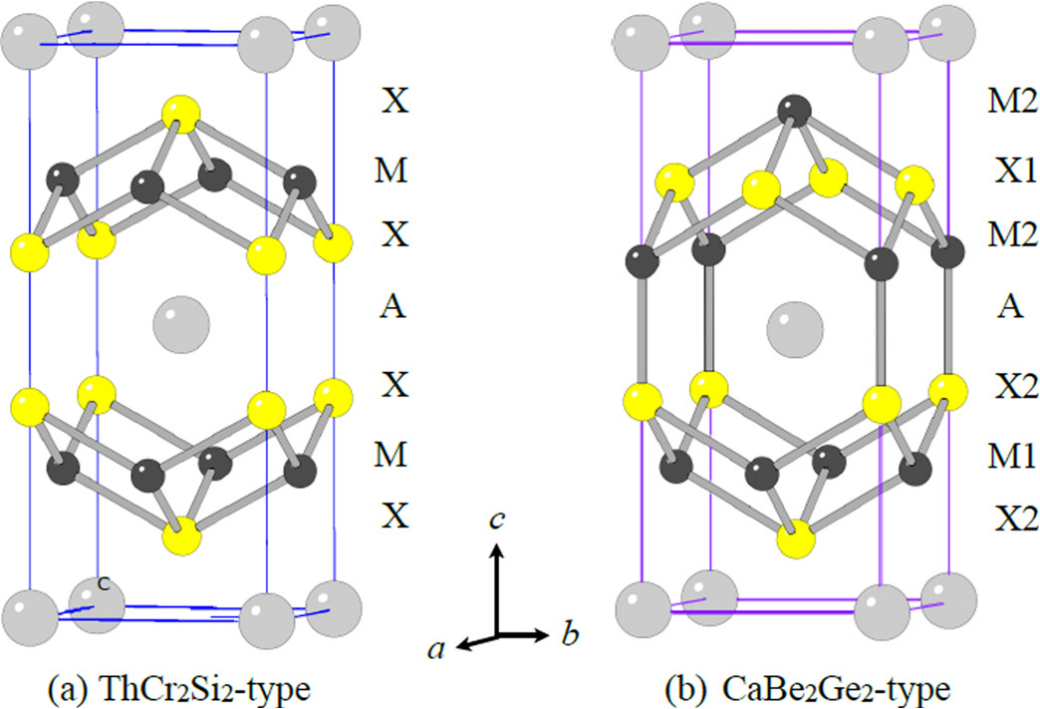
Comparison of the crystal structures of (a) ThCr_2_Si_2_-type and (b) CaBe_2_Ge_2_-type *AM*_2_*X*_2_ compounds. In the latter structure, two nonequivalent sites are present for both the *M* and *X* atoms as denoted by the suffixes 1 and 2. Reprinted with permission from [350]. Copyright 2010 by the Physical Society of Japan.

The 122-type iron arsenide superconductors *AE*Fe_2_As_2_ (*AE* = Ba, Ca, Sr and Eu) have been investigated intensively by many groups including the FIRST Project team (see the preceding section). These compounds have the ThCr_2_Si_2_-type structure and non-iron 122-superconductors with the same structure have also been studied widely; this class of compounds includes SrNi_2_P_2_ [342], BaNi_2_P_2_ [343], BaRh_2_P_2_, BaIr_2_P_2_ [344], LaRu_2_P_2_ [345], LiCu_2_P_2_ [346], SrNi_2_As_2_ [347], BaNi_2_As_2_ [348], etc. The *AEM*_2_*X*_2_ compounds sometimes crystallize in different polymorphs with CaBe_2_Ge_2_-type structure [349]. The two structures of ThCr_2_Si_2_-type and CaBe_2_Ge_2_-type are deeply concerned with each other as compared in figure 51 [350]. In ThCr_2_Si_2_-type *AEM*_2_*X*_2_, *A*, *M* and *X* atom planes are stacked in a sequence of *A*–(*X*–*M*_2_–*X*)-… along the *c*-axis of the tetragonal cell, forming an *MX*_4_ coordination tetrahedron. In the CaBe_2_Ge_2_-type structure, on the other hand, the sequence of the atom planes is *AE*–(*X*–*M*_2_–*X*)–*AE*–(*M*–*X*_2_–*M*)… where the *XM*_4_ tetrahedron is formed as well as the *MX*_4_ tetrahedron. Fewer compounds had been known for the CaBe_2_Ge_2_-type, and SrPt_2_As_2_ with *T*_c_ = 5.2 K is a rare example of the superconductor having this structure [350] (strictly speaking, its structure is an incommensurate orthorhombic variant of the CaBe_2_Ge_2_-type structure [351]). Band structure calculations for CaBe_2_Ge_2_-type SrPt_2_As_2_ revealed that it has two two-dimensional (2D) like Fermi surfaces as well as two three-dimensional (3D) like ones in contrast to the strong 2D character in the electronic structure of ThCr_2_Si_2_-type *A*Fe_2_As_2_ [352]. Several 122-type superconductors with ThCr_2_Si_2_-type, CaBe_2_Ge_2_-type or related structures were discovered in the FIRST Project as overviewed below.

##### 3.4.1.1. 122-type antimonides and related compounds

**Figure 52.**
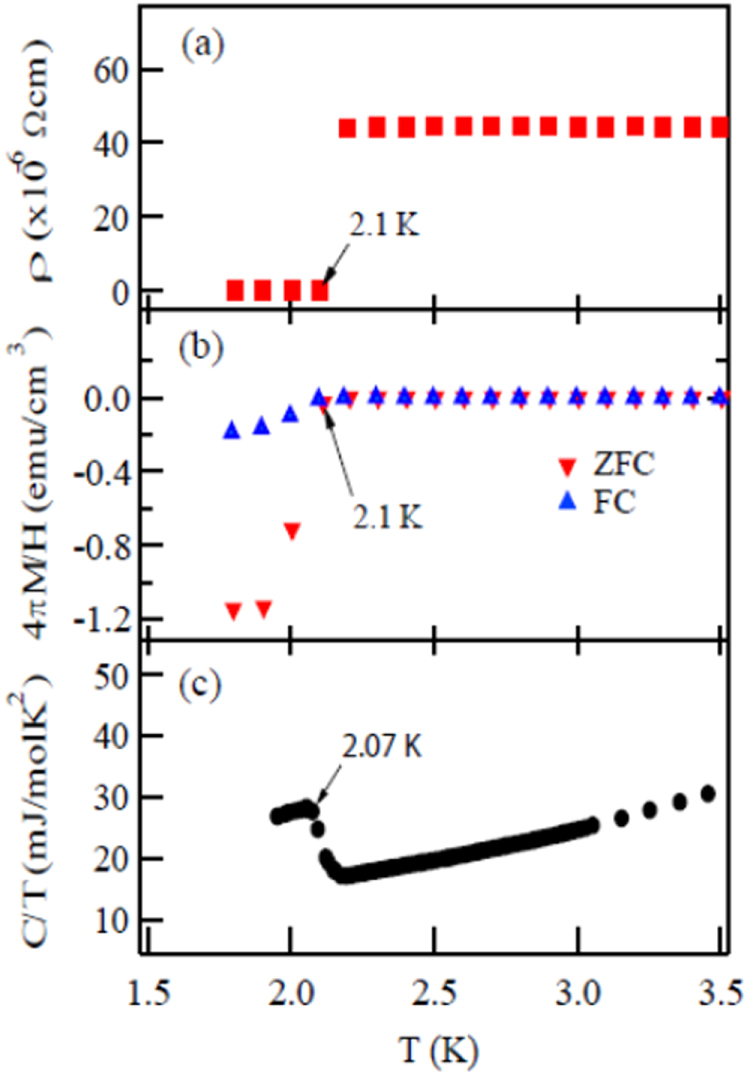
(a) Electrical resistivity (b) magnetization in zero field cooling (ZFC) and field cooling (FC) and (c) specific heat as functions of temperature for SrPt_2_Sb_2_. Reprinted with permission from [41]. Copyright 2013 by IOP Publishing.

The SrPt_2_Sb_2_ had been known to have the CaBe_2_Ge_2_-type structure with tetragonal cell of *a* = 4.603 and *c* = 10.565 Å [351] but any physical properties had not been reported. In the FIRST Project, this material was revisited to elucidate its physical properties [41]. Samples were synthesized starting from Sr, Pt and Sb in two-step procedures: arc melting and re-melting of the arc-melted specimen. Though electron probe microanalysis confirmed the composition of SrPt_2_Sb_2_ (122) for the major part of the ingot obtained, the powder x-ray pattern was not consistent with the CaBe_2_Ge_2_-type tetragonal lattice. Thus, SrPt_2_Sb_2_ has a different structure which may be some derivative of the CaBe_2_Ge_2_-type. As shown in figure 52, electrical resistivity, magnetization and specific heat measurements confirmed a bulk superconducting transition at *T*_c_ = 2.1 K for SrPt_2_Sb_2_. It is a type-II superconductor with a lower critical field (*H*_c1_) of 6 Oe and upper critical field (*H*_c2_) of 1 kOe at 1.8 K. Debye temperature (*Θ*_D_) and electronic specific heat coefficient (*γ*) were derived from specific heat data to be *Θ*_D_ = 183 K and *γ* = 9.2 mJ (mol K^2^)^−1^. The normalized specific heat jump at *T*_c_ was calculated as *ΔC*(*T*_c_)/*γT*_c_ = 1.29, consistent with the BCS weak coupling limit of 1.43. Normal state electrical resistivity of SrPt_2_Sb_2_ exhibited anomalies around 250 K with thermal hysteresis which corresponded to a certain structural transition, though its detail has not yet been elucidated. SrPt_2_Sb_2_ is a 122-type superconducting antimonide discovered for the first time, and detailed studies for the structure and the phase transition are greatly desired.

**Figure 53.**
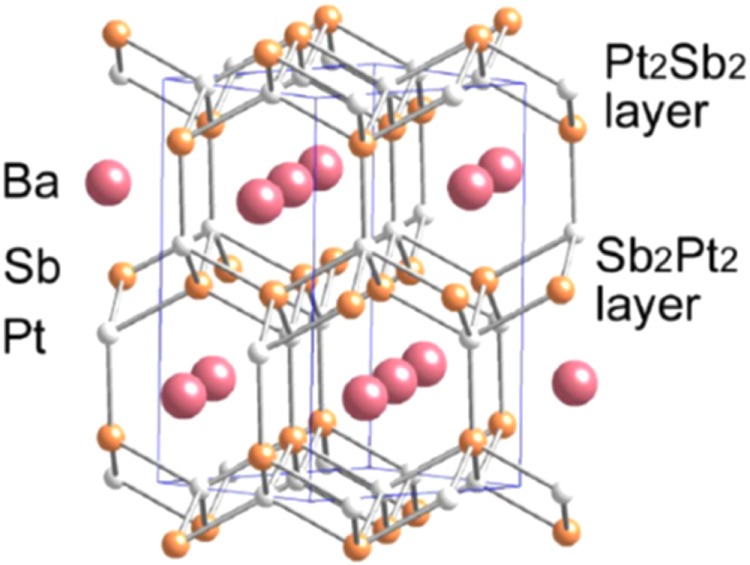
Crystal structure of BaPt_2_Sb_2_. Reprinted with permission from [42]. Copyright 2015 by American Physical Society.

**Figure 54.**
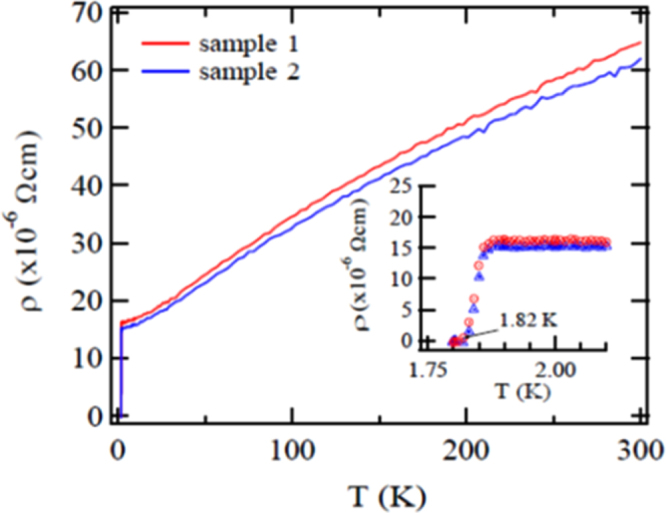
Temperature dependences of the electrical resistivity of two BaPt_2_Sb_2_ samples. The inset is an expanded view of the resistivity at low temperatures ranging from 1.8 to 2.2 K. Reprinted with permission from [42]. Copyright 2015 by American Physical Society.

The Ba-derivative of the aforementioned compound, BaPt_2_Sb_2_ had not been reported, and was prepared first in the FIRST Project by the arc melting method for a Ba, Pt and Sb mixtures [42]. The x-ray pattern of the BaPt_2_Sb_2_ sample was consistent with a monoclinic lattice having parameters of *a* = 6.702 Å, *b* = 6.752 Å, *c* = 10.47 Å and *β* = 91.23°. The structure of BaPt_2_Sb_2_ is shown in figure 53, which can be interpreted as a monoclinic variant of the CaBe_2_Ge_2_-type structure (note that the *a*-axis and *b*-axis of BaPt_2_Sb_2_ correspond to diagonal of the *a*_0_-axis and *b*_0_-axis of the original CaBe_2_Ge_2_-type lattice with the relationship of *a*(*b*) ≈ √2*a*_0_). Figure 54 gives the temperature dependency of the resistivity of BaPt_2_Sb_2_, which confirms superconducting transition at 1.8 K. Specific heat data gave *Θ*_D_ = 146 K and *γ* = 8.6 mJ (mol K^2^)^−1^, deriving *ΔC*(*T*_c_)/*γT*_c_ = 1.37, which is comparable with the BCS weak coupling limit. Magnetization measurements revealed type-II superconductivity with *μ*_0_*H*_c2_(0) = 0.27 T and Ginzburg–Landau (GL) coherent length *ξ*_GL_(0) = 350 Å. Band structure calculations revealed that Fermi surfaces of BaPt_2_Sb_2_ resemble those of SrPt_2_As_2_ with two 2D-like Fermi surfaces and two 3D-like ones. Thus, it has more 3D-like character compared with the AFe_2_As_2_ system, which may account for the relatively lower *T*_c_ of BaPt_2_Sb_2_.

**Figure 55.**
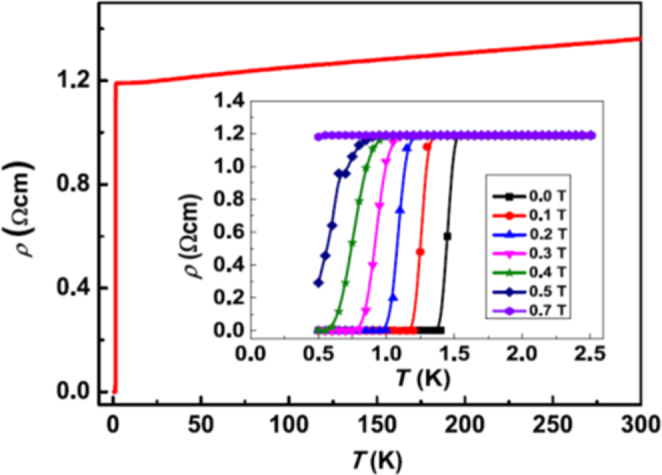
Temperature dependence of electrical resistivity for LaPd_2_Sb_2_. The inset shows the magnetic field dependence. Reprinted from [39] with permission from Elsevier.

**Figure 56.**
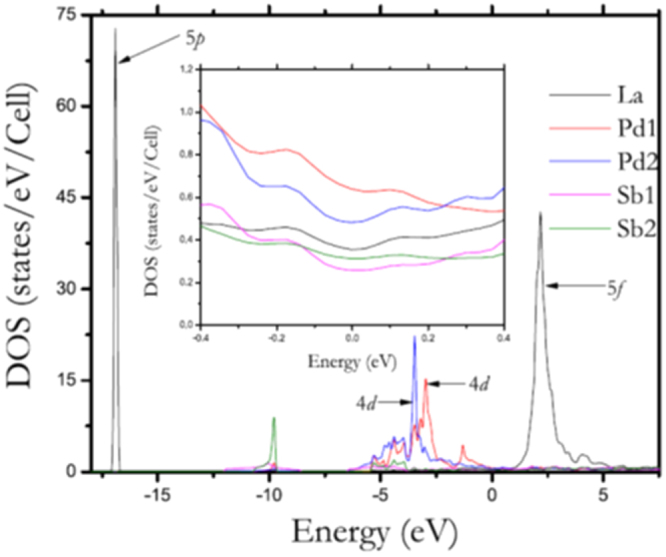
Calculated total density of states for La, Pd1, Pd2, Sb1 and Sb2 (see figure 51 on the atom sites with the suffixes of 1 and 2). The inset shows the contribution to Fermi level from each atom. Reprinted from [39] with permission from Elsevier.

Pd-based CaBe_2_Ge_2_-type antimonides such as *Ln*Pd_2_Sb_2_ (*Ln* = La, Ce, Pr, Nd, Eu) [353] were known to exist but superconductivity was not reported for this class of compounds. In the FIRST Project, LaPd_2_Sb_2_ was selected among them and reinvestigated in detail to find the superconducting transition [39]. The LaPd_2_Sb_2_ sample was prepared at 900 °C starting from La, Sb and Pd in an evacuated silica tube. The resulting specimen was a single phase of the CaBe_2_Ge_2_-type compound though its tetragonal lattice parameters (*a* = 4.568 Å, *c* = 10.266 Å) were slightly different from the previous report [353]. Figure 55 shows the temperature dependence of resistivity for LaPd_2_Sb_2_ under zero and various magnetic fields. The superconducting transition occurs at onset temperature of 1.4 K and zero resistivity temperature of 1.2 K at *H* = 0. Magnetization measurements revealed type-II nature superconductivity with *μ*_0_*H*_c2_(0) = 0.86 T and *ξ*_GL_(0) = 233 Å. Bulk superconductivity was confirmed by specific heat measurements which gave *Θ*_D_ = 210 K, *γ* = 6.89 mJ (mol K^2^)^−1^ and *ΔC*(*T*_c_)/*γT*_c_ = 1.325, consistent with the BCS weak coupling limit. Figure 56 gives the total density of states for La, Pd1, Pd2, Sb1 and Sb2 (see figure 51 on the atom sites with the suffixes of 1 and 2) calculated by DFT. It is seen that Pd contributes the most to the total DOS at the Fermi level, consistent with a general tendency that the DOS at the Fermi level are dominated by the *d*-band of the *M* atom in CaBe_2_Ge_2_-type *AM*_2_*X*_2_. Figure 56 reveals hybridization between Pd 4*d* of Pd1 (Pd2) and Sb 5*p* of Sb2 (Sb1) as demonstrated by synchronized modulation in partial DOS of Pd1 (Pd2) and Sb2 (Sb1). The total DOS at the Fermi level is nearly 40% of that of SrPt_2_As_2_, which may account for relatively lower *T*_c_ of LaPd_2_Sb_2_ compared with SrPt_2_As_2_.

The As-derivative of the aforementioned compound, LaPd_2_As_2_, which was known to have a ThCr_2_Si_2_-type structure [353], was also studied in the FIRST Project [40]. In the ThCr_2_Si_2_-type *AEM*_2_*X*_2_, interlayer *X–X* bonding is sometimes formed between the adjacent *M*_2_*X*_2_ layers to give the collapsed tetragonal (cT) structure with a 3D network. Superconductivity in the collapsed Fe_2_As_2_ planes is often attained under high pressure taking well known examples of *AE*Fe_2_As_2_ (*AE* = Ca, Sr, Ba, Eu) which undergo both superconducting and collapsed transitions under high pressure [354–357]. Superconductivity under ambient pressure in the cT structure is rather rare and has been reported for *AE*Pd_2_As_2_ (*AE* = Ca, *T*_c_ = 1.27 K; *AE* = Sr, *T*_c_ = 0.92 K) [358]. In the FIRST Project, it was elucidated that LaPd_2_As_2_ has the cT structure under ambient pressure with the interlayer As–As distance of 2.318 Å, slightly shorter than the covalent single bond of 2.38 Å for As. Moreover, it was confirmed for the first time that this cT phase shows type-II superconductivity below 1 K. The superconducting and physical parameters obtained for LaPd_2_As_2_ are *μ*_0_*H*_c2_(0) = 0.402 T (by the Werthamer–Helfand–Hohenberg (WHH) theory using the *ρ*_90%_ point as *T*_c_), *ξ*_GL_(0) = 137 Å, *Θ*_D_ = 261 K, *γ* = 5.56 mJ (mol K^2^)^−1^ and *ΔC*(*T*_c_)/*γ T*_c_ = 1.17. The density of states at the Fermi level calculated from the specific heat data is as small as ∼0.84 states per eV per formula unit (fu) which may explain the relatively low *T*_c_ of this cT phase.

##### 3.4.1.2. Co-based superconductor LaCo_2_B_2_

**Figure 57:**
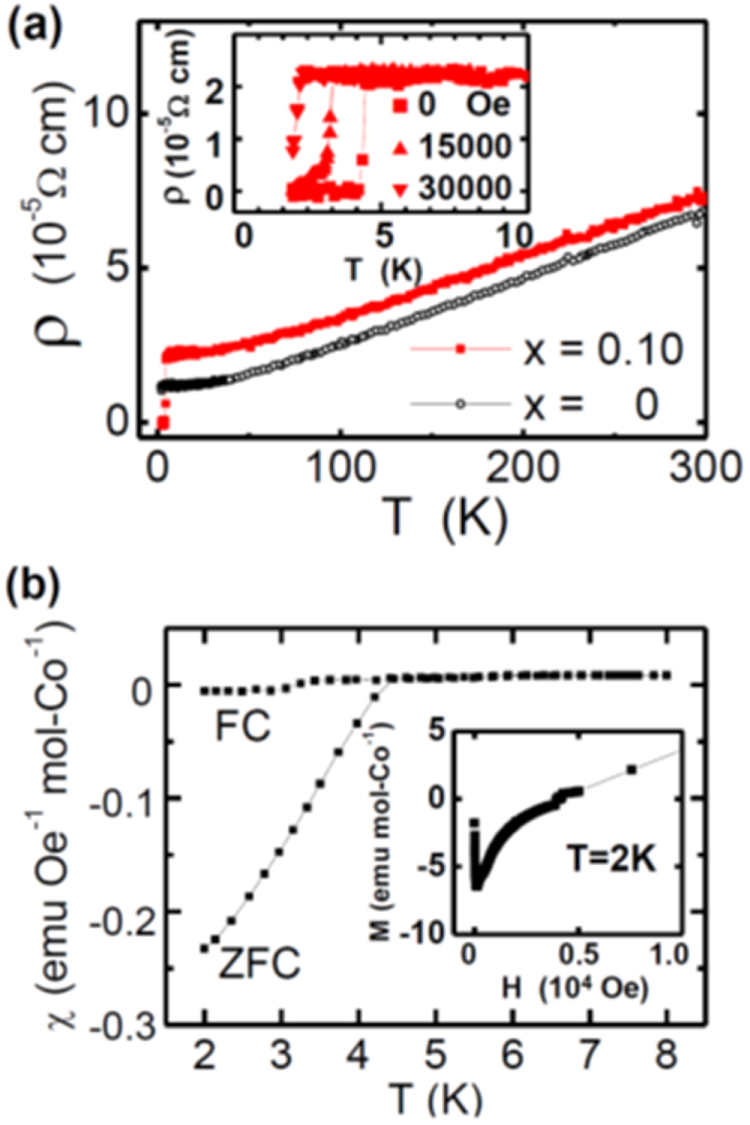
(a) Temperature dependence of electrical resistivity for (La_1−*x*_Y_*x*_)Co_2_B_2_. The inset shows temperature dependence of resistivity for *x* = 0.1 in magnetic field. (b) Temperature dependence of the magnetic susceptibility for the *x* = 0.1 sample under ZFC and FC conditions at 10 Oe. The inset shows the field dependence of magnetization at 2 K. Reprinted with permission from [34]. Copyright 2011 by American Physical Society.

Only few reports are available for Co-based superconducting compounds [359, 360]. In the FIRST Project, LaCo_2_B_2_, which was reported first in 1973 [361], was re-visited and was found to be superconducting after isovalent or aliovalent substitution for the constituent cations [34]. Polycrystalline samples of (La_1−*x*_Y_*x*_)Co_2_B_2_, La(Co_1−*x*_Fe_*x*_)_2_B_2_ and LaCo_2_(B_1−*x*_Si_*x*_)_2_ as well as the mother compound were prepared by the arc-melting method for La, Co, B, Y, Fe mixtures. Powder x-ray diffraction of the arc-melted LaCo_2_B_2_ sample was consistent with the tetragonal ThCr_2_Si_2_-type structure as reported previously [361], having lattice parameters of *a* = 3.610 Å and *c* = 10.20 Å. As shown in figure 57, the 10% Y-doped sample showed superconductivity below 4.4 K. Magnetic susceptibility data revealed Pauli paramagnetism for the normal state of this compound and the *M–H* curve (figure 57(b) inset) at 2 K reveals type-II nature of superconductivity. Superconductivity with *T*_c_ ∼ 4 K was also seen in the Fe-doped system of La(Co_1−*x*_ Fe_*x*_)_2_B_2_ for *x* ≥ 0.1 while the Si-doped samples did not show superconductivity.

**Figure 58:**
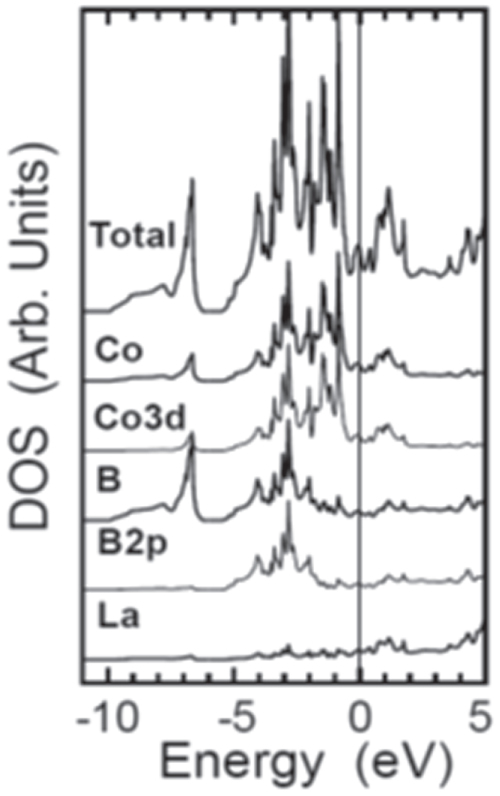
Calculated DOS of LaCo_2_B_2_, with the PDOS for La, Co, Co 3*d*, B, and B 2*p*. Reprinted with permission from [34]. Copyright 2011 by American Physical Society.

Electronic structure of LaCo_2_B_2_ was investigated theoretically by the DFT method; calculated DOS is shown in figure 58. From the DFT calculation, it was confirmed that La ions take +3 state and metallic conduction occurs in the CoB layer composed of highly covalent Co and B. This strong covalency suppresses the spin moment of the Co ion resulting in the Pauli paramagnetic state. Such a situation is caused by the relatively shallow B 2*p* level compared with the As 4*p* level in LaFeAsO where antiferromagnetic state is realized rather than the paramagnetic one.

##### 3.4.1.3. Enhancement of superconductivity by phosphorus doping in BaNi_2_As_2_

BaNi_2_As_2_ has a tetragonal ThCr_2_Si_2_-type structure at room temperature [348, 362, 363] but it undergoes a structural transition at ∼130 K to a triclinic form where alternate Ni–Ni bonds are formed in the Ni plane (see figure 60(a)) [362]. Below 0.7 K, the triclinic phase shows superconductivity, which is believed to be of conventional BCS-type [348, 363–365]. In the FIRST Project, BaNi_2_As_2_ was reinvestigated because of this unique structural transition, i.e., this material was expected to offer a stage for studying chemical tuning of soft phonons by elemental substitution and its effects on superconductivity [35].

**Figure 59.**
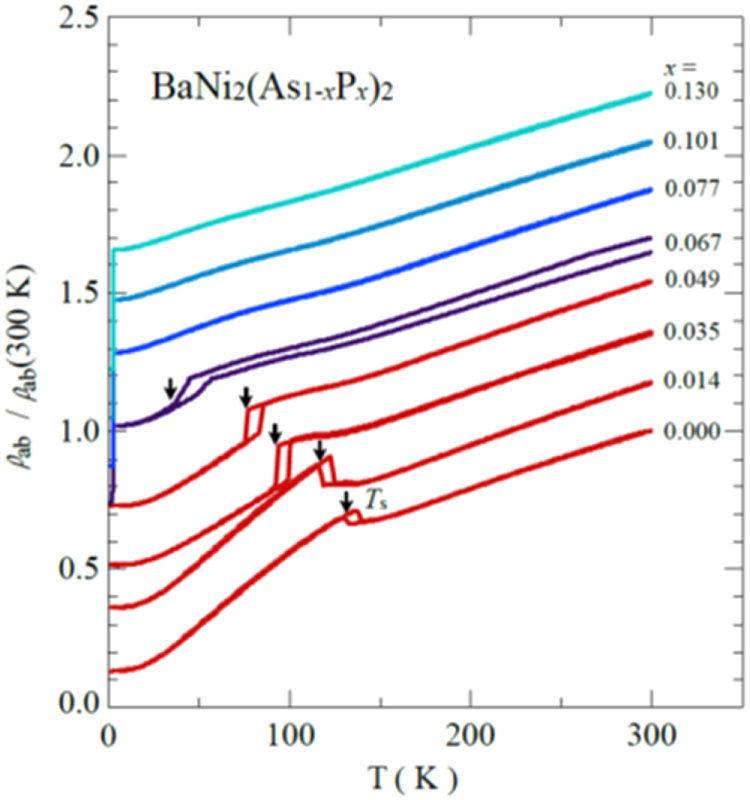
Temperature dependence of electrical resistivity parallel to the *ab* plane, *ρ*_*ab*_, normalized by the value at 300 K for BaNi_2_(As_1−*x*_P_*x*_)_2_. The data measured upon heating and cooling are plotted. For the sake of clarity, *ρ*_*ab*_/*ρ*_*ab*_(300 K) is shifted by 0.175 with respect to all data. *T*_s_ is the phase transition temperature at which the tetragonal-to-triclinic phase transition occurs. Reprinted with permission from [35]. Copyright 2012 by American Physical Society.

**Figure 60.**
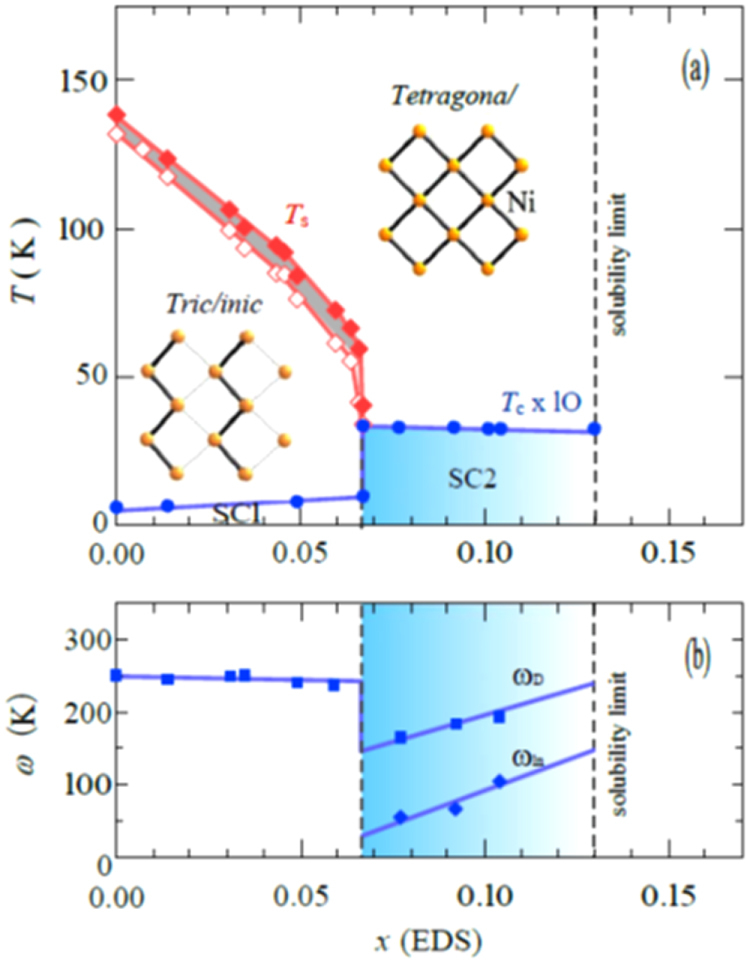
(a) Electronic phase diagram of BaNi_2_(As_1−*x*_P_*x*_)_2_. The closed circles represent the superconducting transition temperatures *T*_c_. For clarity, the values of *T*_c_ have been scaled by a factor of 10. SC1 and SC2 denote the superconducting phases. The open and closed diamonds represent the tetragonal-to-triclinic structural transition temperatures *T*_s_ upon cooling and heating, respectively. The insets show schematic views of Ni planes in the triclinic and tetragonal phase. (b) Debye frequency *ω*_D_ and logarithmic averaged phonon frequency *ω*_ln_ as a function of phosphorus content *x*. Reprinted with permission from [35]. Copyright 2012 by American Physical Society.

Single crystals of BaNi_2_(As_1−*x*_P_*x*_)_2_ were grown by a self-flux method starting from a mixture of Ba, NiAs, Ni and P [35]. The solubility limit was determined to be *x* = 0.13 and powder x-ray diffraction confirmed the single-phase nature for all specimens with *x* ≤ 0.13. Figure 59 shows the temperature dependence of the electrical resistivity parallel to the *ab* plane of the BaNi_2_(As_1−*x*_P_*x*_)_2_ crystal. The tetragonal-to-triclinic transition is clearly seen in this figure as the resistivity anomaly with thermal hysteresis. The transition temperature decreases with increasing *x* and finally the triclinic phase disappears for *x* ≧ 0.07. Enhancement of superconductivity by phosphorus doping is striking; *T*_c_, which is below 0.7 K for the triclinic phase with *x* < 0.07, is suddenly increased to 3.33 K in the tetragonal phase with *x* = 0.077. Figure 60 shows the phase diagram of the BaNi_2_(As_1−*x*_P_*x*_)_2_ system. Triclinic phase formation is suppressed by phosphorus doping and instead superconductivity is enhanced drastically following the disappearance of the triclinic phase. The Debye frequency *ω*_D_ and logarithmic averaged phonon frequency *ω*_ln_ calculated from the specific heat of the tetragonal phase exhibit significant softening near the tetragonal-to-triclinic phase boundary. The low-lying soft phonons seem to play, being strongly coupled with acoustic modes, an important role in the enhancement of superconductivity in the tetragonal phase. Indeed, the normalized specific heat jump *ΔC*(*T*_c_)/*γT*_c_ is increased from 1.3 in the triclinic phase to 1.9 in the tetragonal phase with *x* = 0.077, i.e., the system is transformed from the weak coupling regime to the strong coupling one by phosphorus doping. Such a mechanism of *T*_c_ enhancement is common to other systems of CaC_6_ [366–368] and Te [369] under high pressure.

#### 3.4.2. Transition metal dichalcogenides

The transition metal dichalcogenides, *MX*_2_ have renewed interest recently and were studied intensively in this project. In this section, two topics are presented; one is the CdI_2_-type telluride family and the other is the pyrite (FeS_2_)-type chalcogenide family.

##### 3.4.2.1. CdI_2_-type tellurides

**Figure 61.**
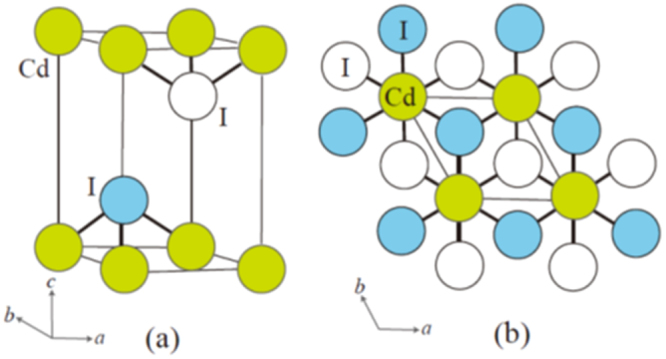
Crystal structure of CdI_2_: (a) unit lattice and (b) composite plane consisting of the CdI_6_ octahedra projected on the *ab* plane where the I atom, represented by the white (blue) circle, is located below (above) the Cd plane.

**Figure 62.**
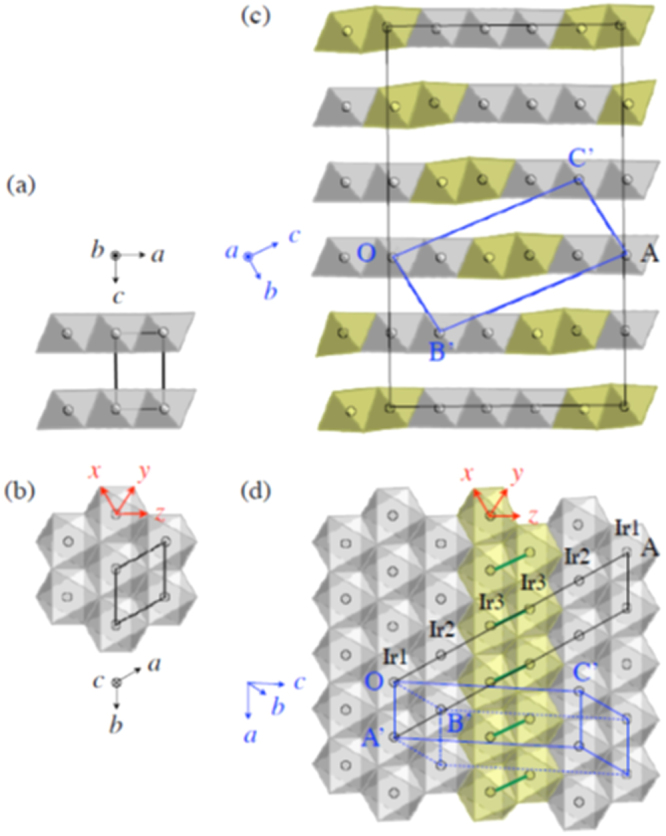
(a), (b) The crystal structure of IrTe_2_ at 300 K. Black lines represent the unit cell of the trigonal lattice. (c), (d) The crystal structure of IrTe_2_ at 20 K. Blue lines represent the unit cell of the triclinic lattice. Black lines represent the 5*a* × *b* × 5*c* supercell (*a*, *b*, and *c* are the high-temperature trigonal-cell parameters). Reprinted with permission from [374]. Copyright 2014 by the Physical Society of Japan.

IrTe_2_ has a trigonal CdI_2_-type structure (see figure 61) with the space group of *P*
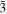
*m*1. The Ir atom is octahedrally coordinated by the Te atoms and the edge-sharing of the IrTe_6_ octahedron forms the Te–Ir–Te composite layers which are stacked along the *c*-axis of the trigonal lattice. Both the Ir and Te atoms form 2D regular triangular lattices within the *ab*-plane with three equivalent Ir–Ir (Te–Te) bonds. IrTe_2_ undergoes a first-order transition at ∼250 K transforming to a low temperature phase for which a monoclinic structure was first proposed [370]. Recently, a CDW-like superlattice modulation with wave vector of ***q*** = (1/5,0,−1/5) was observed in electron diffraction patterns for the low temperature phase [371]. Considering the partly filled *d*-orbitals of the Ir atoms, the orbital degree of freedom is believed to play an important role in this transition [372, 373]. In this project, the low temperature structure was analyzed in detail using x-ray data for single crystals [374]. The structure at 20 K was found to be triclinic (space group *P*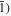 as shown in figure 62. In this triclinic structure, one out of five Ir–Ir bonds along the trigonal *a*-axis shrink considerably forming Ir–Ir dimers as illustrated by the yellow hatch in figure 62. The plane of the dimers propagates with the vector of ***q*** = (1/5, 0, −1/5) consistent with the aforementioned electron diffraction data. This dimerization seems to affect the physical properties of the system seriously as the first-principles band calculations indicated that tilted two-dimensional Fermi surfaces emerge in the triclinic phase with a possible switching of the conduction plane from the vassal (*ab*) plane in the trigonal phase to tilted plane normal to *q* in the triclinic phase [374].

**Figure 63.**
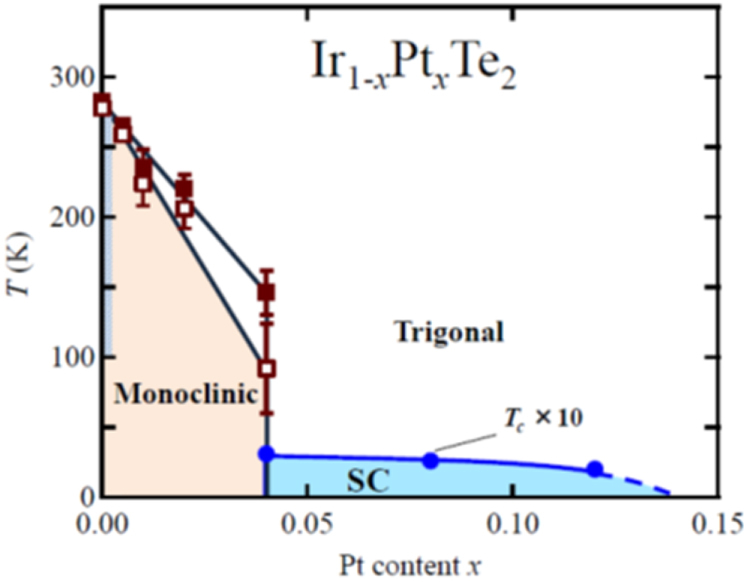
Phase diagram of Ir_1−*x*_Pt_*x*_Te_2_ determined using single-crystal samples. Closed and open squares respectively represent the trigonal–monoclinic transition temperature *T*_s_ upon heating and cooling (the ‘monoclinic’ phase corresponds to the triclinic phase in figure 62, representing the simplified symmetry). Closed circles represent the *T*_*c*_ and SC represents the bulk superconducting region. The hatched area represents a temperature range of broad hysteresis in resistivity. Reprinted with permission from [63]. Copyright 2012 by the Physical Society of Japan.

It was found in 2011 that partial substitution of Pt for Ir in IrTe_2_ suppresses the formation of the low temperature phase resulting in the appearance of superconductivity [375, 376]. A phase diagram of the Ir_1−*x*_Pt_*x*_Te_2_ system was first determined for the polycrystalline samples [63] and then by single crystal data in the FIRST Project [377]. Figure 63 is the phase diagram based on single crystal data; superconductivity appears for ∼0.04 < *x* < ∼0.14 in Ir_1−*x*_Pt_*x*_Te_2_ with the highest *T*_c_ of ∼3.2 K for *x* = 0.04 at the phase boundary of the trigonal and triclinic phases (the monoclinic phase in this figure corresponds to the triclinic phase in figure 62, representing the simplified symmetry). For the polycrystalline sample with *x* = 0.04, type-II superconductivity with *μ*_0_*H*_c2_(0) = 0.17 T and *ΔC*(*T*_c_)/*γT*_c_ = 1.5 has been reported [63]. The Pt substitution works as the electron doping shifting the Fermi level upward and affecting the DOS near the Fermi level (it causes an increase of DOS near the Fermi level in the triclinic phase, and a decrease of DOS in the trigonal phase). Another effect of the substitution, which seems to be more essential, is the suppression of the triclinic phase by breaking the Ir–Ir dimers.

Similar results on suppression of the phase transition and appearance of superconductivity have been reported for several systems such as Ir_1−*x*_Pd_*x*_Te_2_ [371], Pd_*x*_IrTe_2_ [371] and Cu_*x*_IrTe_2_ [378], where substitution or intercalation of Pd or Cu brings about essentially the same effect as the Pt substitution. In the FIRST Project, two more advances were made on IrTe_2_ and related systems. The first is the isovalent Rh doping for IrTe_2_ [64]. The Ir_1−*x*_Rh_*x*_Te_2_ system shows similar features to Ir_1−*x*_Pt_*x*_Te_2_, i.e., triclinic phase formation is suppressed by Rh doping, resulting in the appearance of superconductivity with *T*_c_ ∼ 2.6 K, despite that band filling is unchanged by the isovalent Rh doping as long as a rigid-band picture is concerned. A distinct difference was, however, seen between Ir_1−*x*_Rh_*x*_Te_2_ and Ir_1−*x*_Pt_*x*_Te_2_; in the former phase, a doping of *x* ∼ 0.1 is needed for the complete suppression of the triclinic phase, which is three times larger than *x* = 0.03 in the latter. This difference seems to be caused by the lower volume expansion and the aforementioned unchanged band filling in the case of Rh doping

**Figure 64.**
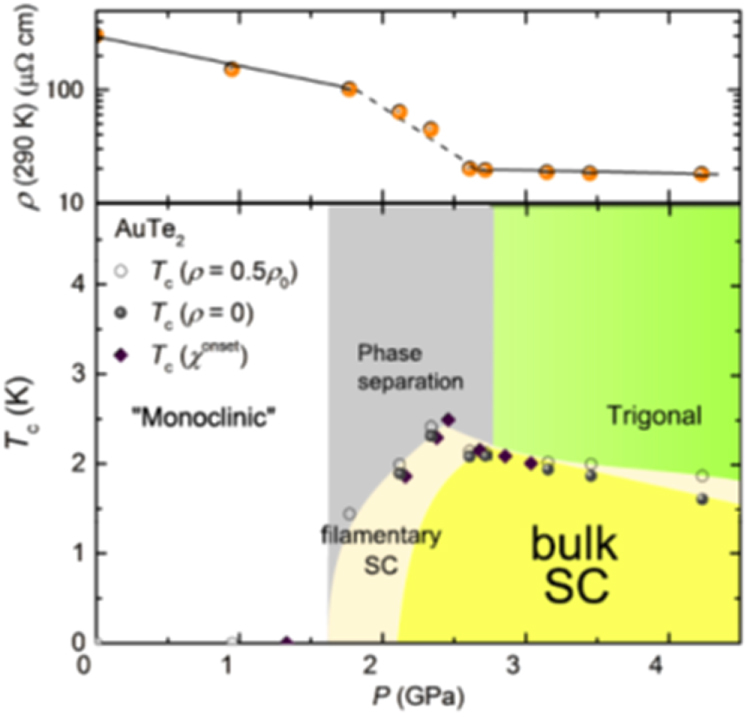
(Top) Pressure dependence of resistivity at 290 K, *ρ*(290 K) (since the applied pressure decreases by approximately 0.2 GPa on cooling, the actual pressure at 290 K is larger than the displayed pressure). (Bottom) *T–P* phase diagram for AuTe_2_. Open (filled) circles represent *T*_*c*_ determined using *ρ*_50%_ point and filled diamonds indicate *T*_*c*_ determined by the onset of the diamagnetic shielding signal [61]. (The ‘monoclinic’ phase corresponds to the triclinic phase in figure 62, representing the simplified symmetry.) Reprinted with permission from [61]. Copyright 2013 by the Physical Society of Japan.

Another advance was attained from the study of a related system of Au_1−*x*_Pt_*x*_Te_2_ [62]. AuTe_2_ has a monoclinically distorted CdI_2_-type average structure with a space group of *C*2/*m* where Te–Te zigzag chains run along the *a*-axis [379]. In actuality, the structure is associated with incommensurate modulation with a wave vector ***q*** = −0.4076*a*∗ + 0.4479*c*∗ and due to this modulation, Te–Te dimers with a short distance of 2.88 Å exist in the real structure instead of the zigzag chains [380, 381]. It was found that Pt doping brings about structural change from the distorted CdI_2_-type for *x* = 0 to distortion-free CdI_2_-type without Te–Te dimers for *x* = 0.35, via the two-phase mixed region for *x* = 0.1 and 0.15 [62]. Type-II superconductivity was observed for the distortion-free *x* = 0.35 sample with *T*_c_ = 4.0 K, *H*_c2_(0) = 12.9 kOe and *ξ*_GL_(0) = 160 Å. The *ΔC*(*T*_c_)/*γT*_c_ is 1.57, exceeding the BCS weak coupling limit. It should be noted that superconductivity appears by breaking of the Te–Te dimers, which shows striking similarity with the Ir_1−*x*_Pt_*x*_Te_2_ system where superconductivity appears by breaking of the Ir–Ir dimers. It was also found that superconductivity is induced in AuTe_2_ by application of pressure instead of Pt doping [61]. As shown in the phase diagram of figure 64, application of mechanical pressure causes a structural change from the distorted CdI_2_-type to the distortion-free CdI_2_-type via the two-phase mixed region. Superconductivity appears above 2.12 GPa with the highest *T*_c_ of 2.3 K at 2.34 GPa.

##### 3.4.2.2. Pyrite-type chalcogenide family

**Figure 65.**
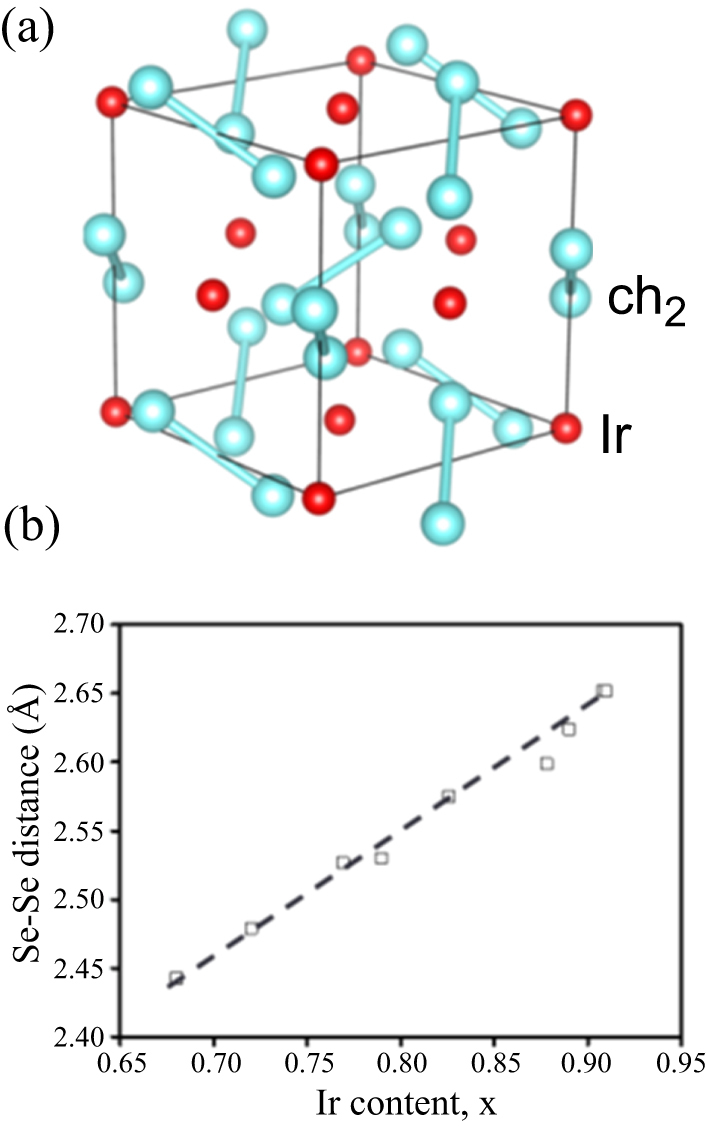
(a) Crystal structure of pyrite Ir_*x*_Se_2_. (b) Variation of the Se–Se distance in the dimer anions with the Ir content *x*. Reprinted with permission from [65]. Copyright 2012 by the American Physical Society.

Iridium dichalcogenides Ir*X*_2_ (*X* = Se and Te) sometimes take pyrite-type (FeS_2_-type) structures after introduction of vacancies for the Ir sites [382]. It is also known that application of high pressure is effective to stabilize the pyrite-type form against the Cd_2_I_2_-type one [383]. In the Ir-deficient phase Ir_*x*_*X*_2_, Ir vacancies are distributed randomly forming a cubic pyrite-type structure with a space group of *Pa*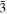 (figure 65). This structure can be interpreted as the NaCl-type constructed by the face centered cubic sublattice of Ir and the *X–X* dimers located at the center of each edge as well as at the body center of the cubic lattice. In the Ir_*x*_*X*_2_ phase with the particular value of *x* = 0.25, Ir vacancies tend to be distributed in an ordered way in which one of four Ir atoms are regularly removed [382]. The vacancy ordering results in a stoichiometric phase of Ir_3_*X*_8_ having a rhombohedral structure with the space group of *R*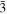 (in Ir_3_*X*_8_, the rhombohedral distortion is far less pronounced and the vacancy ordering may not be perfect compared with the corresponding phase of Rh_3_*X*_8_ [382]).

**Figure 66.**
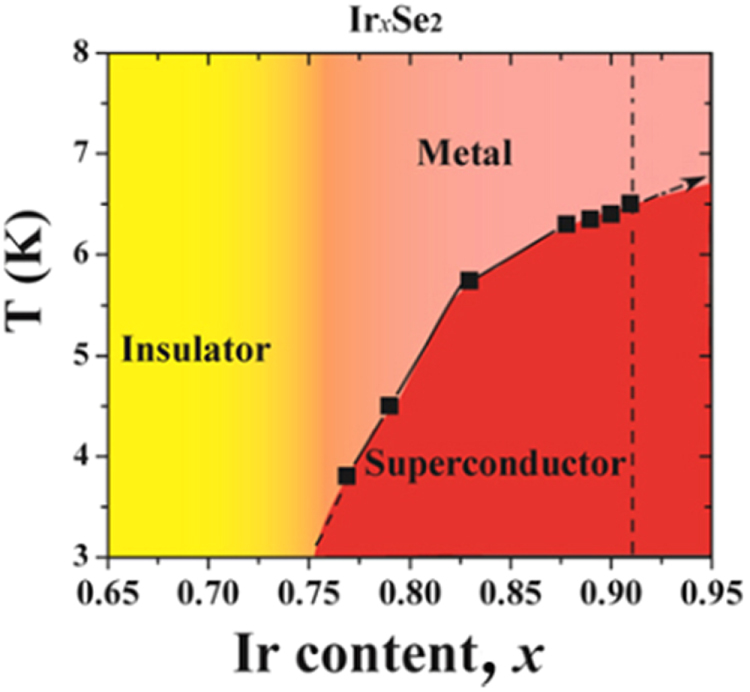
Electronic phase diagram of Ir_*x*_Se_2_. Reprinted with permission from [65]. Copyright 2012 by the American Physical Society.

In the FIRST Project, the pyrite-type Ir_*x*_*X*_2_ samples were synthesized under high pressure and their superconductivity was studied for the first time [65]. Figure 66 gives the electronic phase diagram obtained for the Ir_*x*_Se_2_ system. By increasing *x*, the system changes from insulating to metallic nature and superconductivity starts to emerge at the Ir content of *x* ∼ 0.75 with the highest *T*_c_ of ∼6.4 K at *x* = 0.91 (0.91 is the highest Ir content attained experimentally). The Se–Se distance of the Se dimer increases linearly with increasing *x* as shown in figure 65. A similar phase diagram was obtained for the Ir_*x*_Te_2_ system, with the highest *T*_c_ of ∼4.7 K for *x* = 0.93. DFT calculations were carried out for the vacancy-ordered structural model of Ir_3_Se_8_, where there exist one long Se_1_–Se_1_ dimer (the edge site dimer with *r*_Se1–Se1_ = 2.61 Å) and three short Se_2_–Se_2_ dimers (the body center site dimer with *r*_Se2–Se2_ = 2.50 Å). The DFT calculations revealed that the band crossing the Fermi level consists mainly of the *σ*∗ (anti bonding) orbital of the Se_1_–Se_1_dimer and *d*_z2_ orbitals of the nearest Ir atoms with far less contribution from the *σ*∗ orbitals of the Se_2_–Se_2_ dimers. Such a nature of the electronic structure results in the half-filled narrow conduction band which easily becomes insulating by the electron–electron correlation, electron–lattice interactions and/or the disordered Ir vacancies. DFT calculations were also performed for the vacancy-free pyrite-type structure which is composed of equivalent Se–Se dimers. In this situation, the *σ*∗ orbitals of the Se–Se dimers contribute equally to form a wider conduction band. It should be noted that the Ir vacancy introduction causes a linear increase of the Se–Se distance of the Se dimer in correlation with the monotonous increase of *T*_c_. In the CdI_2_-type IrTe_2_, breaking of the Te–Te dimer is essential for the appearance of superconductivity, while in pyrite-type Ir_x_*X*_2_, control of the bonding state of the *X–X* dimers by elongation and equalization is indispensable for inducing superconductivity. Superconducting parameters obtained for Ir_0.91_Se_2_ are *μ*_0_*H*_c2_(0) = 14.3 T (type-II superconductor), *ξ*_GL_(0) = ∼48Å and *ΔC*(*T*_c_)/*γT*_c_ = 3.1 (strong coupling superconductor).

**Figure 67.**
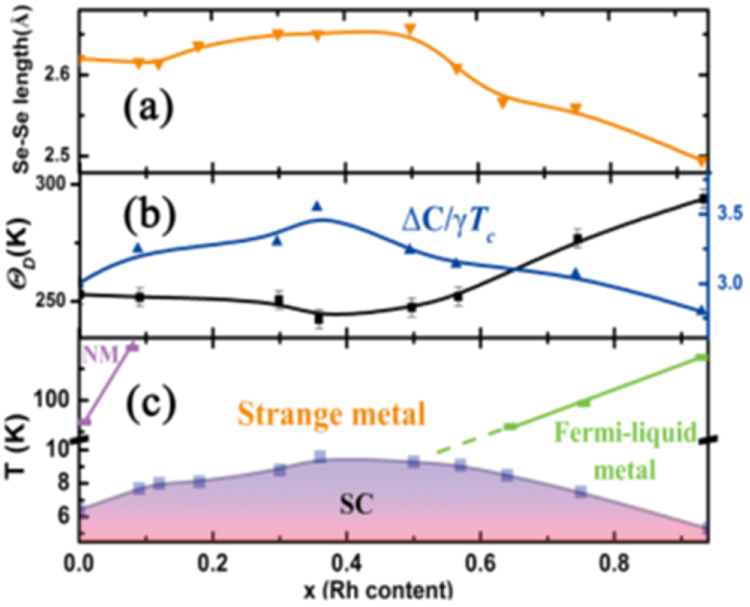
Properties of Ir_0.94–*x*_Rh_*x*_Se_2_. (a) Variation of the Se–Se dimer bond length. (b) Debye temperature *Θ*_D_ and *ΔC*(*T*_*c*_)/*γ*Τ_c_ as a function of the rhodium content *x*. (c) Electronic phase diagram of Ir_0.94–*x*_Rh_*x*_Se_2_. Reprinted with permission from [66]. Copyright 2012 by the American Chemical Society.

The strong correlation between the Se–Se distance and *T*_c_ was also confirmed for the Ir_0.94−*x*_Rh_*x*_Se_2_ system in the FIRST Project [66]. Figure 67 indicates the phase diagram of the Rh-doped system in question. With increasing Rh content, the system undergoes changes from the non-metal state to the normal-metal state with *T*-square resistivity via the strange-metal state with *T*-linear resistivity. Accompanied by this alteration, the Se–Se distance first increases then decreases, taking the maximum at *x* ∼ 0.4. The striking correlation of the Se–Se distance and *T*_c_, *Θ*_D_ and *ΔC*(*T*_c_)/*γT*_c_ is worth noting; both *T*_c_ and *ΔC*(*T*_c_)/*γT*_c_ have maximum values, while *Θ*_D_ has the minimum value when the Se–Se distance is longest. This suggests strengthening of the electron–phonon coupling and softening of phonon due to the structural instability at the edge of weak dimer states when the Rh content is *x* ∼ 0.4.

A similar study has been carried out for the corresponding telluride system of Ir_0.95−*x*_Rh_*x*_Te_2_ [67]. In this system, the Te–Te distance of the Te-dimer shows a parabolic change taking the maximum between *x* = 0.2 and 0.3. Nevertheless, aforementioned correlation was not observed between the Te–Te distance and *T*_c_ nor *ΔC*(*T*_c_)/*γT*_c_; both parameters decrease almost linearly with *x*. This result may be related to the reduced electron–electron repulsion in the telluride system compared with the selenide system, which is caused by the wider width of the conduction band in the telluride system consisting of the more spatially spread Te 5*p* orbital.

#### 3.4.3. Noncentrosymmetric superconductors

Inversion symmetry of a crystal structure is deeply linked to the superconducting state induced in it. Usual superconductors are centrosymmetric with inversion symmetry and, in that case, the state of the Cooper pair can be described by the multiplication of a spin part and an orbital part. In order to satisfy the odd symmetry of the wave function for the exchange of pairing electrons, the spin-singlet state (asymmetric spin part) of the Cooper pair should be combined with the symmetric orbital of the *s*-wave or *d*-wave. In contrast, the spin-triplet (symmetric spin part) state should be combined with the asymmetric *p*-wave. Such a simple picture is not applicable for noncentrosymmetric superconductors; the paring state is no longer classified as singlet or triplet, caused by asymmetric spin–orbit coupling. Theories predicted that mixing of singlet and triplet states (a mixed parity state) occurs for a noncentrosymmetric superconducting system [384–386]. In addition to this striking feature, an anisotropic gap structure with line nodes is predicted for a noncentrosymmetric superconductor [384–386]. The first example of this class of superconductors, CePt_3_Si, was found in 2004 [387] followed by discoveries of a variety of compounds. Among them, Ce-containing heavy-fermion materials with BaNiSn_3_-type structure such as CeCoGe_3_ [388], CeIrSi_3_ [389] and CeRhSi_3_ [390] are worthy of special mention because of their unusually large upper critical fields far beyond the Pauli limit, which seem to be concerned with the mixed parity state.

##### 3.4.3.1. Noncentrosymmetric silicides

A wide variety of silicide superconductors have been known and most of them crystallize in ceontrosymmetric structures showing conventional *s*-wave superconductivity. Some exceptions are the Ce-containing heavy-fermion superconductors described above. In the FIRST Project, we found two new noncentrosymmetric silicide superconductors, SrAuSi_3_ [49] and Li_2_IrSi_3_ [50]. Here, their structural and physical properties are overviewed.

**Figure 68.**
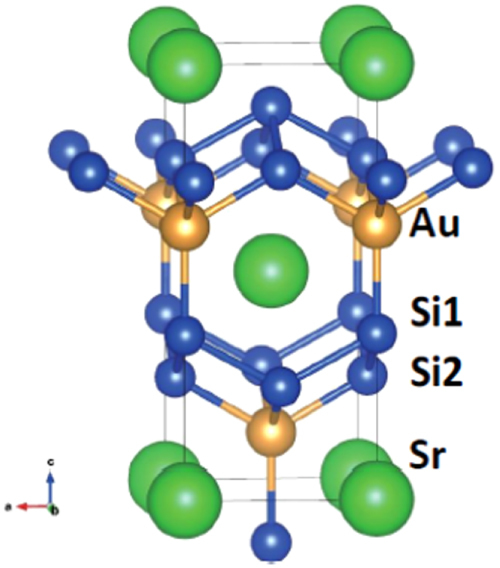
Crystal structure of SrAuSi_3_. Reprinted with permission from [49]. Copyright 2014 by the American Chemical Society.

**Figure 69.**
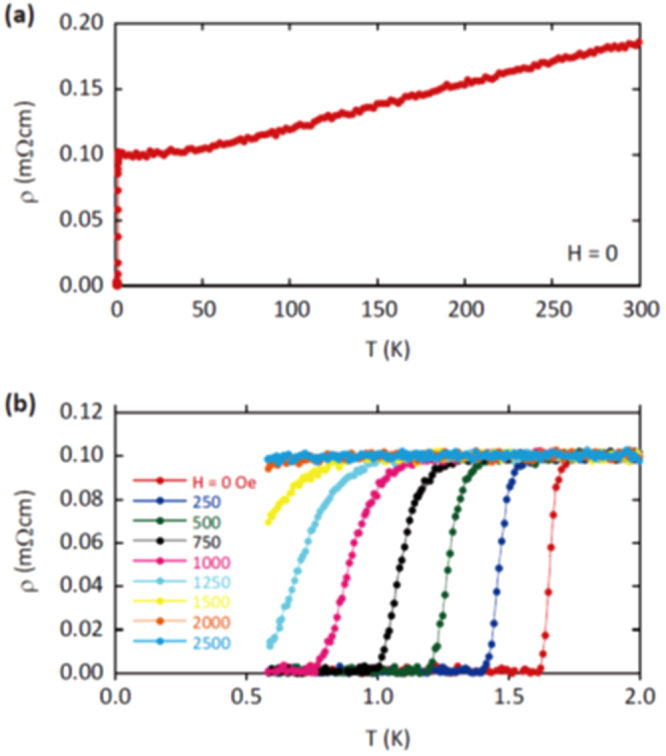
Temperature dependence of the electrical resistivity for a SrAuSi_3_ polycrystalline sample: (a) data taken in the wide temperature range of 0.6–300 K at zero magnetic field (*H* = 0) and (b) data below 2 K at various magnetic field values (*H* = 0–2500 Oe). Reprinted with permission from [49]. Copyright 2014 by the American Chemical Society.

SrAuSi_3_ is the first noncentrosymmetric superconductor containing Au, which is a heavy element and may cause strong spin–orbit coupling. The SrAuSi_3_ is stable only under high pressure and its polycrystalline sample was prepared under high temperature–high pressure conditions [49]. In figure 68, crystal structure of SrAuSi_3_ is shown; it is a BaNiSn_3_-type tetragonal structure in the space group of *I*4*mm*. The structure can be interpreted as sequence of the atom planes along the *c*-axis as Sr–(Au–Si_2_–Si)–Sr–(Au–Si_2_–Si)–Sr… which has a close relationship with the ThCr_2_Si_2_-type and CaBe_2_Ge_2_-type structures (see figure 51). Figure 69 shows the temperature dependence of electrical resistivity measured by varying the magnetic field (*H*). *T*_c_ of SrAuSi_3_ is 1.6 K at *H* = 0 and decreases with increasing magnetic field, giving *H*_c2_(0) ∼ 2.2 kOe which is much lower than the Pauli limit (*H*_p_(0) ∼ 30 kOe). This result suggests that *H*_c2_ is governed by the orbital pair breaking mechanism. Indeed, the orbital limit estimated from the WHH theory is ∼1.5 kOe, comparable with the experimental value. However, *H*_c2_ of SrAuSi_3_ increases almost linearly with temperature deviated substantially from the WHH convex upward curve. This deviation may suggest a nonspherical Fermi surface or gap anisotropy in SrAuSi_3_. The GL coherent length and penetration depth were estimated to be *ξ*_GL_(0) = 390 Å and *λ*(0) = 4400 Å giving the GL parameter *κ*_GL_ = 11 consistent with the type-II nature of superconductivity.

Specific heat data gave *γ* = 6.0 mJ (mol K^2^)^−1^ and *Θ*_D_ = 410 K. The normalized specific heat jump is calculated to be *ΔC*(*T*_c_)/*γT*_c_ = 1.92 and the electron–phonon coupling to be *λ*_ep_ ≈ 0.97 using density of states at the Fermi level, 1.3 states (eV f.u.)^−1^ from the band calculation. These parameters indicate that SrAuSi_3_ is a moderately strong coupling superconductor. *T*_c_ was estimated by the McMillan formula [391], *T*_c_ = (*Θ*_D_/1.45) × exp{–1.04(1 + *λ*_ep_)/[*λ*_ep_ – *μ*∗(1 + 0.62 *λ*_ep_)]}, using the standard value of 0.13 for the Coulomb repulsion parameter *μ*∗, resulting in ∼19 K. The large difference between this value and experimental *T*_c_ of 1.6 K is striking; it may be caused by the parity mixing or the gap anisotropy in the noncentrosymmetric superconductivity. The DFT band calculation for SrAuSi_3_ revealed that two-types of carrier exist on multiple Fermi surfaces. The major carriers conduct in the Si layers while other types of carriers conduct through the 3D network in the structure. Some of the latter carriers looked to have asymmetric spin–orbit coupling, suggesting that they may cause the unusual behaviors in SrAuSi_3_.

**Figure 70.**
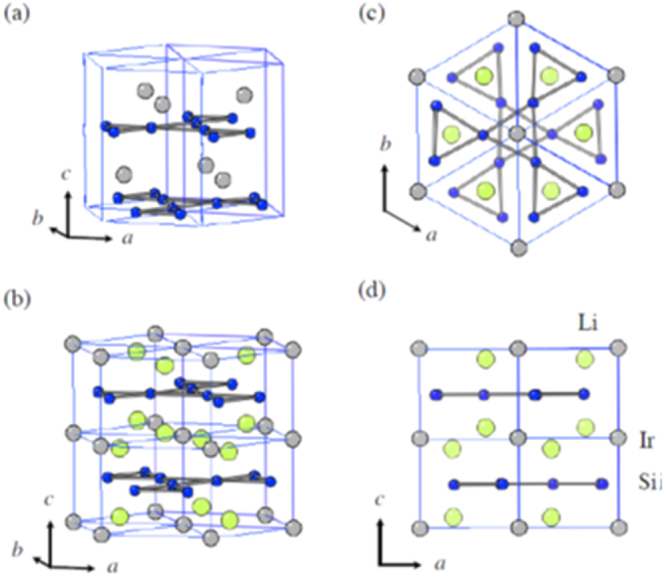
Crystal structures of (a) IrSi_3_ (hexagonal, space group P6_3_mc) and (b) Li_2_IrSi_3_ (trigonal, space group *P*31*c*). Blue circles, yellow circles, and gray circles denote Si, Li, and Ir, respectively. (c) and (d) show the top and side views of the crystal structure of Li_2_IrSi_3_. Reprinted with permission from [50]. Copyright 2014 by the Physical Society of Japan.

IrSi_3_ crystallizes in a hexagonal structure with the space group *P*6_3_*mc* [392] (figure 70) where planar layers of four-fold Si (a distorted kagome network) are stacked along the *c*-axis sandwiching the Ir atoms. The Ir atoms are placed with unequal distances from the upper and lower neighboring Si planes leading to a polar structure with no central symmetry. In the FIRST Project, Li atoms were intercalated to this structure for the first time to obtain Li_2_IrSi_3_ [50]. Li_2_IrSi_3_ has a trigonal structure with the space group of *P*31*c* as given in figure 70 where the essential nature of the Si plane stacking is preserved. In Li_2_IrSi_3_, the positions of Ir and Li are not symmetric concerning the distances from the upper and lower neighboring Si planes, leading to the nonpolar asymmetry. However, displacement from the symmetrical equivalent position is *Δz*/*c* ∼ 0.007, which is one order of magnitude smaller than that of the mother compound of IrSi_3_.

**Figure 71.**
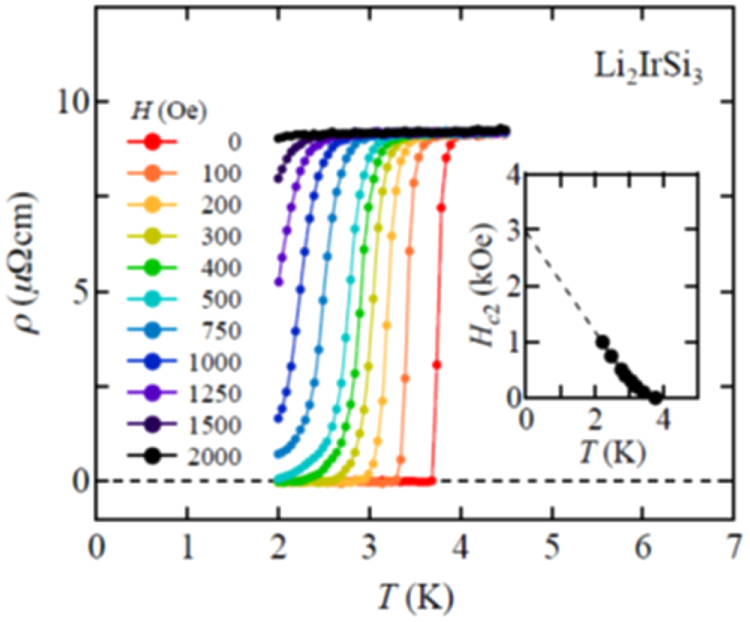
Temperature dependence of electrical resistivity for Li_2_IrSi_3_ at magnetic field *H* up to 2000 Oe. The inset shows the temperature dependence of the upper critical field *H*_c2_. The dashed line represents the linear extrapolation of *H*_c2_(T). Reprinted with permission from [50]. Copyright 2014 by the Physical Society of Japan.

Figure 71 shows resistivity in magnetic field as a function of temperature. Li_2_IrSi_3_ exhibits type-II superconductivity with *T*_c_ of 3.8 K. Temperature dependence of *μ*_0_*H*_c2_ gave *μ*_0_*H*_c2_(0) = 0.3 T and *ξ*_GL_(0) = 330 Å (see the inset in figure 71) while the specific heat data gave *γ* = 5.3 mJ (mol K^2^)^−1^ and *Θ*_D_ = 484 K, which are worth comparing with *γ* = 0.73 mJ (mol K^2^)^−1^ and *Θ*_D_ = 516 K for IrSi_3_. The normalized specific heat jump at *T*_c_ is *ΔC*(*T*_c_)/*γT*_c_ = 1.41, consistent with the BCS weak coupling limit. All these experimental data suggest the conventional nature of superconductivity in Li_2_IrSi_3_; in particular, *μ*_0_*H*_c2_(0) (0.3 T) is much lower than the Pauli limit of *μ*_0_*H*_P_(0) = 6.9 T. The weakened inversion symmetry breaking seems to account for the less noticeable unique nature of superconductivity. The appearance of superconductivity in Li_2_IrSi_3_ may be concerned with the enhancement of *γ*, i.e., a seven-fold increase of the electronic DOS at the Fermi level after the intercalation of Li for IrSi_3_.

##### 3.4.3.2. Ternary equiatomic pnictides, LaMPn (M=Ir, Rh; Pn=P, As)

**Figure 72.**
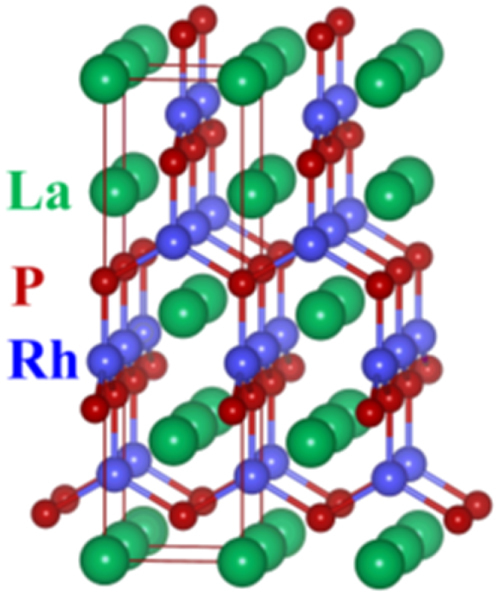
Crystal structure of LaRhP. Reprinted with permission from [51]. Copyright 2014 by the American Physical Society.

The ternary equiatomic pnictides with the general formula, *M’MPn* (*M’* is a large sized electropositive transition metal, *M* is a smaller transition metal, *Pn* is a pnictogen) form a large family of compounds. In the FIRST Project, the equiatomic pnictides with 4*d* and 5*d* transition metals for the *M* site were studied because previous studies have been rather confined to the systems with 3*d* transition metals for *M*. Consequently, type-II superconducting transitions were observed for the first time in LaIrP, LaIrAs and LaRhP, which were prepared using the high-pressure synthesis technique [51]. Lattice parameters for LaRhAs and LaIrP were in good agreement with previous reports [393, 394]. In figure 72, the crystal structure is shown for LaRhP, which has a tetragonal lattice with the space group of *I*4_1_*md* and is an ordered ternary derivative of the *α*-ThSi_2_-type structure. In the structure, the Rh and P atoms are linked forming a 3D network with a trigonal planar coordination, and the La atoms are placed in the cavities of the network. There are two sets of Rh–P zigzag chains running toward the perpendicular directions.

In table 13, superconducting parameters and the lattice parameters are shown for LaIrP, LaIrAs and LaRhP. Among them, LaIrP has the highest *T*_c_ of 5.3 K and the highest *H*_c2_(0) of 13.8 kOe (WHH value) or 16.4 kOe (GL value) corresponding to the highest value of electron–phonon coupling, *λ*_ep_ = 0.67. The *ΔC*(*T*_c_)/*γT*_c_ values are less than unity for all three compounds indicating the weak electron–phonon coupling regime. For every compound, the experimental *H*_c2_(0) is much lower than the Pauli limit of field, suggesting that the asymmetric spin–orbital coupling induced by the lack of central symmetry is not significant in the La*MPn* system.

**Table 13.** Superconducting and lattice parameters for the LaMX compounds [51].

Compound	LaIrP	LaIrAs	LaRhP
Superconducting parameters			
T_C_(K)	5.3	3.1	2.5
H_C2_(0) (WHH) (kOe)	13.8	5.5	2.1
H_C2_(0) (GL) (kOe)	16.4	6.4	2.7
ξ_GL_(0) (Å)	14.2	22.7	34.9
*Δ*C(T_C_)/*γ*T_C_	0.65	0.84	0.73
*λ*_ep_	0.67	0.58	0.52
Lattice parameters (tetragonal I4_1_md)			
a (Å)	4.2065	4.1505	4.1846
b (Å)	14.9379	14.3277	14.9358

#### 3.4.4. Miscellaneous non-iron superconductors

Besides the superconductors described in the preceding sections, various new non-iron superconductors were discovered through the FIRST Project: they include NbSiAs (*T*_c_ = 8.2 K) [30], CeNi_0.8_Bi_2_ (*T*_c_ = 4 K) [37], LaNiBN (*T*_c_ = 4.1 K), LaPtBN (*T*_c_ = 6.7 K), La_3_Ni_2_B_2_N_3_ (*T*_c_ = 15 K) [38], La_2_Sb (*T*_c_ = 5.3 K) [43], Ba_*n*+2_Ir_4*n*_Ge_12*n*+4_ (*T*_c_ = 6.1 K for *n* = 1; *T*_c_ = 3.2 K for *n* = 2) [73], Nb_4_NiSi (*T*_c_ = 7.7 K) [72], and Ca_2_InN (*T*_c_ = 0.6 K) [74].

## 4. Thin films and wires of iron-based superconductors

### 4.1. Epitaxial thin film deposition

#### 4.1.1. Deposition of Ba(Fe,Co)_2_As_2_ epitaxial films

Fabrication of high-quality epitaxial films is essential for the study of new superconductors such as IBSCs, from the viewpoints not only of the investigation of their anisotropic physical properties but also their application to superconducting wires or tapes as well as electronic devices. Among a variety of IBSCs, the 122 type compounds have been expected to be the most promising candidates for wire application, because of their anisotropy which is substantially smaller than that of the 1111 compounds and comparable to that of MgB_2_ [395]. For thin-film growth, carrier doping by the substitution of Co or P seems easier than F doping in the 1111 compounds.

Actually, the first superconducting epitaxial films of the IBSCs were realized in the 122 type compound Sr(Fe,Co)_2_As_2_ (Sr-122:Co) films with a 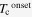 and 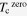 of approximately 20 and 15 K on LSAT (001) single-crystal substrates by Hiramatsu *et al* of Tokyo Institute of Technology (Tokyo Tech) [396]. They employed pulsed laser deposition (PLD) using a second-harmonic neodymium-doped yttrium aluminum garnet (Nd:YAG) laser (*λ* = 532 nm). Later Katase *et al* succeeded in fabricating Co-doped Ba-122 (Ba-122:Co) epitaxial films on LSAT substrates by using the same PLD technique and demonstrated that they had much higher stability against water vapor than Sr-122 films [397]. However, their rather large resistive transition width (*ΔT*_c_) of approximately 3 K and low critical current density (*J*_c_) at 5 K of 10^5^ A cm^−2^ indicated that their film quality was not good enough to apply them to electronic devices and superconducting tapes.

**Figure 73.**
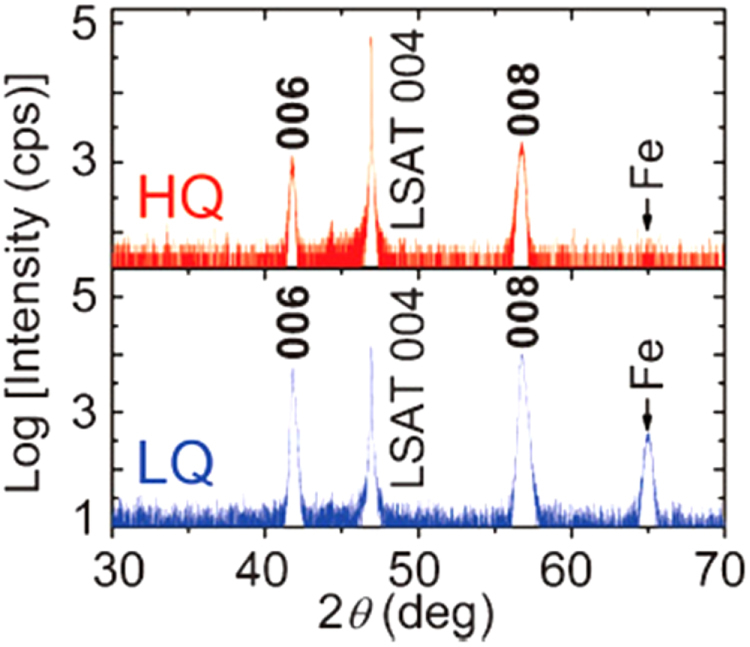
X-ray diffraction patterns for high-quality (HQ) and low-quality (LQ) Ba(Fe,Co)_2_As_2_ (Ba-122:Co) epitaxial films on LSAT substrates fabricated by PLD. High-quality films were obtained by improving the purity of targets and homogeneity of substrate temperature. Reprinted with permission from [398]. Copyright 2010 by the Japan Society of Applied Physics.

**Figure 74.**
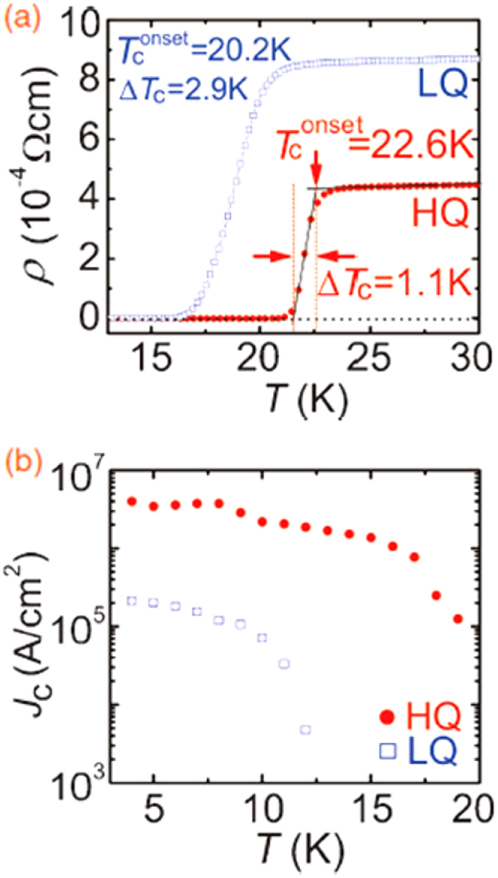
Temperature dependences of (a) resistivity and (b) *J*_c_ for high-quality (HQ) and lower-quality (LQ) Ba-122:Co epitaxial thin films on LSAT substrates. Reprinted with permission from [398]. Copyright 2010 by the Japan Society of Applied Physics.

In order to obtain higher-quality Ba-122:Co epitaxial films, Katase *et al* of Tokyo Tech Hosono group tried improving the purity of the Ba-122:Co target and homogeneity of substrate temperature [398]. A key point to obtain a high-purity Ba-122:Co target was use of fine Ba metal pieces to synthesize the BaAs precursor. Figure 73 shows x-ray diffraction patterns for Ba-122:Co films on LSAT obtained before and after such improvement. The diffraction peak from Fe impurities observed in the previous film almost disappears in the improved film. The in-plane alignment of the 122 grains was also found to be improved. The improved Ba-122:Co film exhibited a substantially higher 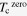 above 20 K, narrower transition width of about 1 K, and a higher self-field *J*_c_ up to 4 MA cm^−2^ at 4 K, as shown in figure 74.

They also comprehensively studied the deposition conditions for Ba(Sr)122 thin films [238] and found that Ba-122 epitaxial films could be grown in a wider temperature range of 700–900 °C than that for Sr-122. In particular, Ba-122 epitaxial films with a two-dimensional growth mode, high crystallinity, and a high self-field *J*_c_ at 4 K above 1 MA cm^−2^ could be obtained in a temperature range of 800–850 °C at a growth rate between 0.28 and 0.33 nm s^−1^. Investigation of the Co content dependence of the transport properties for Ba(Fe_1−*x*_Co_*x*_)_2_As_2_ (Ba-122:Co) epitaxial films revealed that the highest *T*_c_ of approximately 25.5 K was obtained for *x* = 0.075 [238]. They also demonstrated that Ba-122:Co epitaxial films with a self-field *J*_c_ above 1 MA cm^−2^ could be directly prepared on MgO (100) single-crystal substrates without using a conducting buffer layer, such as the Fe buffer reported by Iida *et al* [399].

**Figure 75.**
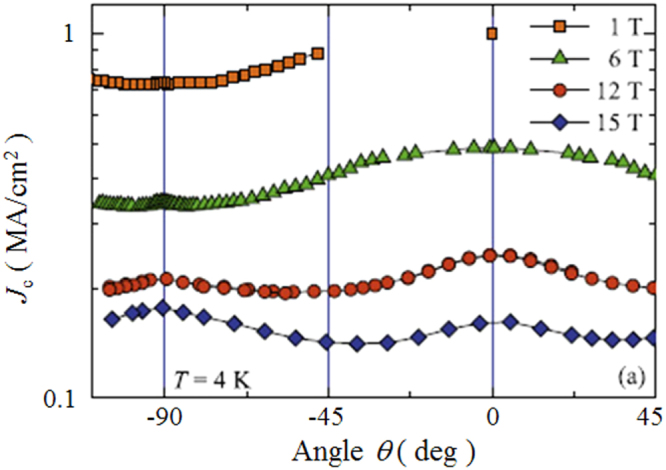
Angular dependence of *J*_c_ at 4 K in fields of 1–15 T for a high-quality Ba-122:Co epitaxial film on LSAT. Reprinted with permission from [400]. Copyright 2011 by IOP Publishing.

Figure 75 shows the field angular dependence of *J*_c_ for a high-quality Ba-122:Co epitaxial film on LSAT [400]. A broad *J*_c_ peak around the *c*-axis direction is observed at 6 T. This *c*-axis peak becomes less prominent at higher fields but still exists even at a high field of 15 T. This result suggested that naturally formed defects along the *c*-axis such as dislocations work as rather strong pinning centers in the Ba-122:Co epitaxial film on LSAT. This is quite different from the result reported for the Ba-122:Co epitaxial films on a Fe buffer layer where no *c*-axis peak was observed [401].

#### 4.1.2. Deposition of BaFe_2_(As,P)_2_ epitaxial films

##### 4.1.2.1. Superconducting properties of BaFe_2_(As_0.6_P_0.4_)_2_ epitaxial films

As described in the previous section, the 122 compounds seem the most suitable for application to superconducting wires or tapes among IBSCs. The 122 compounds exhibit superconductivity by substituting Co for the Fe site, K for the Ba(Sr) site, or P for the As site. K-doped Ba(Sr)122 has the highest *T*_c_ and upper critical field (*H*_c2_) as summarized in table 14. Although synthesis of K-doped Ba-122 (Ba-122:K) epitaxial films by a molecular beam epitaxy (MBE) method was reported [402], the existence of volatile K makes it difficult to fabricate its films by a PLD method. It was also reported that the films were not stable in ambient atmosphere [402]. P-doped Ba-122 (Ba-122:P) has a higher *T*_c_ than Ba-122:Co of approximately 30 K [403] and is expected to be stable in ambient atmosphere. Adachi *et al* of ISTEC group chose this compound as a material candidate for production of superconducting tapes by a PLD method using a second-harmonic Nd:YAG laser, and examined the film preparation conditions on MgO single crystal substrates [404].

**Table 14.** Superconducting properties of representative iron-based superconductors.

Material	*T*_c_ (K)	*μ*_0_*H*_c2_^//c^(0)^∗^ (T)	*γ*_H_
*R*FeAs(O, F) (*R*=Nd, Sm)	47–55	80–100	5–10
(Ba, K)Fe_2_As_2_	37–38	70–135	1.5–2
Ba(Fe, Co)_2_As_2_	22–25	47–50	1.5–1.9
BaFe_2_(As, P)_2_	30–31	~60	1.5–1.9
Fe(Se, Te)	14–16	~50	1.1–1.9

**Figure 76.**
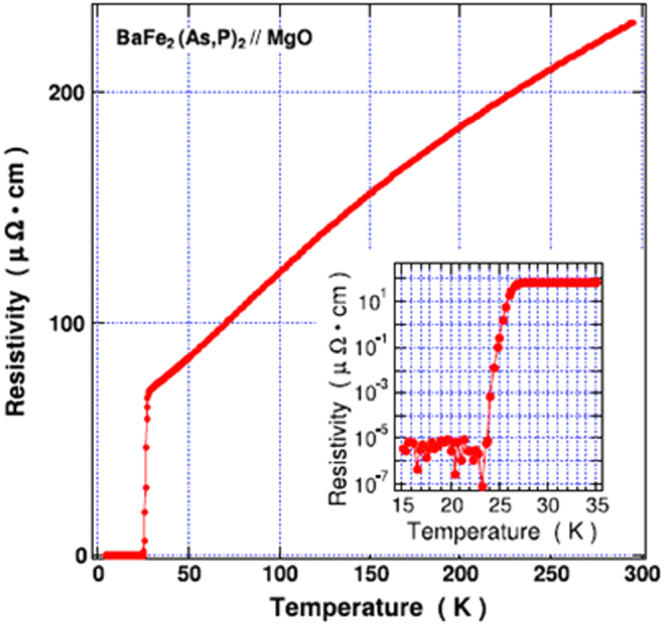
Temperature dependence of resistivity for a BaFe_2_(As_0.6_P_0.4_)_2_ (Ba-122:P) epitaxial film on MgO substrate fabricated by PLD. The inset magnifies the curve near *T*_*c*_. Reprinted with permission from [404]. Copyright 2012 by IOP Publishing.

**Figure 77.**
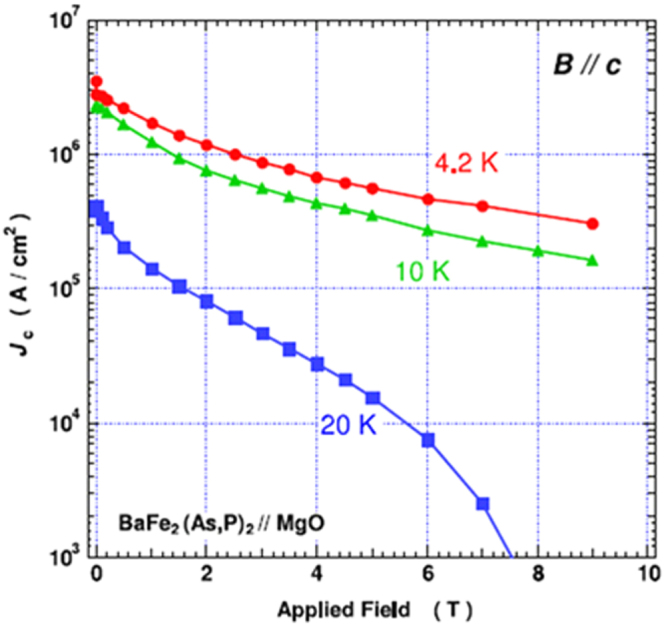
Magnetic field dependence of *J*_c_ at 4.2, 10, and 20 K for a Ba-122:P epitaxial film on MgO substrate fabricated by a PLD method. Magnetic field was applied parallel to the *c*-axis. Reprinted with permission from [404]. Copyright 2012 by IOP Publishing.

High-purity targets with a nominal composition of BaFe_2_(As_0.6_P_0.4_)_2_ were carefully synthesized by a conventional solid-state reaction method. Epitaxial films were obtained at a substrate temperature of approximately 800 °C. The energy density on the target was relatively high, approximately 10 J cm^−2^, leading to a deposition rate of 5 nm s^−1^ at the repetition rate of 10 Hz and the substrate–target distance of 7 cm. The average FWHM value of the peaks in the *ϕ*-scan was about 1.5°. The obtained film exhibited a 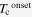 and 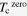 of 26.5 and 24.0 K, as seen in the resistive transition curve of figure 76. An even higher *T*_c_ of 27.0 K was also observed for a film without patterning. The first synthesis of Ba-122:P epitaxial films was previously achieved by an MBE method [405]. The observed *T*_c_ values of the PLD films are comparable to those reported for the MBE films [405] and the single crystals [403]. Figure 77 shows the dependence of *J*_c_ on the applied field along the *c*-axis at different temperatures for the Ba-122:P epitaxial film with the 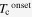 of 26.5 K and a self-field *J*_c_ value at 4.2 K of 3.5 MA cm^−2^. The film exhibited rather high in-field *J*_c_ values, for example, approximately 1 MA cm^−2^ at 4.2 K, 3 T and 10 K, 1 T, which are higher than those for Ba-122:Co films [406].

##### 4.1.2.2. Superconducting properties of BaFe_2_(As_1−x_P_x_)_2_ epitaxial films

**Figure 78.**
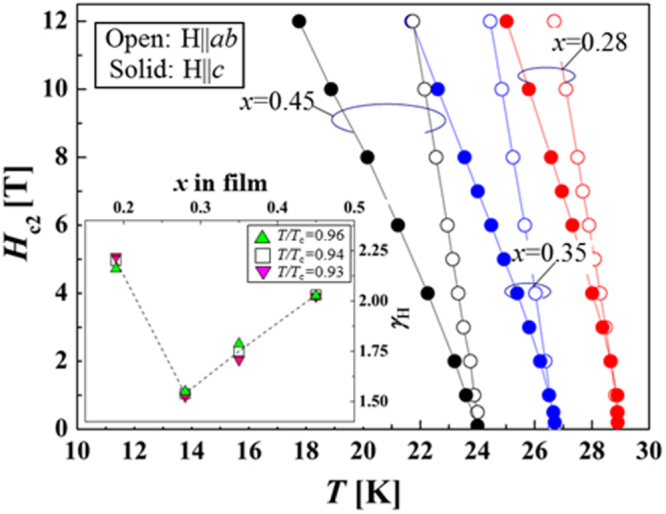
Temperature dependence of upper critical fields (*H*_c2_) for Ba-122:P epitaxial films on MgO with analyzed P content *x* of 0.28, 0.35, and 0.45. The inset shows the *x* dependence of anisotropy parameter *γ*_H_ at *T*/*T*_*c*_ = 0.93, 0.94, and 0.96. Reprinted with permission from [407]. Copyright 2013 by the Japan Society of Applied Physics.

**Figure 79.**
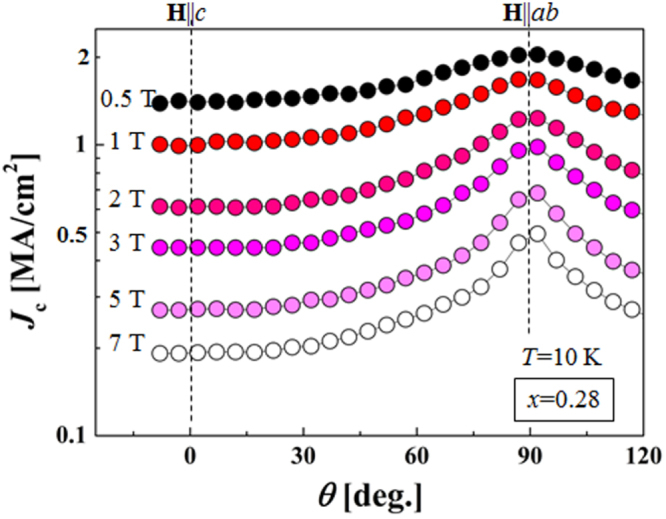
Angular dependences of in-field *J*_c_ at 10 K in fields of 0.5–7 T for the Ba-122:P film with *x* = 0.28. Reprinted with permission from [407]. Copyright 2013 by the Japan Society of Applied Physics.

The dependence of superconducting properties of BaFe_2_(As_1−*x*_P_*x*_)_2_ (Ba-122:P) epitaxial films on the P content was systematically investigated by Miura *et al* by using Ba-122:P targets with nominal P content *x* of 0.25, 0.33, 0.40 and 0.50 [407]. The *x* values in the films analyzed using an electron probe micro analyzer (EPMA) were found to be slightly smaller by approximately 0.05 than the nominal *x* values in bulk targets. Figure 78 shows the temperature dependence of *H*_c2_ for Ba-122:P films with various analyzed *x* values in magnetic fields up to 12 T. The *x* = 0.28 film exhibits a maximum 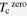 of 26.5 K, and a further increase in *x* leads to a reduction in 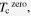 while the *x* = 0.19 film showed a broad transition and 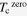 of about 12 K. This *x* dependence of *T*_c_ is similar to that observed in single crystals [403]. The *x* = 0.28 film also exhibits the highest *H*_c2_ in both field directions. The inset shows the anisotropy of *H*_c2_, *γ*_H_ = 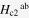/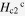 for the films. The *x* = 0.28 film with the optimal *T*_c_ shows the smallest *γ*_H_ value of 1.54. This is different from the case of cuprate superconductors [408], and preferable from the viewpoint of wire or tape application. Figure 79 shows the angular dependence of *J*_c_ for the *x* = 0.28 film at 10 K under different magnetic fields. This film also exhibits high in-field *J*_c_ values. It is also found that *J*_c_ shows minimum values in the *c*-axis direction at all fields from 0.5 to 7 T, indicating that no *c*-axis-correlated pinning centers are included in this film.

#### 4.1.3. BaFe_2_(As,P)_2_ films with strong pinning centers

##### 4.1.3.1. Addition of artificial pinning centers consisting of BaZrO_3_ nanoparticles

The Ba-122:P epitaxial films on MgO substrates fabricated by a PLD method showed relatively high in-field *J*_c_ values. However, these values do not seem to be high enough for high-field application. Since the Ba-122 superconductors have very high *H*_c2_(0) values over 60 T, a remarkable improvement in in-field *J*_c_ would be expected, if effective vortex pinning centers could be introduced into the materials. Actually, amorphous tracks induced in Ba-122:K single crystals by heavy-ion irradiation significantly enhanced their in-field *J*_c_ and resulted in a matching field as high as 21 T without *T*_c_ degradation [409], although heavy-ion irradiation is not practical for fabrication of long wires or tapes. For the case of Ba-122 epitaxial films, naturally formed defects in Ba-122:Co films enhanced the *J*_c_ in the fields parallel to the *c*-axis to some extent [400], as described in the previous section. It was also reported that naturally formed nanopillars along the *c*-axis in the Ba-122:Co thin films prepared by PLD using a SrTiO_3_ buffer layer and oxygen-rich targets significantly enhanced the in-field *J*_c_ around the *c*-axis direction [410, 411]. However, the *J*_c_ around the *ab* plane direction was not much improved.

For application to superconducting tapes, it is desirable to find a controllable and practical way of enhancing vortex pinning in Ba-122 films in an isotropic way. In cuprate superconductor films such as *RE*Ba_2_Cu_3_O_y_ (REBCO; *RE* = Y or rare-earth elements) films, the addition of nanoparticles consisting of second oxide phases has been found to be effective in enhancing their in-field *J*_c_ in an isotropic way [412–414]. However, in order to apply a similar technique to Ba-122 epitaxial films, the second phases need to be chemically stable and crystallographically compatible with the Ba-122 phase, and its size should be small enough to avoid blocking of the current path.

**Figure 80.**
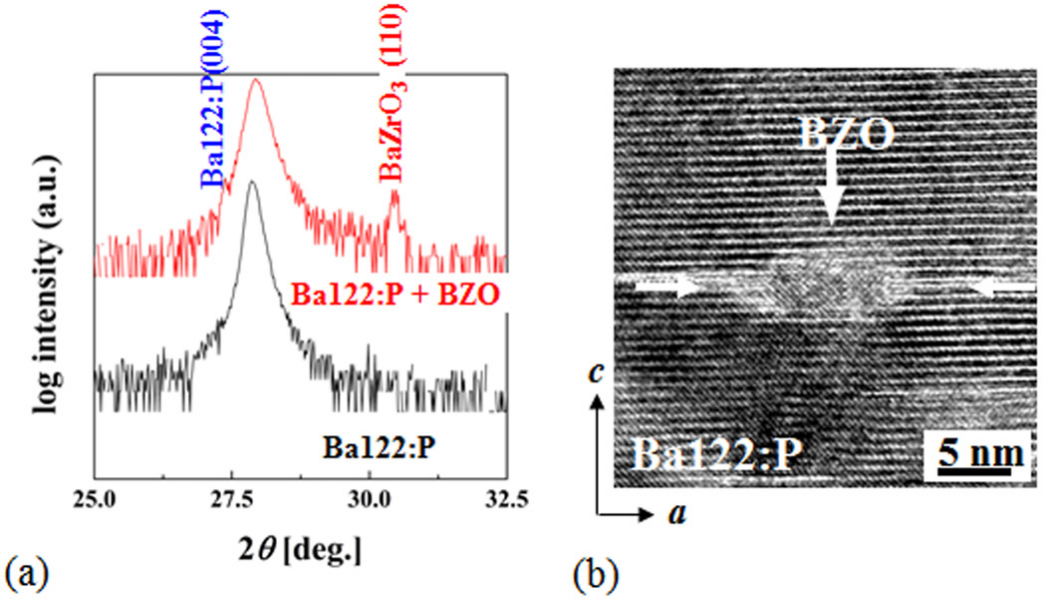
(a) X-ray diffraction patterns for a Ba-122:P and a Ba-122:P + 3 mol% BaZrO_3_(BZO) film prepared on MgO substrates. (b) Cross-sectional high-resolution TEM (HRTEM) image for a BZO nanoparticle in the Ba-122:P + 3 mol% BZO film. Reprinted with permission from Macmillan Publishers Ltd: [415], Copyright 2013.

Miura *et al* tried introducing BaZrO_3_ (BZO) nanoparticles, which are known to be effective pinning centers in REBCO films, into Ba-122:P epitaxial films on MgO by a PLD method [415]. Approximately 80 nm thick BZO-doped Ba-122:P epitaxial films were grown from 1 mol.% and 3 mol.% BZO-doped Ba-122:P targets with the nominal P content of 0.33. The deposition conditions were similar to those employed by Adachi *et al* for the synthesis of undoped Ba-122:P films [404]. The cross-sectional elemental maps and x-ray diffraction pattern shown in figure 80(a) of the film grown from the 3 mol.% BZO-doped target indicated the presence of homogenously dispersed BZO nanoparticles. The average nanoparticle size and the average spacing were found to be 8 nm and 24 nm, respectively, leading to a density *n* of approximately 6.8 × 10^22^ m^−3^. The cross-sectional TEM image of a typical BZO nanoparticle is shown in figure 80(b). The size of the nanoparticle is about 5 and 10 nm parallel and perpendicular to the *c*-axis, respectively. The periodicity of the Ba-122 planes around the nanoparticles is only perturbed by the creation of stacking faults. However, nano-beam diffraction patterns for the nanoparticle and the Ba-122:P matrix indicated that the BZO nanoparticles are not epitaxially oriented along the Ba-122:P matrix. The 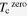 values for the undoped and doped films are 26.3 and 25.0 K, respectively, indicating that introduction of the BZO nanoparticles does not induce significant *T*_c_ degradation.

**Figure 81.**
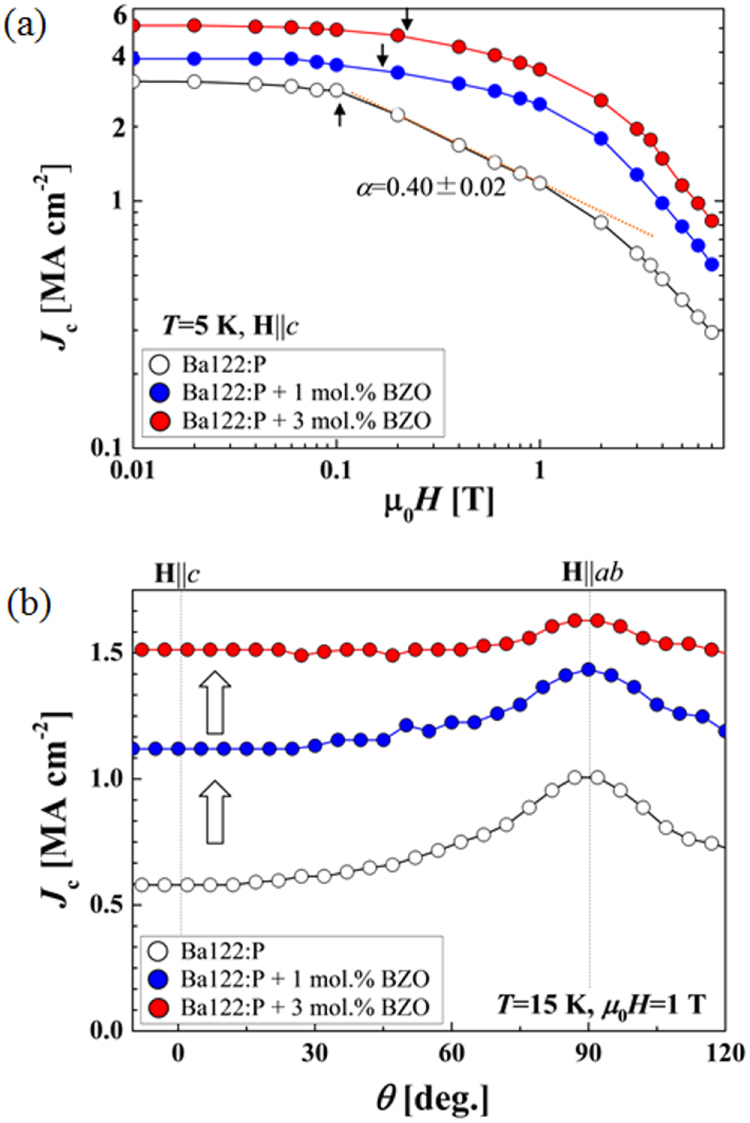
(a) Magnetic field dependence of *J*_c_ at 5 K for Ba-122:P, Ba-122:P + 1 mol% BZO and Ba-122:P + 3 mol% BZO films on MgO. A magnetic field was applied parallel to the *c*-axis. (b) The angular dependence of in-field *J*_c_ at 15 K, 1 T for the Ba-122:P, Ba-122:P + 1 mol% BZO and Ba-122:P + 3 mol% BZO films. Reprinted with permission from Macmillan Publishers Ltd: [415], Copyright 2013.

Figure 81(a) shows the field dependence of *J*_c_ (*H*//*c*) at 5 K for the undoped Ba-122:P and BZO added Ba-122:P films. The self-field *J*_c_ (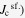 increases monotonically with the amount of BZO additive and the field decay of *J*_c_ is greatly reduced within the measured field range. The Ba-122:P film shows a characteristic crossover field *H*∗ (90% of *J*_c_(*H*)/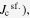 which is indicated by the arrows, followed by a power-law regime (*J*_c_ ∝ *H*^−*α*^) with *α* ∼ 0.40 at intermediate fields. A more rapid decay of *J*_c_ is observed as *H* approaches the irreversibility field, *H*_irr_. For the Ba-122:P + BZO films, *H*∗ also increases with the BZO content. At intermediate fields, we find that both films with BZO show a slower decay of *J*_c_(*H*), indicating the importance of BZO nanoparticles to enhance *J*_c_(*H*) in magnetic fields. The non-power-law dependence observed for the Ba-122:P + BZO films is similar to that observed in RE123 films with strong pinning coming from uniformly dispersed nanoparticles [413, 414, 416].

Figure 81(b) shows the angular dependence of *J*_c_, *J*_c_(*θ*), curves measured for the three films at 1 T, 15 K. By adding BZO the *J*_c_ increases for all orientations with respect to the *J*_c_ of the Ba-122:P film. In particular, the Ba-122:P + 3 mol% BZO film exhibits an almost isotropic *J*_c_ with the value for *H*//*c* 2.6 times higher than that of the Ba-122:P film. The minimum value of *J*_c_(*θ*), *J*_c,min_, of 1.5 MA cm^−2^ at 1 T, 15 K is over 28 times and 7 times higher than that of Ba-122:Co films with *c*-axis columnar defects [410] and Ba-122:Co films with super-lattice structures [417], respectively, in very similar field and temperature conditions, indicating strong isotropic pinning by the BZO nanoparticles.

**Figure 82.**
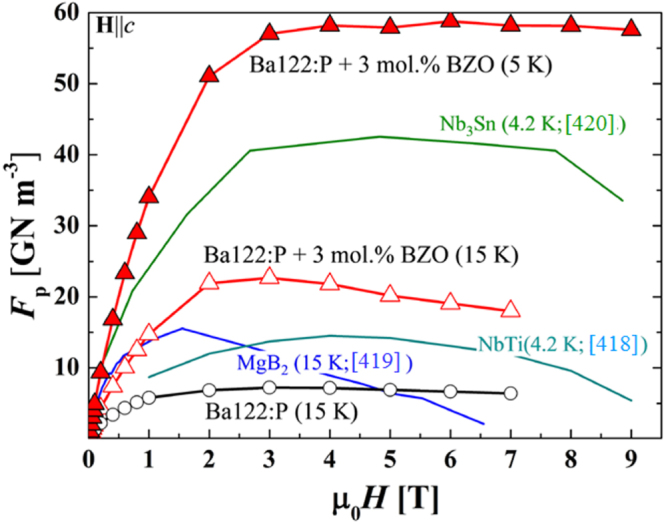
Pinning force *F*_p_ at magnetic fields applied parallel to the *c*-axis for the Ba-122:P + 3 mol% BZO film at 5 K and 15 K and the Ba-122:P film at 15 K. For comparison, the data for NbTi at 4.2 K [418], MgB_2_ (*H//ab*) at 15 K [419], Nb_3_Sn at 4.2 K [420] are included. Reprinted with permission from Macmillan Publishers Ltd: [415], Copyright 2013.

In figure 82, the pinning force, *F*_p_ = *J*_c_(*H*) × *μ*_0_*H* is compared with that of several superconductor materials. At 15 K, *F*_p_ for the Ba-122:P + 3 mol% BZO film is over 3 times higher than that for the Ba-122:P film and higher than NbTi [418] at 4.2 K at all magnetic fields. Comparing with MgB_2_ data at 15 K [419], *F*_p_ is clearly higher for *μ*_0_*H* > 0.5 T. At *T* = 5 K, the *F*_p_ of the Ba-122:P + 3 mol.% BZO film, which is the minimum value in all field directions, reaches ∼59 GN m^−3^ for *μ*_0_*H* > 3 T up to the highest field we measured (9 T), a 50% increase over Nb_3_Sn at 4.2 K [420].

Miura *et al* also found that the characteristic magnetic field, where the maximum relative *J*_c_ enhancement by BZO addition was observed, increased monotonically by increasing the nanoparticle density. From the comparison with the previous results of REBCO films with BZO nanoparticles [421], it was deduced that the effective way to optimize the *J*_c_ performance of IBSCs as well as cuprates is dispersing nanoparticles with the average size smaller than ∼3∗(2*ξ*(*T*)) and with densities such that the nanoparticle spacing matches the intervortex distance. These results suggested the possibility of further enhancing in-field *J*_c_ properties, in particular at higher fields, by optimizing the landscape of nanoparticles or nanoscale defects in Ba-122:P epitaxial films.

##### 4.1.3.2. Intentionally grown c-axis pinning centers

Sato *et al* carefully investigated the properties of Ba-122:P epitaxial films on MgO (001) single-crystal substrates prepared by PLD using the second harmonic of an Nd:YAG laser and found that films with very high and less anisotropic in-field *J*_c_ could be obtained at certain deposition conditions [422]. They employed a semiconductor infrared diode (*λ* = 975 nm, and maximum power = 300 W) for substrate heating and achieved high substrate temperature (*T*_s_) up to 1400 °C. High-purity Ba-122:P targets with the nominal P content *x* of 0.30 were used. It was found that 150–200 nm thick epitaxial films with high crystallinity (*Δϕ* and *Δω* well below 0.8°) were grown at rather high *T*_s_ of 1000–1100 °C (optimum at 1050 °C) and low growth rate of 0.2–0.4 nm s^−1^ for laser fluence of 3.0–3.5 J cm^−2^. Their lattice parameters slightly different from the single crystal data [403] indicated existence of tensile strain in the films. The optimum film exhibited a *T*_c_ of 26.5 K, a narrow transition width *ΔT*_c_ of 1.5 K, and a very high self-field *J*_c_ up to 7 MA cm^−2^. This *J*_c_ value is comparable to the recently reported high value for a film grown by an MBE method [423].

**Figure 83.**
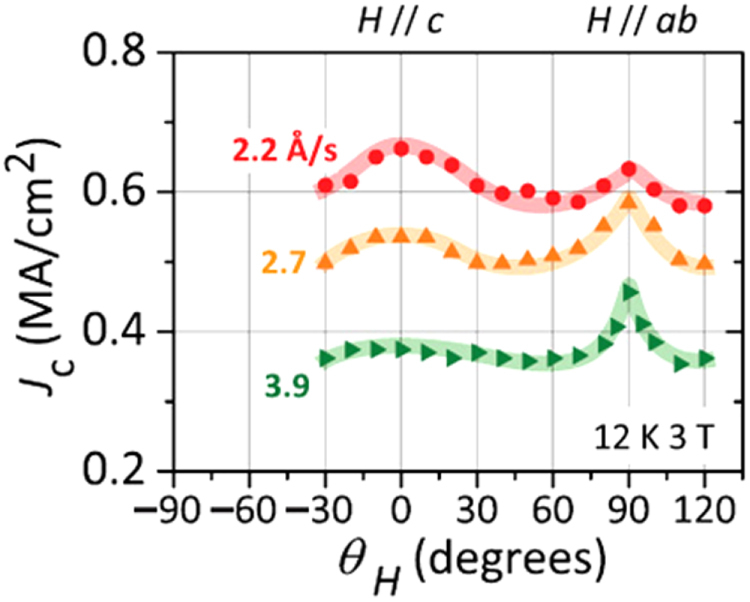
Angular dependence of in-field *J*_c_ at 12 K, 3 T for high-quality Ba-122:P epitaxial films grown on MgO by PLD at relatively high substrate temperatures and low deposition rates of 0.22, 0.27, and 0.39 nm s^−1^. Reprinted with permission from [422]. Copyright 2014 by AIP Publishing LLC.

Figure 83 shows the angular dependence of *J*_c_ at 12 K, 3 T for Ba-122:P epitaxial films grown at the optimum *T*_s_. The *J*_c_(*θ*_Η_) curves exhibit a broad peak around the *c*-axis direction (*θ*_Η_ = 0°) in addition to the intrinsic *J*_c_ peak at *θ*_Η_ = 90°, indicating existence of pinning centers along the *c*-axis. With decreasing growth rate from 0.39 nm s^−1^ to 0.22 nm s^−1^, the *θ*_Η_ = 0° peak becomes more prominent and the *J*_c_ values in all directions become remarkably higher, resulting in less anisotropic angular dependence. These results indicate that the vortex pinning properties and *J*_c_ anisotropy of Ba-122:P epitaxial films can be controlled by tuning the growth rate.

**Figure 84.**
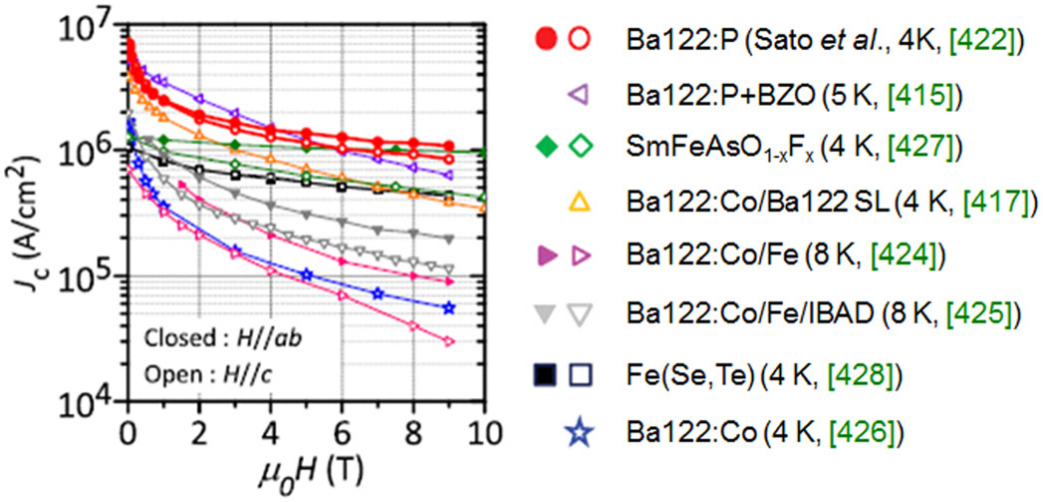
Comparison of magnetic field dependence of *J*_c_ for the optimum Ba-122:P epitaxial film with those reported for other Ba-122 films as well as SmFeAsO_1−*x*_F_*x*_ and Fe(Se,Te) films with high *J*_c_ values. The closed and open symbols represent *J*_c_ for fields parallel to the *a*,*b*-axes and *c*-axis, respectively. Reprinted with permission from [422]. Copyright 2014 by AIP Publishing LLC.

In figure 84, the magnetic field dependence of 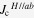 (closed symbols) and 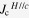 (open symbols) of the optimum Ba-122:P epitaxial films is compared with those reported for other Ba-122 epitaxial films with high *J*_c_ [415, 417, 424–426] as well as SmFeAsO_1−*x*_F_*x*_ and Fe(Se,Te) films [427, 428]. The optimum Ba-122:P film exhibits an even higher 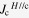 than the Ba-122:P film with BZO nanoparticles at *H* > 4 T and a 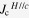 as high as 0.8 MA cm^−2^ at 4 K, 9 T, resulting in the highest pinning force of 72 GN m^−3^. It would also be the noteworthy that the 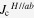 value of 1.1 MA cm^−2^ at 9 T giving a pinning force of 99 GN m^−3^ is the highest obtained for IBSC films.

**Figure 85.**
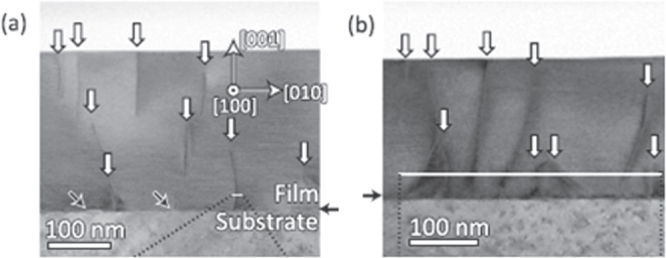
Cross-sectional bright-field STEM images for Ba-122:P epitaxial films grown at the growth rate of (a) 0.22 nm s^−1^ and (b) 0.39 nm s^−1^. The arrows indicate the positions of vertical defects which are considered as strong pinning centers. Reprinted with permission from [422]. Copyright 2014 by AIP Publishing LLC.

Figure 85 shows the cross-sectional bright-field scanning TEM (STEM) images for Ba-122:P epitaxial films grown at the growth rate of 0.22 nm s^−1^ and 0.39 nm s^−1^. As indicated by the vertical white arrows in figure 85, there are many vertical defects with a substantially higher density than that observed in the Ba-122:Co epitaxial films by Katase *et al.* It is also found that most of the defects in the film grown at 0.22 nm s^−1^ start appearing at mid-thickness and are oriented parallel to the *c*-axis, while the defects in the latter film originate just at the substrate surface and are tiled with respect to the *c*-axis. Other planar or line defects in the *ab* plane, such as stacking faults, were not observed. Energy dispersive x-ray spectroscopy (EDX) combined with STEM revealed that the chemical composition of the defects is the same as that of the matrix region and that the impurity oxygen concentration in the films is less than the detection limit. These results indicated that the defects are not an impurity phase such as BaFeO_2_ [429] but may be edge or threading dislocations and/or domain boundaries. The stronger vortex pinning along the *c*-axis for the film grown at the lower rate can be explained by a high density of straight defects with a lateral size (4 nm) close to the double of the *ξ*_*ab*_ of Ba-122:P at 4 K [407]. The very high in-field *J*_c_ observed in these epitaxial films indicates a high potential of Ba-122:P epitaxial films for application to superconducting tapes or wires, though the very high *T*_s_ would not be favorable to film fabrication on metal substrates.

### 4.2. Fabrication of superconducting thin film devices

#### 4.2.1. Bi-crystal Josephson junctions using Ba(Fe,Co)_2_As_2_ epitaxial films

The Josephson junction is the most important basic element in electronic application of superconductors. For low temperature application using liquid He or a cryocooler, Josephson junctions using Nb thin films and a thin Al oxide layer as a tunnel barrier [430] have been in practical use. This sandwich-type junction exhibits ideal superconductor–insulator–superconductor (SIS) type current–voltage (*I*–*V*) characteristics and is very reliable. For cuprate superconductors such as YBa_2_Cu_3_O_7−*x*_ (YBCO), it is very difficult to fabricate SIS type Josephson junctions because of their peculiar physical properties such as very short coherence length and *d*-wave symmetry of the superconducting gap. The very high substrate temperature of 700–800 °C for fabrication of their epitaxial films also makes it very difficult to realize a sharp film–barrier interface required for SIS junctions. However, Josephson junctions exhibiting weak-link-type or superconductor–normal metal–superconductor (SNS) type *I*–*V* characteristics can be readily obtained by utilizing weak links naturally formed at high-angle grain boundaries (GBs) of their thin films [431]. This weak-link behavior at a high-angle GB comes from carrier depletion due to local structural disorder near the GB. Actually, GB Josephson junctions using an epitaxial film on a bicrystal substrate (bicrystal junction) or a substrate with an artificially formed step (step-edge junction) have been applied to electronic devices such as superconducting quantum interference devices (SQUIDs) operating at the liquid-nitrogen temperature [432].

IBSCs also exhibit superconductivity only when a proper amount of charge carrier is doped, though their parent materials are not antiferromagnetic insulators but antiferromagnetic metals. Their short coherence length and rather high film growth temperature would make fabrication of SIS Josephson junctions difficult.

**Figure 86.**
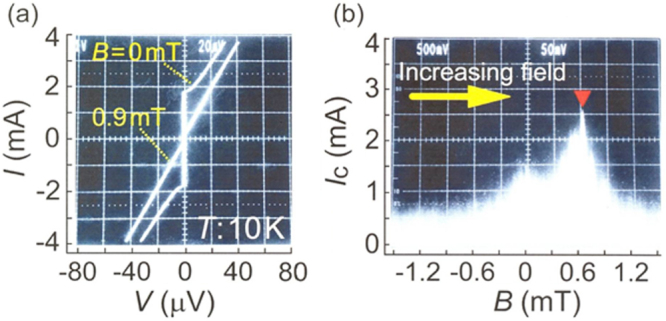
(a) Current–voltage (*I*–*V*) curve under *B* = 0 and 0.9 mT and (b) magnetic field dependence of *I*_c_ (*I*_c_–*B*) curve at 10 K for a 10 *μ*m-wide bridge patterned across the bicrystal GB (BGB) in a Ba-122:Co epitaxial film on an LSAT [001]-tilt bicrystal substrate with the misorientation angle *θ*_GB_ = 30º. Reprinted with permission from [433]. Copyright 2010 by AIP Publishing LLC.

Katase *et al* tried fabricating Josephson junctions using high-quality Ba-122:Co epitaxial films and bicrystal substrates [433]. Ba-122:Co thin films were deposited on LSAT bicrystal substrates with a symmetrical [001]-tilt boundary having a misorientation angle *θ*_GB_ of 30°. Figure 86 shows the *I*–*V* curve and magnetic field dependence of *I*_c_ (*I*_c_–*B* curve) at 10 K for a 10 *μ*m-wide bridge patterned across the bicrystal GB (BGB). The bridge clearly shows a resistively-shunted-junction (RSJ) type curve. The magnitude of *I*_c_ modulation, which is here defined as [*I*_c_ (0) – *I*_c_ (0.9 mT)]/*I*_c_ (0), is approximately 95%, indicating that most of the supercurrent originates from the Josephson current. This is actually the first demonstration of a thin film Josephson junction in IBSCs. However, the hysteresis observed in the *I*_c_–*B* curves suggests that flux trapping occurs in the BGB regions, probably due to inhomogeneity in their microstructures. A 10 *μ*m-wide bridge patterned in a single grain region of the same substrate showed an *I*_c_ of 40 mA at 10 K, implying that the *I*_c_ across the GB is suppressed to less than 1/20 of the film *I*_c_.

**Figure 87.**
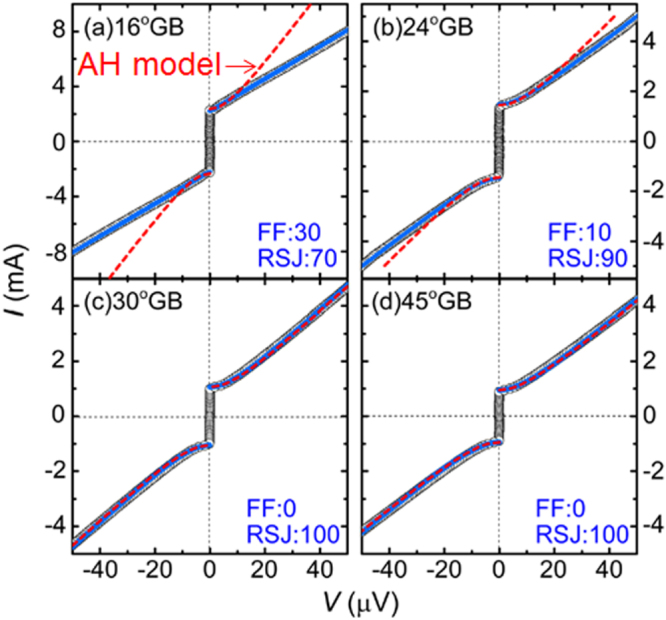
*I*–*V* curves at 12 K for Ba-122:Co BGB junctions with *θ*_GB_ = 16°, 24°, 30°, and 45° grown on MgO bicrystal substrates. The red dotted lines indicate the fits by the AH model, and the blue lines show the fits to the *I*–*V* curves with the phenomenological model combining the RSJ behavior and the FF behavior.

Katase *et al* also fabricated Ba-122:Co epitaxial films on [001]-tilt MgO bicrystal substrates with various misorientation angles and examined the GB transport properties [434]. Though the details will be described in section 4.2.4, *I*–*V* curves for BGB junctions with *θ*_GB_ = 30° and 45° were found to be well fitted by the Ambegaokar–Halperin (AH) model [435], or the RSJ model taking account of thermal fluctuation, confirming that the excess current ratio is minimal for these high-angle BGB junctions. In contrast, BGB junctions with lower *θ*_GB_ showed *I*–*V* curves containing flux–flow behavior. Figure 87 shows the *I*–*V* curves for BGB junctions with *θ*_GB_ = 16°, 24°, 30° and 45°. The dotted lines are the fits by the AH model, while the solid lines are fits by a phenomenological model previously proposed to explain the fractions of flux–flow and RSJ behaviors [436]. The latter fits indicate that the fractions of the RSJ current are approximately 70%, 90%, 100% and 100% for the BGB junctions with *θ*_GB_ = 16°, 24°, 30° and 45°, respectively.

**Figure 88.**
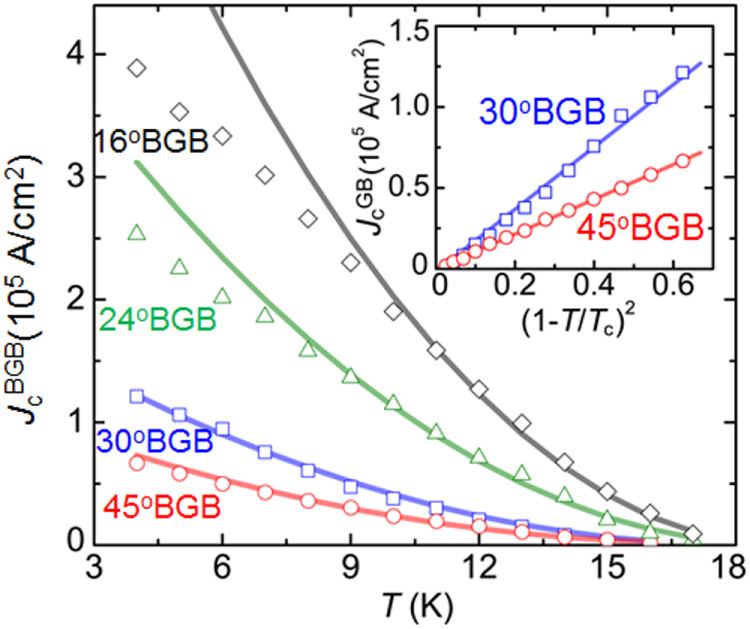
Temperature dependences of *J*_c_ for the BGB junctions with *θ*_GB_ = 16°, 24°, 30°, and 45° grown on MgO bicrystal substrates. The solid lines show the temperature dependences of *J*_c_ predicted from the de Gennes theory. The inset shows a linearized plot of the quadratic temperature dependences for the *θ*_GB_ = 30° and 45° junctions.

The BGB junctions using Ba-122:Co epitaxial films were found to exhibit the specific resistance *AR*_N_ (*A* is the junction area and *R*_N_ is the junction’s normal resistance) of 10^−10^ − 10^−9^ *Ω*cm^2^, which is more than one order of magnitude lower than that for YBCO [001]-tilt bicrystal junctions [431], and metallic temperature dependence. Figure 88 shows the temperature dependence of *J*_c_ for the BGB junctions with *θ*_GB_ = 16°, 24°, 30°, and 45° [434]. Clear quadratic temperature dependence is observed for these high-angle BGB junctions. The solid lines in the figure are the fits to the de Gennes theory based on a conventional proximity effect in the dirty limit [437]

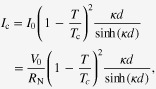
where *d* is the barrier thickness, *κ*^−1^ is the decay length for a normal metal, and *V*_0_ is the characteristic voltage, which is approximately proportional to the energy gap near the barrier. By using the junction resistance *R*_N_ estimated from the *I*–*V* characteristics, all the curves can be well fitted, confirming that the Ba-122:Co BGB junctions are SNS type junctions. This is in contrast to the case of the YBCO BGB junctions, which are basically SIS junctions as indicated by their quasi-linear temperature dependence of *I*_c_ and small hysteresis at low temperatures [431, 438].

#### 4.2.2. Fabrication of DC SQUIDs using bi-crystal junctions shown in section 4.2.1

**Figure 89.**
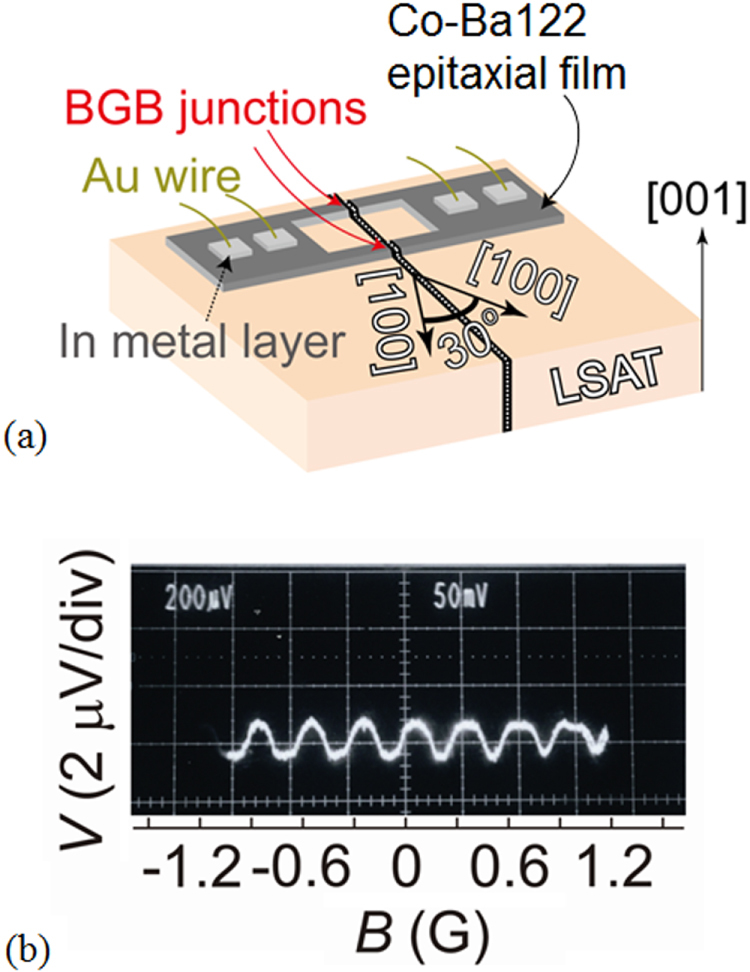
(a) Schematic of DC SQUID structure fabricated using a Ba-122:Co film on LSAT bicrystal substrate with *θ*_GB_ = 30°. (b) Voltage–flux (*V*–*Φ*) characteristics of the DC SQUID measured at 14 K. Reprinted with permission from [439]. Copyright 2010 by IOP Publishing.

Katase *et al* fabricated DC SQUIDs using Ba-122:Co BGB junctions on LSAT [001]-tilt bicrystal substrates with *θ*_GB_ = 30° and demonstrated their operation for the first time [439]. As schematically shown in figure 89(a), the SQUIDs have a loop with 18 × 8 *μ*m^2^ size containing two 3 *μ*m-wide BGB junctions. Figure 89(b) shows the voltage–flux (*V*−*Φ*) characteristics at 14 K. A clear voltage modulation with *ΔV* = 1.4 *μ*V is seen, though its magnitude is one order of magnitude smaller than that typically observed in practical SQUIDs [432].

**Figure 90.**
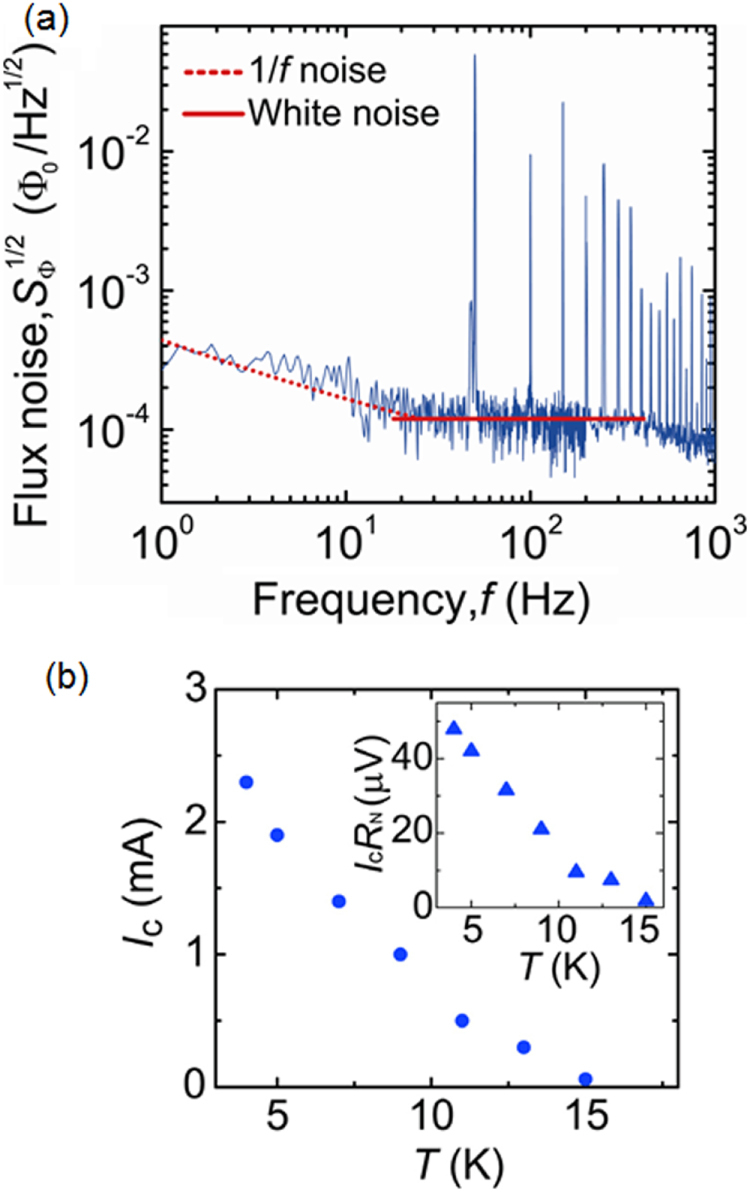
(a) Flux noise (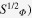 spectrum, including the equivalent input noise in the FLL circuit, measured at 14 K for a Ba-122:Co DC SQUID. (b) Temperature (*T*) dependence of the critical current (*I*_c_) for the SQUID. The inset shows the temperature dependence of the *I*_c_*R*_N_ product. Reprinted with permission from [439]. Copyright 2010 by IOP Publishing.

Figure 90(a) shows the flux noise spectrum for the SQUID at 14 K measured using a commercial flux-locked-loop (FLL) circuit. The white noise level is 1.2 × 10^−4^ *Φ*_0_/Hz^1/2^ and the onset of 1/*f* noise is observed at about 20 Hz. The intrinsic white noise level was estimated to be 9.1 × 10^−5^ *Φ*_0_/Hz^1/2^ by subtracting the contribution of the amplifier noise. This is about one order of magnitude larger than the white noise typically observed for YBCO SQUIDs at 77 K [432, 440, 441]. As shown in figure 90(b), the SQUID or the BGB junctions exhibit a rather steep quadratic temperature dependence of *I*_c_ due to the metallic nature of the barrier. The SQUID shows *I*_c_ less than 100 *μ*A, which is required for operation using the FLL circuit, only at temperatures very close to the junction *T*_c_. However, *I*_c_*R*_N_ at 14 K for instance is as small as 10 *μ*V. The observed small voltage modulation and the large white noise level for the Ba-122: Co SQUIDs can be explained by this small *I*_c_*R*_N_ as well as the small *R*_N_. Thus improvement of BGB junction properties seems necessary to make a SQUID based on doped Ba-122 a practical one.

#### 4.2.3. Fabrication of BaFe_2_(As,P)_2_ step-edge junctions

Ishimaru *et al* tried fabricating step-edge junctions using Ba-122:P epitaxial films [442]. One motivation is the fact that larger *I*_c_*R*_N_ products than BGB junctions were reported in REBCO step-edge junctions [443, 444], although this was more or less attributed to the *d*_*x*_^2^–_*y*_^2^ symmetry of the order parameter in REBCO. Another motivation is that the GB properties in Ba-122:P could be different from those for the Ba-122:Co BGB junctions. Actually substantially higher BGB *J*_c_ has been reported in Ba-122:P epitaxial films fabricated by an MBE method [423].

**Figure 91.**
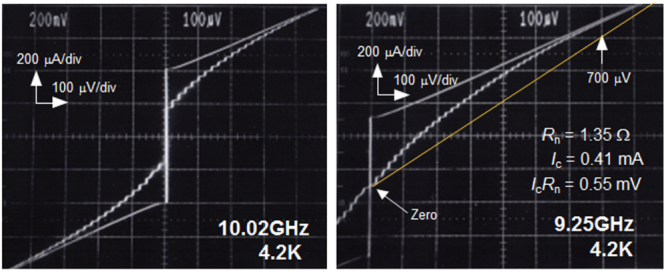
*I*–*V* characteristics at 4.2 K with and without microwave irradiation for a Ba-122:P step-edge junction fabricated on an MgO substrate.

**Figure 92.**
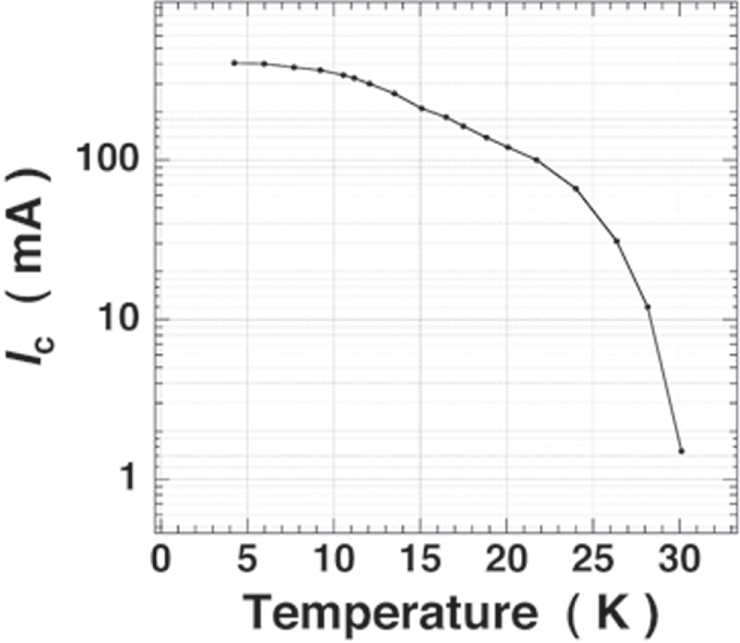
Temperature dependence of *I*_c_ for a Ba-122:P step-edge junction fabricated on an MgO substrate.

A step structure with height of about 70 nm and an angle of 20° was fabricated on MgO (100) substrates by an Ar ion milling technique. An approximately 90 nm thick Ba-122:P epitaxial film deposited on the substrate exhibited a self-field *J*_c_ as high as 6.7 MA cm^−2^ at 4.2 K. Figure 91 shows the *I*–*V* curves for a 10 *μ*m-wide bridge across the substrate step. The *I*–*V* curve without microwave irradiation is RSJ-type. The junction *I*_c_ and *R*_N_ are 0.41 mA and 1.35 *Ω*, respectively, resulting in an *I*_c_*R*_N_ product of 0.55 mV. This *I*_c_*R*_N_ product is one order of magnitude larger than that for the Ba-122:Co BGB junctions [433, 439]. Upon irradiation of 10.02 GHz microwave, clear Shapiro steps are observed. By changing the irradiation frequency and power, the junction *I*_c_ could be suppressed to zero, while Shapiro steps were observed up to the voltage of 0.7 mV. This confirms that the observed supercurrent has Josephson current origin. As shown in figure 92, the junction *I*_c_ could be observed up to the temperature of approximately 30 K, which is very close to the film *T*_c_. However, magnetic field modulation of *I*_c_ was not observed for this step-edge junction. Cross-sectional TEM observation revealed that the Ba-122:P film on the step-slope did not have a single orientation and complicated grain structures, suggesting that Josephson junction is formed not along a single GB but at a GB with a very small area between certain grains. These results indicate the possibility of fabricating GB junctions with better performance for IBSCs, though control of microstructure of the junction region is required.

#### 4.2.4. Effect of grain boundary angle on inter-grain transport properties

The properties of GBs are of great importance for the application of superconducting materials, in particular, to wires and tapes [445]. It is well known that GBs block the supercurrent in cuprate superconductors such as REBCO. For REBCO, *J*_c_ across the grain boundary starts to decrease at a critical angle (*θ*_c_) of approximately 3–5° and shows nearly exponential rapid decay with further increasing *θ*_GB_ [431, 446]. Because of this weak-link behavior, strict control of the in-plane orientation by employing a biaxially textured buffer layer, which is realized, for example, by an ion-beam-assisted deposition (IBAD) technique [447], is required to fabricate REBCO superconducting tapes on flexible metal substrates or coated conductors with a high *J*_c_.

**Figure 93.**
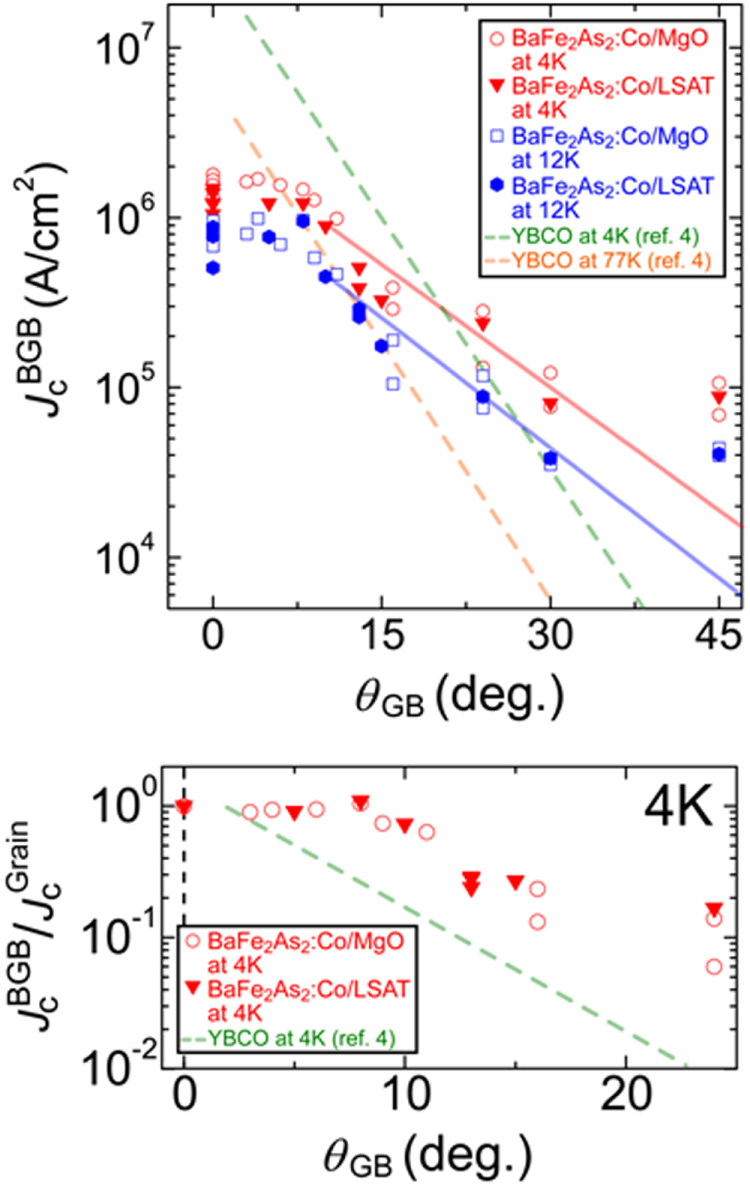
(a) Intergrain transport critical current density 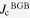 at 4 and 12 K in a self-field as a function of misorientation angle *θ*_GB_ for Ba-122:Co BGB junctions grown on [001]-tilt bicrystal substrates of MgO and LSAT. The red and blue solid lines are fits to the empirical equation 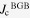 = *J*_c0_exp(–*θ*_GB_/*θ*_0_). The average data for the YBCO BGB junctions taken at 4 and 77 K [431] are also indicated by the green and orange dashed lines, respectively, for comparison. (b) Ratio of 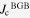 to the intragrain *J*_c_ (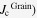 in the range *θ*_GB_ = 0–25^o^ at 4 K. The dashed green line shows the result for the YBCO BGB junctions. Reprinted with permission from Macmillan Publishers Ltd: [434], Copyright 2011.

The first study on the GB properties of IBSCs using Ba-122:Co epitaxial thin films on STO [001]-tilt bicrystal substrates was reported by Lee *et al* [448]. They found that *J*_c_ across the BGB, even with a low misorientation angle of 6°, was strongly suppressed in their low-temperature laser scanning microscope imaging and transport *J*_c_ measurements in a magnetic field of 0.2–0.5 T. Katase *et al* performed a more systematic study on the transport properties of BGBs with *θ*_GB_ = 3–45° using high-quality Ba-122:Co epitaxial thin films with a self-field *J*_c_ of well above 1 MA cm^−2^ at 4 K prepared on both MgO and LSAT bicrystal substrates [434]. Figure 93(a) shows the self-field *J*_c_ of BGB (*J*_c BGB_) at 4 and 12 K as a function of *θ*_GB_. Nearly exponential decay of *J*_c BGB_ for *θ*_GB_ above approximately 10° at both temperatures is observed, indicating that high-angle BGBs act as weak links. However, the exponential decay is more gradual than that for YBCO BGBs. Figure 93(b) shows the *θ*_GB_ dependence of *J*_c BGB_ normalized by the film *J*_c_ at 4 K for lower-angle BGBs. The ratio starts to decrease at a critical angle *θ*_c_ of approximately 9–10°, while there is no reduction for *θ*_GB_ < *θ*_c_, indicating that BGBs in this region are strong links. This critical angle is substantially larger than the value of 3–5° reported for YBCO BGBs [431].

**Figure 94.**
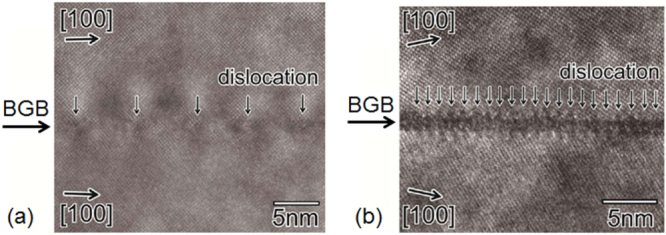
[001] plan-view HRTEM images of the Ba-122:Co BGB junctions on MgO bicrystal substrates with *θ*_GB_ = (a) 4°, and (b) 24°. Misfit dislocations are marked by the down-pointing arrows [434]. Reprinted with permission from Macmillan Publishers Ltd: [434], Copyright 2011.

Figure 94 shows [001] plan-view HR-TEM images of the Ba-122:Co BGB junctions on MgO bicrystal substrates. The BGBs with *θ*_GB_ = 4° and 24° clearly indicated an array of misfit dislocations with the periodic distance of approximately 5.0 nm for *θ*_GB_ = 4^o^ and 1.2 nm for *θ*_GB_ = 24^o^. Using a geometric tilted boundary model, the grain boundary dislocation spacing *D* is given by 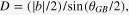 where 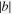 is the norm of the corresponding Burgers vector. With the lattice constant *a* = 0.396 nm of Ba-122, *D* is estimated to be 5.7 nm and 1.0 nm for *θ*_GB_ = 4^o^ and 24^o^, respectively. The estimated *D* values are very similar to the *D* values observed above. Energy dispersive spectroscopy (EDS) line spectra across the BGBs and parallel to the BGBs confirmed that the chemical compositions of the BGBs and the film region are homogeneous, and no secondary phase was observed in the BGB regions.

The critical angle of 9° observed in Ba-122:Co BGBs corresponds to the spacing between the misfit dislocations of approximately 2.8 nm, which is comparable to or slightly larger than the coherence length *ξ*_*ab*_(T) of 2.6 nm at 4 K [319]. This is consistent with the notion that strong supercurrent channels still remain between the dislocations for the case of *θ*_GB_ < *θ*_c_, while a coherent supercurrent cannot pass through the BGBs at *θ*_GB_ > *θ*_c_, giving rise to the weak-link behavior. For the case of YBCO, the critical angle of 5° corresponds to *D* = 4.5 nm, which is much larger than *ξ*_*ab*_(T) of 1.6 nm at 4 K. It was previously pointed out that strain near the dislocation cores induces a local transition to an antiferromagnetic phase and forms insulating regions near dislocation cores in cuprate superconductors [446]. The smaller critical angle for YBCO could be explained by the formation of such insulating regions.

For higher-angle BGBs, the distance between the dislocations becomes smaller and eventually dislocations overlap each other. For the case of YBCO, the GB regions become carrier-depleted and thus insulating, though several origins for this, such as shift of chemical potential due to excess ion charge [446], band bending [431], and local structural distortion [449], were proposed. Since carrier-depleted IBSC materials are antiferromagnetic metals (or semi-metals) not insulators, a different nature of high-angle GBs is expected. Actually, as indicated in figure 88, the Ba-122:Co high-angle BGB junctions exhibit normal-metal-like behavior of the junction barrier. The more gradual exponential decay of *J*_c BGB_ in Ba-122:Co BGB junctions than that for YBCO BGB junctions in figure 93 can be attributed to this metallic nature of the GB region. The metallic nature of the GB region could also explain the critical angle *θ*_c_ which is substantially larger than that of YBCO and consistent with the dislocation distance *D*.

The observed larger critical angle appears to afford a great advantage for application to superconducting tapes since less strict control of the in-plane alignment for buffer layers would be required to obtain high-*J*_c_ IBSC films on flexible metal substrates. Moreover, this would be advantageous for the fabrication of superconducting wires, for example, based on the powder-in-tube (PIT) method where grains are not highly oriented.

#### 4.2.5. Approach to electrostatic field controlled device

**Figure 95.**
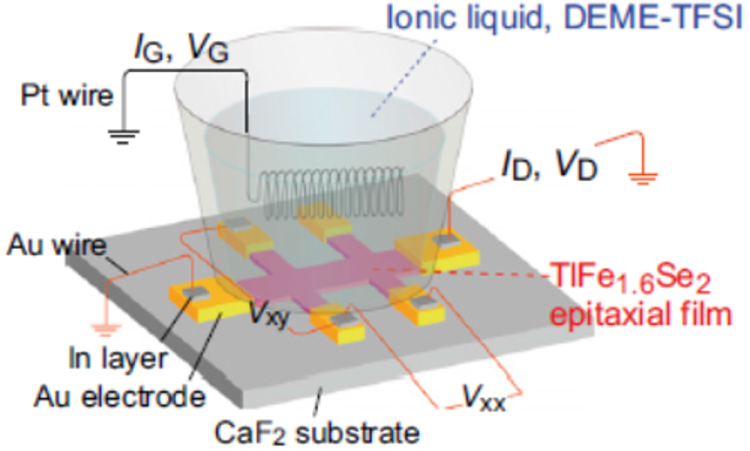
Schematic of the EDLT using TlFe_1.6_Se_2_ epitaxial film with a six-terminal Hall bar structure on a CaF_2_ substrate. *V*_G_ was applied via a Pt counter electrode through the ionic liquid, DEME-TFSI, contained in a silica glass cup. Electrical contacts were formed using Au wires and In/Au pads. Reprinted with permission from [464]. Copyright 2014 by the National Academy of Sciences.

The modulation of electronic properties by applying electric fields is a commonly used technique in semiconducting materials. The electric double-layer transistor (EDLT) type device composed of an atomically flat film of an insulator/semiconductor and an ionic liquid (or a polymer electrolyte) as a gate electrode (shown in figure 95 as an example) is one of the efficient electric-field devices because such devices can accumulate extremely high currier density up to 10^15^ cm^−2^ and insulator-to-metal transitions have been demonstrated for various materials such as organic polymers, InO_*x*_ polycrystalline films, and ZnO single-crystal films [450–453].

In 2008, Ueno *et al* [454] reported that the superconductivity of pristine SrTiO_3_ single-crystal surface emerged using an EDLT structure. The critical temperature (*T*_c_ = 0.4 K) was comparable to the maximum value for a chemically doped bulk crystal [455]. This success proposed a new route to evolve superconductivity. Inspired by this result, insulator–superconductor transitions using EDLT devices have been reported in KTaO_3_ (*T*_c_ = 0.047 K) [456], ZrNCl (*T*_c_ = 15.2 K) [457], and MoS_2_ (*T*_c_ = 9–11 K) [458, 459]. The similar device has been applied on cuprate superconductors. In the case of epitaxial film of La_2−*x*_Sr_*x*_CuO_4_, its underdoped and insulating film changed into a superconductor (*T*_c_ = 30 K) by applying −4.5 V to the gate electrode [460]. In the case of YBCO, the enhancement of *T*_c_ (from 83 to 134 K by applying −3 V (hole accumulation) of gate voltage (*V*_G_)) was observed but its decrease in resistivity from onset *T*_c_ to lower temperature (i.e., superconducting transition) was very broad [461]. When the positive *V*_G_ was applied (i.e., electron accumulation), *T*_c_ decreased with increasing *V*_G_ [461–463], whereas the resistivity changes seriously by keeping the device under the constant *V*_G_. From this phenomenon, it is considered that the modulation of *T*_c_ in YBCO devices is due not only to the carrier accumulation but also the changing in the defect concentration (possibly oxygen) by the electro-chemical effect.

Though the parent materials of IBSCs are generally antiferromagnetic metals, only *A*_0.8_Fe_1.6_Se_2_ (*A*: K, Rb, Cs, Tl) is an AFM Mott insulator, where iron vacancy (*V*_Fe_) order forms a √5 × √5 × 1 supercell [155]. This material is called the 245 type of IBSC of which the crystal structure of the fundamental cell (tetragonal ThCr_2_Si_2_-type) is the same as that of the 122 type (see section 3.1.1). By decreasing *V*_Fe_, it changes to metal and subsequently reveals superconductivity (maximum *T*_c_ = 32 K) [154].

**Figure 96.**
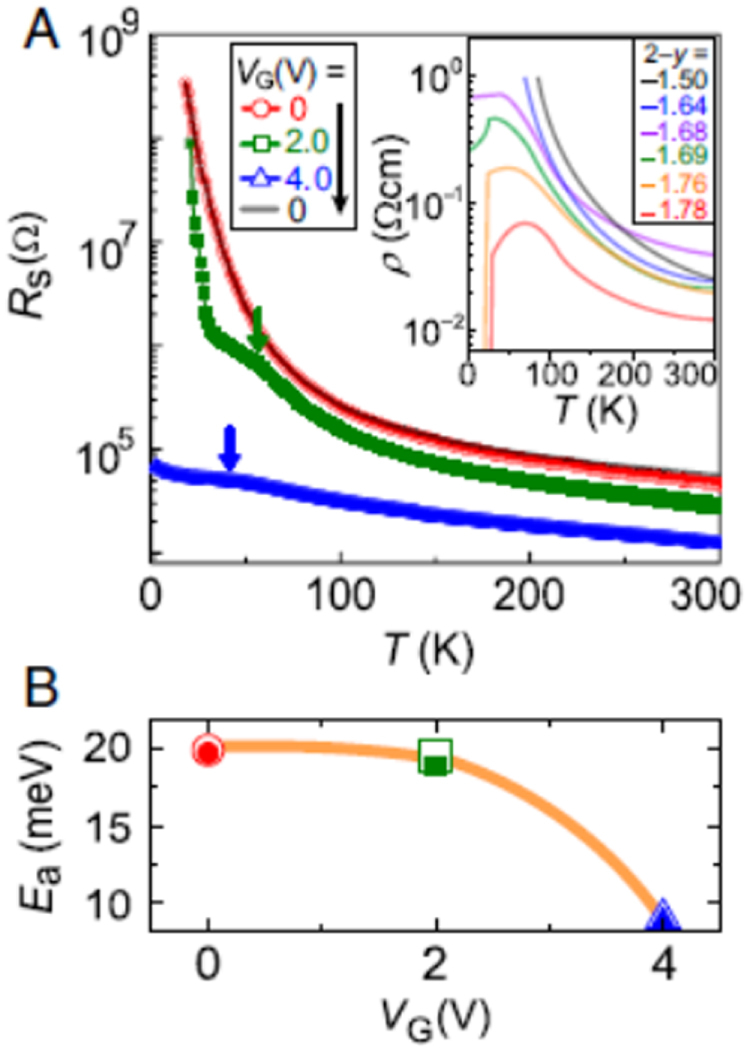
(A) *T* dependences of *R*_s_ for the TlFe_1.6_Se_2_ EDLT measured with increasing *T* (open symbols) and decreasing *T* (filled symbols) at *V*_G_ = 0 → +2.0 → +4.0 → 0 V. The arrows indicate the positions of resistance humps. The reported *ρ–T* curves of (Tl,K) Fe_2−*y*_Se_2_ bulk materials [156] are shown for comparison (inset). A resistance hump appears at 2−*y* ≥ 1.68, and superconductivity emerges at 2−*y* ≥ 1.76. (B) The *E*_a_ estimated from the high *T* region of the *ρ–T* curve shown in (A) as a function of *V*_G_. Reprinted with permission from [464]. Copyright 2014 by the National Academy of Sciences.

The FIRST Project attempted to tune the transport property of TlFe_1.6_Se_2_ by carrier accumulation using EDLT structure [464]. The reason to select TlFe_1.6_Se_2_ is its chemical stability compared to alkali metal-based (K, Rb and Cs) 245-type compounds. Employing CaF_2_ substrate, TlFe_1.6_Se_2_ film was deposited by PLD method with epitaxial relationships of [001] TlFe_1.6_Se_2_//[001] CaF_2_ (out of plane) and [310] TlFe_1.6_Se_2_//[100] CaF_2_ (in plane). The ordering of *V*_Fe_, which indicates the insulating phase, was clearly observed in the high-angle annular dark field STEM images. The EDLT type device was fabricated using a 20 nm thick TlFe_1.6_Se_2_ film. The ionic liquid, N,N-diethyl-N-methyl N-(2-methoxyethyl)-ammonium bis-(trifluoromethylsulfonyl) imide, covered the EDLT device and a Pt coil electrode was inserted into the ionic liquid to act as a gate electrode. Figure 96 shows the *R*–*T* curves under applying *V*_G_ at 0, 2 and 4 V and activation energy in the high temperature region. Unfortunately, a superconductivity transition did not emerge but the electrostatic carrier doping has been controlled successfully by the EDLT structure. Moreover, the EDLT structure induced the phase transition (resistance humps marked by the arrow in figure 96(a)) assignable to a magnetic phase transition or the formation of an orbital-sensitive Mott phase, which has been commonly observed as a precursory phenomenon of transition to a superconducting state. This demonstration of carrier doping of the Mott insulator by the electrostatic method offers a way to extend the exploration of high-*T*_c_ superconductors to even insulating materials.

### 4.3. Fabrication of superconducting tapes by deposition

#### 4.3.1. Fabrication of a short Ba(Fe,Co)_2_As_2_ tape

Superconducting tapes or coated conductors which are fabricated by depositing superconducting films on flexible metal tapes with proper buffer layers have been demonstrated to be practical conductors with high critical current for the case of REBCO cuprate superconductors [465]. In order to realize a high-*J*_c_ REBCO film layer on a biaxially textured oxide buffer layer, the IBAD technique [447] or rolling-assisted biaxially textured substrate (RABiTS) technique [466] has been employed. For the case of MgB_2_ with no significant GB problem, the PIT technique enables production of round superconducting wires which are more favorable for magnet application [467], though they can only be used at low temperatures below 20 K. Since IBSCs also exhibit weak-link behavior at GBs with the misorientation angle larger than the critical angle of approximately 9°, as described in the previous section [434], the coated conductor technique would be one promising candidate for production of practical conductors.

The first trial fabrication of iron-based 122 compound films on flexible metal substrates with biaxially textured buffer layers was reported by Iida *et al* [468]. By employing the Fe buffer architecture, they realized the biaxially textured growth of Ba-122:Co thin films on IBAD–MgO-buffered Hastelloy substrates, which are typically used for the fabrication of REBCO coated conductors. The films exhibited in-plane misorientation *Δϕ* of about 5°, which was slightly smaller than that of the homoepitaxial MgO/IBAD–MgO layer. They also showed a broader transition width and a substantially lower self-field *J*_c_ than those for films on MgO single-crystal substrates, although a substantial improvement in *J*_c_ has recently been reported [425].

**Figure 97.**
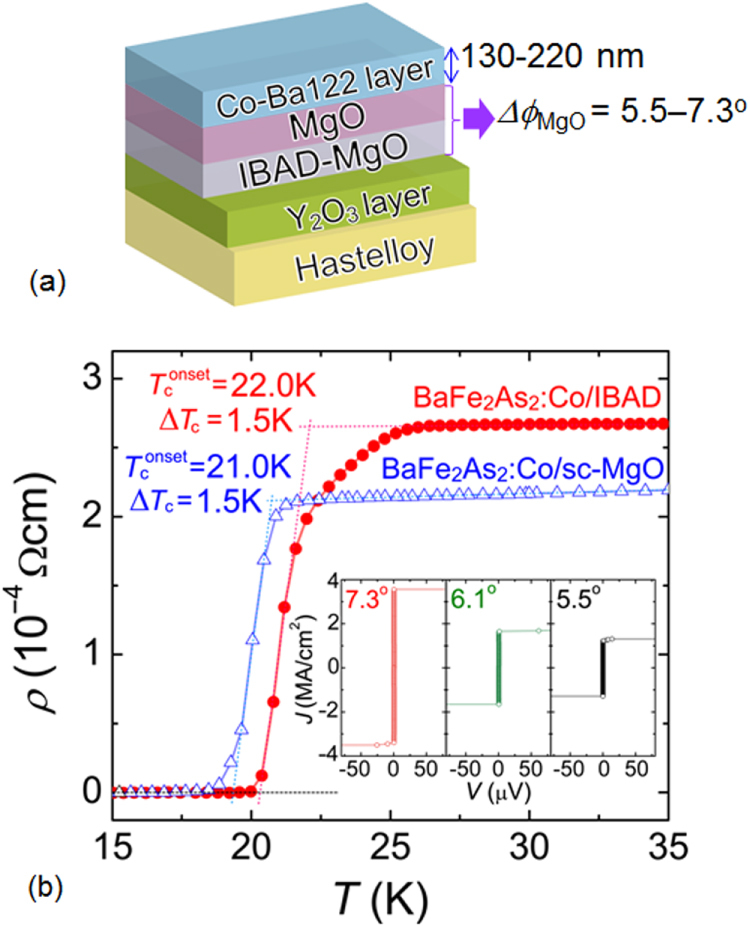
(a) Schematic cross-section and (b) *ρ*–*T* curves for Ba-122:Co thin films on IBAD–MgO substrate (circles) and single-crystal MgO (triangles). The inset shows the *J*–*V* characteristics at 2 K of the films on IBAD–MgO with (left) *Δϕ*_MgO_ = 7.3°, (middle) 6.1°, and (right) 5.5°. Reprinted with permission from [406]. Copyright 2011 by AIP Publishing LLC.

**Figure 98.**
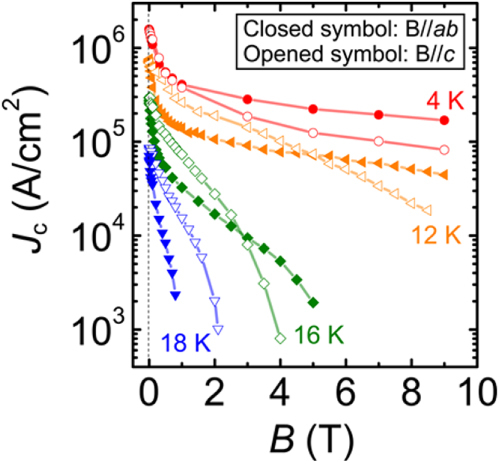
Magnetic field dependence of *J*_c_ at 4, 12, 16, and 18 K for a Ba-122:Co thin films on IBAD–MgO substrate. The closed and open symbols represent *J*_c_ for fields parallel to the *a*, *b*-axes and *c*-axis, respectively. Reprinted with permission from [406]. Copyright 2011 by AIP Publishing LLC.

Katase *et al* succeeded in preparing biaxially textured Ba-122:Co thin films directly on IBAD–MgO-buffered Hastelloy substrates [406]. Figure 97(a) schematically shows the film on the structure. They used 10 × 10 mm^2^ substrates with moderate in-plane alignment for the epitaxial-MgO layer on IBAD MgO *Δϕ*_MgO_ of 5.5–7.3°. X-ray diffraction revealed that the films had a substantially smaller *Δϕ* of approximately 3°, irrespective of the *Δϕ*_MgO_ value. As shown in figure 97(b), the Ba-122:Co films exhibited a resistive transition as sharp as that for films on MgO single crystal substrates and high self-field *J*_c_ values of 1.2–3.6 MA cm^−2^ at 2 K. Figure 98 shows the magnetic field dependence of *J*_c_ (*J*_c_–*H*) at 4–18 K for the film on the substrate with *Δϕ*_MgO_ = 6.1°. At 4 K, *J*_c_(*H*//*ab*) is larger than *J*_c_(*H*//*c*) in almost the whole field range, while crossovers are observed at 5.0 T at 12 K and 2.9 T at 16 K, respectively. At a higher *T* of 18 K, *J*_c_(*H*//*c*) is greater than *J*_c_(*H*//*ab*) in the whole *H* region. These results suggest the existence of naturally-formed relatively strong vortex pinning centers along the *c*-axis in the Ba-122 film on the metal substrate, as observed in the Ba-122:Co epitaxial films on LSAT substrates [400]. The pinning force density *F*_p_ was found to show the largest value of ∼8 GN m^−3^ at 4 K which is smaller than those of Ba-122:Co epitaxial films on LSAT substrates. This suggests that much higher in-field *J*_c_ would also be realized on IBAD substrates by further optimizing or introducing the *c*-axis correlated pinning centers. These results demonstrated the possibility of fabricating high-*J*_c_ coated conductors with Ba-122 compounds by a rather simple low-cost process using less textured templates with a large *Δϕ*.

#### 4.3.2. Fabrication of a long BaFe_2_(As, P)_2_ tape

**Figure 99.**
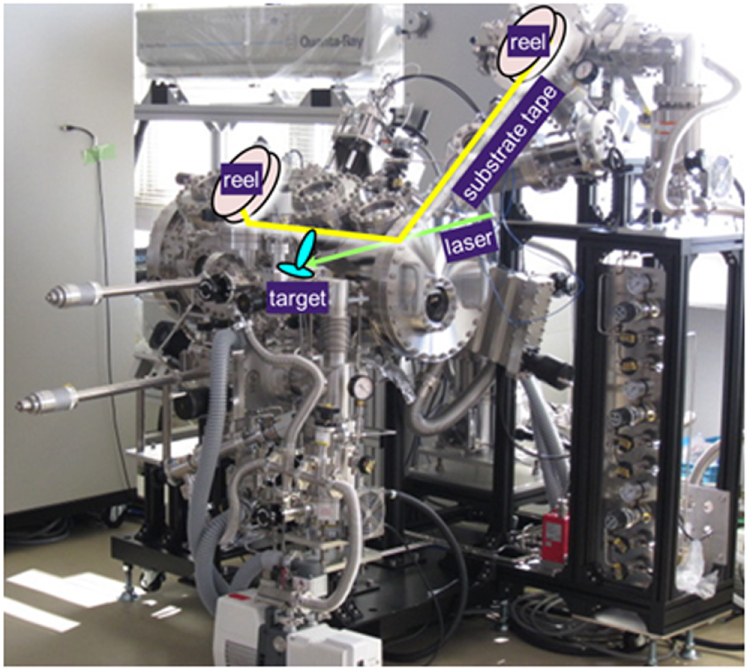
Photograph of a reel-to-reel PLD system designed for fabrication of long Ba-122:P coated conductors.

As described in sections 4.1.2 and 4.1.3, Ba-122:P epitaxial films exhibited a higher *T*_c_ than Ba-122:Co films and a substantially higher in-field *J*_c_ by introducing artificial pinning centers consisting of oxide nanoparticles or tuning naturally formed pinning centers along the *c*-axis [415, 422]. Thus Miyata *et al* [469] tried fabricating long coated conductors using Ba-122:P films on flexible metal tapes using a PLD system shown in figure 99. The system is equipped with a reel-to-reel tape feeding mechanism that enables deposition on a tape longer than 1 m. Two second-harmonic Nd:YAG lasers can simultaneously generate two laser plumes on a target. The target–tape distance is variable between 32 and 57 mm. The energy density of one plume on the target was 2–3 J cm^−2^ and the repetition rate was 2.5–20 Hz. The target could be changed to a new one during a short interval of deposition without breaking vacuum.

**Figure 100.**
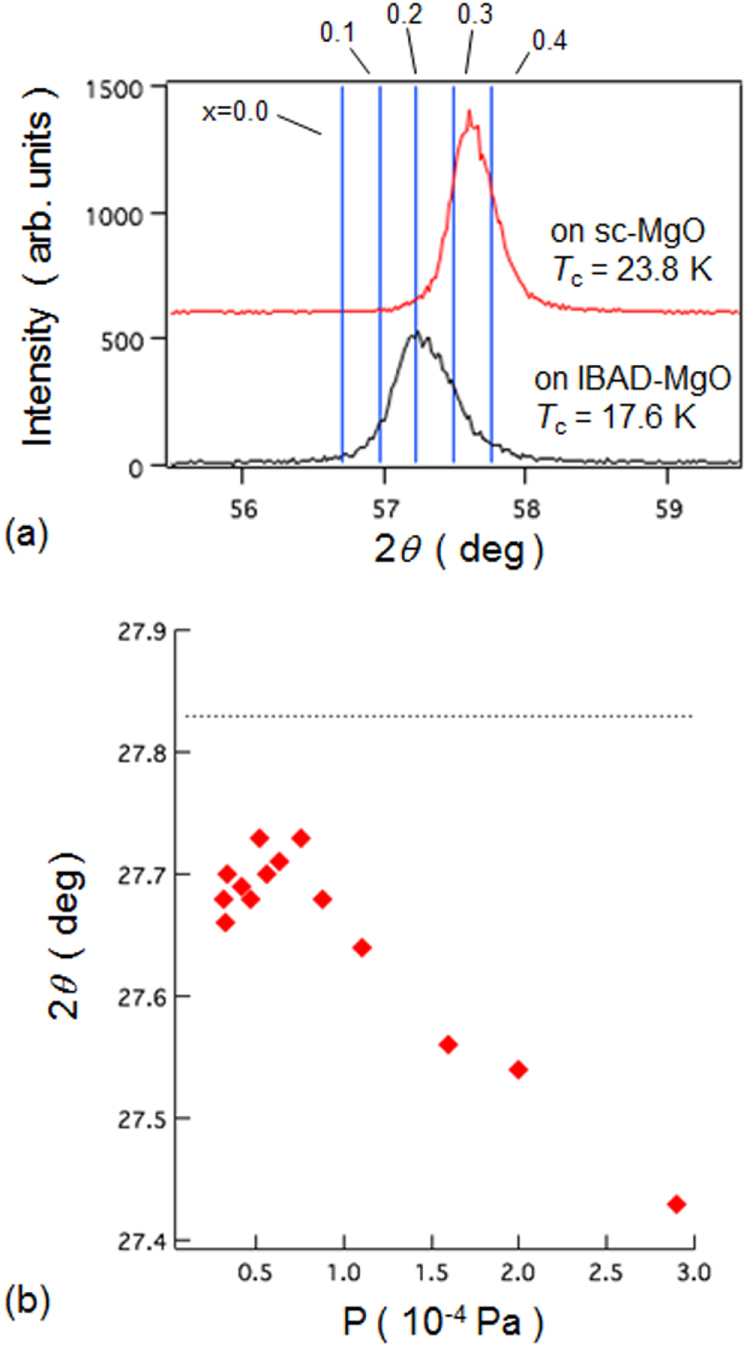
(a) Comparison of x-ray diffraction patterns for typical Ba-122:P films prepared on IBAD–MgO and single crystal (sc-) MgO substrates using a PLD target with the nominal P content *x* of 0.40. (b) Correlation between the position of the (004) diffraction peak with the residual pressure before deposition for Ba-122:P films on IBAD–MgO substrates.

Two types of IBAD–MgO-buffered Hastelloy tapes, commercial and home-made, were used. The commercial tape employed homoepitaxial-MgO (epi-MgO)/IBAD-MgO/solution-deposited (SDP) Y_2_O_3_/Hastelloy architecture, while the latter employed epi-MgO/IBAD-MgO/sputtered Gd_2_Zr_2_O_7_ (GZO)/Hastelloy architecture. In either case, 50–100 nm-thick epi-MgO layers exhibited *Δϕ* of ∼5°. As the first step, deposition was performed using a Ba-122:P target with the nominal P content of 0.40 on a fixed tape without travelling at a substrate temperature of approximately 850 °C. The deposited film exhibited biaxial texture and a resistive transition with a 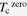 of 17.6 K, which was substantially lower that for the film on single-crystal (sc) MgO (∼24 K) [404]. In figure 100(a), x-ray diffraction patterns for the film on IBAD–MgO and sc-MgO are compared. A clear shift of the (008) diffraction peak to a lower angle side for the former film is observed. This peak position is close to that for Ba-122:P with *x* = 0.2, suggesting a loss of P in the film. Cross-sectional elemental map analysis suggested the existence of a phase consisting of Ba–P–O which grew along the *c*-axis. It was also found that the (004) as well as (008) peak position was sensitive to the background pressure before deposition, as shown in figure 100(b). Since a more than 1 m-long metal tape was loaded between two reels even for deposition of a short-length sample, careful degassing of the whole the tape was required to obtain low residual gas pressure.

**Figure 101.**
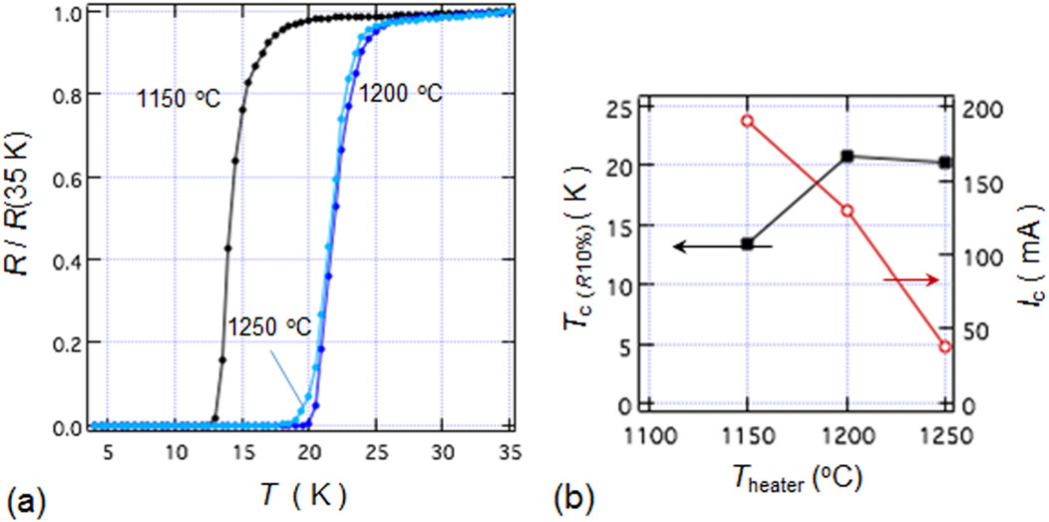
(a) Temperature dependences of resistance for Ba-122:P films deposited on fixed IBAD–MgO-buffered metal tapes under low residual pressure below 10^−4^ Pa at heater setting temperatures of 1150–1250 °C. (b) Dependence of *T*_c_ (*R* 10%) and *I*_c_ on the heater setting temperature for 2 mm-wide Ba-122:P films on IBAD–MgO-buffered metal tapes.

**Figure 102.**
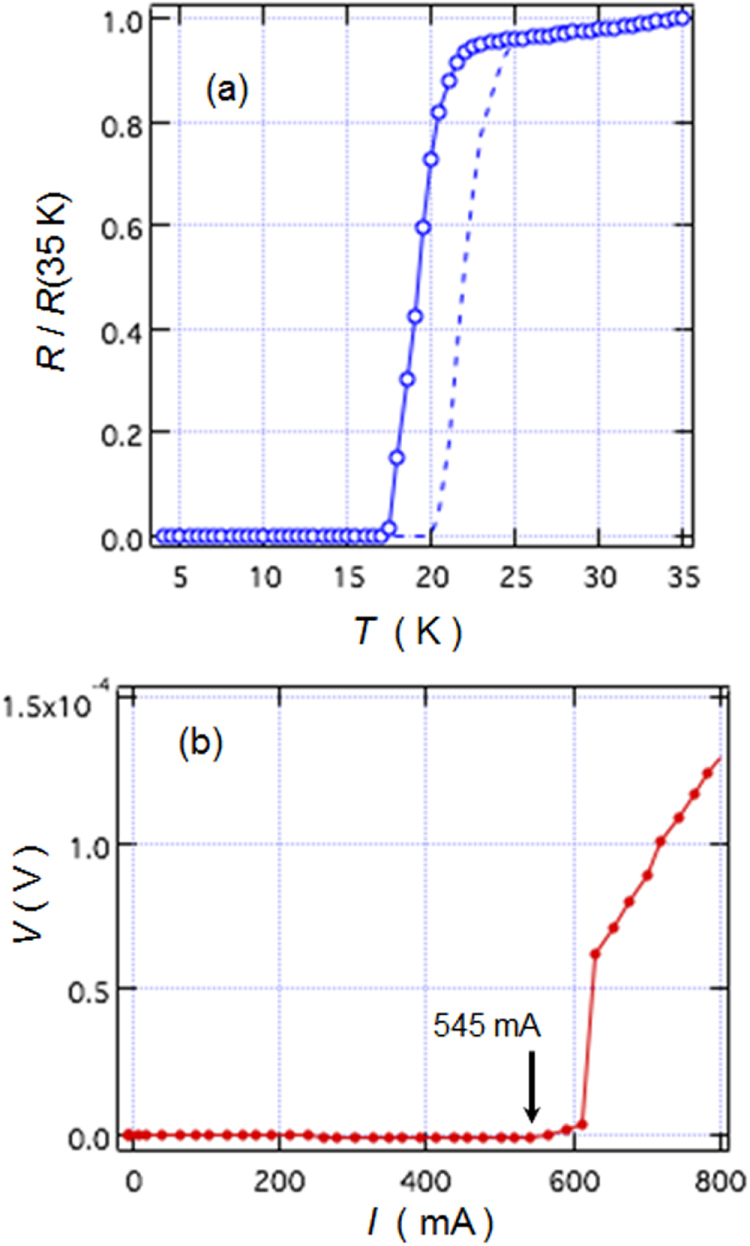
(a) *R*–*T* curve (open circle) and (b) *I*–*V* curve at 4.2 K for a 2 mm-wide rectangular piece cut from a 5 cm long Ba-122:P coated conductor which was fabricated at a tape travelling speed of 6 mm min^−1^ using a Ba-122:P (*x* = 0.40) target. The dashed line in (a) shows the *R*–*T* curve of the tape fabricated under static conditions.

**Figure 103.**
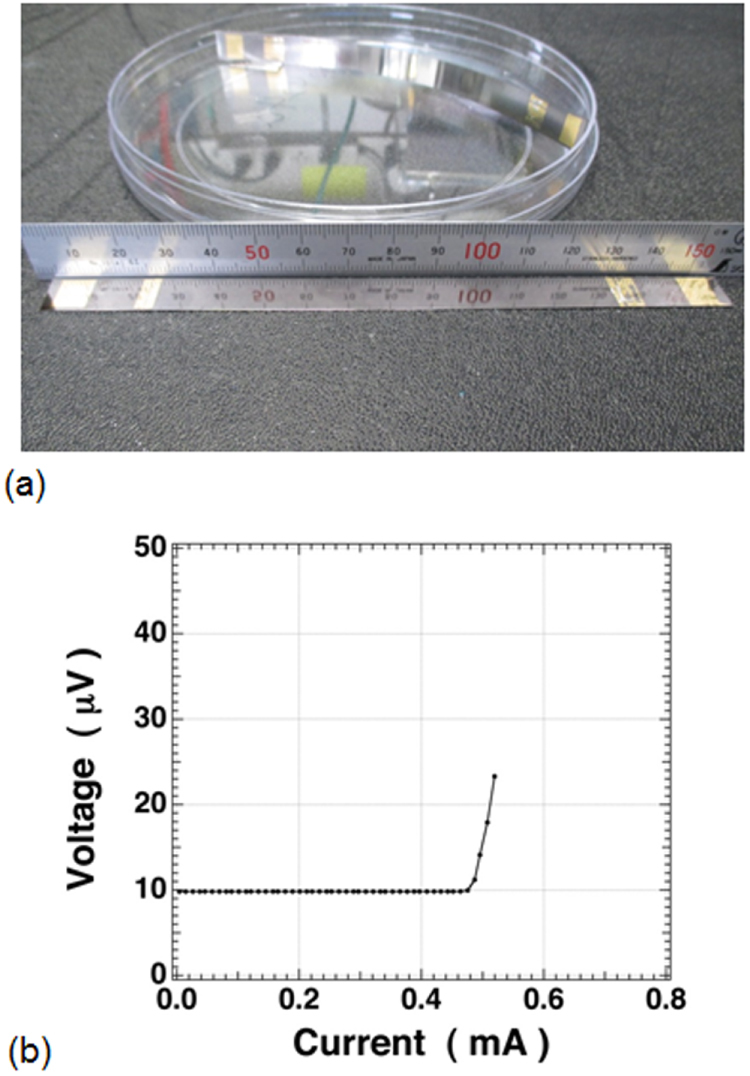
(a) Photograph and (b) *I*–*V* curve measured at 4.2 K for a 15 cm-long Ba-122:P coated conductor fabricated at a tape travelling speed of 6 mm min^−1^. The width of the tape is 1 cm and the Au electrodes of the four probe method are attached where the distance between the inner electrodes is 10 cm.

Figure 101(a) shows the temperature dependence of resistance for films deposited on fixed tapes under low residual pressure below 10^−4^ Pa at heater setting temperatures of 1150–1250 °C. The films deposited at a temperature of 1200 °C exhibited the highest *T*_c_ (*R* 10%) of 20.7 K, while the film *I*_c_ tends to decrease with increasing the temperature, as shown in figure 101(b), suggesting a change in film morphology. Figures 102(a) and (b) show the *R*–*T* curve and the *I*–*V* curve at 4.2 K, respectively, for a 2 mm-wide rectangular piece cut from a 5 cm-long coated conductor which was fabricated at a tape travelling speed of 6 mm min^−1^ using another Ba-122:P (*x* = 0.40) target. Although its *T*_c_ (*R* 10%) (=17.8 K), which means the temperature where resistivity down to 10% of normal state by superconductivity transition, is lower than that for the film deposited on a fixed tape, the observed self-field *I*_c_ of 0.55 mA corresponds to a *J*_c_ of 1.1 × 10^5^ A cm^−2^. Figure 103(a) shows a picture of a 15 cm-long coated conductor fabricated at a tape travelling speed of 6 mm min^−1^. The coated conductor showed a *T*_c_ (*R* 10%) of 18.7 K and an overall self-field *I*_c_ at 4.2 K for 1cm width and 10 cm length of 0.47 mA, as shown in figure 103(b). This *I*_c_ corresponds to a *J*_c_ of 4.7 × 10^4^ A cm^−2^. This lower overall *J*_c_ than that for the shorter sample indicates the inhomogeneity of the film properties along the length and/or possibly across the width.

**Figure 104.**
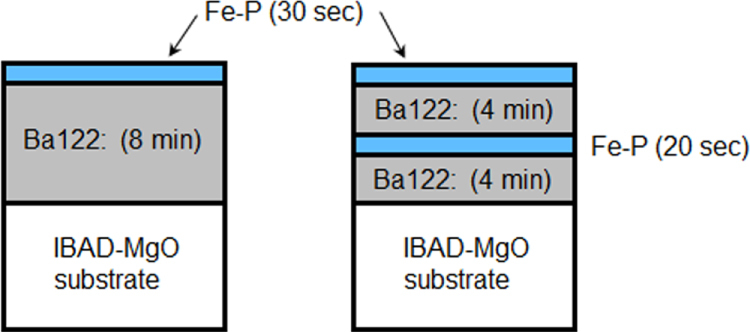
Schematic cross-section of Ba-122:P/Fe–P multilayer films on IBAD–MgO substrates.

**Figure 105.**
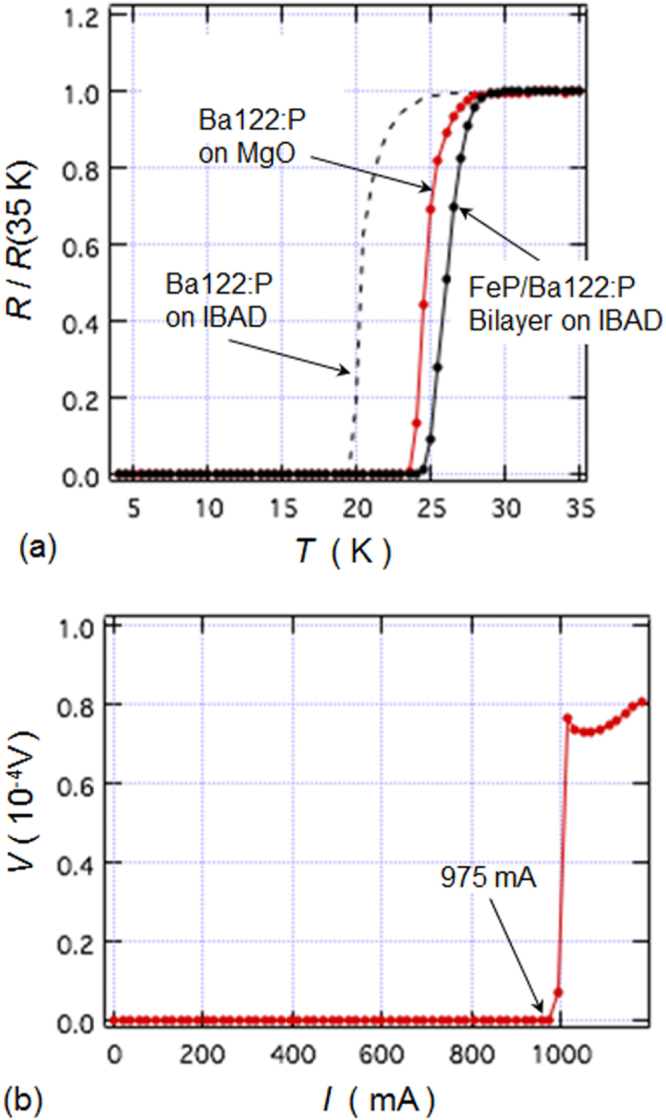
(a) *R*–*T* curves and (b) *I*–*V* curve measured at 4.2 K for 280 nm thick Fe_3_P/Ba122:P bilayer films.

Although the fabrication conditions have not yet been optimized, the superconducting properties of the trial-fabricated coated conductors are inferior to those for the Ba-122:P films on sc-MgO. One reason for the inferior properties could be a difference in film morphology. Scanning electron microscopy (SEM) observation of the surface of a coated conductor fabricated on a moving tape revealed a rather rough surface with bumps. This is clearly different from the surface for the high-*J*_c_ films on sc-MgO with average surface roughness *R*_a_ smaller than 4 nm. The lower *T*_c_ values for the coated conductors also suggest that the actual P content is still lower than the optimal P content in the target. In order to examine the influence of the P content, Ishimaru *et al* [470] fabricated multilayer films on the fixed IBAD–MgO-buffered tapes, as schematically shown in figure 104, using Ba-122:P (*x* = 0.33) and Fe_3_P targets. Figure 105 shows the *R*–*T* curve and the *I*–*V* curve at 4.2 K for a 280 nm thick Fe_3_P/Ba-122:P bilayer film. The film exhibits a *T*_c_ (R 10%) of approximately 24.0 K, which is closer to the *T*_c_ for the film on sc-MgO. The self-field *I*_c_ corresponds to a *J*_c_ of 1.75 × 10^5^ A cm^−2^, which is substantially improved as compared with the single-layer film. The four-layer film showed an even higher *T*_c_ (*R* 10%) of 29.5 K, though its self-field *J*_c_ was decreased (0.88 × 10^5^ A cm^−2^). These results confirm that the films on IBAD–MgO-buffered tapes are actually P-deficient. Since the EDS results for the films fabricated under higher residual gas pressure indicated the formation of a Ba–P–O phase, the surface oxygen on the epi-MgO layer might react with the Ba-122:P films to some extent, leading to the P-deficient composition. The superconducting properties of Ba-122:P coated conductors could be further improved by employing a more stable thin buffer layer, for example, BZO, in addition to optimization of deposition parameters.

### 4.4. Fabrication of tapes and wires by the powder-in-tube method

Among IBSCs, K-doped (Ba,K)Fe_2_As_2_(Ba-122) and (Sr,K)Fe_2_As_2_(Sr-122) are most potentially useful for high field applications due to their high critical temperature (*T*_c_) value of ∼39 K and high upper critical field (*H*_c2_) of over 50 T [319]. The relatively smaller anisotropy of Ba-122 compounds compared to those of cuprate superconductors [194] is also attractive for magnet applications because it is expected to bring about a higher irreversibility field, *H*_irr_. Furthermore, the critical angle (*θ*_c_) of the transition from a strong link to a weak link for Ba-122 is substantially larger than that for YBCO-based conductors [434]. For the high magnetic field magnet applications, we have to develop a high performance superconducting tape or wire conductor. In order to evaluate the potential for tape and wire applications, the development of a tape and wire processing technique is essential.

For Ba-122 and Sr-122 superconductors, PIT and coated conductor processes have been developed for tape and wire fabrications. Iron-based coated conductors have been grown by several groups utilizing existing YBCO coated conductor technology and have been found to have a self-field *J*_c_ of over 1 MA cm^−2^ [425, 471, 472]. Although, at an early stage of development, the transport *J*_c_ in IBSCs reported was disappointingly low due to the weak link grain boundary problem [448, 473–485], astonishing progress has been made for Ba(Sr)-122 tapes in the past several years. *J*_c_ for Ba(Sr)-122 tapes and wires approaches 10^4^ A cm^−2^ at 4.2 K and 10 T through metal addition plus the rolling induced texture process, hot isostatic press method, cold press method, hot press method, and so on [486–495]. These results demonstrated that mechanical deformation is critical for producing high quality superconducting tapes and wires, which plays an important role in densifying the conductor core and aligning the grains of the superconducting phase. An understanding of the influence of mechanical deformation on the microstructure and superconducting properties will accelerate the development of the appropriate processes and further improve the transport *J*_c_ of Ba(Sr)-122 tapes and wires. In this section, we report our recent development of Ba(Sr)-122 tape conductors by applying a conventional PIT method. Emphasis was placed on the relation between the microstructure and critical current properties. The influence of superconducting core density, grain alignment, and microstructure on *J*_c_ in the tapes was systematically investigated.

#### 4.4.1. Fabrication of tape conductors

**Figure 106.**
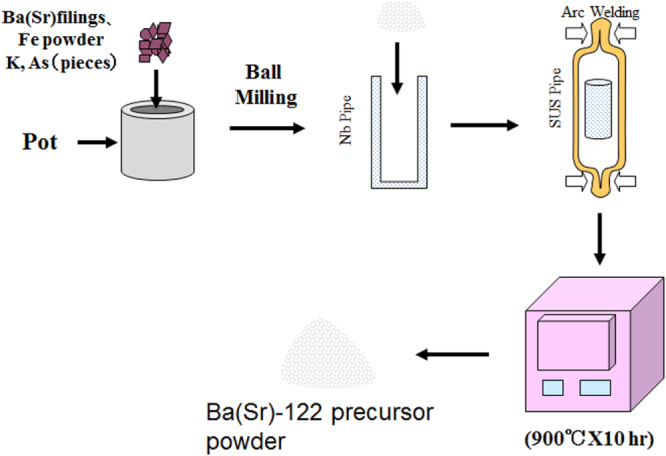
Fabrication of the Ba(Sr)-122 precursor powder.

**Figure 107.**
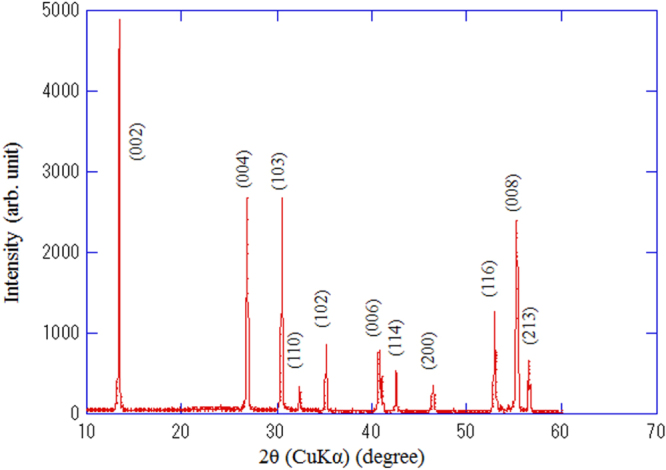
X-ray diffraction pattern of the Ba-122 precursor powder. Reprinted with permission from [495]. Copyright 2013 by IOP Publishing.

Ba-122 and Sr-122 tapes were fabricated by applying a standard *ex situ* PIT process [496]. First we prepared the precursor powders of Ba-122 and Sr-122 as shown in figure 106 [495]. Ba or Sr filings, K plates, Fe powder and As pieces were mixed to the nominal composition of Ba_0.6_K_0.4_Fe_2_As_2.1_ and Sr_0.6_K_0.4_Fe_2_As_2.1_ in an Ar gas atmosphere using a ball-milling machine and the materials were put into a Nb tube of 6 mm outer diameter and 5 mm inner diameter. The Nb tube was put into a stainless steel tube, both ends of which were pressed and sealed by arc welding in an Ar-gas atmosphere. In order to compensate for loss of elements, the starting mixture contained 10–20% excess K. The stainless steel tube was heat treated at 900 °C for 10 h and then cooled to a room temperature in a box furnace. After the heat treatment, the precursor was removed from the Nb tube and ground into powder with an agate mortar in a glove box filled with high purity argon gas. Figure 107 shows the XRD pattern of the precursor powder [495]. This analysis together with the magnetization measurement indicate that the precursor powder obtained by this method has fairly good quality.

The precursor powder was packed into an Ag tube of outer diameter: 6–8 mm, inner diameter: 3.5–4 mm and length: 50 mm, and the Ag tube was groove rolled into a wire with a rectangular cross section of ∼2 × ∼2 mm. The wire was then cold rolled into a tape using a flat rolling machine, initially into 0.8 mm in thickness followed by intermediate annealing at 800 °C for 2 h and then into 0.40 ∼ 0.20 mm in thickness. For some of the tapes we applied uniaxial pressing. For this uniaxial pressing, the flat rolled tape was cut into samples of 35 mm in length and the sample was sandwiched between two hardened steel dies and pressed. The uniaxial pressure was widely changed from ∼0.1 to ∼4 GPa. The flat rolled and uniaxially pressed tapes were subjected to a final sintering heat treatment at 850 °C for 2 ∼ 10 h to obtain Ba-122/Ag and Sr-122/Ag superconducting tapes. All the sintering heat treatments were carried out by putting the tapes into a stainless steel tube, both ends of which were pressed and sealed by arc welding in an Ar atmosphere. We also fabricated seven-filamentary tapes. A cold worked mono-filamentary wire with 1.3 mm diameter was cut into seven short wires with length of 40 mm. The seven wires were bundled together and put into another Ag tube, and the assemblage was cold worked into tape with intermediate annealing, uniaxially pressed, and finally heat treated under the same condition as mono-filamentary tapes.

#### 4.4.2. Critical current and microstructure

The transport critical current *I*_c_ at 4.2 K in magnetic fields were measured by the standard four-probe resistive method in liquid helium (4.2 K) using a 12 T superconducting magnet. The criterion voltage to measure *I*_c_ was 1 *μ*V cm^−1^. The transport critical current density, *J*_c_ was estimated by dividing *I*_c_ by the cross sectional area of the Ba-122 or Sr-122 superconducting core. Magnetic fields up to 12 T were applied parallel to the tape surface and perpendicular to the tape axis.

**Figure 108.**
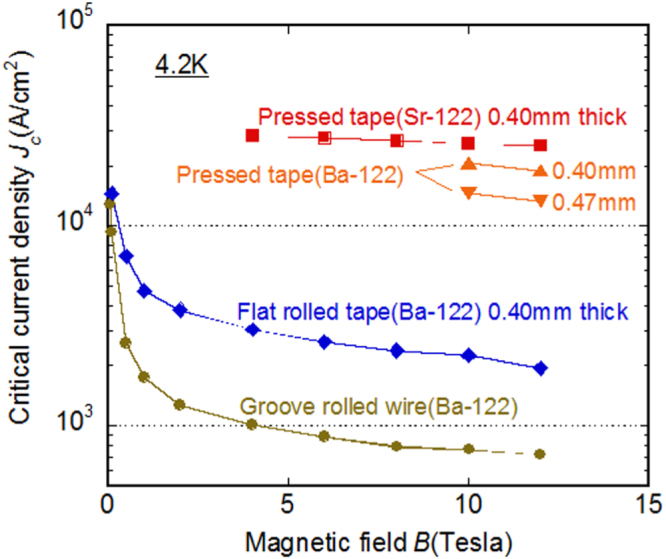
*J*_c_–*B* curves of uniaxially pressed Ba-122/Ag and Sr-122/Ag tapes [495, 497]. The data of groove rolled wire and flat rolled tape are also shown for comparison. Reprinted with permission from [495]. Copyright 2013 by IOP Publishing.

Figure 108 shows *J*_c_ versus magnetic field curves at 4.2 K of two uniaxially pressed Ba-122/Ag tapes with final tape thicknesses of 0.40 and 0.47 mm [495] and Sr-122/Ag tape with 0.40 mm thickness [497]. For comparison, the *J*_c_–*B* curves of a groove rolled Ba-122/Ag wire with a rectangular cross-section (∼2 × ∼2 mm) and a flat rolled tape (0.4 mm thickness) with no uniaxial pressing are also shown in the figure. All the wires and tapes were subjected to a final heat treatment of 850 °C for 10 h. The figure clearly indicates that the *J*_c_ increases with the process from groove rolling to uniaxial pressing. The *J*_c_ of the grooved rolled wire is as low as ∼10^3^ A cm^−2^ in applied magnetic fields. Similar low *J*_c_ values were reported for Ba-122 and Sr-122 round wires fabricated by the conventional PIT process [486, 487, 498]. A substantial *J*_c_ increase was obtained by the application of the cycles of flat rolling and subsequent heat treatment [494]. However, the most remarkable result in this figure is that another large *J*_c_ enhancement was obtained by the application of uniaxial pressing. All the pressed tapes show *J*_c_ over 10^4^ A cm^−2^ at 10 T, indicating that a high *J*_c_ is obtained with good reproducibility. The data in figure 108 suggest that the application of higher uniaxial pressure will result in higher *J*_c_ values. This can be realized as will be discussed later.

**Figure 109.**
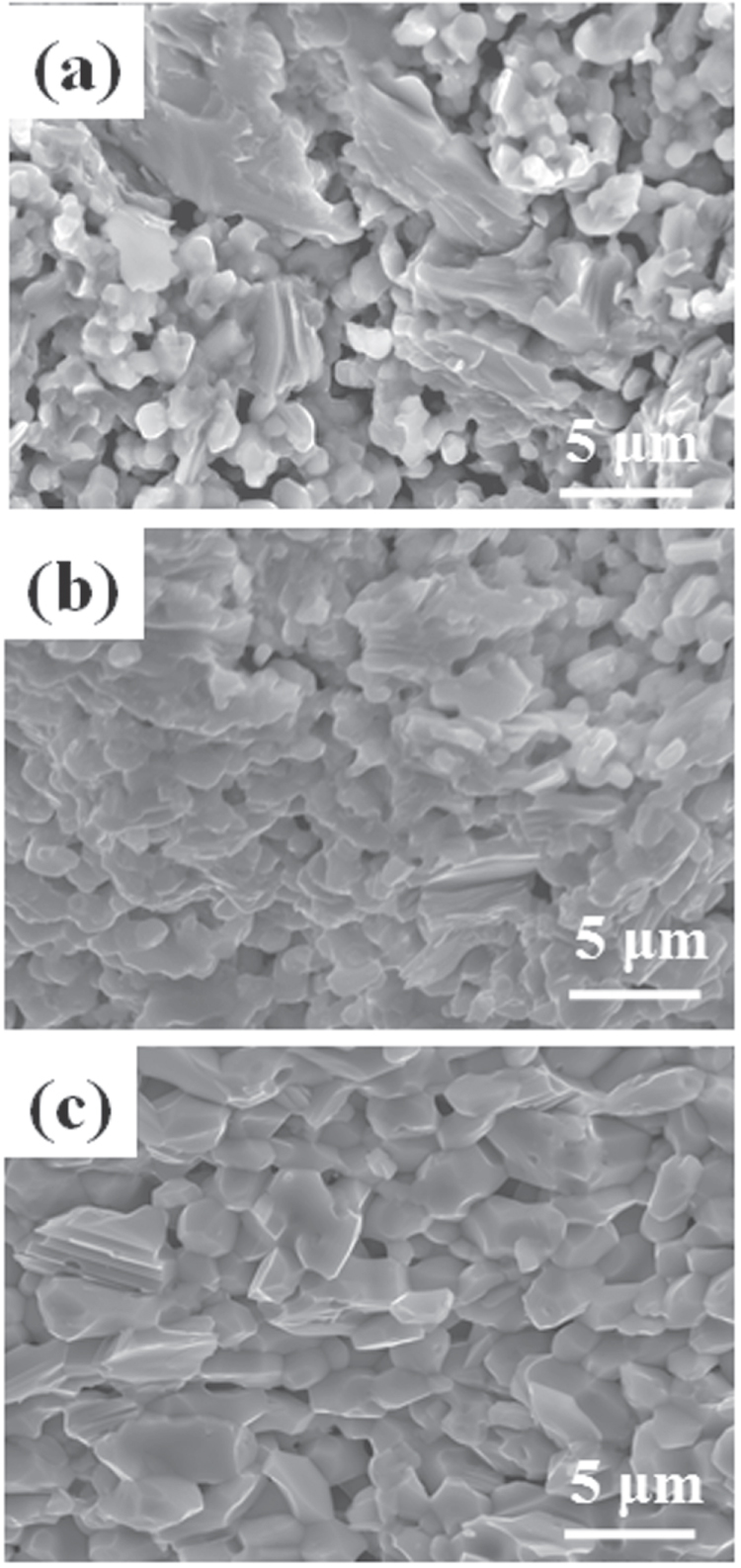
Grain structures of the Ba-122 core for the (a) groove rolled wire, (b) flat rolled tape and (c) uniaxially pressed tape. Reprinted with permission from [495]. Copyright 2013 by IOP Publishing.

**Figure 110.**
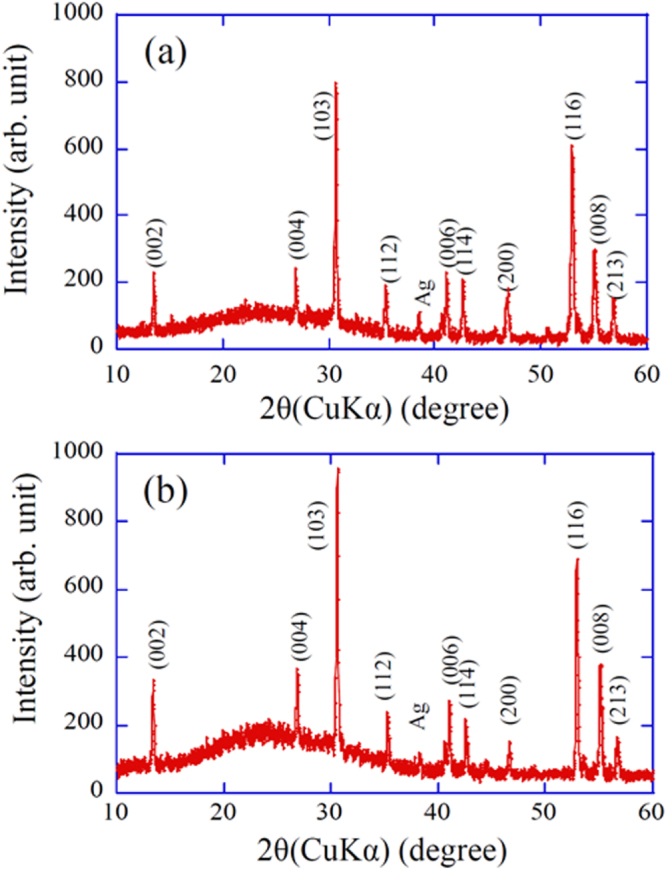
X-ray diffraction patterns of (a) flat rolled and (b) uniaxially pressed tapes. Reprinted with permission from [495]. Copyright 2013 by IOP Publishing.

In order to investigate the mechanism of *J*_c_ enhancement, we observed the microstructure change during the process. Fractured cross-sections of the wires and tapes were observed by SEM. Figures 109(a)–(c) show the grain structures of the Ba-122 core for the groove rolled wire, the flat rolled tape and uniaxially pressed tape, respectively [495]. All of the wires and tapes were finally heat treated at 850 °C for 10 h. The microstructure of the groove rolled wire shows non-uniformity in the grain size distributed widely from ∼1 to ∼10 *μ*m. The flat rolled and pressed tapes show more uniform grain structure, whose average size is a few micrometers. We consider that the cycles of deformation and subsequent heat treatment break up larger grains into smaller grains, resulting in the more uniform grain structure of the Ba-122 core. The difference of grain structure between the flat rolled tape and pressed tape is not significant, although a large *J*_c_ difference is obtained for rolled and pressed tapes. This large *J*_c_ difference can be explained by the difference of Ba-122 core density as will be discussed later. Figures 110(a) and (b) show XRD patterns of the Ba-122 core surface of the flat rolled and pressed tapes, respectively [495]. For the XRD observation the Ag sheath was peeled off. It should be noted that the relative intensities of the (00*l*) peaks of both tapes are not as high as those observed for the pressed precursor powder in figure 106 and show more random orientation. This is different from PIT processed Bi-2223/Ag tapes, in which the cycles of flat rolling and subsequent heat treatment produce stronger *c*-axis grain alignment due to the larger anisotropic morphology of the Bi-2223 crystal [499]. Our results for Ba-122/Ag tapes are also in contrast to the Fe-sheathed PIT Sr-122 tape [484, 489, 491], in which much stronger grain orientation was observed, similar to the Bi-2223 tape. This difference seems to be caused by the different sheath material and different processing parameters such as reduction ratio. Almost no difference of relative (00*l*) peaks was observed between rolled and pressed tapes, indicating that the grain orientation of the pressed tape is as low as the flat rolled tape. This is consistent with the grain structure shown in figures 109(b) and (c). From the almost same grain morphology and XRD pattern between the rolled and pressed tapes, the grain orientation can be ruled out as a possible origin of the large enhancement of *J*_c_ by the uniaxial pressing.

**Figure 111.**
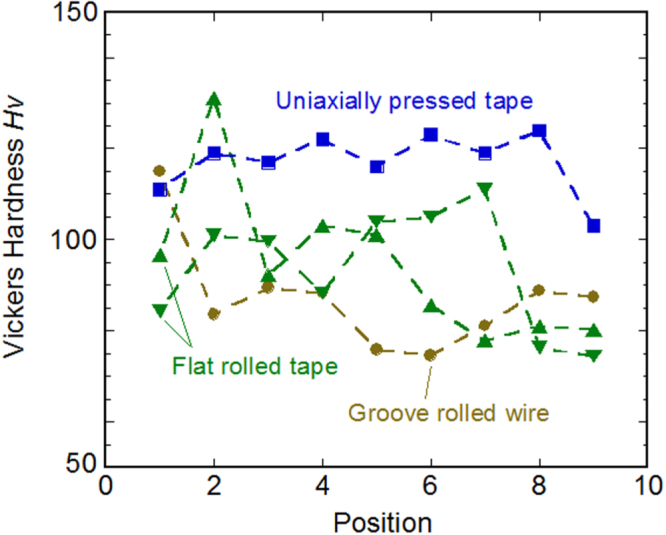
Vickers micro-hardness (*HV*) of the groove rolled wire, flat rolled tape and uniaxially pressed tape. Reprinted with permission from [495]. Copyright 2013 by IOP Publishing.

For the PIT processed superconducting wires and tapes, the density of the superconducting core seems to be one of the major factors that governs the *J*_c_ values of tapes and wires. Because it is difficult to directly measure the density of the thin superconducting core, researchers sometimes use Vickers hardness as an indicator of the density of the superconducting core [500, 501]. In this study of the superconducting wires and tapes fabricated by the PIT method, we performed the Vickers micro-hardness (*HV*) measurements of the Ba-122 core in order to investigate the influence of the core density on the *J*_c_ of our wire and tapes. Figure 111 shows the *HV* of the groove rolled wire, flat rolled tape and uniaxially pressed tape [495]. All the wires and tapes were finally heat treated at 850 °C for 10 h. The *HV* measurements were made on a polished transverse cross-section of each sample with 0.05 kg load and 10 s duration in a row at the center of the cross section. For the rolled tape, the *HV* at two different cross-sections was measured, because *HV* varies more widely in the rolled tape depending on the position. Although the scattering of *HV* is large, the figure clearly shows that the average *HV* increases with the progression of deformation process from groove rolling to uniaxial pressing. The average *HV* values of the groove-rolled wire, flat-rolled tape and uniaxially pressed tape are 87.1, 94.0 and 117, respectively. This increase of *HV* should be attributed to the increase of the Ba-122 superconducting core density. Thus, the increase of *J*_c_ with the deformation process in figure 108 can be explained by the increase of Ba-122 core density in the wires and tapes.

In the case of PIT processed Bi-2223/Ag tapes, it is reported that cold uniaxial pressing is more effective in enhancing transport *J*_c_ than flat rolling [502]. This can be attributed to a change in the micro-crack structure and more uniform deformation achieved by pressing rather than rolling. The uniaxial pressing of the Bi-2223/Ag tape introduces micro-cracks along the longitudinal direction of the tape, while in the flat rolled tape cracks appear along the transverse direction of the tape. During rolling, pressure varies along the arc of contact between the two rolls and the tape. The stress induced by the inhomogeneous pressure in the tape promotes the alignment of cracks transverse to the length direction [495]. However, in the case of uniaxial pressing, inhomogeneous deformation of the superconducting core occurs along the width of the tape, resulting in cracks along the direction of the tape length. In addition, the forces applied by uniaxial pressing are uniformly distributed perpendicular to the surface of the tape, thus resulting in higher and homogeneous compression. The higher uniform pressure reduces voids, improves texture formation, and thus further improves *J*_c_. It is supposed that the longitudinal cracks have no influence on the transport superconducting currents along the tape, while the transverse cracks should be barriers of the superconducting currents. This influence of cracks on the superconducting current flow along the tape was directly evidenced by magneto-optical imaging.

**Figure 112.**
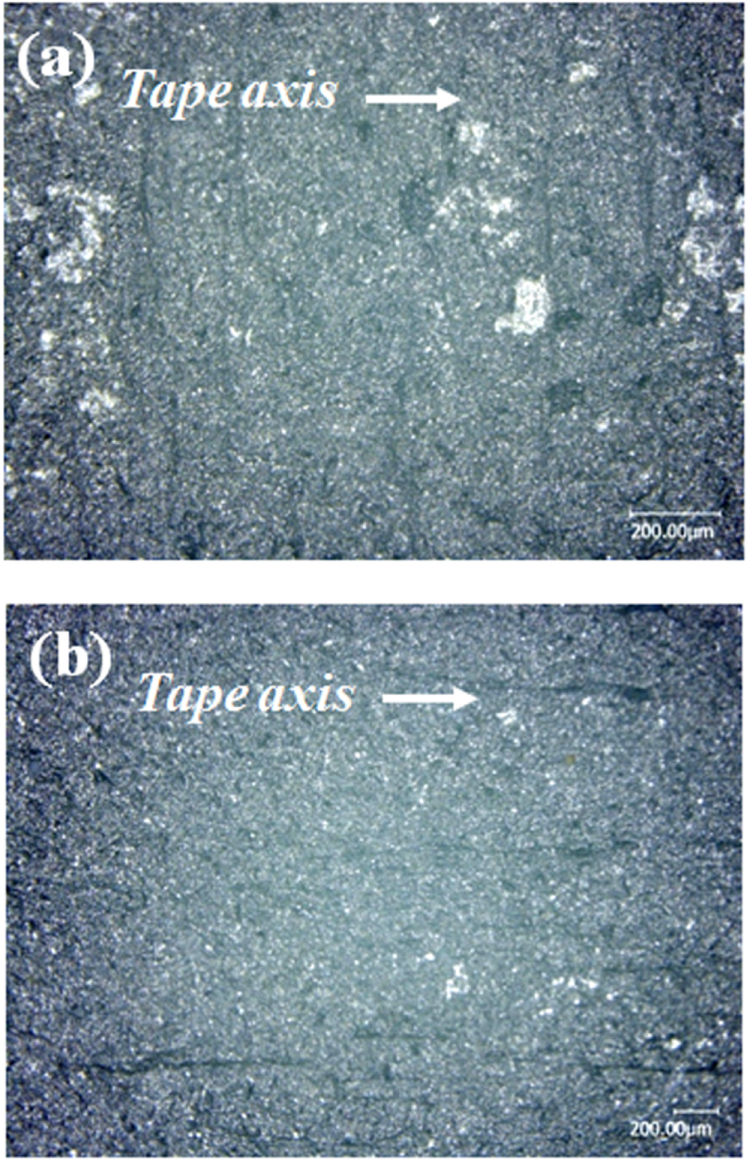
Optical microscopy images of (a) flat rolled and (b) uniaxially pressed tapes.

It is expected that cracks are also introduced in our Ba-122 tape and that the different direction of residual cracks is another important reason for the difference of *J*_c_ in the rolled and pressed tapes in addition to the difference of densification of superconducting cores. An apparent difference in crack structures between the rolled and pressed tapes was also observed. Figures 112(a) and (b) show SEM images of the cracks in the flat rolled and pressed tapes, respectively. The observation was carried out on the tape plane of the as-rolled or as-pressed tapes without subsequent heat treatment. The photographs were taken of the core surface after the Ag sheath was peeled off. As shown in figure 112(a), all cracks observed in the as-rolled tape run transverse to the tape length, while they run parallel to the tape length in the as-pressed tape as shown in figure 112(b). We consider that the observed difference in crack direction provides another key to elucidating the mechanism of the positive influence of applying uniaxial pressing [495]. It is speculated that the stress situation induced by the pressing, together with the heat treatment after pressing, acts so as to heal the transverse cracks produced by the previous rolling process, which run transverse to the tape length and reduce the effective cross-sectional area for superconducting current flow along the tape length.

#### 4.4.3. Effects of high uniaxial pressure and degree of flat rolling

**Figure 113.**
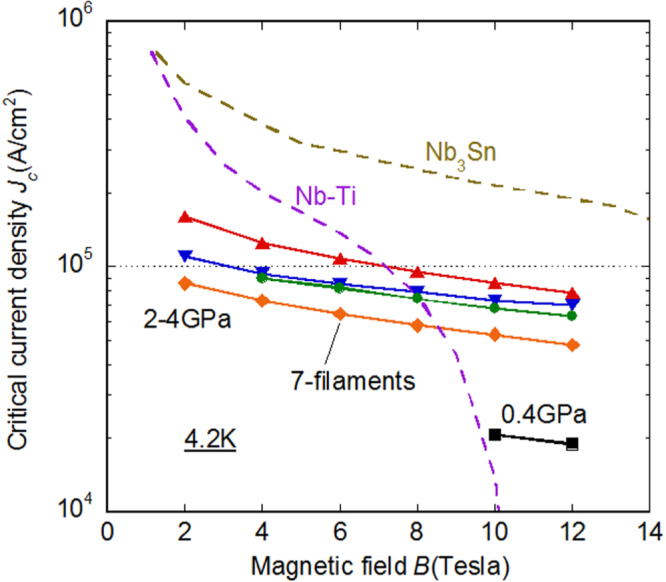
*J*_c_–*B* curves at 4.2 K of uniaxially pressed Ba-122/Ag tapes. Pressure was changed from 2 to 4 GPa. For comparison the data of Ba-122 tape (figure 108) and Nb–Ti and Nb_3_Sn commercial wires are also included in the figure. Reprinted with permission from Macmillan Publishers Ltd: [503], Copyright 2014.

As mentioned before, *J*_c_ significantly enhanced by the uniaxial pressing under 0.4 GPa. This suggests that much higher *J*_c_ values can be obtained by increasing the uniaxial pressure. Thus, we increased the uniaxial pressure up to 4 GPa [503]. Figure 113 shows *J*_c_–*B* curves of the single and seven filamentary Ba-122/Ag tapes uniaxially pressed under 2–4 GPa before final heat treatment at 850 °C for 2–4 h [503]. The *J*_c_–*B* curves of the Ba-122 tape pressed under 0.4 GPa, commercial Nb–Ti and Nb_3_Sn wires are also shown for comparison. Much higher *J*_c_ values than the tape pressed under 0.4 GPa are obtained by increasing the uniaxial pressure. *J*_c_ values of all the pressed Ba-122 tapes show very small field dependence as observed for the tapes shown in figure 108, and *J*_c_ values well above 5.0 × 10^4^ A cm^−2^ in 10 T are obtained, indicating that a high *J*_c_ values are obtained with good reproducibility. It should be noted that a high *J*_c_ exceeding the practical level of 10^5^ A cm^−2^ at 4.2 K is obtained in magnetic fields up to 6 T for mono-filamentary tape and *J*_c_ maintains a high value of 8.6 × 10^4^ A cm^−2^ in 10 T. The seven-filamentary tape also sustains *J*_c_ as high as 5.3 × 10^4^ A cm^−2^ at 10 T. These high *J*_c_ values highlight the importance of uniaxial pressing for enhancing the *J*_c_ of Ba-122 tape conductors.

The *J*_c_ values of the Ba-122/Ag tapes exceed the values of the Nb–Ti conductor in magnetic fields higher than ∼8 T. Furthermore, in comparison with the Nb_3_Sn, the *J*_c_ of Ba-122 tapes show very small magnetic field dependence. As will be described later, Ba-122 surpasses the *J*_c_ performance of Nb_3_Sn in high magnetic fields. Our previous study [486, 487] indicates that the magnetic field dependence of *J*_c_ at 4.2 K of the flat rolled Ba-122 tape is very small, comparable to that of the melt-textured Bi-2212/Ag tape [504]. These results suggest that the Ba-122 superconducting wires will be competitive with well-established Nb-based superconductors and Bi-based oxide for high magnetic field applications in the near future.

**Figure 114.**
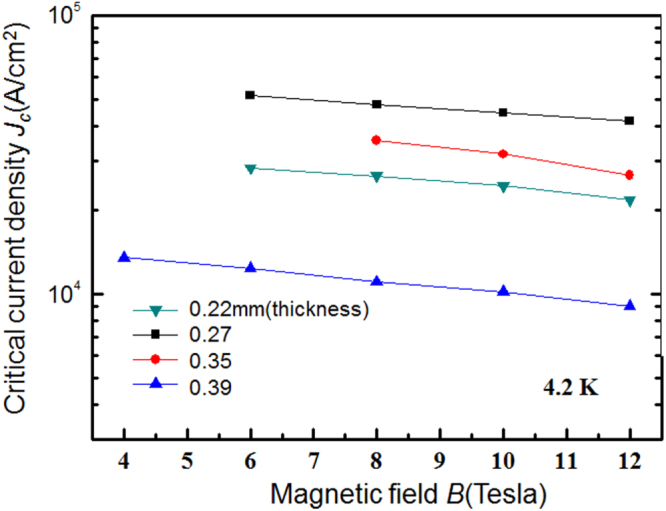
*J*_c_–*B* curves at 4.2 K of flat rolled Ba-122/Ag tapes. Reprinted with permission from Macmillan Publishers Ltd: [503], Copyright 2014.

The large reduction of tape thickness only by applying flat rolling is also found to be effective in enhancing *J*_c_ values [503]. Figure 114 shows the *J*_c_–*B* curves of the flat rolled Ba-122 tapes. These *J*_c_ values are much higher than that of the flat rolled tape shown in figure 108. Figure 114 clearly indicates that *J*_c_ significantly increases when the tape thickness is reduced by flat rolling. *J*_c_ reached a maximum value of 4.5 × 10^4^ A cm^−2^ at 10 T when the thickness of the tape reduced to 0.26 mm. But when the thickness is reduced further, degradation in *J*_c_ was observed. However, further improvement in *J*_c_ values could be achieved for thinner tapes when we applied uniaxial pressing instead of flat rolling, as shown in figure 113. SEM observation of these thin tapes indicates that the transverse micro-cracks observed in figure 112 appear to decrease in number with decreasing tape thickness. With decreasing tape thickness, the stress and strain distribution in the rolling process seems to become similar to those in uniaxial pressing when the rolling diameter is constant, leading to the disappearance of transverse micro-cracks. This is one of the reasons why *J*_c_ increases with decreasing tape thickness in the flat rolling process.

**Figure 115.**
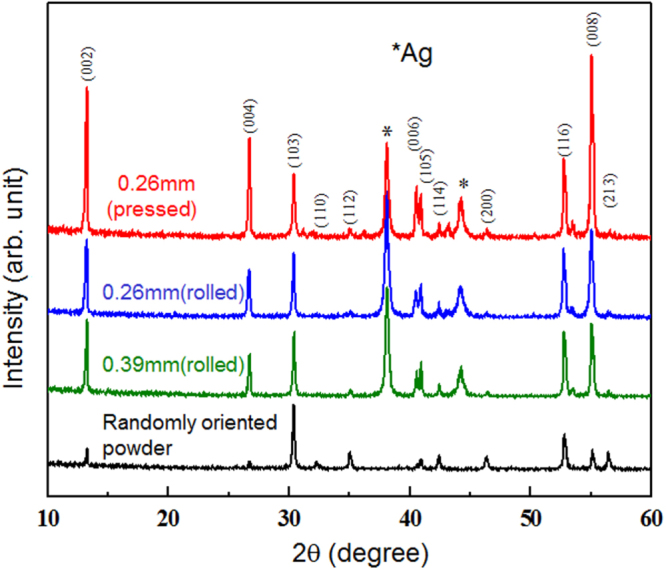
X-ray diffraction patterns of rolled and pressed Ba-122/Ag tapes. The data of Ba-122 powder with random grain orientation are also shown for comparison. Reprinted with permission from Macmillan Publishers Ltd: [503], Copyright 2014.

Figure 115 shows XRD patterns of the flat rolled and uniaxially pressed Ba-122/Ag tapes shown in figures 113 and 114 [503]. The data of randomly orientated Ba-122 precursor powder are also included in the figure for comparison. All the tapes consist of a main phase, Ba_1−*x*_K_*x*_Fe_2_As_2_, however, Ag peaks from the sheath material are also detected. The relative intensities of the (00*l*) peaks with respect to that of the (103) peak in all tapes are strongly enhanced, when compared to the randomly oriented powder, indicating that a well-defined *c*-axis grain orientation is obtained by flat rolling and pressing. The relative intensities of the (00*l*) peak are higher than those of the flat rolled tape in figure 110(a), indicating that a large degree of flat rolling is effective in enhancing the *c*-axis grain orientation. However, it remains at almost the same level when decreasing the tape thickness below 0.39 mm, as shown in figure 115, suggesting that the grain orientation is hardly further improved by any further flat rolling process. Therefore, grain orientation can be ruled out as a possible mechanism of *J*_c_ enhancement with decreasing tape thickness in figure 114. In contrast, a higher relative intensity of (00*l*) peaks was observed in the pressed tape. This relative intensity in figure 115 is much higher than that of the uniaxially pressed tape in figure 110(b). Thus, the high uniaxial pressure is effective in enhancing the *c*-axis grain orientation. These results indicate that high uniaxial pressure is more effective in improving the *c*-axis grain orientation than the large degree of flat rolling. However, it should be noted that the degree of *c*-axis grain orientation in our pressed tapes is still lower than that in the Fe sheathed PIT processed Sr-122 tapes [489, 491]. This suggests that the grain orientation could be further enhanced by optimizing the processing parameters of the tape conductors or by using harder sheath materials.

**Figure 116.**
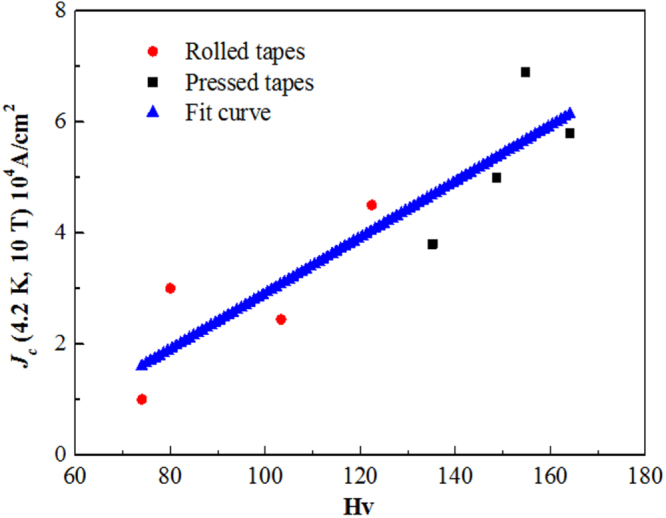
*J*_c_ at 4.2 K and 10 T as a function of Vickers hardness *HV* for the flat rolled and uniaxially pressed Ba-122/Ag tapes. Reprinted with permission from Macmillan Publishers Ltd: [503], Copyright 2014.

We evaluated the effect of flat rolling on *HV* and *J*_c_. Both the *HV* and *J*_c_ clearly increase with decreasing tape thickness. This indicates that in addition to the disappearance of transverse cracks, the increase of Ba-122 density might be another reason for *J*_*c*_ enhancement by the hard flat rolling. Figure 116 shows *J*_c_ (10 T, 4.2 K) as a function of *HV* for both rolled and pressed tapes [503]. *J*_c_ increases with increasing *HV* and, hence, with increasing Ba-122 core density. A strong correlation between the hardness of the tapes and *J*_c_ was observed. With increasing hardness, the *J*_c_ of the Ba-122 core increased, however the hardness and *J*_c_ of flat rolled tapes do not surpass the hardness and *J*_c_ of the uniaxially pressed tapes, respectively. This suggests that uniaxial pressing of tapes yields much better *J*_c_*–H* characteristics than flat rolling [495]. However, it seems that there is no discontinuity in the *J*_c_*–HV* curve between flat rolling and uniaxial pressing. This suggests that there is no essential difference in the superconducting current limiting mechanism between flat rolling and uniaxial pressing. Thus, we can expect that high *J*_c_ values comparable to or higher than pressed tapes can be obtained only by applying a high degree of flat rolling. As is well known, flat rolling is more convenient and useful than uniaxial pressing for the fabrication of practical level long tape conductors.

**Figure 117.**
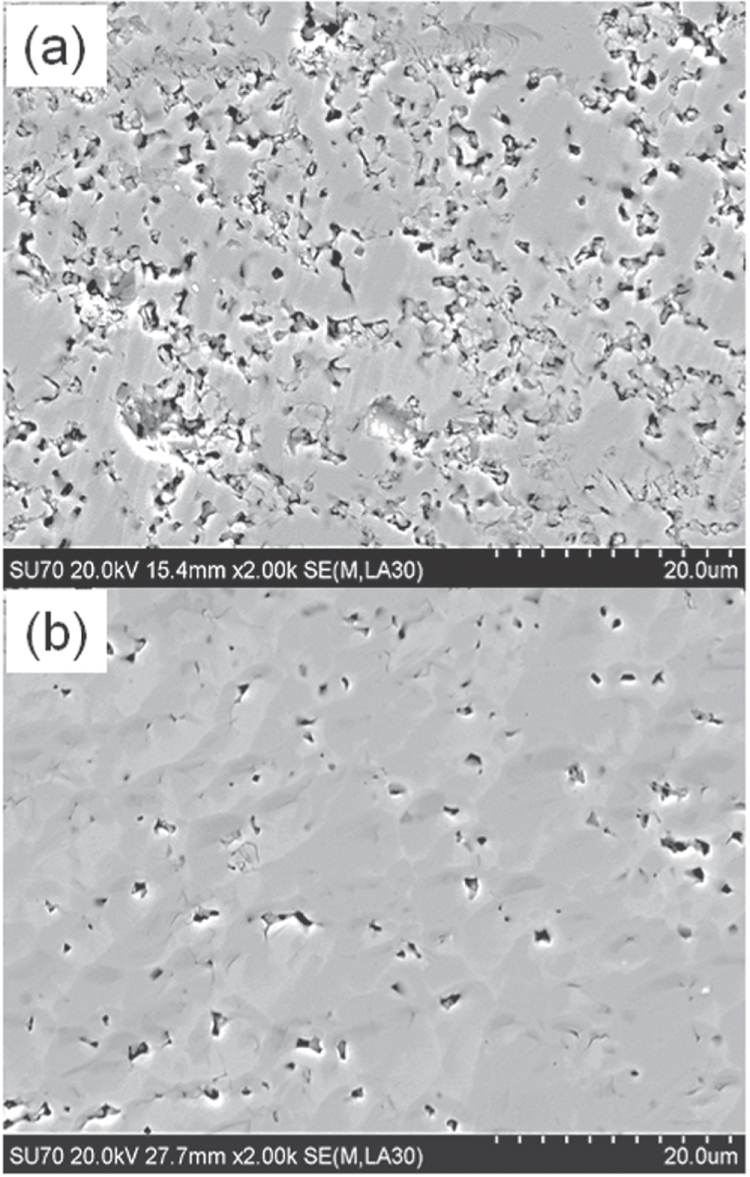
Scanning electron microscopy images of (a) flat rolled and (b) uniaxially pressed Ba-122/Ag tapes.

Figures 117(a) and (b) show typical SEM images of the polished surface for the flat rolled and uniaxially pressed Ba-122/Ag tapes. These observations were carried out on the tape plane of the tapes. Although rolling can reduce voids and improve the density of the Ba-122 core, the microstructures are still porous and quite inhomogeneous. In contrast, the pressed tapes with higher hardness and *J*_c_ apparently have a denser and more uniform microstructure than the rolled tapes with lower hardness and *J*_c_. This result is consistent with the *HV* analysis in figure 116. Generally speaking, the pressure introduced by the uniaxial pressing is higher and more uniform than that introduced by the rolling. Thus, the microstructure in the pressed sample is denser and more uniform than that in the rolled one.

**Figure 118.**
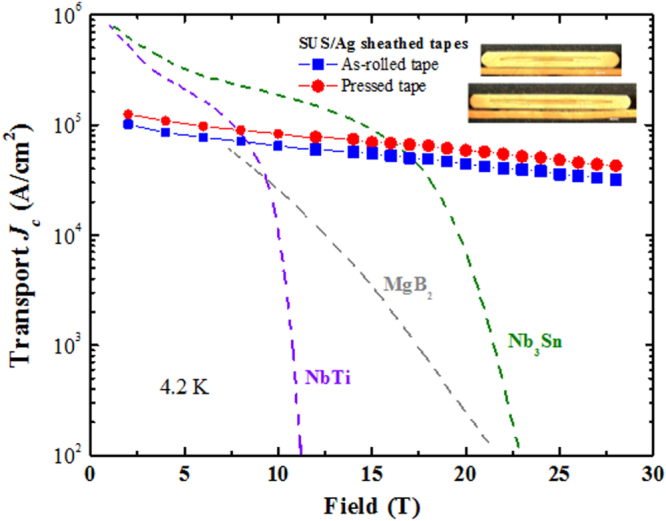
*J*_c_ versus magnetic field curves at 4.2 K of double sheathed Ba-122 tapes fabricated with rolling and pressing. For comparison, MgB_2_ wires are shown in the figure. Reprinted with permission from [505]. Copyright 2015 by IOP Publishing.

In the previous sections, we used an Ag tube as the metal sheath of Ba-122 tapes. The Ag sheath was completely annealed by the heat treatment and became very soft after the heat treatment. Thus, the Ag sheathed Ba-122 tape is not practical from the aspect of mechanical strength. In order to solve this problem we fabricated Ba-122 tape conductors applying a new sheath structure of double sheaths; stainless steel as an outer sheath and Ag as an inner sheath [505]. The inner Ag sheath was used to avoid the reaction between stainless steel and Ba-122. Figure 118 shows *J*_c_*–B* curves at 4.2 K of the double sheathed Ba-122 tapes fabricated with flat rolling and uniaxial pressing. The inset shows transverse cross sections of flat rolled (top) and pressed (bottom) tapes. For comparison, data of commercial superconductors, Nb*–*Ti and Nb_3_Sn, are also shown in the figure. We found that the rolled tapes show *J*_c_ values of 7.7 × 10^4^ A cm^−2^ at 4.2 K and 10 T with high homogeneity. These *J*_c_ are the highest values reported so far for IBSC tapes and wires fabricated by a scalable rolling process. It should be noted that the use of hard stainless steel as the outer sheath increases not only the mechanical strength of the tape but also the density of Ba-122 core. The application of uniaxial pressing to the double sheathed tape further increased *J*_c_ at 4.2 K and 10 T up to 9.0 × 10^4^ A cm^−2^. The transport *J*_c_*–B* curves for both rolled and pressed tapes show extremely small magnetic field dependence and the *J*_c_ values exceed 3 × 10^4^ A cm^−2^ in 28 T, which are much higher than those of low-temperature commercial superconductors. The microstructure investigations indicate that such high *J*_c_ values were achieved by the higher density of the core, more uniform deformation of the Ba-122 core and higher degree of grain orientation [505]. These results indicate that the combination of the double sheath and rolling is very promising for fabricating long Ba-122 tape conductors for a high magnetic field magnet which can generate fields higher than 20 T.

#### 4.4.4. Temperature dependence of critical current density

The temperature dependence of the transport critical current was measured using a 10 T split type superconducting magnet with a variable temperature insert cryostat that can control the temperature between 10 and 40 K [497]. We also investigated the magnetic field orientation dependence of *J*_c_ for Sr-122/Ag tapes to evaluate the *J*_c_ anisotropy of the tape conductors.

**Figure 119.**
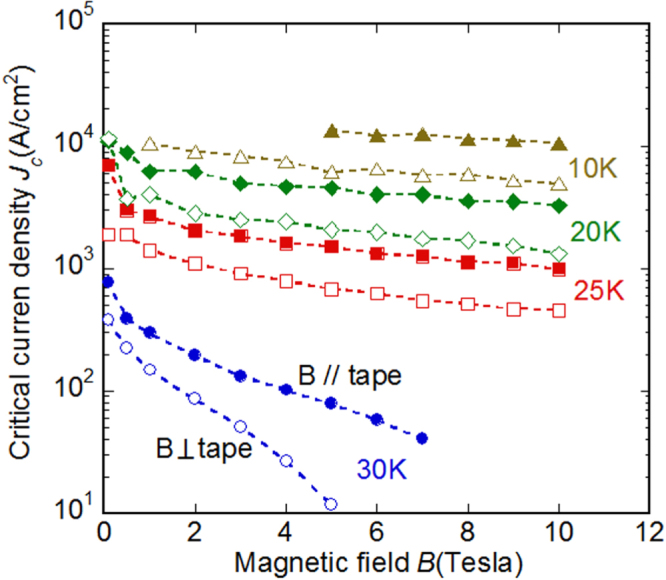
Temperature dependence of *J*_c_*–B* curves of the Sr-122/Ag tape. A magnetic field was applied parallel (perpendicular to the tape axis) and perpendicular to the tape surface. Reprinted with permission from [497]. Copyright 2014 by IOP Publishing.

Figure 119 shows *J*_c_–*B* curves of a uniaxially pressed Sr-122/Ag wire at four different temperatures of 10, 20, 25 and 30 K in external magnetic fields applied parallel and perpendicular to the tape surface [497]. *J*_c_ was estimated by dividing the *I*_c_ by the cross sectional area of the Sr-122 superconducting core of the tape conductor. The uniaxial pressure was 0.4 GPa, which brought a thickness reduction ratio of 20%. At 20 K, *J*_c_ value was almost 10^4^ A cm^−2^ at 0 T. This *J*_c_ decreases gradually with increasing magnetic field applied parallel to the tape surface. *J*_c_ values in perpendicular fields are lower than those in parallel fields; however, field dependence is still small. The *J*_c_–*B* curves at temperatures below 25 K show small slopes, nearly equal to those at 10 K, suggesting that the *ex situ* Sr-122/Ag tape has a high potential in high magnetic fields at temperatures below 25 K. Furthermore, even at 30 K, which is close to the *T*_c_ of this superconducting material, *J*_c_ was still observed in fields up to around 7 T. These results are due to the high *B*_c*2*_ values of Sr-122. The *J*_c_ value in a magnetic field applied perpendicular to the tape surface was about half the value in the magnetic field applied parallel to the tape surface at temperatures below 20 K. This *J*_c_ anisotropy of the Sr-122/Ag tape is smaller than those of Bi-2212/Ag and Bi-2223/Ag tape conductors. This result also suggests that the Sr-122/Ag tape shows lower anisotropy in *J*_c_ and is useful for practical magnet applications.

**Figure 120.**
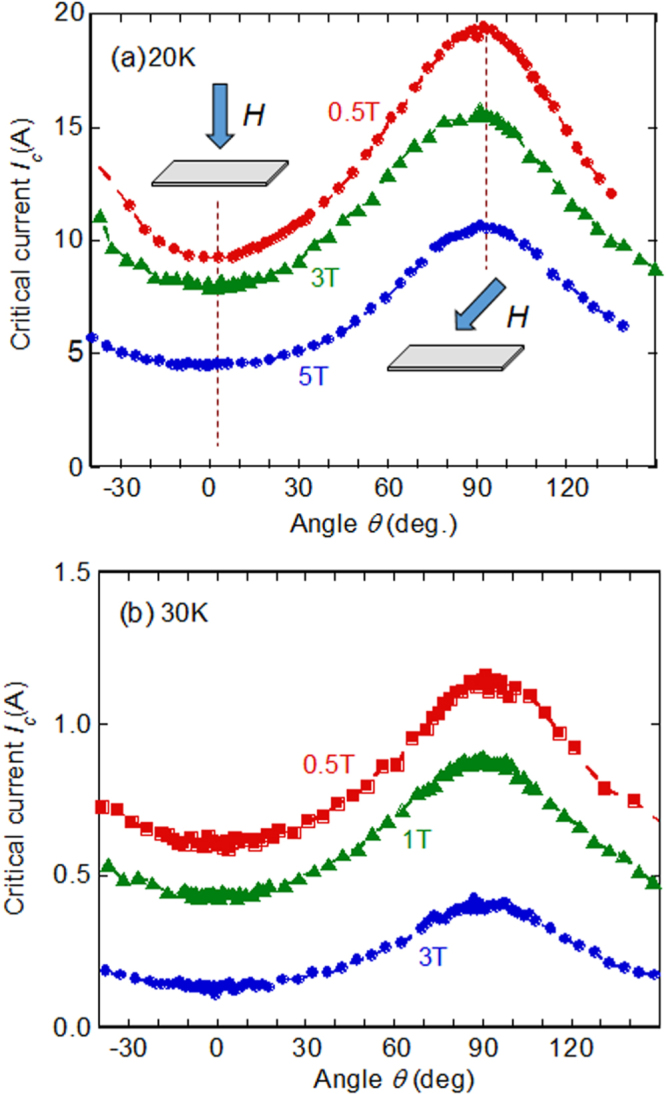
*I*_c_ at 20 K and 30 K as a function of strength and direction of external magnetic field. Reprinted with permission from [497]. Copyright 2014 by IOP Publishing.

Figures 120(a) and (b) show *J*_c_ of a pressed Sr-122/Ag tape as a function of external magnetic field directions to the tape surface at 20 and 30 K, respectively [497]. Zero and 90° in the figure correspond to the magnetic field directions perpendicular and parallel to the tape surface, respectively. Maximum and minimum *J*_c_ values were obtained at around 0° and 90° in every magnetic field. This angular dependence is similar to the anisotropy of high-*T*_c_ oxide superconducting tapes, Y-123(coated conductor), 2212/Ag and B-2223/Ag tapes. The ratio between maximum and minimum *J*_c_ values (*J*_c_ anisotropy) is almost two at every magnetic field up to 3 T. However, the *J*_c_ anisotropy increased with increasing magnetic field as shown in figure 119.

#### 4.4.5. Summary and future prospects

Among the many fabrication stages of superconducting tapes, flat rolling has been commonly used to densify and realize the grain orientation of the superconducting core [494, 496, 505]. Our results showed a large increase in *J*_c_ due to the improvement of the Ba(Sr)-122 core density and preferred orientation in the initial step by the rolling process. Upon further rolling to smaller tape thicknesses, degradation of critical current density was observed due to the introduction of transverse micro-cracks. However, a larger reduction of tape thickness by flat rolling tends to decrease the transverse micro-cracks and to enhance *J*_c_ values. This suggests that higher *J*_c_ can be realized by only applying flat rolling.

Furthermore, when the tape was uniaxially pressed, *J*_c_ values were significantly increased by another improvement of the core density and grain orientation. Excellent transport *J*_c_ values of ∼10^5^ A cm^−2^ under magnetic fields up to 6 T were obtained in uniaxially pressed Ba-122/Ag tapes. A higher core density and more grain orientation are responsible for this high *J*_c_ performance in the pressed samples. The use of a hard metal as the outer sheath of a double sheathed tape is also effective in increasing the core density and *J*_c_ values. However, it should be emphasized that practical applications of uniaxial pressing for the manufacture of long tapes require specialized machines for continuous pressing of the tape. Fortunately, there have been successful attempts at producing long Bi-2223/Ag wires by periodic pressing [506] and eccentric rolling [507], which might be also applied to the production of long Ba-122 wires with high transport *J*_c_.

Ba(Sr)-122/Ag tape conductors show very small field dependence of *J*_c_ with small *J*_c_ anisotropy. With further improvements in the critical current density and wire fabrication technology, Ba(Sr)-122 superconductors will have a very promising future in high-field applications.

## 5. New functional materials and devices found within this project

Exploration of novel superconductors needs a non-conventional approach in various aspects, such as the material system and synthetic processes as high pressure and electric field effect. As a result, there should be a much higher probability than other materials research to find new functionalities or to encounter new phenomena during the research [508]. A well-known example is the discovery of high-performance thermoelectric properties in NaCo_2_O_4_ in the course of comparative study of high-*T*_c_ cuprates with layered cobaltites [509]. This feature comes from the richness of materials in which we are engaging.

In this project, this team proceeded exploration of new superconductors along with seeking new functional materials. Some of representative achievements are briefly described in this section.

### 5.1. Catalytic activity for ambient pressure NH_3_ synthesis

Ammonia is the simplest active nitrogen molecule and one of the most commercially produced chemicals, reaching 160 million tons per year. While industrial ammonia synthesis from N_2_ and H_2_ is conducted using the Haber*–*Bosch process using iron-based catalysts [510] at 400*–*600 °C and 20*–*40 MPa, such high reaction temperatures are disadvantageous with respect to the equilibrium and exothermic reaction (46.1 kJ mol^−1^) of ammonia synthesis. The rate-determining step of ammonia synthesis is cleavage of the N≡N bond, because the bond energy is extremely large (945 kJ mol^−1^). Transition metals such as Fe or Ru are indispensable for the promotion of N≡N bond cleavage, in addition to electron donors that provide electrons to the transition metals. An N_2_ molecule is fixed to form a bond with a transition metal by donating electrons from its bonding orbitals and accepting electrons to the antibonding *π* orbitals (back-donation) [511]. This back-donation is effectively enhanced by electron donors, which further weakens the N≡N bond and results in the cleavage of N_2_. We assume electron injection is essentially indispensable to enhancing the efficiency of ammonia synthesis using Fe or Ru-catalysts. However, the situation is not so simple, i.e., it is extremely difficult to realize a low work function and chemical and thermal stability; both are generally incompatible. Although the catalytic activity of Ru is drastically enhanced by adding alkali or alkaline earth metals with small work functions [512], these metals are practically inapplicable for ammonia synthesis because these metals are so chemically active that the reaction with the produced ammonia and/or N_2_ forms metal nitrides and amides.

**Figure 121.**
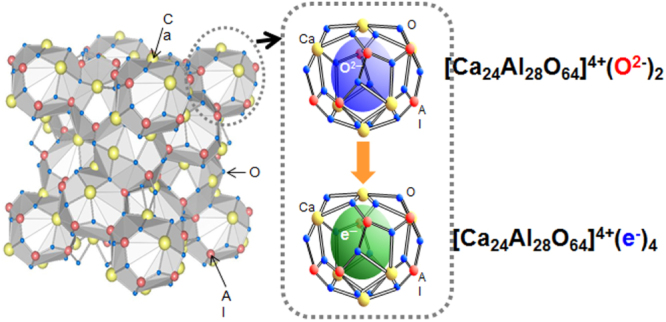
Crystal structure of 12CaO · 7Al_2_O_3_(C12A7). C12A7 has a cubic symmetry and two formula units with the chemical composition of Ca_24_Al_28_O_64_, and is an insulator with a bad gap of ∼7 eV. Stoichiometric C12A7 (C12A7:O^2−^) accommodates two O^2−^ ions in two cages out of 12 cages constituting a unit cell to preserve the electro-neutrality of the positively charged cage walls and its chemical formula may be expressed as [Ca_24_Al_28_O_63_]^4+^(O^2−^)_2_. All of these in-cage oxygen ions may be replaced with electrons keeping the original case structure by an appropriate chemical treatment. The resulting material with a formula [Ca_24_Al_28_O_63_]^4+^(4e^−^) may be regarded as an electride. C12A7 electride, abbreviated as C12A7:e, is a metallic conductor and exhibits a metal*–*superconductor transition at 0.2*–*0.4 K.

We examined the catalytic activity of a stable electride, C12A7:e^−^ as an efficient promoter with high electron donating power and chemical stability for a Ru catalyst utilizing unique properties of this material, i.e., low work function (2.4 eV) comparable metal potassium but chemical inertness [513]. Electride is an ionic crystal in which electrons serve as anions. 12CaO · 7Al_2_O_3_ (C12A7), which is a constituent of commercial alumina cement, works as complexant to electrons and the resulting material became the first electride that is stable at temperatures above room temperature and at ambient atmosphere [514, 515]. As shown figure 121, the unit cell of C12A7 has a positively charged framework structure composed of 12 sub-nanometer-sized cages which are connected to each other by sharing a mono-oxide layer to embrace 2O^2−^ in the two cages as the counter anions to compensate the electro-neutrality. Chemical reduction processes are used to inject four electrons into four of the 12 cages by extracting two O^2−^ ions accommodated in the cavities as counter anions to compensate for the positive charge on the cage wall. The resultant chemical formula is represented by [Ca_24_Al_28_O_64_]^4+^(e^−^)_4_. The injected electrons occupy a unique conduction band called ‘the cage conduction band’ (CCB), which is derived from the 3-dimensionally connected cages by sharing an oxide monolayer, and can migrate through the thin cage wall by tunneling, which leads to metallic conduction (about 1500 S cm^−1^ at room temperature). This electron-trapped cage structure of the bulk is retained up to the top surface if the sample is appropriately heated. Such electrons encapsulated in the cages of C12A7:e^−^ can be readily replaced with a hydride ion (H^−^) by heating in H_2_ gas. The incorporated H^−^ ions desorb as H_2_ molecules at about 400 °C, leaving electrons in the positively charged framework of C12A7: the incorporation and release of H^−^ ions on C12A7:e^−^ are entirely reversible. Please note that the electride formation and reversible storage ability of H^−^ totally originate from the unique crystal structure of C12A7 described above. Such formation was impossible for other oxides bearing Al_2_O_3_ and CaO.

We found that C12A7:e^−^ exhibits a superconductor transition at 0.2*–*0.4 K depending on the carrier concentration under an ambient atmosphere [516]. Although *T*_c_ is very low, this is the first *s*-metal superconductor under ambient pressure. Alkali and alkaline-earth metals do not show superconductivity under ambient pressure but some high pressure phases exhibit *T*_c_. We found the crystal structure of C12A7 is similar to that of superconducting phases and considered that the admixture of Ca *d*-orbitals to the *s*-orbital of the cage electrons is the origin of the superconductivity of C12A7:e^−^ [517].

**Figure 122.**
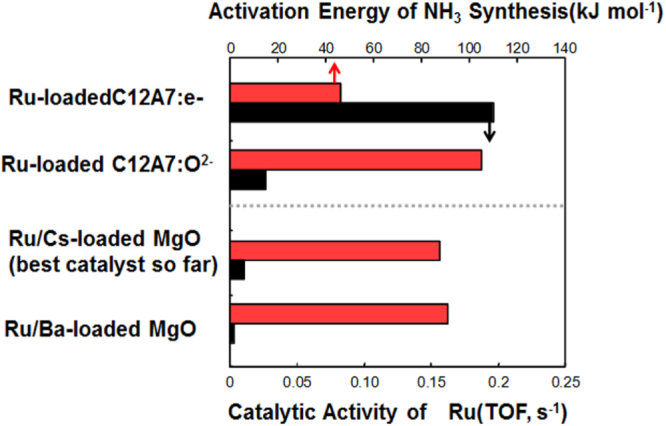
Comparison of Ru-loaded catalysts for ammonia synthesis. The reaction conditions; catalyst 0.3 g, flow rate of H_2_/N_2_ (3:1) gas 60 ml min^−1^, temperature 400 °C, and pressure 0.1 MPa. Reprinted with permission from Macmillan Publishers Ltd: [108], Copyright 2012.

We deposited nano-sized Ru particles to C12A7:e^−^ powders using the chemical vapor deposition method to enhance the amount of captured N_2_ on their surfaces. Figure 122 shows a comparison in catalytic activity for NH_3_ synthesis [108]. The activation energy over Ru-loaded C12A7:e^−^ is reduced to almost half of that over other Ru-catalysts and the turn-over-frequency (TOF, the measure of catalytic activity per active site) is larger by an order of magnitude than that of the latter including the best Ru-based catalyst (Ru/Cs-loaded MgO). The excellent activity of C12A7:e^−^ demonstrates the electron-donating effect plays an essential role in NH_3_ synthesis. Very recently, it has been clarified by examining the kinetics of the isotope exchange reaction between ^14^N and ^15^N with DFT calculations that the rate determining step of NH_3_ synthesis over Ru-loaded C12A7:e^−^ shifts from dissociation of the N≡N bond to the N*–*H bond formation process [518].

**Figure 123.**
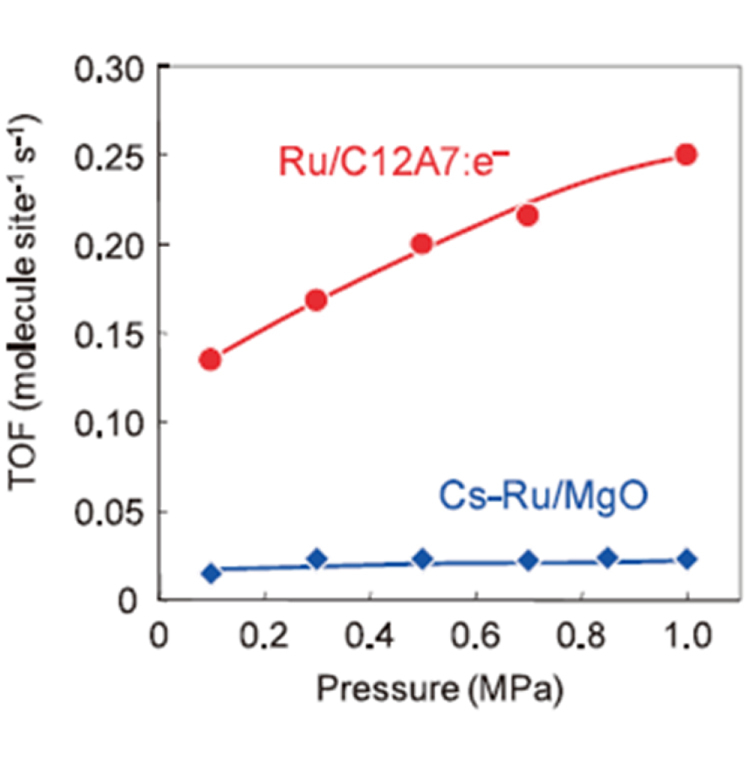
Catalytic activity of Ru-loaded C12A7:e^−^ as a function of partial hydrogen pressure. Data of a representative Ru-catalyst are shown for comparison. Note that the activity is proportional to partial H_2_ pressure. There is no serious H_2_-poisoning which is a common drawback of Ru-catalysts. Reprinted with permission from Macmillan Publishers Ltd: [108], Copyright 2012.

**Figure 124.**
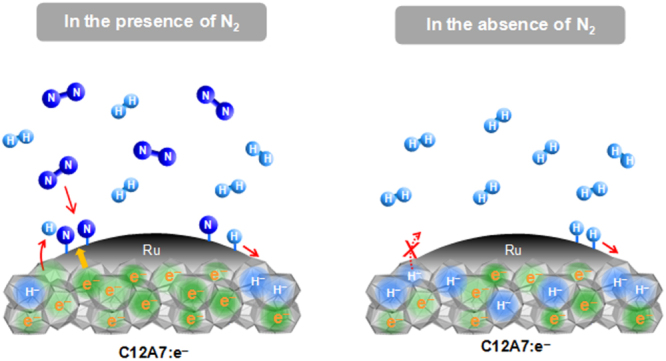
Tentative reaction mechanism to explain suppression of H_2_-poisoning in Ru-loaded C12A7:e^−^. Reprinted with permission from Macmillan Publishers Ltd: [108], Copyright 2012.

Another surprising finding is that Ru-loaded C12A7:e^−^ does not exhibit hydrogen-poisoning [108] as shown in figure 123. An obstacle for industrial ammonia synthesis with a Ru-loaded catalyst is hydrogen-poisoning in high H_2_ pressure. Because NH_3_ synthesis over Ru catalysts in general degrades by hydrogen adatoms formed on Ru surfaces, the reaction order for H_2_ on Ru catalysts often approaches −1 (i.e., the reaction rate *decreases* with the partial pressure of H_2_). Such H_2_ poisoning on Ru catalysts is a serious obstacle for industrial ammonia production, which requires high pressure conditions to collect the resulting NH_3_ in the form of liquid (NH_3_ easily becomes liquid above 0.85 MPa at room temperature). The chemical industry is therefore currently searching for a supported Ru catalyst that promotes N_2_ dissociation but suppresses H_2_ poisoning. Figure 124 shows a tentative mechanism for ammonia synthesis over Ru-loaded C12A7:e^−^. The robustness of Ru-loaded C12A7:e^−^ to H_2_-poisoning comes from its ability of reversible H-storage and release. C12A7:e^−^ reacts with H_2_ to form C12A7:H^−^ in which H is incorporated into the cage as H^−^. Although this reaction over Ru-free C12A7:e^−^ is irreversible in temperature range below ∼450 °C, the H-release temperature is drastically reduced to ∼300 °C [519]. Desorption of H^−^ to react with activated nitrogen species on Ru surfaces to form N*–*H bonds, leaving an electron in the cage. We consider that hydrogen poisoning on Ru-surfaces is suppressed by preferential entrapping of a H adatom in the cage to form H^−^ and the incorporated H^−^ in the cage is activated by a strong driving force for stable N*–*H bond formation.

### 5.2. Bipolar oxide thin film transistor

Thin film transistor (TFT) is a fundamental building block of modern integrated circuits. Oxide semiconductor-based TFTs are an emerging technology to take over the current Si TFT technology because they have superior properties such as larger electron mobility, lower defect density, low temperature fabrication and high optical transparency [520]. In particular, progress in TFTs using transparent amorphous oxide semiconductors—such as InGaZnO_*x*_ (IGZO) [521, 522]—is so remarkable that the application of IGZO-TFTs has started to drive energy-saving and high resolution displays for mobile phones, tablet PCs, PC monitors and large-screen TVs using organic light-emitting diodes. The next challenge in oxide TFTs is application to logic circuits. So far, oxide TFTs work only as unipolar devices mostly *n*-type, or recently realized *p*-type [523], but do not exhibit inversion/ambipolar operation; i.e., complementary circuits cannot be made from TFTs of the same oxide material. Therefore, the next challenge is to realize oxide-based ambipolar TFTs and complementary circuits using only a single oxide semiconductor channel.

**Figure 125.**
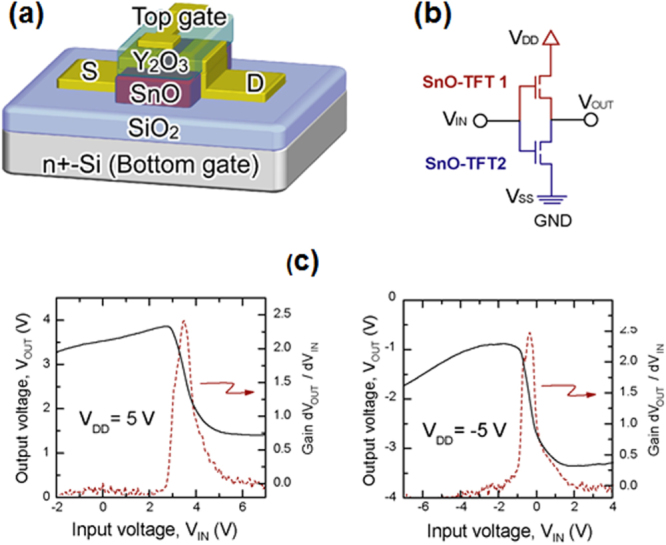
Ambipolar oxide TFTs using SnO. (a) TFT structure, (b) equivalent circuit of SnO complementary metal-oxide semiconductor (CMOS) inverter, and (c) their characteristics [111]. Reprinted with permission from [111]. Copyright 2011 by John Wiley and Sons.

In this project, we succeeded in fabricating an ambipolar oxide TFT using an SnO channel, and demonstrated operation of a complementary-like inverter [111]. It is of interest to note that SnO has the same crystal structure as FeSe, which is a parent compound of IBSCs, and exhibits a insulator*–*metal*–*superconductor transition under high pressure [524]. This is the first success in oxide TFTs. We achieved clear ambipolar operation with saturation mobilities of ∼0.81 for the *p*-channel and ∼10^−3^ cm^2^ (Vs)^−1^ for *n*-channel modes in the SnO TFTs. The maximum voltage gain of ∼2.5 was obtained in the complementary-like inverter circuit as shown in figure 125. This is the first demonstration of a complementary-like circuit using a single oxide semiconductor channel and would provide an important step toward practical oxide electronics. Further, the low temperature process (250 °C at maximum) is compatible with emerging flexible electronics technology.

### 5.3. Metallic ferroelectric materials: slater insulators

In 1995, Anderson and Blount [525] predicted the possibility of a ferroelectric metal in which a ferroelectric-like structural transition occurs in the metallic state. They were motivated by the BCS theory and considered the possibility of such a material which would improve *T*_c_ utilizing strong electron*–*phonon coupling. It is almost a consensus that metals do not exhibit ferroelectricity because static internal electric fields are screened by conduction electrons. In fact, no clear example of such a material had been reported up to 2013. In this project, we could identify this type of material. One is LiOsO_3_ [106] and another is LaFeAsO_0.5_H_0.5_ [211]. Since the latter was described in section 3.1, we focus on the former hereafter.

**Figure 126.**
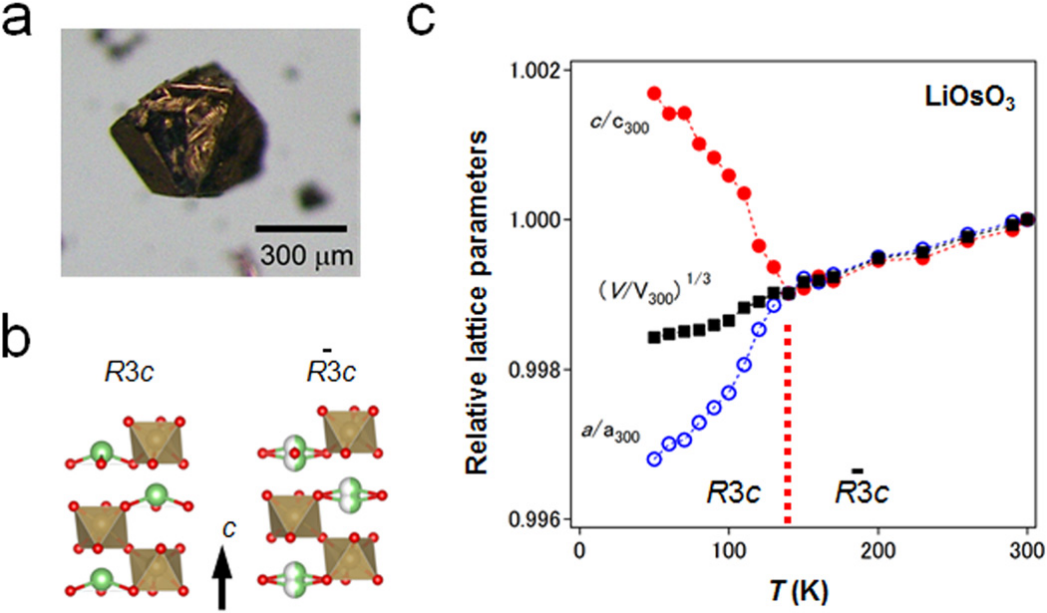
Slater insulator LiOsO_3_. (a) Photo of a flex-grown single crystal, (b) schematic views of centrosymmetric (right) and non-centrosymmetric (left) LiNbO_3_-type structures, (c) thermal evolution of the rhombohedral lattice parameters of LiOsO_3_ measured by neutron diffraction. The parameters are normalized by the 300 K values of *a* = 5.0699(1) Å and *c* = 13.2237(2) Å [106]. Reprinted with permission from Macmillan Publishers Ltd: [106], Copyright 2013.

Yamaura (a member of this project) and his collaborators found [106] that the high-pressure-synthesized material LiOsO_3_ exhibits a structural transition at a temperature (*T*_s_) of 140 K as shown in figure 126. A complementary usage of XRD and neutron diffraction revealed that structural transition at 140 K is due to centrosymmetric (*R*
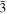
*c*) to non-centrosymmetric (*R*3*c*) phase in metallic LiOsO_3_. This transition is structurally equivalent to the ferroelectric transition of LiNbO_3_ [526], involving a continuous shift in the mean position of Li^+^ ions on cooling below 140 K. Its discovery realizes the scenario suggested by Anderson and Blount, i.e., the existence of ferroelectric-like soft phonons could stabilize non-centrosymmetric superconductivity at enhanced temperatures.

## 6. Perspective on superconducting tapes and wires

Among recently discovered new superconductors, IBSCs exhibit the properties most attractive for tape or wire applications. Although their *T*_c_ of 30–56 K is below liquid nitrogen temperature, their upper critical fields (*H*_c2_) of 60–100 T are comparable to those for cuprate superconductors with *T*_c_ values over 90 K [319]. In particular, 122 compounds, such as Ba-122 doped with K, P, or Co, have very low anisotropy comparable to or lower than that of MgB_2_ [319, 395, 407], implying that high irreversibility fields close to *H*_c2_ or high in-field *J*_c_ are expected. Moreover, 122 compounds were found to have the critical angle for the transition from strong-link to weak-link behavior approximately twice of the value for REBCO, suggesting less sensitivity to grain misorientation at GBs [434].

**Figure 127.**
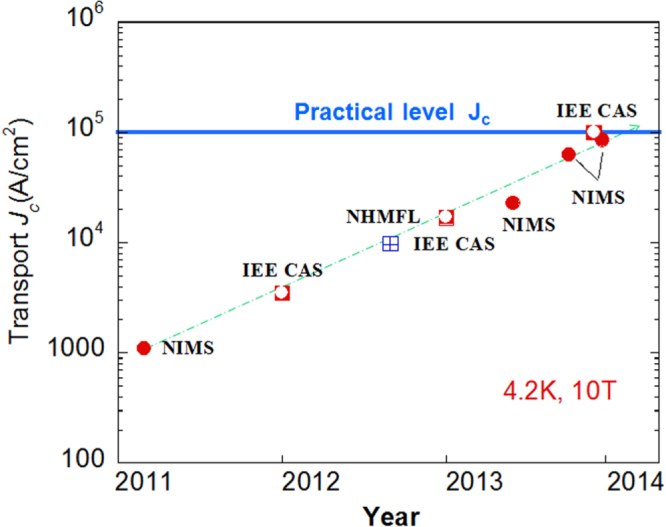
Recent evolution of transport *J*_c_ at 4.2 K, 10 T for Ba(Sr)122:K PIT wires or tapes reported by groups of groups from the National Institute for Materials Science (NIMS), Japan, Institute of Electrical Engineering of the Chinese Academy of Science (IEECAS) and National High Magnetic Field Laboratory (NHMFL), USA.

Actually, the in-field *J*_c_ of Ba(Sr)122:K wires having superconducting cores without biaxial textures which are fabricated by an *ex situ* PIT method has recently been improved greatly, as shown in figure 127. *J*_c_ at 4.2 K and 10 T for Ba(Sr)122:K wires has reached 10^4^ A cm^−2^ through metal addition plus a rolling induced texture process such as hot isostatic or cold press methods [490, 491, 493, 495]. Such mechanical deformation was found to be effective for densifying the conductor core and aligning the grains of the superconducting phase, resulting in high quality superconducting wires. By applying uniaxial pressure of 2–4 GPa, *J*_c_ has been further improved and approached 10^5^ A cm^−2^, which is a practical level as a superconducting wire [503]. In section 4.4, we showed the magnetic field dependence of *J*_c_ at 4.2 K for Ba-122:K wires with a Ag sheath (figure 113), which were fabricated by NIMS group using a uniaxial pressure process and a more practical flat rolling process. The wires fabricated by employing the former process exhibit a *J*_c_ at 10 T close to 10^5^ A cm^−2^ for a single core wire and a high *J*_c_ of 5 × 10^4^ A cm^−2^ even for a 7-filament wire. Though *J*_c_ of the wires through the latter process is still lower, 4 × 10^5^ A cm^−2^ at 10 T, these *J*_c_ values are well over that of the Nb–Ti practical conductor. Moreover, in comparison with the Nb_3_Sn conductor, the *J*_c_ of Ba-122:K tapes through both processes show very small magnetic field dependence, indicating that Ba-122 has great potential to surpass the *J*_c_ performance of Nb_3_Sn in high magnetic fields.

**Figure 128.**
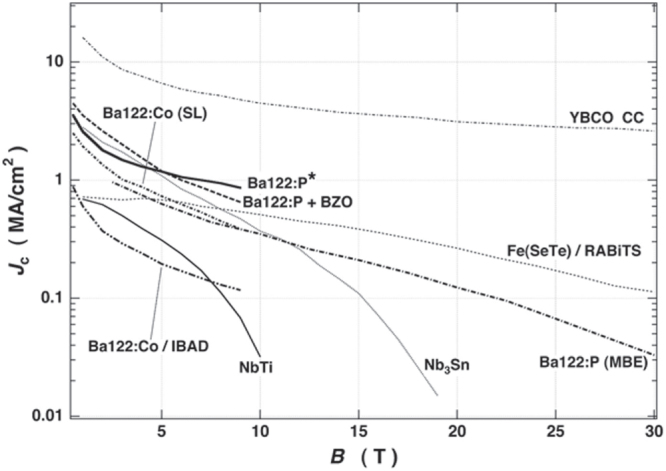
*J*_c_–*B*(//*c*) curves at 4.2 K reported for Ba-122 and Fe(Se,Te) films on single-crystal substrates and some technical substrates such as IBAD and RABiTS (rolling assisted biaxially textured substrates). The data of Nb–Ti and Nb_3_Sn commercial wires and YBCO coated conductors are also shown for comparison. Ba-122:P∗; Tokyo Institute of Technology (TIT) [422], Ba-122:P + BZO; ISTEC [415], Ba-122:Co (SL); Wisconsin Univ. and NHMFL [417], Ba-122:P (MBE); Nagoya Univ. and IFW Dresden [527], Ba-122:Co/IBAD; IFW Dresden [425], Fe(Se,Te)/RABiTS; Brookhaven National Laboratory (BNL), USA and NHMFL [528].

The superior *J*_c_ properties of Ba-122 at higher fields have been demonstrated more clearly in thin film works. In figure 128, *J*_c_–*H* properties at 4.2 K for various Ba-122 films on single-crystal and IBAD–MgO-buffered metal substrates are compared with those for Nb–Ti and Nb_3_Sn conductors. Nb_3_Sn exhibits a steep decrease of *J*_c_ at fields near 20 T, which is close to its *H*_c2_. The Ba-122:P film on MgO fabricated by an MBE method shows *J*_c_ (*H*//*c*) over 10^5^ and 10^4^ A cm^−2^ at 20 and 35 T, respectively [527]. The Ba-122:P films with dense *c*-axis-correlated pinning centers (line dislocations in the mother phase) [422] or BaZrO_3_ nanoparticles [415] by a PLD method exhibit even higher *J*_c_ values at fields below 9 T and a rather slow decay, suggesting that their *J*_c_ values at higher fields would be higher than that for the MBE film. Another significance of these results is that the in-field performance of IBSCs, in particular Ba-122, can be remarkably improved by introduced nanometer-size vortex pinning centers, as already demonstrated in REBCO. This would be true not only for films or coated conductors but also for PIT wires. Although the in-field *J*_c_ performance of Ba-122 coated conductors, in particular longer-length ones, is still substantially lower than that for high-quality films on single crystal substrates, this difference could be mainly attributed to technical problems and should be overcome in the near future. In fact, for the case of 11 compound Fe(Se,Te) of which films can be grown at lower substrate temperatures, coated conductors, not long, with *J*_c_ over 10^5^ A cm^−2^ at 30 T have been demonstrated [528].

The superior *J*_c_ performance of IBSCs such as Ba-122 mentioned above indicates that their most promising application would be wires or tapes for magnets used at low temperatures and high fields well over 20 T. The competitive candidate for such applications is REBCO coated conductors, which exhibit even higher in-field *J*_c_ at low temperatures. However, one of the current critical issues of REBCO coated conductors for this application is suppression of shielding current, which is required to generate stable and highly precise fields [529]. In order to overcome this difficulty, the fabrication technique for narrow multi-filamentary structures is being developed [530]. Another problem is the mechanical strength against delamination which originates from its multilayer structure [531]. If the *J*_c_ performance of iron-based multifilamentary PIT wires is further improved by refining the fabrication process and reaches a practical level even at fields of 20–30 T, high *J*_c_ in higher magnetic fields of IBSCs could be a concrete advantage over REBCO coated conductors, though there are some further issues such as the sheath material with higher mechanical strength and lower cost. Concerning iron-based coated conductors, more technical challenges still remain to demonstrate an advantage over practical conductors, especially REBCO coated conductors. These include high in-field *J*_c_ on less-textured buffer layers, narrow and thicker conductors with a lower aspect ratio, higher mechanical strength, and so on. Although coated conductors have been fabricated using the same oxide buffer-layer technology as for REBCO, special buffer-layer materials more compatible with IBSCs would be necessary to demonstrate their own potential.

Another challenge common to PIT wires and coated conductors is the development of IBSCs with less toxic elements, which would further stimulate their application. Considering the history that it needed more than 20 years to develop commercial REBCO coated conductors, further continuing research and development is definitely required to realize practical wires or tapes based on IBSCs discovered only seven years ago. Fortunately, the material variety of IBSCs is the largest among all the superconductor families, and the discovery of new types of superconducting materials has been continuing to date. The intrinsic nature of this materials system [508, 532] is to have a Fermi surface composed of multi-pockets arising from multi-orbitals of Fe 3*d*. This nature provides a wide opportunity in which various degrees of freedom can contribute to the emergence of superconductivity. Recent research has clarified the contribution of orbital [533], charge [534] and phonons [535] in addition to spin [159, 160], which was initially proposed. The current status may be regarded as ‘actors are ready!’. These actors may work cooperatively to raise *T*_c_, which has been experimentally suggested recently. We may expect discoveries of new IBSCs which exhibit higher *J*_c_ keeping less anisotropy from the background. *Iron is still hot*!
